# The Ophthalmology Mini-Elective Gives Vision to Preclinical Medical Students

**DOI:** 10.15766/mep_2374-8265.11024

**Published:** 2020-11-23

**Authors:** Peter Mortensen, Rikki Enzor, Kevin Keppel, Ryan Williamson, Peter Jones, Gideon Nkrumah, Zaid Safiullah, Sarah Michelson, Sameera Nadimpalli, Ann Shue, Evan Waxman

**Affiliations:** 1 Ophthalmology Resident, Department of Ophthalmology, University of Pittsburgh Medical Center; 2 Preliminary Year Resident, University of Pittsburgh Medical Center; 3 Medical Student, University of Pittsburgh School of Medicine; 4 Ophthalmology Resident, Department of Ophthalmology, University of Michigan Medical School; 5 Ophthalmology Resident, Department of Ophthalmology, Northwestern University Feinberg School of Medicine; 6 Clinical Assistant Professor, Department of Ophthalmology, Stanford University School of Medicine; 7 Residency Program Director and Associate Professor, Department of Ophthalmology, University of Pittsburgh Medical Center

**Keywords:** Ophthalmology, Mentoring, Eye Examination, Wet Lab, Elective Course

## Abstract

**Introduction:**

Ophthalmology education during medical school is often very limited. To provide exposure to areas beyond its standard curriculum, the University of Pittsburgh School of Medicine offers mini-elective courses in various disciplines. We developed such a course to provide instruction in the basics of clinical ophthalmology to interested preclinical medical students.

**Methods:**

First- and second-year medical students electively enrolled in our course (mean number of students per year = 12), which included four sessions combining didactics and hands-on learning. Additionally, each student individually spent time with an ophthalmologist in the operating room. Our course was held each year from 2015 to 2019.

**Results:**

Participants completed pre- (*n* = 25) and postsurveys (*n* = 20), reflecting increased comfort with the ophthalmologic history and physical examination. In 2019, participants also completed pre- and posttests, demonstrating increased knowledge of ophthalmology.

**Discussion:**

The Ophthalmology Mini-Elective is a unique educational tool that introduces the principles of ophthalmology to preclinical medical students, addressing an area of medicine that is generally minimally included in the required curriculum.

## Educational Objectives

By the end of this activity, learners will be able to:

1. Describe the basic components of an ophthalmic history, including the chief complaint, history of present illness, past ocular history, and a pertinent review of systems.
2. Perform an eye examination, including the eye vitals, slit lamp exam, and fundoscopic exam.
3. Discuss common ocular pathologies and their management.
4. Present an ophthalmic patient utilizing appropriate format.

## Introduction

Ophthalmology plays a limited role in the curriculum of most medical schools, resulting in medical school and residency graduates who lack the skills necessary to care for patients with ophthalmic diagnoses.^1^ Primary care and emergency physicians are frequently the first to see patients with eye-related complaints.^2^ For this reason, the Association of University Professors of Ophthalmology (AUPO) outlined its Policy Statement on Medical Student Education in 1990, providing a minimum standard for skills in eye-related diagnosis and management that should be met by all physicians.^1^ The AUPO policy statement includes assessing visual acuity, pupils, and ocular motility and alignment; evaluating a red or traumatized eye; performing direct ophthalmoscopy; and initiating management and/or referral of a patient with an ophthalmologic problem.^1^ Updates to the AUPO standards were provided by Mottow-Lippa in 2009 and by Graubart and colleagues in 2018.^3,4^ According to a survey of residency program directors in primary care specialties including family medicine, pediatrics, and internal medicine, less than half of their incoming residents met the AUPO standards, although 85% of the surveyed residency program directors expected this training to occur during medical school. The same survey found that upon graduation from residency, one-third of internal medicine residents still did not meet AUPO standards.^1^

The goal of preclinical curricula is to provide a foundational knowledge of medicine, including building strong history and physical examination skills. Information regarding many clinical disciplines must be covered; thus, there is insufficient time to cover every area of medicine in depth. Ophthalmology coverage has diminished in the preclinical curricula of most medical schools according to a survey of U.S. and Canadian medical schools conducted in 2012–2013.^5^ While ophthalmology was once a required clinical rotation at a majority of medical schools, most North American medical school graduates are now completing medical school without exposure to ophthalmology during their clinical years.^6,7^ A highly technical field, ophthalmology requires a unique set of physical examination skills, and evidence in the literature indicates that repeated teaching over time produces more durable learning.^8^ This increases our degree of concern about the diminishing emphasis on ophthalmology during the preclinical years of medical school, particularly for students who may ultimately enter primary care specialties.

While a body of resources exists for teaching ophthalmologic examination skills to residents, resources focused on medical students are limited. A search for the keyword *ophthalmology* in *MedEdPORTAL* yielded nine publications pertaining to ophthalmology. Eight focus on graduate medical education programs, with just two of these applying to nonophthalmology residents.^9,10^ Only one resource in *MedEdPORTAL* aims to teach ophthalmic techniques to medical students, providing a curriculum for teaching direct ophthalmoscopy.^11^ A broader search of the educational literature found several articles about teaching direct ophthalmoscopy to medical students in the preclinical and clinical stages of training.^12–15^ Additionally, we found two articles describing intensive single-day courses in ophthalmology, both offering didactic learning in the morning and practice of examination skills in the afternoon.^16,17^ While similar to our own course, both courses have been designed for medical students in their clinical years of training.

The University of Pittsburgh School of Medicine (UPSOM) has developed mini-elective courses for medical students to explore areas of medicine falling outside the core curriculum. These courses aim “to provide well-structured, rigorous and high quality experiences in areas not typically available to students”^18^ Currently, UPSOM offers 52 mini-elective courses covering a broad range of topics, including refugee health, vascular surgery, urology, and sports medicine. These courses typically occur during the spring term of the preclinical years and consist of four to eight evening sessions.^18^

At UPSOM, the Ophthalmology Mini-Elective (OME) is part of a larger curriculum in ophthalmology. Preclinical medical students participate in required lectures about neuro-ophthalmology as part of their neuroscience course. The Ophthalmology Interest Group offers optional lectures to supplement the required preclinical curriculum, providing case-based teaching on topics such as neuro-ophthalmology, infectious diseases in ophthalmology, and ophthalmologic manifestations of systemic diseases. Among the core clinical clerkships, UPSOM provides a required weeklong experience in clinical ophthalmology. During this week, medical students are placed in a comprehensive eye clinic and learn to independently perform the basic ophthalmology history and physical examination (including eye vitals, slit lamp examination, and direct ophthalmoscopy). The students' clinical time is supplemented with ophthalmology lectures.^19^ Of note, our institution is among the few that still requires a clinical rotation in ophthalmology, and this rotation is specifically designed to address the AUPO standards.

Interestingly, our OME course was heavily drawn from a predecessor course, which was developed to support the efforts of our robust clinical ophthalmology volunteer program. The Guerrilla Eye Service (GES) is a free, mobile eye clinic that provides ophthalmology care to underserved patients in Pittsburgh and the surrounding communities.^20^ While GES draws volunteers from all levels of training, the majority of our volunteers are in their preclinical years. Medical students run the clinic, with the supervision and support of ophthalmology residents and attendings. Participating students learn how to take a thorough ophthalmic history and perform a basic ophthalmologic examination, including assessment of visual acuity, intraocular pressure, pupils, confrontation visual fields, and motility; slit lamp examination; and direct ophthalmoscopy. In 2010, a mini-elective course titled GES Boot Camp was offered at UPSOM to supplement medical students' learning in GES, with an emphasis on ophthalmology physical examination skills. Lectures were given addressing the most frequently encountered pathologies at GES, including refractive error, diabetic retinopathy, glaucoma, narrow angles, and cataract.^20^ Our current OME course is largely based on the previously offered GES Boot Camp.

UPSOM has offered the OME for preclinical medical students annually for the past 5 years. While the required ophthalmology clerkship addresses AUPO standards, the OME has been designed to play a broader role in providing medical students with basic skills needed in the ophthalmology clinic, primary care setting, and emergency department. The OME consists of brief lectures followed by small-group sessions devoted to ophthalmologic physical examination techniques. Given the importance of history and physical examination skills in preclinical training, this is the major focus of our course. Here, we present the components of the OME so that interested parties can offer a similar course at their own institutions.

## Methods

The target audience for the OME was first- and second-year medical students. No prior knowledge or experience was required. Students who completed all course requirements earned a certificate of completion at the conclusion of the course. The OME consisted of 2-hour sessions held weekly for 4 consecutive weeks. Each session began with a lecture for 30–45 minutes and concluded with hands-on learning in small groups for the remainder of the 2 hours. Prior to each lecture, learners reviewed relevant chapters from the free online textbook OphthoBook.^21^ Following the conclusion of the didactic sessions, medical students also shadowed an ophthalmologist in the operating room.

Instructors for the course varied each year but generally included an ophthalmology faculty member, ophthalmology residents at all levels of training, and third- and fourth-year medical students. Instructors for the course were expected to have an understanding of ocular anatomy and the components of the ophthalmologic examination, including ocular vital signs (visual acuity, pupillary examination, intraocular pressure, motility, and confrontation visual fields), anterior segment examination using a slit lamp microscope, and ophthalmoscopy. Prior to the course, instructors familiarized themselves with the course syllabus (Appendix A) and an introduction for instructors of the course (Appendix B). Prior to each session, instructors reviewed the corresponding weekly course time line and objectives (Appendix C), which outlined the recommended time to allot to each educational activity as well as the required materials for individual sessions. Each of the four sessions also had a corresponding set of PowerPoint slides (Appendices D–G) that instructors reviewed prior to teaching the session. In our course, the lectures were prepared and presented by senior medical students and ophthalmology residents.

Generally, we began each session with 30–45 minutes of case-based teaching and discussion using a PowerPoint format. The first session started with a brief introduction and course outline by the ophthalmology faculty mentor. Next, we administered a precourse survey (Appendix I) and content-based pretest (Appendix J) to assess participants' goals for the course, monitor course feedback, and assess participant learning. Afterwards, we presented the Session 1: Introduction to Ophthalmology PowerPoint (Appendix D) to introduce learners to the ophthalmic history and physical examination, as well as the different subspecialties in ophthalmology. We devoted the remaining time for the first session to hands-on learning in which groups of one instructor and three to four participants practiced assessing the vital signs of the eye, including visual acuity, intraocular pressure, pupillary examination, motility, and confrontation visual fields (Appendix D). During the second and third sessions, we devoted 45 minutes to case-based discussions of the anterior segment (Session 2, Appendix E) and posterior segment (Session 3, Appendix F), followed by instruction in slit lamp examination and ophthalmoscopy, respectively. The fourth and final session consisted of a case-based presentation on ocular trauma and eye emergencies (Appendix G), followed by a wet lab experience utilizing pig eyes. During the wet lab, instructors taught learners to complete a modified extracapsular cataract extraction by creating a paracentesis at the corneal limbus, filling the anterior chamber with ophthalmic viscoelastic material, performing a can-opener capsulotomy, and enlarging the paracentesis into a sizable corneal incision to remove the intraocular lens (Appendix H).

The course required a room with audiovisual equipment for the lecture component of each of the four course sessions. For hands-on teaching, the first three sessions required ophthalmology clinic rooms, with access to slit lamps, direct ophthalmoscopes, and other standard examination equipment (see Appendix C for specifics for each session). The fourth and final session required access to a laboratory space, pig eyes, surgical instruments, and surgical microscopes. After the course, each medical student observed in the operating room, as a required component of the course.

We administered surveys to medical students before and after completion of the mini-elective in 2016, 2017, and 2019 (Appendices I and K). The surveys included a series of questions regarding students' interest in and comfort with ophthalmology, with responses using a 5-point Likert scale (1 = *not at all,* 5 = *very*). On the administered version of the survey, scores of 2–4 were not assigned words to describe the numerical values; on the version provided in Appendices I and K, these values have been assigned verbal descriptions. For 2019, medical students also completed a 15-question multiple-choice pre- and posttest (Appendix J), which we administered at the start of the first session and end of the fourth session. The test scores were reported as a percentage correct out of 15 (pre- and posttest answers and explanations are provided in Appendix L). We compared the median responses of pre- and postsurveys using an unpaired Mann-Whitney *U* test (we could not reliably pair these surveys) and used a paired Wilcoxon signed rank test to assess significance of differences between pre- and posttest results. A bootstrap procedure with 1,000 repetitions was used to compute 95% confidence intervals for mean survey responses and test scores.

The postcourse survey also included three open-ended course assessment questions on which we performed a qualitative analysis (Table 1). We grouped the responses into thematic categories for each question and report the percentages for each category. When a response could be categorized into multiple groups, we did so. For example, a response of “More hands-on experiences” was included in both the physical exam practice and the surgical practice categories.

**Table 1.**
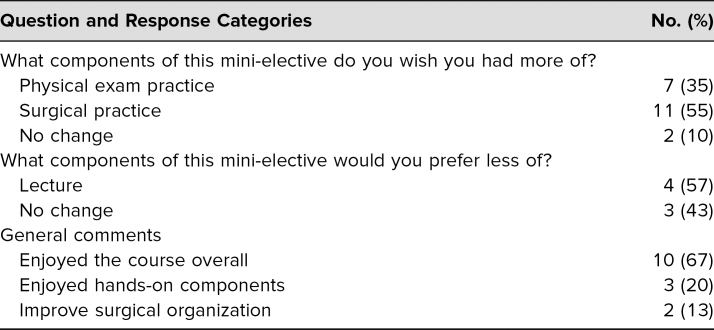
Analysis of Postsurvey Qualitative Responses (*N* = 20)

To determine whether the OME impacted the number of graduates who matched into ophthalmology, we referred to the match lists and OME enrollment logs. The OME was first held in 2015 for first- and second-year medical students. Thus, we began evaluating for graduates matching into ophthalmology in 2017.

## Results

The number of students enrolled in our course ranged from six to 21 per year, with a mean of 12 students per year. In the years 2016, 2017, and 2019, we administered pre- and postsurveys to all enrolled students, including seven students in 2016, six students in 2017, and 15 students in 2019, with one enrolled student not completing the course. We administered pre- and postsurveys to 28 students and 27 students, respectively (Table 2); 25 presurveys (89%) and 20 postsurveys (74%) were returned. Students responded to questions on a 5-point Likert scale (1 = *not at all,* 5 = *very*; scores 2–4 were shown but not described). Survey responses (Table 3) demonstrated a significant increase in interest in ophthalmology as a career (*p* = .007) and indicated that students felt more confident in their fund of ophthalmic knowledge (*p* < .001). Students also expressed increased comfort with fundamental skills, including obtaining a history, performing an examination, and presenting to attendings. In posttest surveys, we included an additional question asking if the mini-elective met students' expectations, and all 20 surveyed students responded with a score of 5.

**Table 2.**
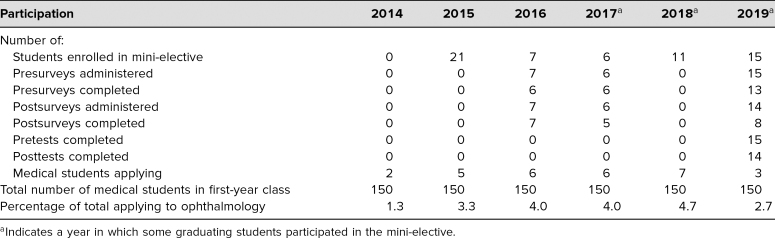
Mini-Elective Participation by Year

**Table 3.**
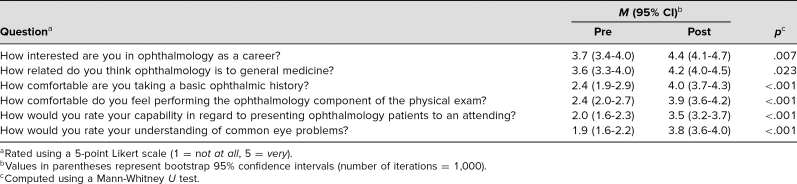
Survey Administered to Students Before (*N* = 25) and After (*N* = 20) the Mini-Elective

In addition to the abovementioned quantitative responses, we also asked students a series of open-ended questions (Table 1). Typically, students expressed that they wanted more hands-on practice with examination techniques (41%) and the surgical lab (65%). A small number of students requested less lecture time (57%) or improved organization of the surgical experience (13%). Overall, students expressed that they enjoyed the course (67%), especially the hands-on experiences (20%). A selection of students' quotes is listed below by question.

• What components of this mini-elective do you wish you had more of?
  ○ “Physical examination practice!”
  ○ “More hands-on experience!”
  ○ “Loved the indirect ophthalmoscope (wanted more time).”
  ○ “A lecture on surgical procedures before the eye surgery day.”
• What components of this mini-elective would you prefer less of?
  ○ “Smaller presentation time in the first session.”
  ○ “Lecture (maybe shorten to 30 minutes of lecture and 90 minutes of clinical).”
• General comments.
  ○ “I loved this elective and the balance of ophthalmology exam practice and wet lab with lectures.”
  ○ “Wish there were more surgical tools when we did the surgeries on the pig eyes, because I spent a good portion of it just sitting around waiting for a tool. Wish there was more guidance before it, too.”
  ○ “I learned a lot in this elective, much more than I previously knew. I found interactions in small groups with residents most helpful.”

To assess knowledge, we administered a test with 15 multiple-choice questions to our 2019 mini-elective cohort (*n* = 14). The average score increased significantly between pre- and posttests: pretest *M* = 49.0% (95% CI, 43.3%-54.3%), posttest *M* = 71.0% (95% CI, 67.6%-75.2%), score difference (posttest minus pretest) = 21.9% (95% CI, 15.2%–29.0%), *p* = .002 paired Wilcoxon signed rank test.

## Discussion

The OME aims to teach preclinical medical students the ophthalmologic history and examination and to familiarize them with the diagnosis and management of common ophthalmic pathologies. In setting these goals, we attempted to supplement related endeavors at our institution that collectively prepare students to manage ophthalmic issues in primary care, emergency, and specialty care settings. To achieve these goals, we paired didactic sessions with hands-on learning and a surgical shadowing experience.

To evaluate the efficacy of our course, we administered pre- and postsurveys to gauge student learning. At the conclusion of the course, participants reported that they felt more comfortable with the ophthalmologic history and examination and that their knowledge of ophthalmology had improved. In 2019, we conducted knowledge-based pre- and posttests, which showed a dramatic improvement in learners' understanding of ophthalmology, supporting students' perceptions of an improved knowledge base. In postsurvey feedback, the learners consistently mentioned hands-on experiences, including small groups focused on examination skills and the wet lab, as positive elements of our course. Learners also voiced appreciation of the opportunity to gain early exposure to ophthalmic surgery.

The OME has evolved over several years based on feedback collected in pre- and postsurveys. When we first offered the course, each session consisted of 1 hour of lecture and 1 hour of small-group learning or wet lab. Based on students' comments that they would like more time devoted to physical examination techniques and the wet lab, we increased the amount of time for these activities, with the goal of reserving 30–45 minutes for lectures and assessments at the start of each session. The majority of constructive feedback in 2019 centered on improving the efficiency and organization of the wet lab. To address these suggestions, we developed a detailed wet lab handout that can be used by instructors and participants in the wet lab in the future (see Appendix H). Overall, postsurvey comments indicate that students are generally satisfied with the course.

It remains unclear whether the OME has encouraged additional students to pursue the field of ophthalmology. Given the small number of students who apply for ophthalmology annually, the individual numbers fluctuate significantly from year to year. Furthermore, the goal of the course is not primarily to encourage students to apply for ophthalmology residency programs but to improve their ability to diagnose and manage common ophthalmologic conditions that may be encountered across a broad range of health care settings.

In addition to learners, teachers of the OME have reported benefiting from their participation in the course. Over time, the course has developed a system of multilevel mentoring, with both senior residents and senior medical students playing unique roles. The senior resident leadership has participated in course design, recruitment of additional resident volunteers as small-group teachers, and mentoring of senior medical students. The senior medical student leaders have prepared and taught the didactic sessions, as well as participating in teaching the small-group sessions. As participants in the leadership team for this course, we believe that our experiences with the OME have made us more comfortable teaching the basics of ophthalmology. Thus, the OME offers valuable educational opportunities for both teachers and learners.

Due to our institution's large ophthalmology department and preexisting student engagement in our volunteer eye clinic (GES), the challenges that we faced in implementing this course were largely logistical and curricular. Each year, dates must be selected based on the preclinical medical students' schedules, while also considering availability of clinical medical student, resident, and faculty volunteers. Notably, the timing of USMLE Step 1 examinations at our institution has resulted in a majority of first-year medical student participants in our course. Regarding the curriculum, the main challenges have been choosing what to teach in a limited amount of time and how to divide the time between didactic and hands-on experiences. Enrollment in our course depends heavily on student interest, with the number of students varying dramatically from year to year.

A significant benefit of our course is its generalizability to other medical schools. However, there may be challenges to applying our course in a setting with different available resources. Our institution has a large ophthalmology department with 18 residents. Each year, one or two residents are the leadership team and attend all four teaching sessions, with a handful of additional residents volunteering to attend individual sessions to lead the small groups in learning ophthalmologic examination or surgical skills. We anticipate that medical schools with an ophthalmology department and large residency program could readily develop and implement a similar program. However, an institution with a smaller residency program may require increased commitment by residents to successfully implement such a course. We acknowledge that a program like ours may be difficult in a setting without an ophthalmology department and/or residency program. Yet these institutions may be able to provide such a program for interested students if they develop collaborations with community-based ophthalmologists supportive of such an endeavor.

Many limitations surround the methods of evaluating our course. In the 3 years that surveys were administered, a total of 28 students participated in the course. Among these students, 89% completed a presurvey, and only 74% completed a postsurvey. There was minimal attrition, with only one student failing to complete the course. However, it was difficult to obtain postsurveys from students after the completion of the course. To ensure that more postsurveys are obtained in future years, we will administer surveys in person and/or require students to complete the postsurvey prior to issuing credit for the course. One weakness of our current pre- and postsurveys is that they inquire about interest in ophthalmology as a career but do not gauge interest in primary care and other medical or surgical specialties. We plan to add this question to surveys administered in the future.

Our pre- and posttests contained 15 questions. A longer test could more adequately assess students' knowledge. However, participating students took approximately 20 minutes to complete the 15-question tests, reducing the available time for teaching during two sessions. Administering these tests in person ensured that the students' answers would accurately reflect their knowledge about ophthalmology. However, we would consider a take-home test in the future, given the elective nature of this course, the increased flexibility regarding the length of the test, and the ability to preserve in-person time with students for teaching.

Another limitation is the lack of a standardized assessment of students' ophthalmology physical examination skills at the end of the course. Students reported improved subjective comfort with the ophthalmology physical examination in the pre- and postsurveys. We may consider adding a standardized assessment of ophthalmology physical examination skills to future courses to better evaluate students' learning during the small-group sessions. Because many medical students participate in both the OME and GES, we may consider evaluating whether students' physical exam skills improve during the GES missions after participation in our course.

Our wet lab session gives medical students the opportunity to perform intraocular surgery under a surgical microscope and to examine the anatomy of a porcine eye, with slightly over an hour dedicated to the wet lab. We considered teaching students to suture under a surgical microscope. However, most preclinical medical students at UPSOM have not yet learned how to place sutures. Previous experience with beginning ophthalmology residents at our institution demonstrated that even with prior suturing experience, 2–3 hours in the wet lab was often warranted for a microsurgical suturing lab. Thus, we focused on teaching basic intraocular surgical skills while working under a surgical microscope. In 2019, all 14 participants in our novel cataract surgery–focused wet lab were able to successfully remove the lens from a porcine eye. After completion of our modified cataract surgical procedure, many students proceeded to dissect the eye in order to identify additional intraocular structures. Thus, we believe that our current wet lab design is well suited to preclinical medical students learning about ophthalmology. While our residency program has a designated laboratory with surgical microscopes, any laboratory space could be adapted to provide a similar wet lab experience to medical students. In a setting where surgical microscopes are unavailable, creative solutions might include the use of high-plus reading glasses for surgical loupes and bicycle headlamps as a light source.

The OME covers a broad range of ophthalmic content. Given its limited time frame, its scope is accordingly limited. Our institution's required preclinical lectures and required ophthalmology clinical clerkship intentionally strive to help each student meet AUPO guidelines regarding the acquisition of ophthalmology-related skills during undergraduate medical training. Our preclinical mini-elective course attempts to supplement these curricular requirements by strengthening students' ophthalmologic history and physical examination skills and promoting knowledge of common eye-related problems that may be seen in a variety of clinical settings. We do not cover many topics in neuro-ophthalmology, such as optic neuritis, ischemic optic neuropathies (including giant cell arteritis), and thyroid eye disease. In our institution, these topics are covered extensively during students' preclinical neurology block and neurology clerkship. For medical schools that do not cover these topics in the required curriculum, an additional session dedicated to neuro-ophthalmology would be a useful addition to the mini-elective curriculum. In conclusion, we find that the OME has provided meaningful teaching to preclinical medical students at UPSOM, and we offer our course design here as a template for other medical schools wanting to provide a similar learning experience.

## Appendices

Course Syllabus.docxInstructor Introduction.docxWeekly Course Time Line & Objectives.docxSession 1 - Intro to Ophthalmology.pptxSession 2 - Anterior Segment.pptxSession 3 - Posterior Segment.pptxSession 4 - Eye Emergencies and Trauma.pptxLaboratory Session Guide.pdfPrecourse Survey.docxPre- and Posttest.docxPostcourse Survey.docxPre- and Posttest Answers.docx
All appendices are peer reviewed as integral parts of the Original Publication.
