# Supplementary material for: The Ophthalmology Mini-Elective Gives Vision to Preclinical Medical Students
Source: MedEdPORTAL. 2020 Nov 23;16:11024. doi: 10.15766/mep_2374-8265.11024 (PMC7703479; doi:10.15766/mep_2374-8265.11024)
Supplement: Supplementary file 1 — Course Syllabus.docxInstructor Introduction.docxWeekly Course Time Line & Objectives.docxSession 1 - Intro to Ophthalmology.pptxSession 2 - Anterior Segment.pptxSession 3 - Posterior Segment.pptxSession 4 - Eye Emergencies and Trauma.pptxLaboratory Session Guide.pdfPrecourse Survey.docxPre- and Posttest.docxPostcourse Survey.docxPre- and Posttest Answers.docx [file mep_2374-8265.11024-s001.zip › A. Course Syllabus.docx]

**Ophthalmology Mini-Elective Syllabus**

**Maximum Students:** 20

**Class Year:** MS1 and MS2

**Prerequisites:** None

**Requirements for Certificate:**

- Attendance and participation in all four sessions
- One day of shadowing in the OR (students will be provided with a schedule of options)
- Review all assigned readings in *OphthoBook^^[[1]](#footnote-1)^^* (free online resource)

**Course Description:**

Ophthalmology is a unique medical field to which students receive little exposure during medical school. However, eye complaints are prevalent in many clinical settings, and it is highly beneficial to have some knowledge about the most common eye problems, regardless of future specialty. In this elective, we will teach students about the ophthalmic history and physical examination, and we will provide a basic introduction to the techniques of the slit lamp examination and ophthalmoscopy. Students will also discuss common ocular pathology, such as cataracts, glaucoma, conjunctivitis, and diabetic retinopathy in a case-based format. Additionally, students will have the opportunity to gain exposure to ophthalmic surgery by spending time in the operating room. At the end of the mini-elective, we hope that students will have gained a strong foundation in basic ophthalmology.

**Course Objectives:**

By the completion of this mini-elective, students will be able to:

1. Describe the basic components of an ophthalmic history, including the chief complaint, history of present illness, past ocular history, and a pertinent review of systems
2. Perform proper eye examination techniques, including eye vitals, slit lamp exam, and funduscopic exam
3. Discuss common ocular pathologies and their management
4. Present an ophthalmic patient utilizing appropriate format

**Course Outline:**

1. **Session 1: Introduction to the Ophthalmic History and Physical Examination**
   1. The session will start with an overall introduction to the field of ophthalmology and its subspecialties. Students will be taught the basics of the ophthalmic H&P and will practice the exam on each other. The proper format of ophthalmology presentations will also be discussed.
   2. **Recommended reading**: *OphthoBook* Chapters 1, 2
2. **Session 2: Anterior Eye Pathologies**
   1. Students will discuss cases related to the anterior segment of the eye, such as cataracts, glaucoma, and corneal ulcers. Students will be taken to clinic rooms to learn how to perform slit lamp examination and, time permitting, refraction.
   2. **Recommended reading**: *OphthoBook* Chapters 3, 10
3. **Session 3: Posterior Eye Pathologies**
   1. Students will discuss cases related to the posterior segment of the eye, such as retinal detachment and diabetic retinopathy. Students will be taken to clinic rooms to learn how to perform the dilated fundus examination on each other, which will involve each student having one eye dilated.
   2. **Recommended reading**: *OphthoBook* Chapter 4
4. **Session 4: Eye Emergencies**
   1. Students will discuss the evaluation of traumatic eye injuries and simple eye infections. Students will then participate in a hands-on surgical simulation activity in the wet lab.
   2. **Recommended reading**: *OphthoBook* Chapters 5, 8
5. **Operating Room Experience (students will individually schedule)**
   1. Students will be paired with a current ophthalmology resident to shadow in the operating room. Alternatively, students can opt to be provided with a list of available attending surgeons whom they can contact and arrange a time to observe on their own.
   2. **Recommended reading**: Review material related to the scheduled case(s)

1. Root T. OphthoBook - the free ophthalmology textbook for new students. Tim Root - Virtual Eye Professor. https://timroot.com/ophthobook/. Published 2017. Accessed August 3, 2018. [↑](#footnote-ref-1)
