# Supplementary material for: The Ophthalmology Mini-Elective Gives Vision to Preclinical Medical Students
Source: MedEdPORTAL. 2020 Nov 23;16:11024. doi: 10.15766/mep_2374-8265.11024 (PMC7703479; doi:10.15766/mep_2374-8265.11024)
Supplement: Supplementary file 1 — Course Syllabus.docxInstructor Introduction.docxWeekly Course Time Line & Objectives.docxSession 1 - Intro to Ophthalmology.pptxSession 2 - Anterior Segment.pptxSession 3 - Posterior Segment.pptxSession 4 - Eye Emergencies and Trauma.pptxLaboratory Session Guide.pdfPrecourse Survey.docxPre- and Posttest.docxPostcourse Survey.docxPre- and Posttest Answers.docx [file mep_2374-8265.11024-s001.zip › B. Instructor Introduction.docx]

**Introduction for Instructors of the Ophthalmology Mini-Elective**

Instructors of this elective may include attendings, senior residents, junior residents, and senior medical students. This document will serve as an overall guideline for how to teach this course.

The Ophthalmology Mini-Elective is designed to provide an introduction to the field of ophthalmology to preclinical medical students with very little exposure to this specialty at this point in their training. Thus, instructors will cover introductory concepts like the ophthalmic history and exam, common eye diseases, and methods of medical and surgical treatment.

The elective comprises four formal sessions, with each individual session including a didactic component and a hands-on activity. Students are expected to complete pre-reading before each session so that the didactic component can be kept short, allowing more time for hands-on practice. Instructors are advised to send out reading materials 5-7 days in advance of each session to give students ample time to build a relevant knowledge base.

Instructors are encouraged to use the *Weekly Course Timeline and Objectives* document for specific guidance on each session. This document includes timelines and lists of necessary materials. In general, one or two presenters (usually senior medical students) begin the session by teaching from the designated PowerPoint. This is followed by hands-on practice in small groups, with each group led by one or two instructors (usually residents). If possible, it is ideal to have at least one resident available per group of 2-3 first-year medical students.

The *fourth* small group session will be unique in that it gives students an opportunity to practice surgical techniques in the wet lab. This will require more in-depth planning to ensure the workspaces and materials will be available. Guidelines and materials for an exemplary wet lab session are listed in the *Laboratory Session Guide*, but the actual logistics and procedures may vary between institutions depending on available resources.

Finally, in addition to the four formal sessions, the Ophthalmology Mini-Elective offers an individually arranged *fifth* “session” during which students shadow an ophthalmologist in the operating room. This may also vary between institutions, but instructors are advised to compile a schedule of recommended OR days that spans several weeks following the final formal session. This can be provided to students, who can then “sign up” to follow a surgeon on a specific date. Students should be encouraged to contact the ophthalmologist directly to confirm their shadowing time and to read about the scheduled procedures in advance.

As instructors and coordinators of this course for the last several years, we have truly enjoyed our time introducing first-year medical students to the fascinating field of ophthalmology. In our experience, the vast majority of students that take this course are eager to learn, regardless of their future career plans. Still, ophthalmology is complex and at times difficult to teach and learn; this course was not an immediate success, but we were able to make significant improvements by listening to the feedback of our students. As a result, we have learned the importance of clinical examples and hands-on practice to maximally enrich the educational experience, and we have implemented changes to achieve that effect. We are confident that a dedicated and enthusiastic approach to this teaching opportunity will lead to the general success of this curriculum at institutions beyond our own.
