# Supplementary material for: The Ophthalmology Mini-Elective Gives Vision to Preclinical Medical Students
Source: MedEdPORTAL. 2020 Nov 23;16:11024. doi: 10.15766/mep_2374-8265.11024 (PMC7703479; doi:10.15766/mep_2374-8265.11024)
Supplement: Supplementary file 1 — Course Syllabus.docxInstructor Introduction.docxWeekly Course Time Line & Objectives.docxSession 1 - Intro to Ophthalmology.pptxSession 2 - Anterior Segment.pptxSession 3 - Posterior Segment.pptxSession 4 - Eye Emergencies and Trauma.pptxLaboratory Session Guide.pdfPrecourse Survey.docxPre- and Posttest.docxPostcourse Survey.docxPre- and Posttest Answers.docx [file mep_2374-8265.11024-s001.zip › C. Weekly Course Time line & Objectives.docx]

**
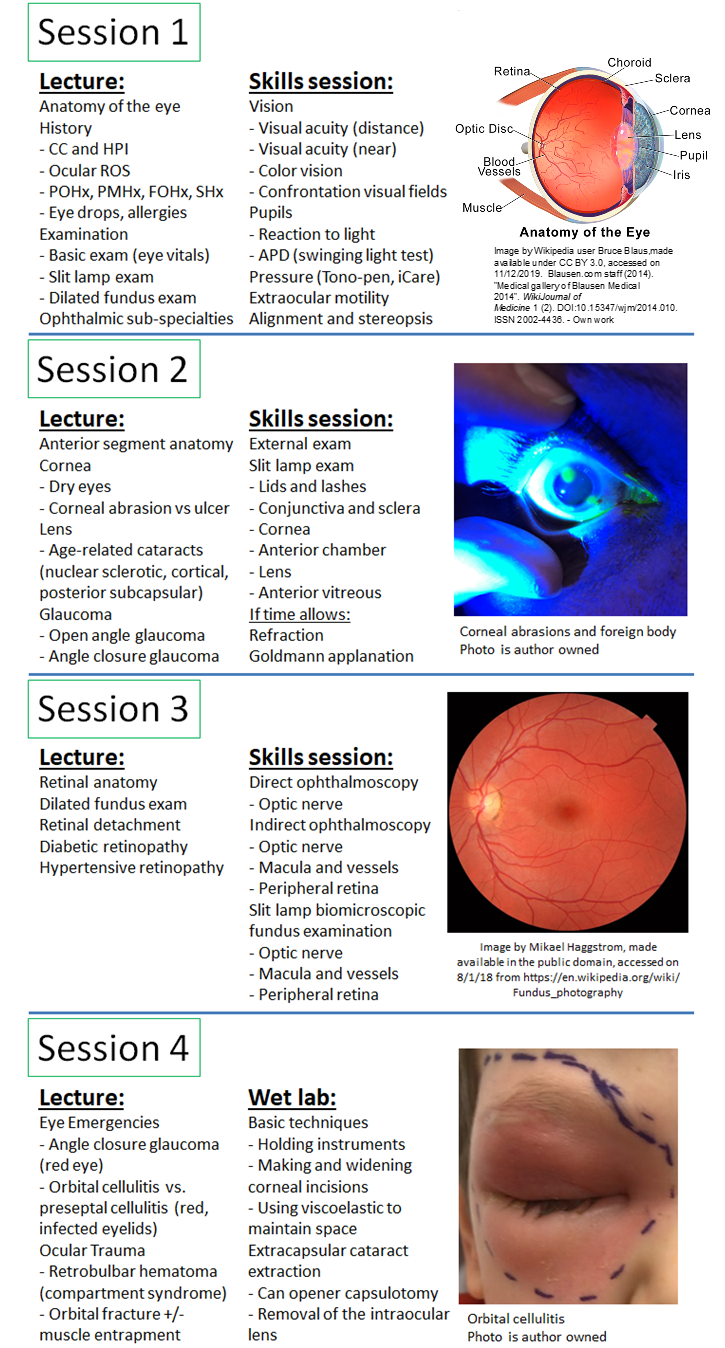
**

**Weekly Course Objectives**

A Guideline for Instructors

**Session 1 Objectives – Introduction to Ophthalmic History and Physical Examination**

First 45 Minutes

Presentation – Conference Room

- Brief course introduction by faculty preceptor
- Hand out pre-course survey and pre-course test
  - Give students approximately 20 minutes to complete them
  - Explain that answers will be provided *after* students take the same test as a *post-course* test during session 4
- Introductory PowerPoint presentation: ophthalmologic history and physical including the ophthalmologic “vital signs,” documenting exam findings, overview of subspecialties and pathologies

*Required Materials for Small Group Practice:*

- *Proparacaine drops*
- *Occluders*
- *Distance Snellen charts and/or near cards*
- *Pupil lights (pen lights or muscle lights)*
- *Tonopen and/or i-Care devices*

*Optional:*

- *Ishihara color vision book*
- *Stereo fly book*
- *Phoropters*

Remaining 1 Hour and 15 Minutes

Small Group Practice – Exam Rooms

- Taking a history
- Visual acuity (distance and near) including pinhole
- Pupils including assessing for anisocoria and APD
- Pressures (Tonopen and/or iCare)
- Extraocular movements
- Confrontation visual fields
- Color vision
- Stereovision
- Time-permitting, refraction with phoropters

**Session 2 Objectives – Anterior Eye Pathologies**

First 45 Minutes

Presentation – Conference Room

- 5-minute recap of last session: ophthalmologic history and physical, ophthalmologic “vital signs,” documenting exam findings
- Anterior segment PowerPoint presentation: anterior segment anatomy, corneal pathology, cataract, glaucoma

*Required Materials for Small Group Practice:*

- *Proparacaine drops*
- *Tonopen and i-Care*
- *Exam rooms containing slit lamps*

Remaining 1 Hour and 15 Minutes

Small Group Practice – Exam Rooms

- Review “vital signs” of eye (vision, pupils, pressure)
- Anterior segment anatomy
- Slit lamp exam (external, lids/lashes, conjunctiva/sclera, cornea, anterior chamber, iris, lens, vitreous)

**Session 3 Objectives – Posterior Eye Pathologies**

First 45 Minutes

Presentation – Conference Room

- 5-minute recap of last session: anterior segment anatomy, corneal pathology, cataract, glaucoma
- Posterior segment PowerPoint presentation: posterior segment anatomy, dilated fundus examination, retinal detachment, diabetic retinopathy

*Required Materials for Small Group Practice:*

- *Proparacaine drops and dilating drops*
- *90 D and 20 D lenses*
- *Exam rooms containing slit lamps*
- *Direct and indirect ophthalmoscopes*

*Optional:*

- *Retinal camera*

Remaining 1 Hour and 15 Minutes

Small Group Practice – Exam Rooms

- Dilate one eye
- Review slit lamp examination of the anterior segment
- Direct ophthalmoscopy
- Slit lamp biomicroscopy with 90 D lens
- Indirect ophthalmoscopy with 20 D lens

**Session 4 Objectives – Ophthalmic Emergencies and Surgical Wet Lab**

First 45 Minutes

Presentation – Conference Room

- 5-minute recap of last session: posterior segment anatomy, dilated fundus examination, retinal detachment, diabetic retinopathy
- Ophthalmic emergencies PowerPoint presentation: ocular trauma, acute red eye, and orbital cellulitis

*Required Materials for Surgical Wet Lab (see also Surgical Lab Handout):*

- Stereomicroscopes
- 27 G needles, hemostats, and syringes to make bent-needle cystotomes
- Sideport/paracentesis blades
- Pig eyes, heavy metal pins and styrofoam bases to mount them
- Ophthalmic viscoelastic material
- Flexible plastic rulers

Remaining 1 Hour and 15 Minutes

Extracapsular Cataract Surgery Simulation – Wet Lab

- Learn to hold instruments, use stereomicroscope
- Create a temporal paracentesis
- Perform a can-opener capsulotomy
- Hydrodissect and prolapse lens
- Widen an incision (increase temporal paracentesis to 6-7 mm)
- Remove the lens through corneal incision
