# Supplementary material for: The Ophthalmology Mini-Elective Gives Vision to Preclinical Medical Students
Source: MedEdPORTAL. 2020 Nov 23;16:11024. doi: 10.15766/mep_2374-8265.11024 (PMC7703479; doi:10.15766/mep_2374-8265.11024)
Supplement: Supplementary file 1 — Course Syllabus.docxInstructor Introduction.docxWeekly Course Time Line & Objectives.docxSession 1 - Intro to Ophthalmology.pptxSession 2 - Anterior Segment.pptxSession 3 - Posterior Segment.pptxSession 4 - Eye Emergencies and Trauma.pptxLaboratory Session Guide.pdfPrecourse Survey.docxPre- and Posttest.docxPostcourse Survey.docxPre- and Posttest Answers.docx [file mep_2374-8265.11024-s001.zip › D. Session 1 - Intro to Ophthalmology.pptx]

## Slide 1
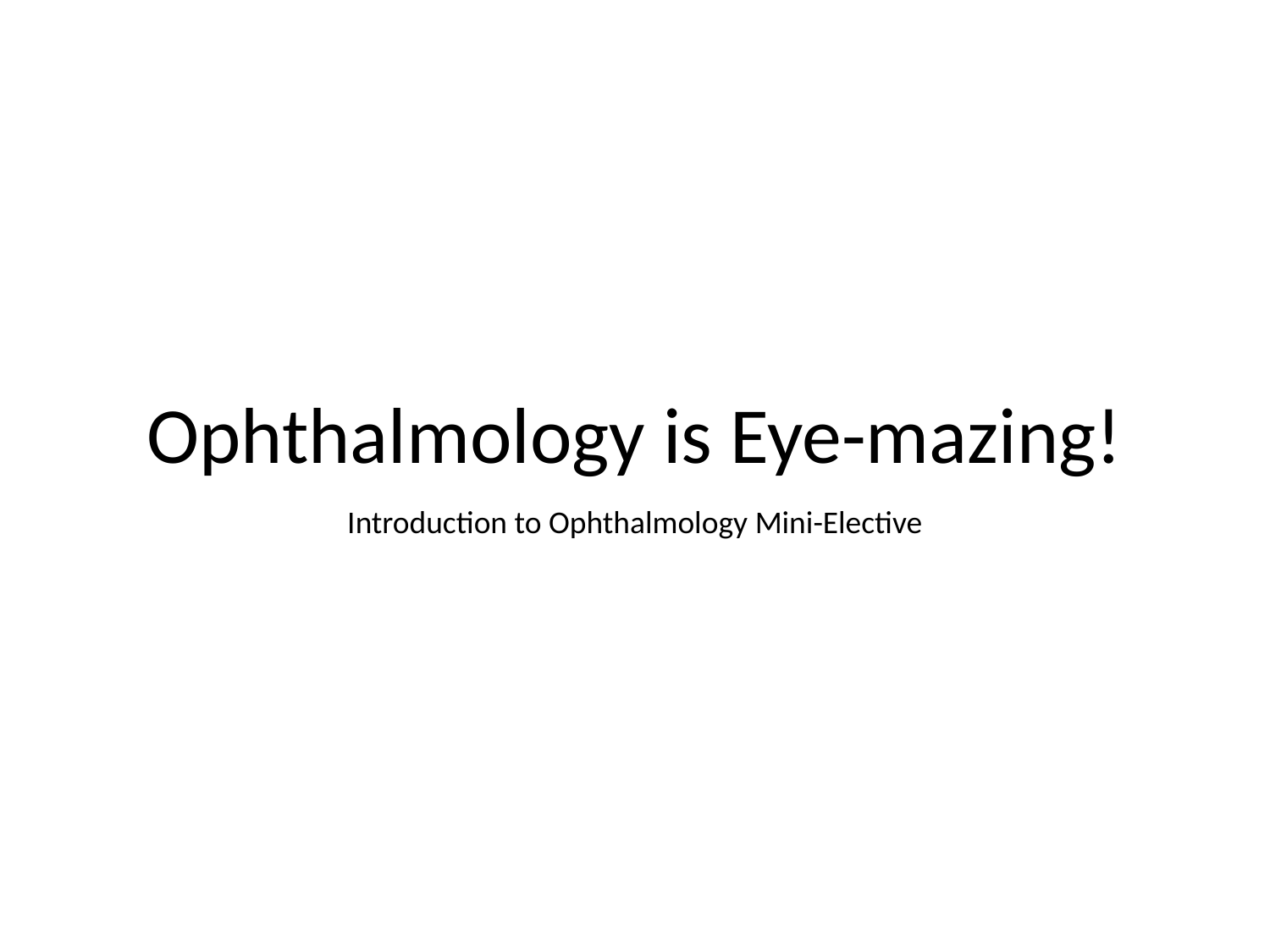

# Ophthalmology is Eye-mazing!
Introduction to Ophthalmology Mini-Elective

## Slide 2
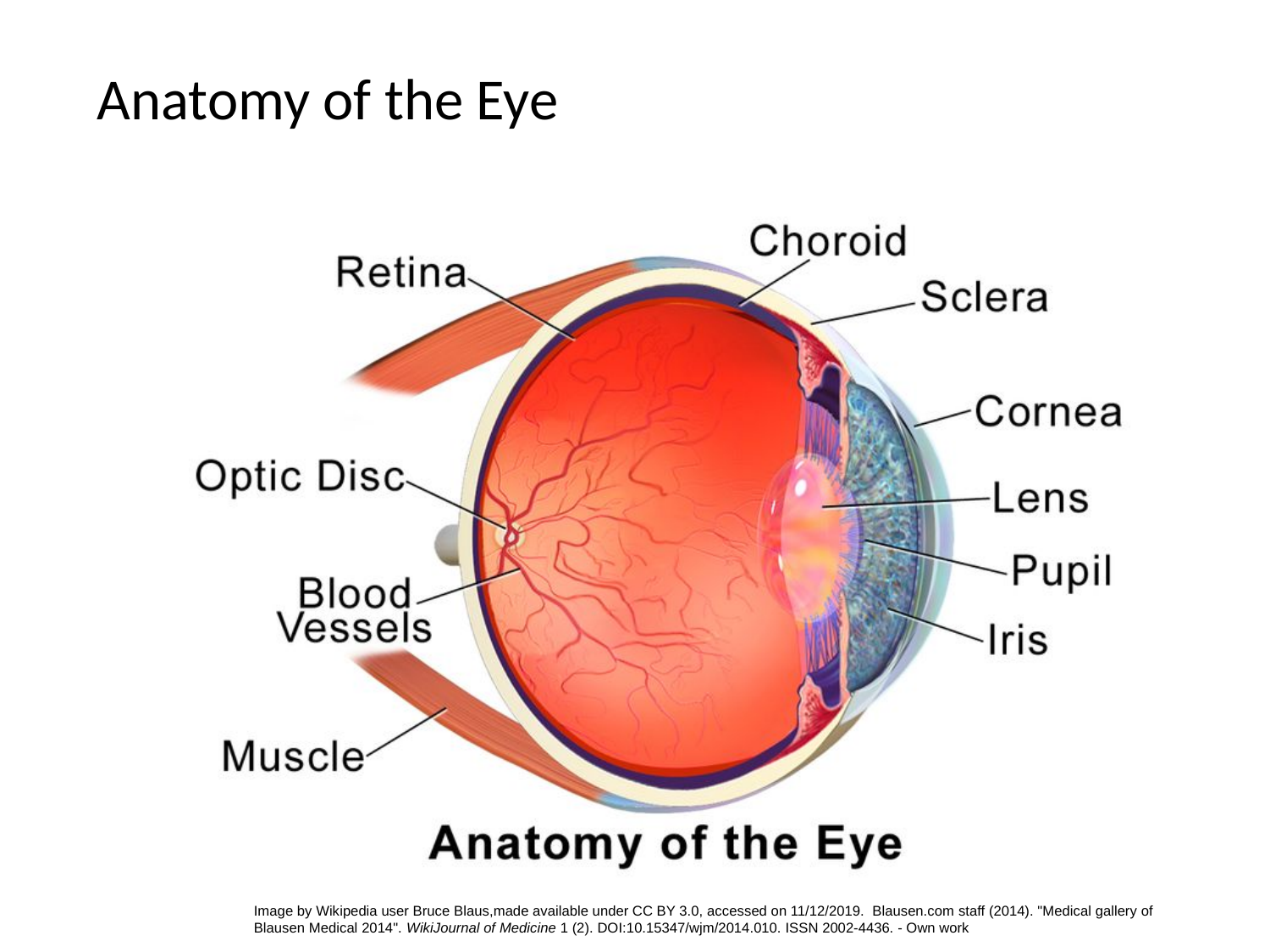

# Anatomy of the Eye
Image by Wikipedia user Bruce Blaus,made available under CC BY 3.0, accessed on 11/12/2019. Blausen.com staff (2014). "Medical gallery of Blausen Medical 2014". WikiJournal of Medicine 1 (2). DOI:10.15347/wjm/2014.010. ISSN 2002-4436. - Own work

## Slide 3
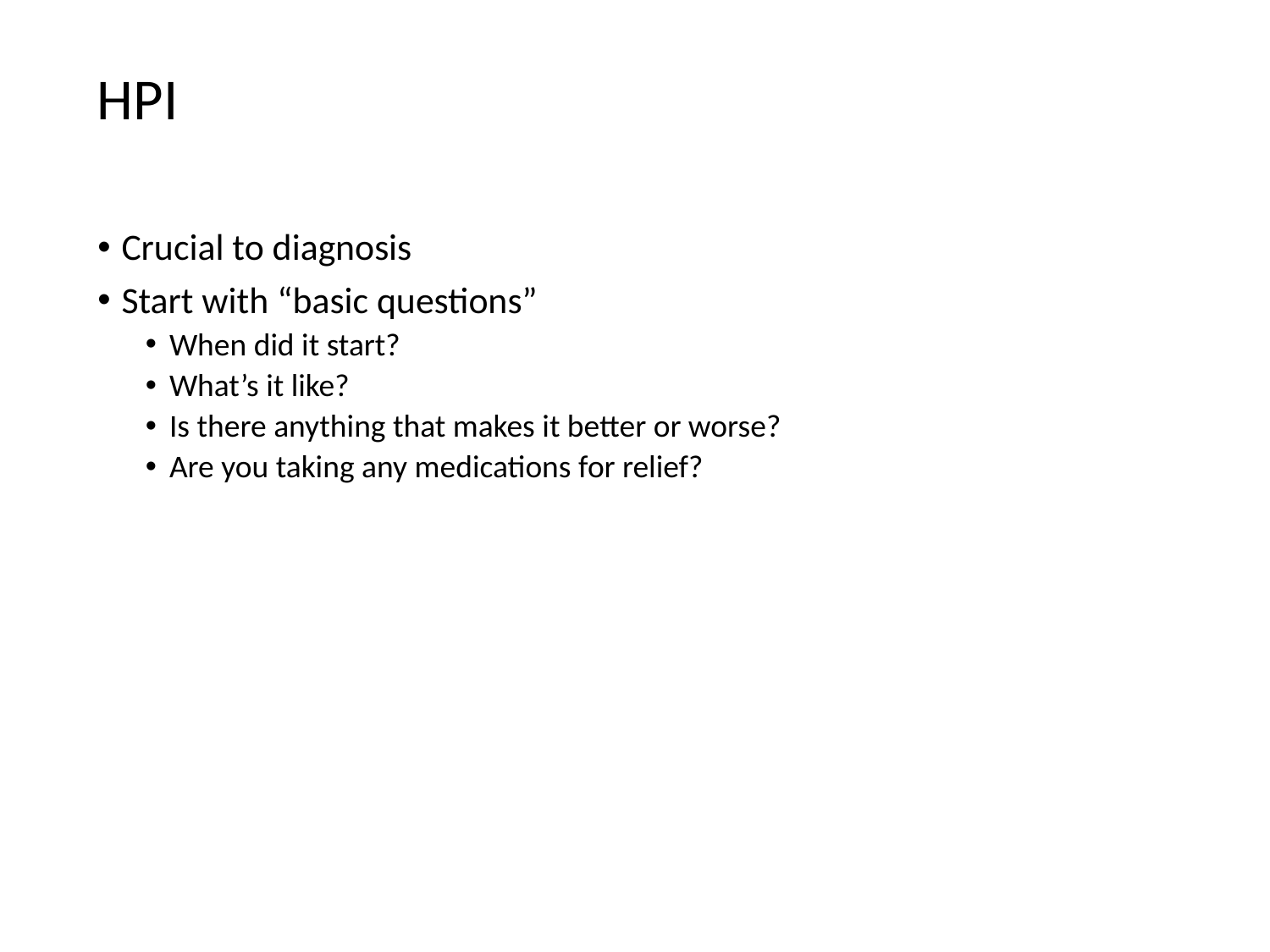

# HPI
Crucial to diagnosis
Start with “basic questions”
When did it start?
What’s it like?
Is there anything that makes it better or worse?
Are you taking any medications for relief?

## Slide 4
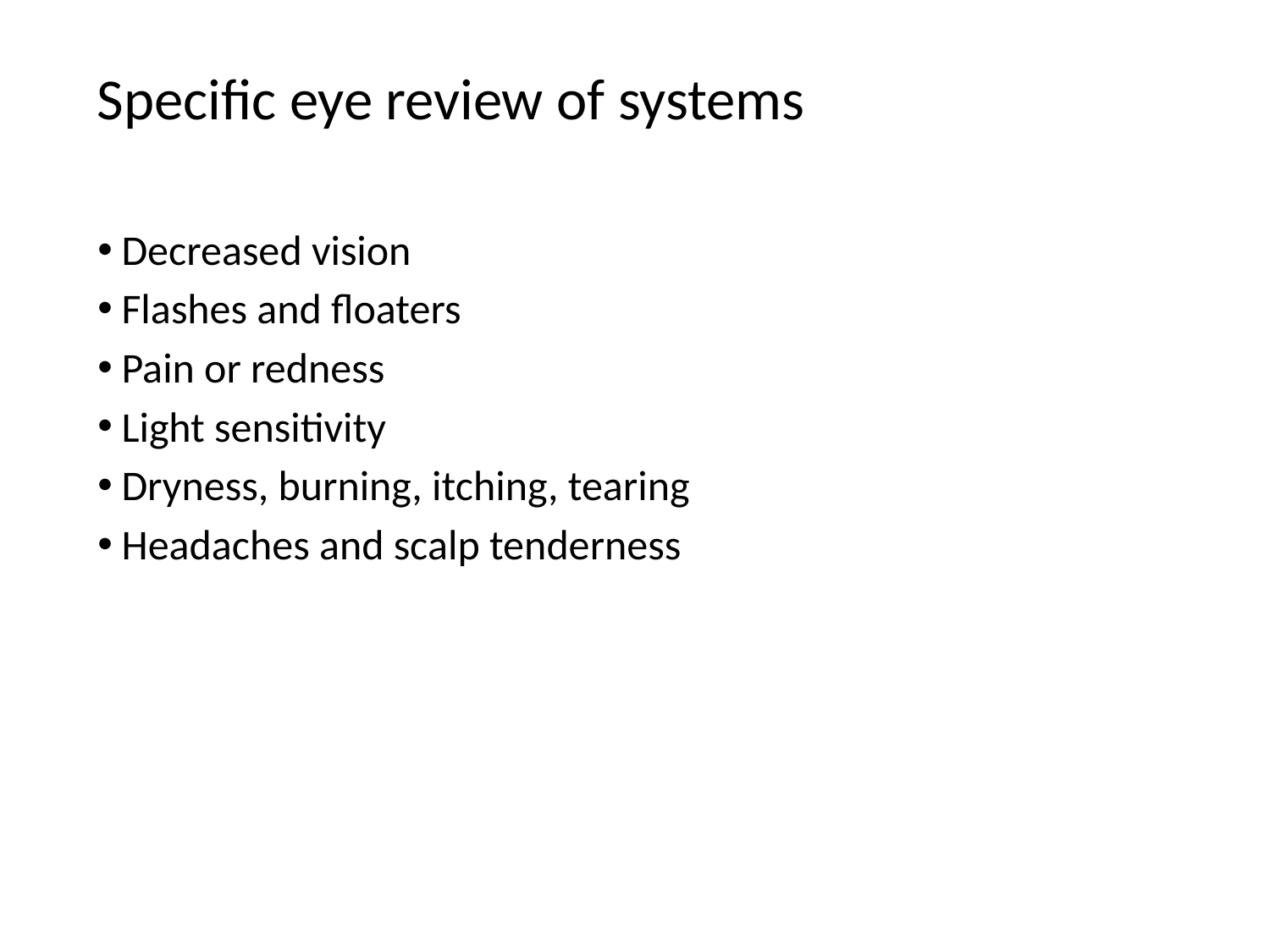

# Specific eye review of systems
Decreased vision
Flashes and floaters
Pain or redness
Light sensitivity
Dryness, burning, itching, tearing
Headaches and scalp tenderness

## Slide 5
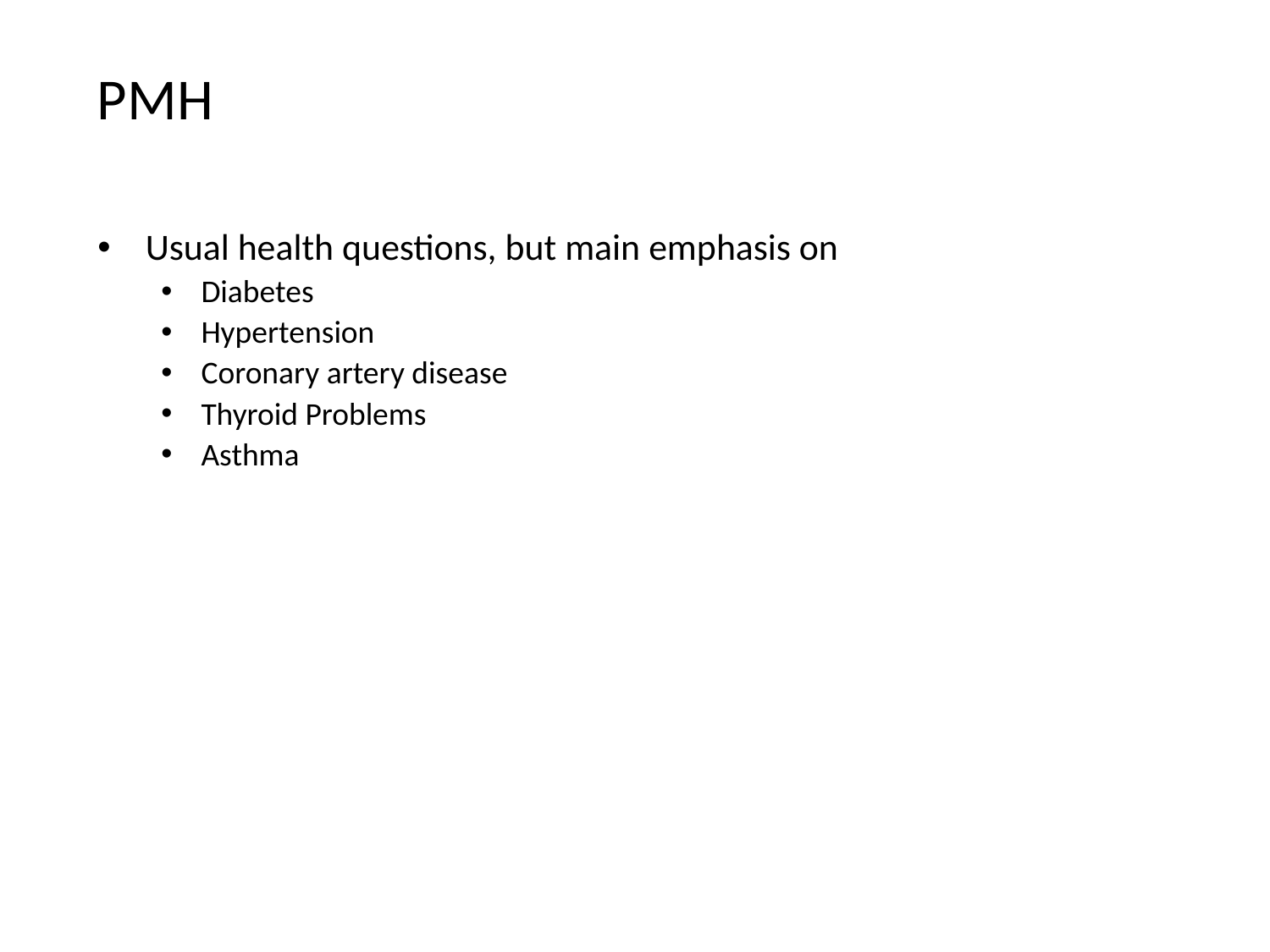

# PMH
Usual health questions, but main emphasis on
Diabetes
Hypertension
Coronary artery disease
Thyroid Problems
Asthma

## Slide 6
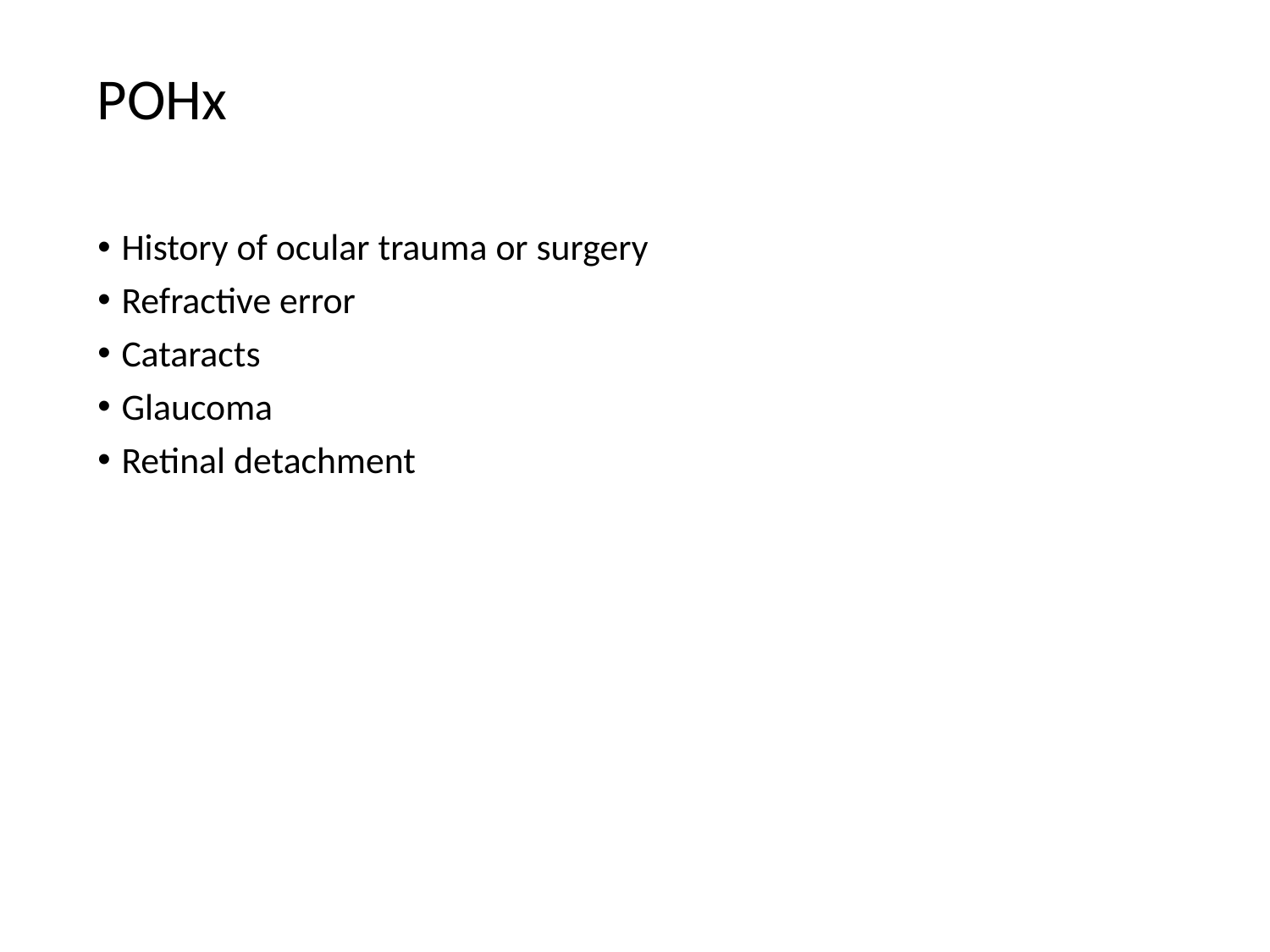

# POHx
History of ocular trauma or surgery
Refractive error
Cataracts
Glaucoma
Retinal detachment

## Slide 7
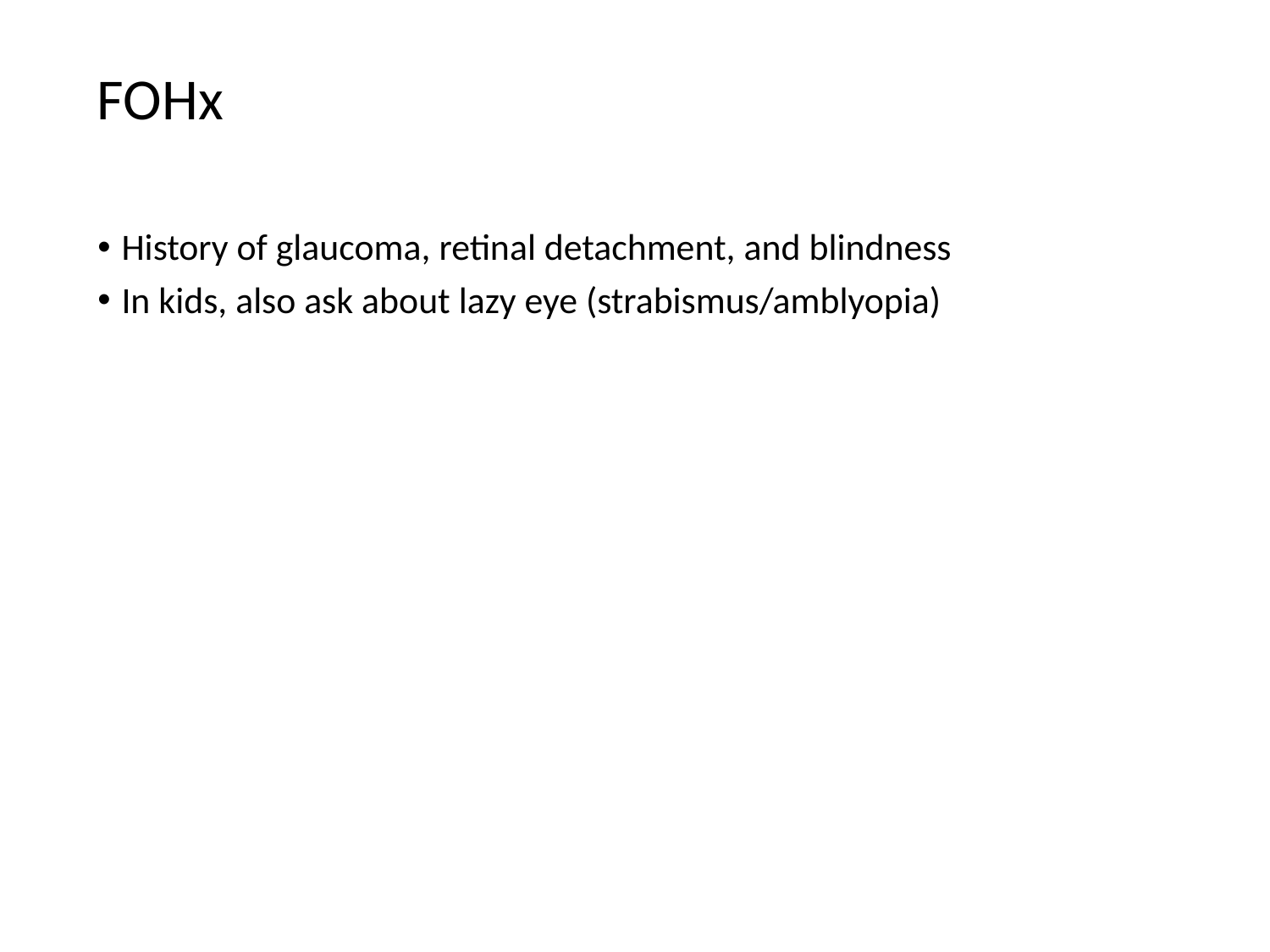

# FOHx
History of glaucoma, retinal detachment, and blindness
In kids, also ask about lazy eye (strabismus/amblyopia)

## Slide 8
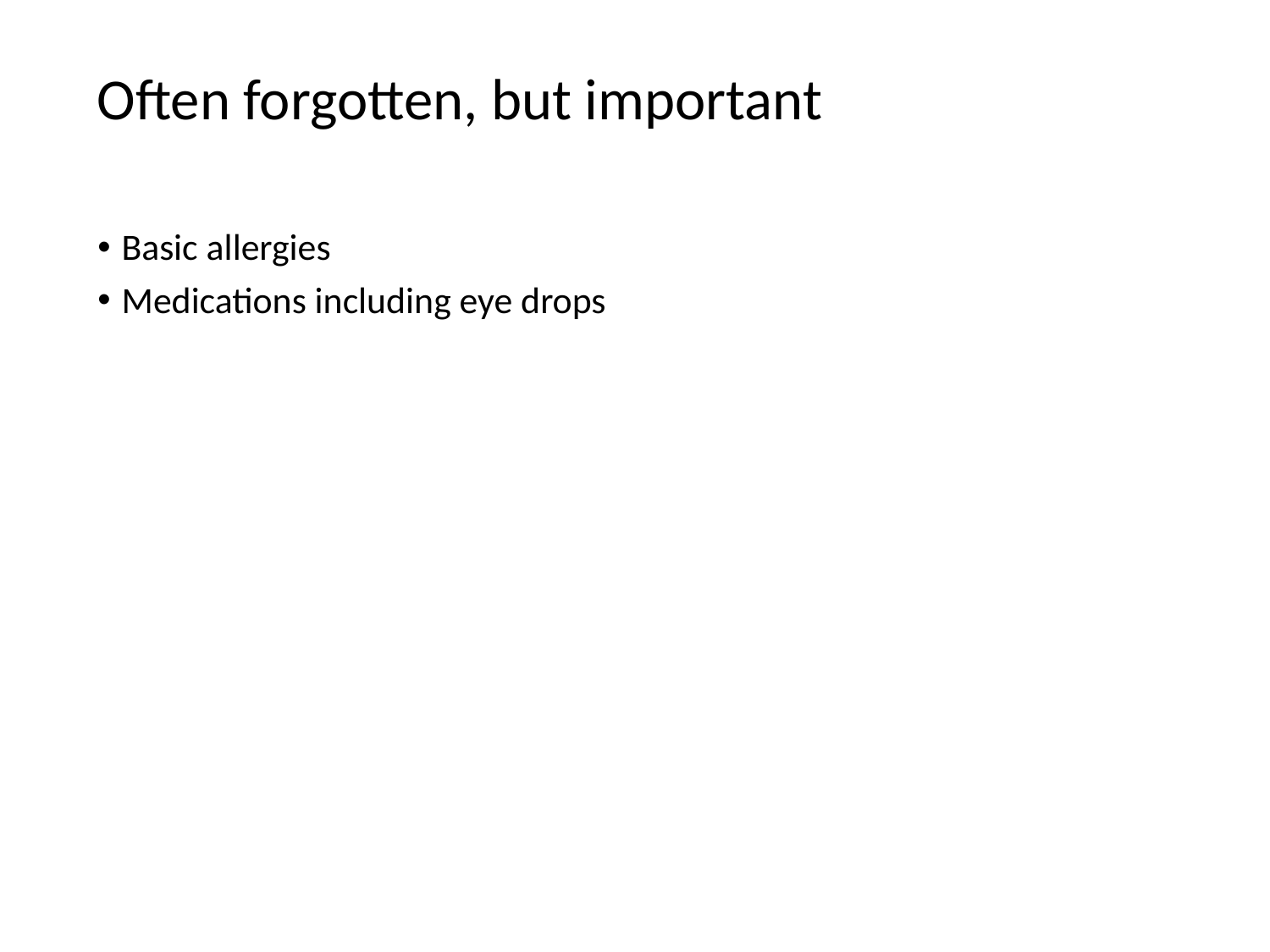

# Often forgotten, but important
Basic allergies
Medications including eye drops

## Slide 9
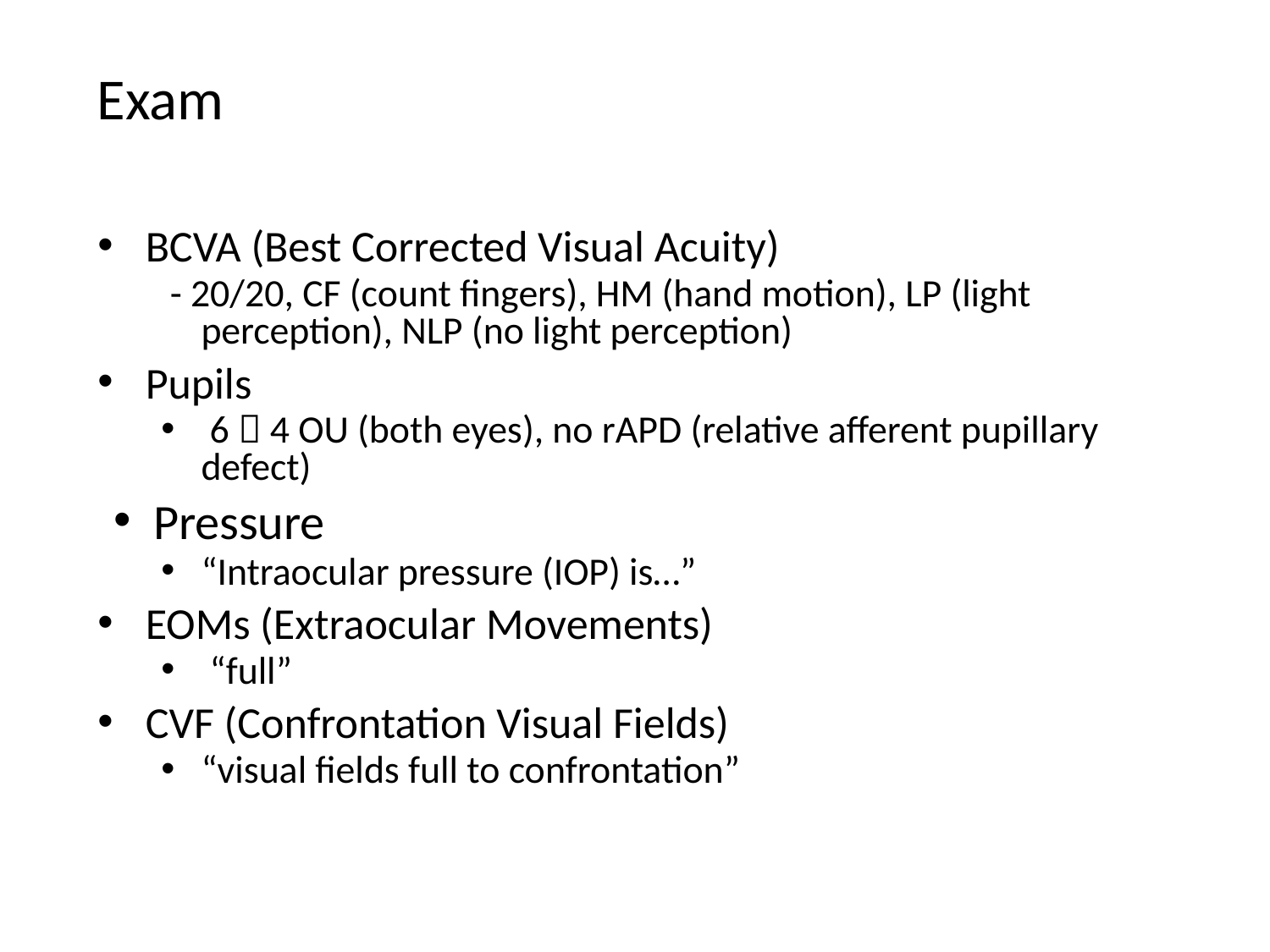

# Exam
BCVA (Best Corrected Visual Acuity)
 - 20/20, CF (count fingers), HM (hand motion), LP (light perception), NLP (no light perception)
Pupils
 6  4 OU (both eyes), no rAPD (relative afferent pupillary defect)
Pressure
“Intraocular pressure (IOP) is…”
EOMs (Extraocular Movements)
 “full”
CVF (Confrontation Visual Fields)
“visual fields full to confrontation”

## Slide 10
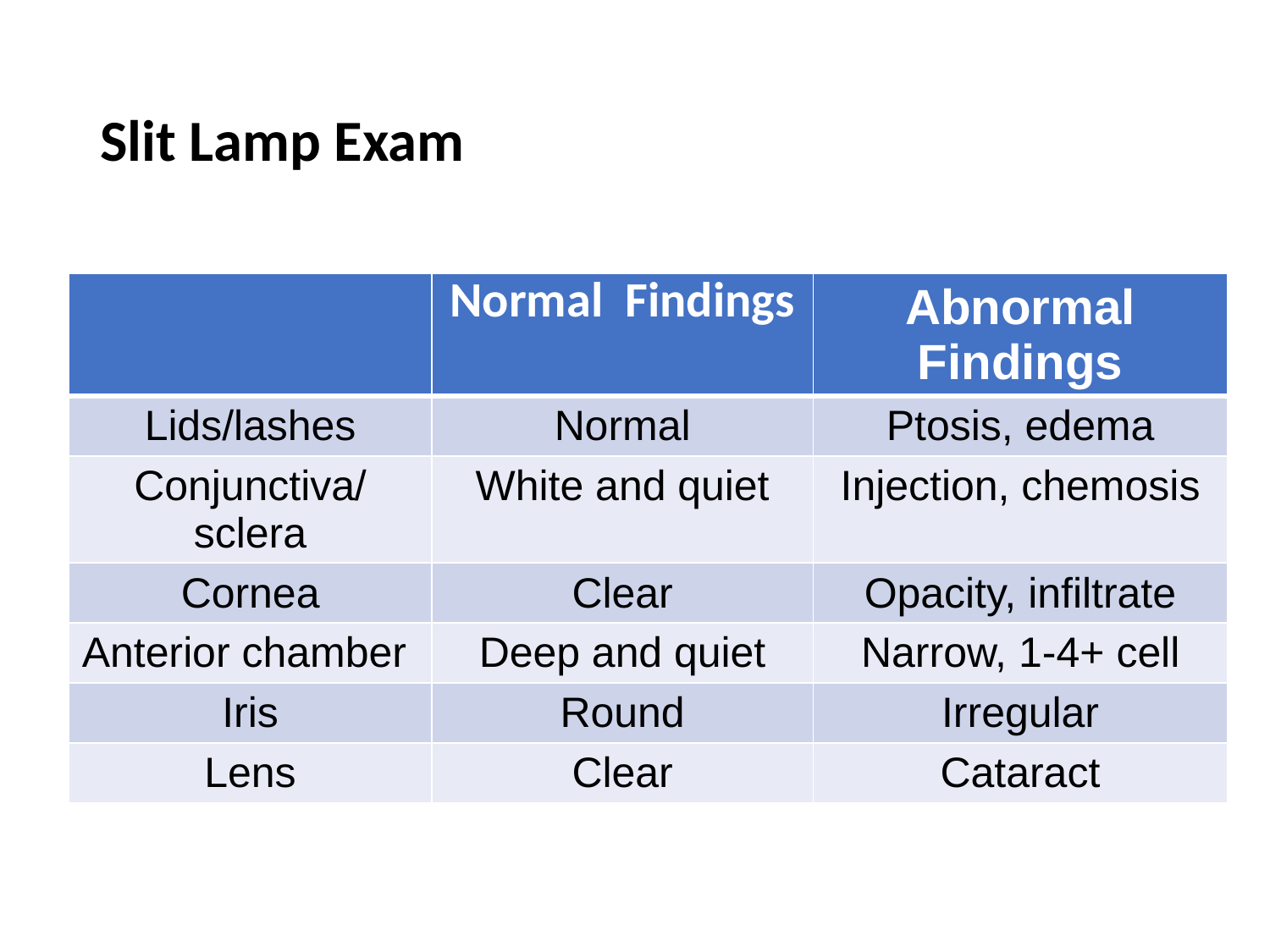

# Slit Lamp Exam
| | Normal Findings | Abnormal Findings |
| --- | --- | --- |
| Lids/lashes | Normal | Ptosis, edema |
| Conjunctiva/sclera | White and quiet | Injection, chemosis |
| Cornea | Clear | Opacity, infiltrate |
| Anterior chamber | Deep and quiet | Narrow, 1-4+ cell |
| Iris | Round | Irregular |
| Lens | Clear | Cataract |

## Slide 11
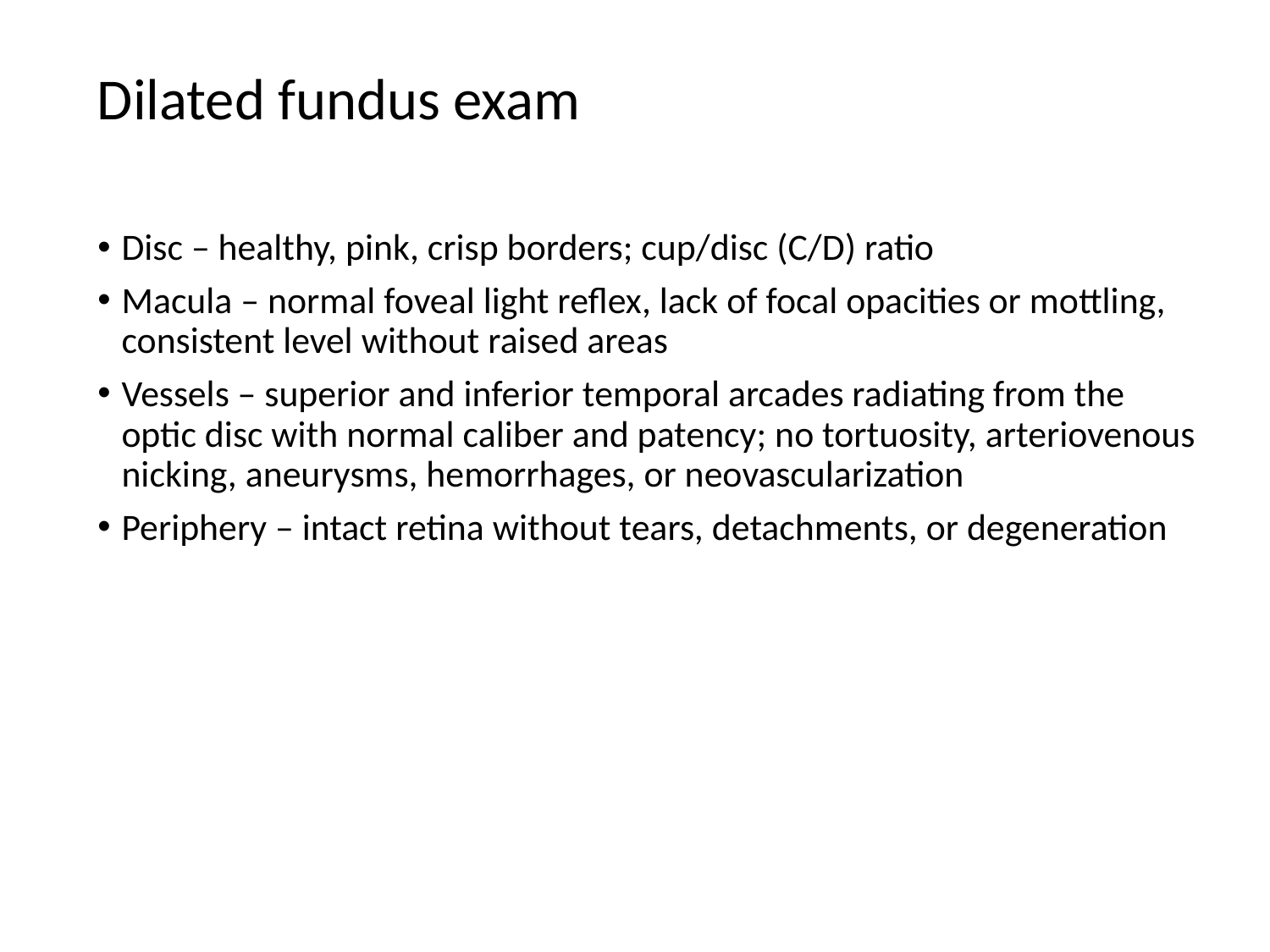

# Dilated fundus exam
Disc – healthy, pink, crisp borders; cup/disc (C/D) ratio
Macula – normal foveal light reflex, lack of focal opacities or mottling, consistent level without raised areas
Vessels – superior and inferior temporal arcades radiating from the optic disc with normal caliber and patency; no tortuosity, arteriovenous nicking, aneurysms, hemorrhages, or neovascularization
Periphery – intact retina without tears, detachments, or degeneration

## Slide 12
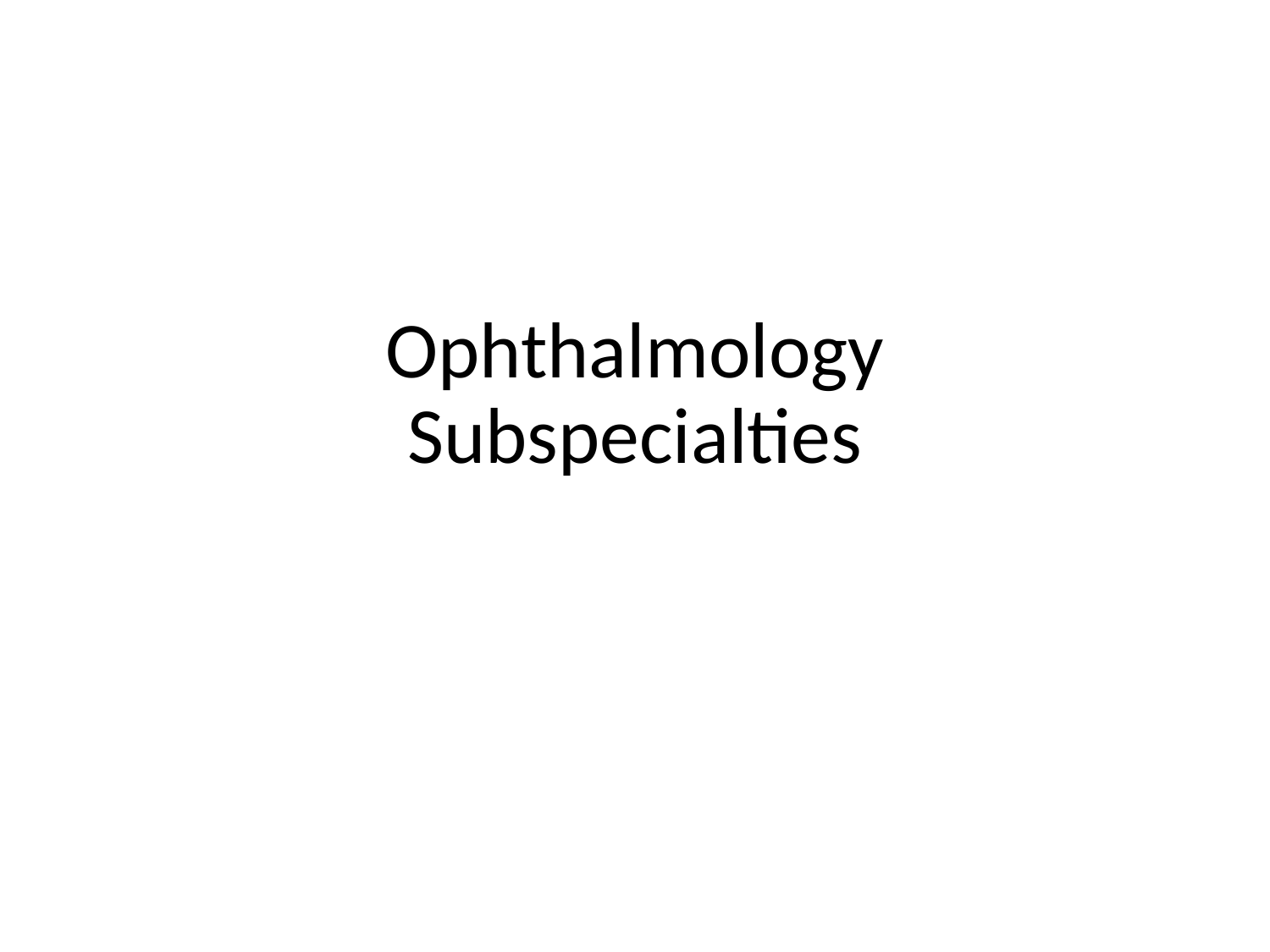

# Ophthalmology Subspecialties

## Slide 13
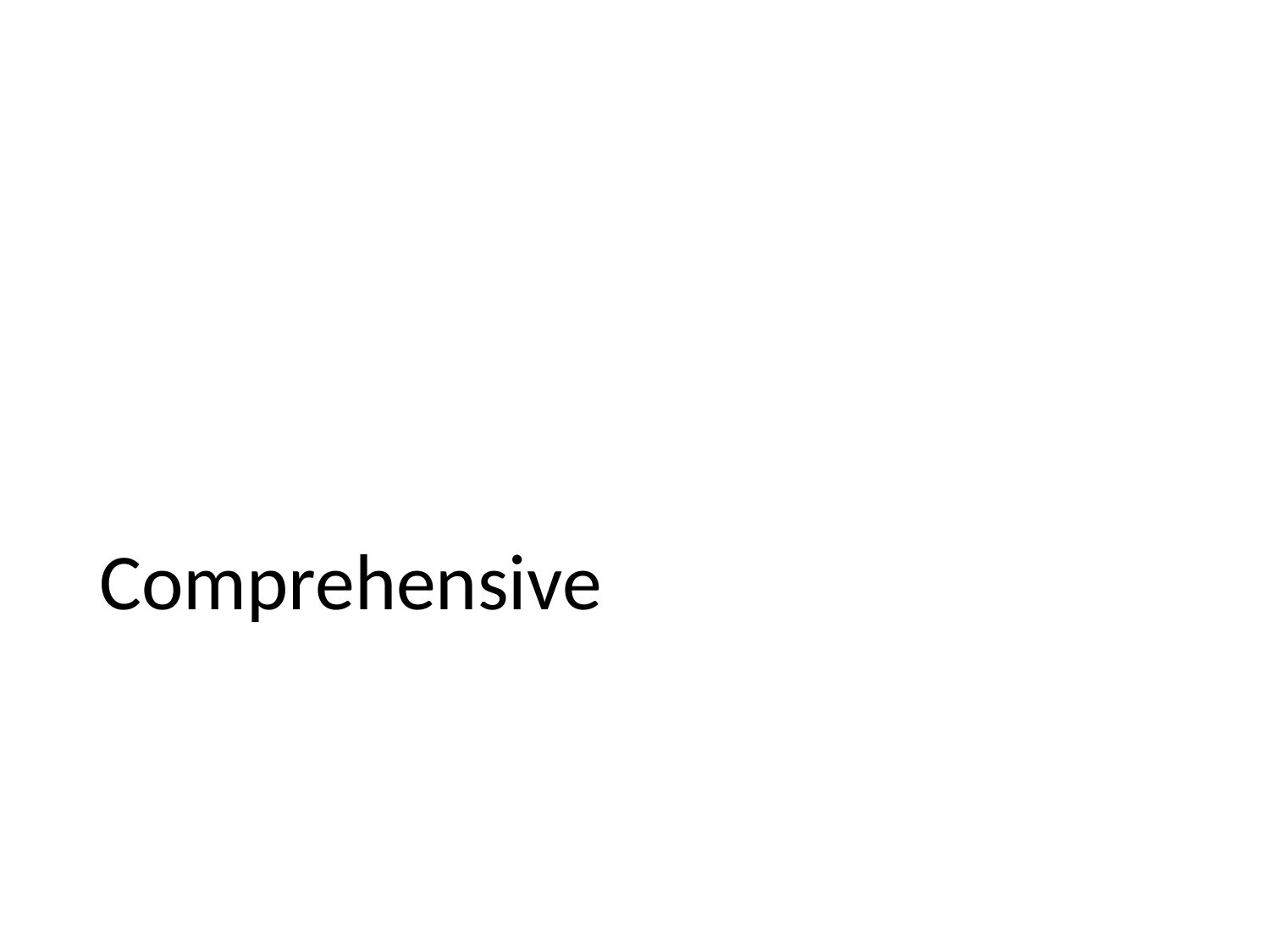

# Comprehensive

## Slide 14
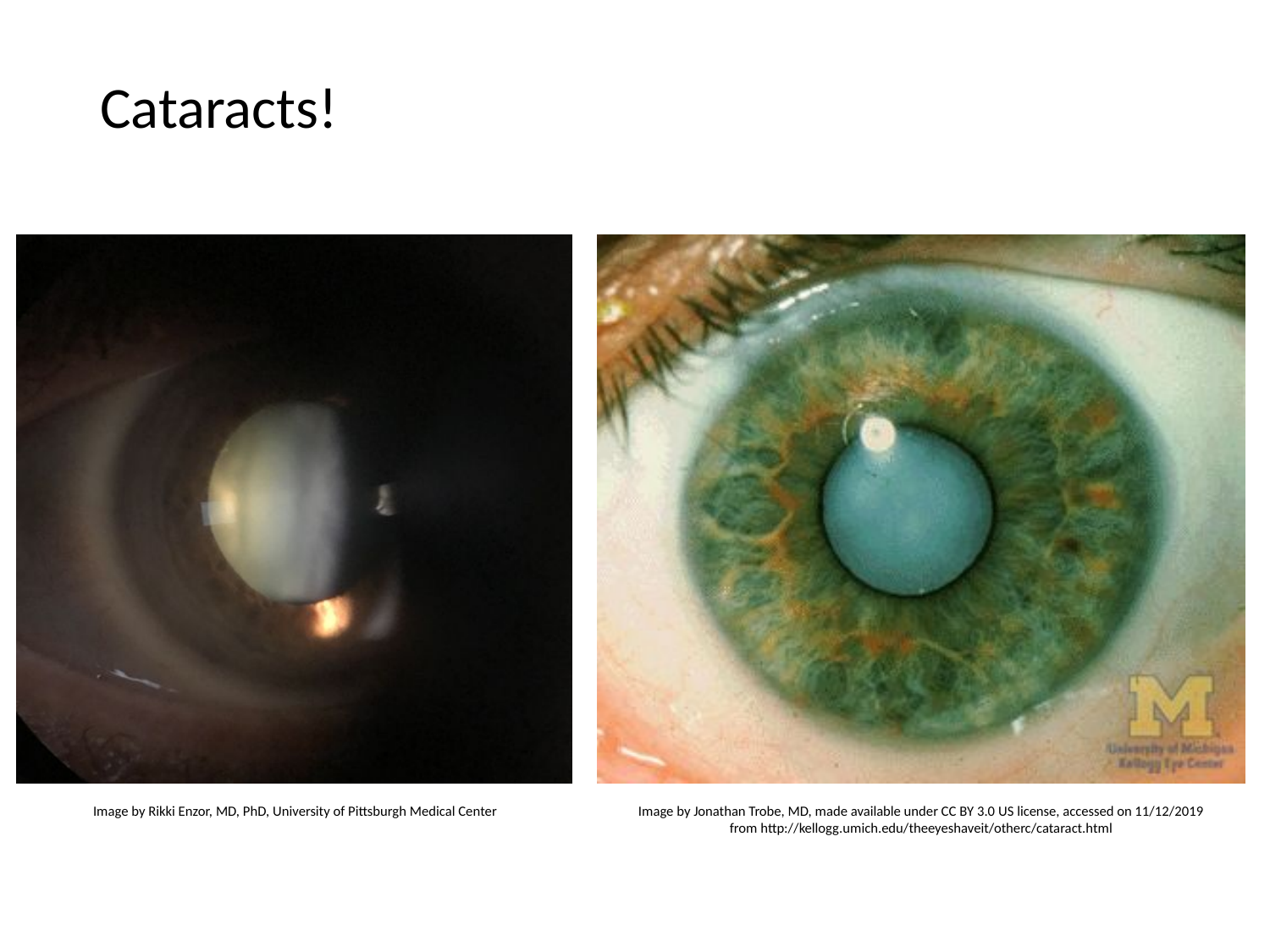

# Cataracts!
Image by Rikki Enzor, MD, PhD, University of Pittsburgh Medical Center
Image by Jonathan Trobe, MD, made available under CC BY 3.0 US license, accessed on 11/12/2019 from http://kellogg.umich.edu/theeyeshaveit/otherc/cataract.html

## Slide 15
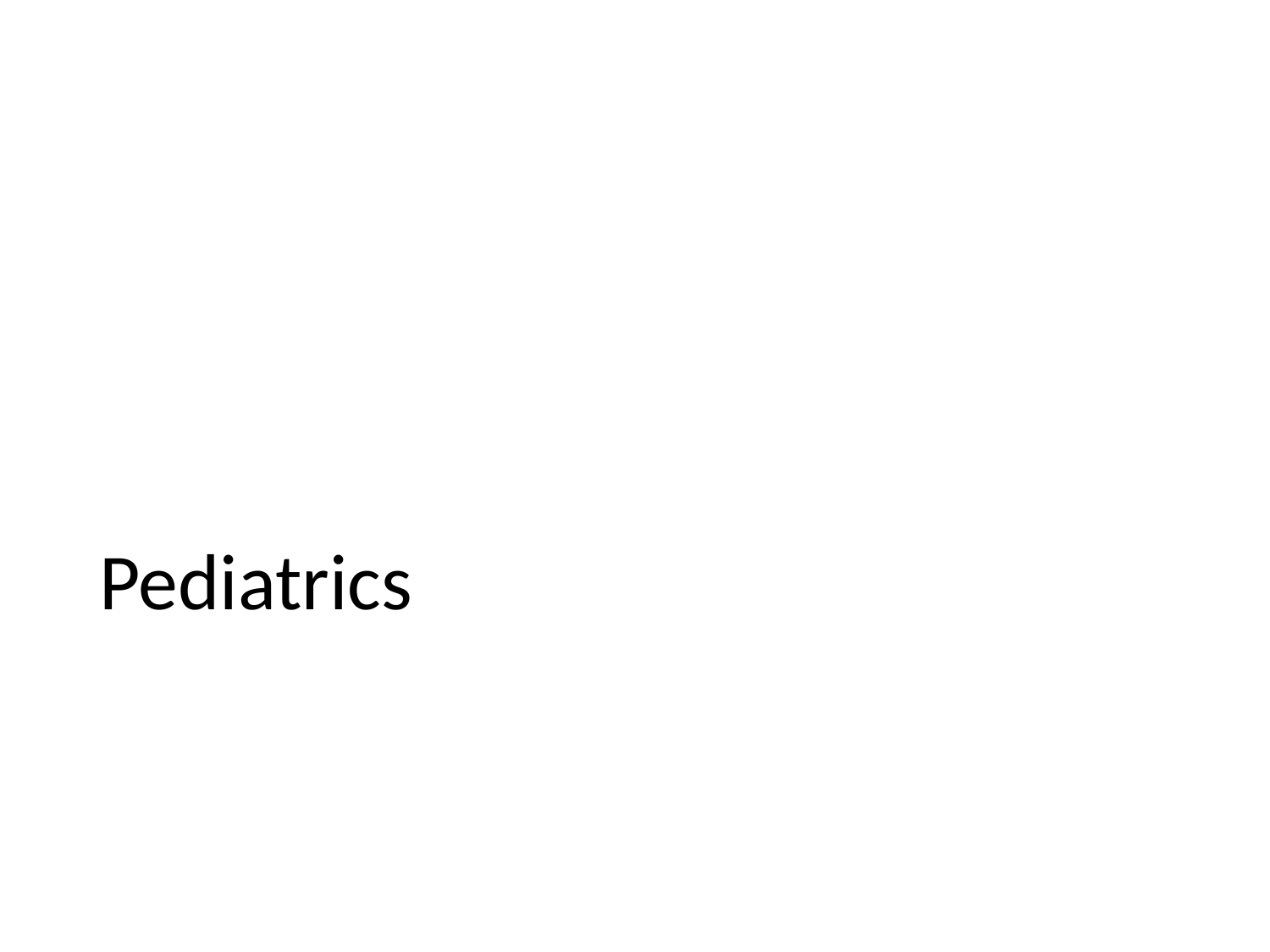

# Pediatrics

## Slide 16
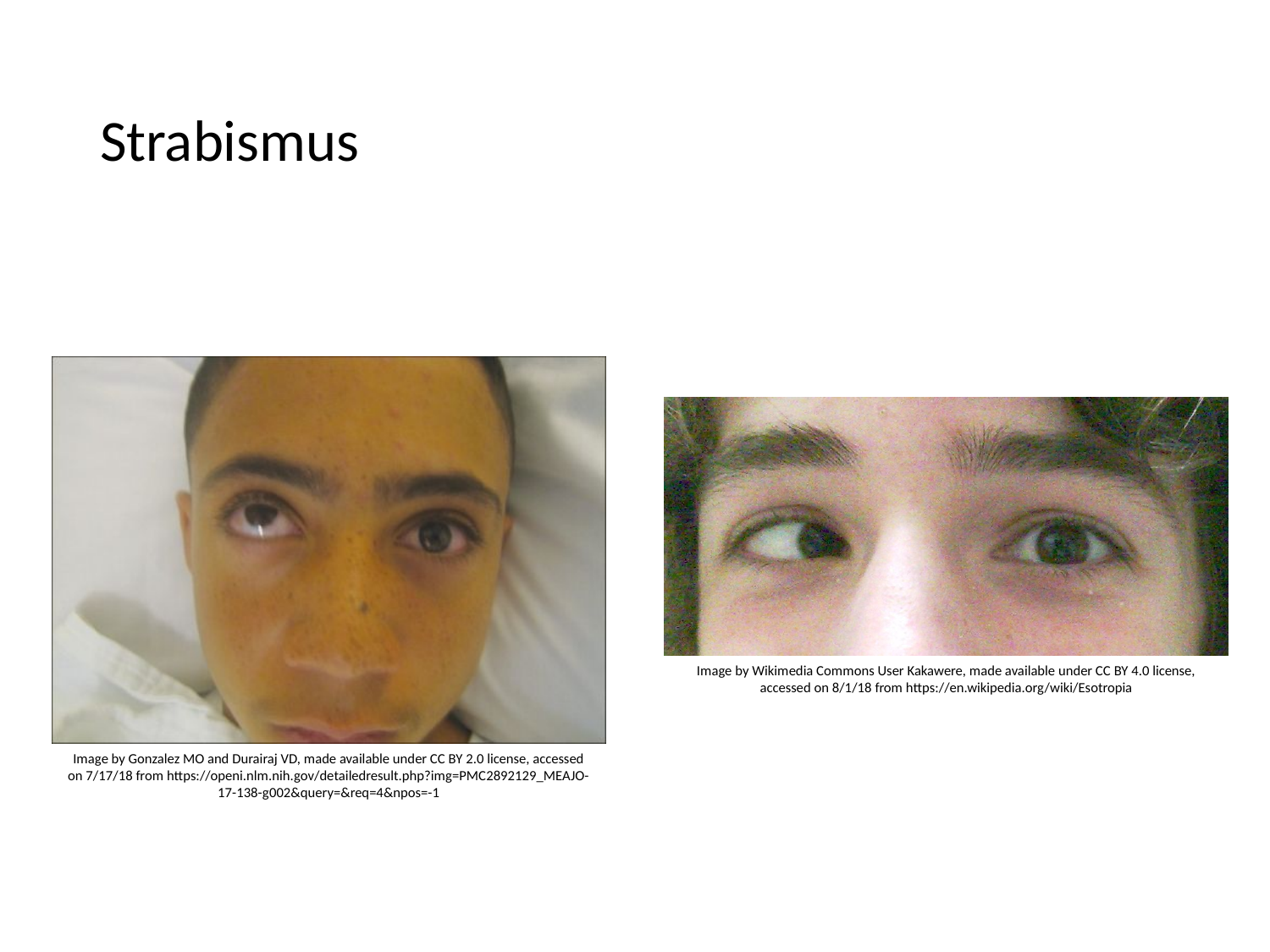

# Strabismus
Image by Gonzalez MO and Durairaj VD, made available under CC BY 2.0 license, accessed on 7/17/18 from https://openi.nlm.nih.gov/detailedresult.php?img=PMC2892129_MEAJO-17-138-g002&query=&req=4&npos=-1
Image by Wikimedia Commons User Kakawere, made available under CC BY 4.0 license, accessed on 8/1/18 from https://en.wikipedia.org/wiki/Esotropia

## Slide 17
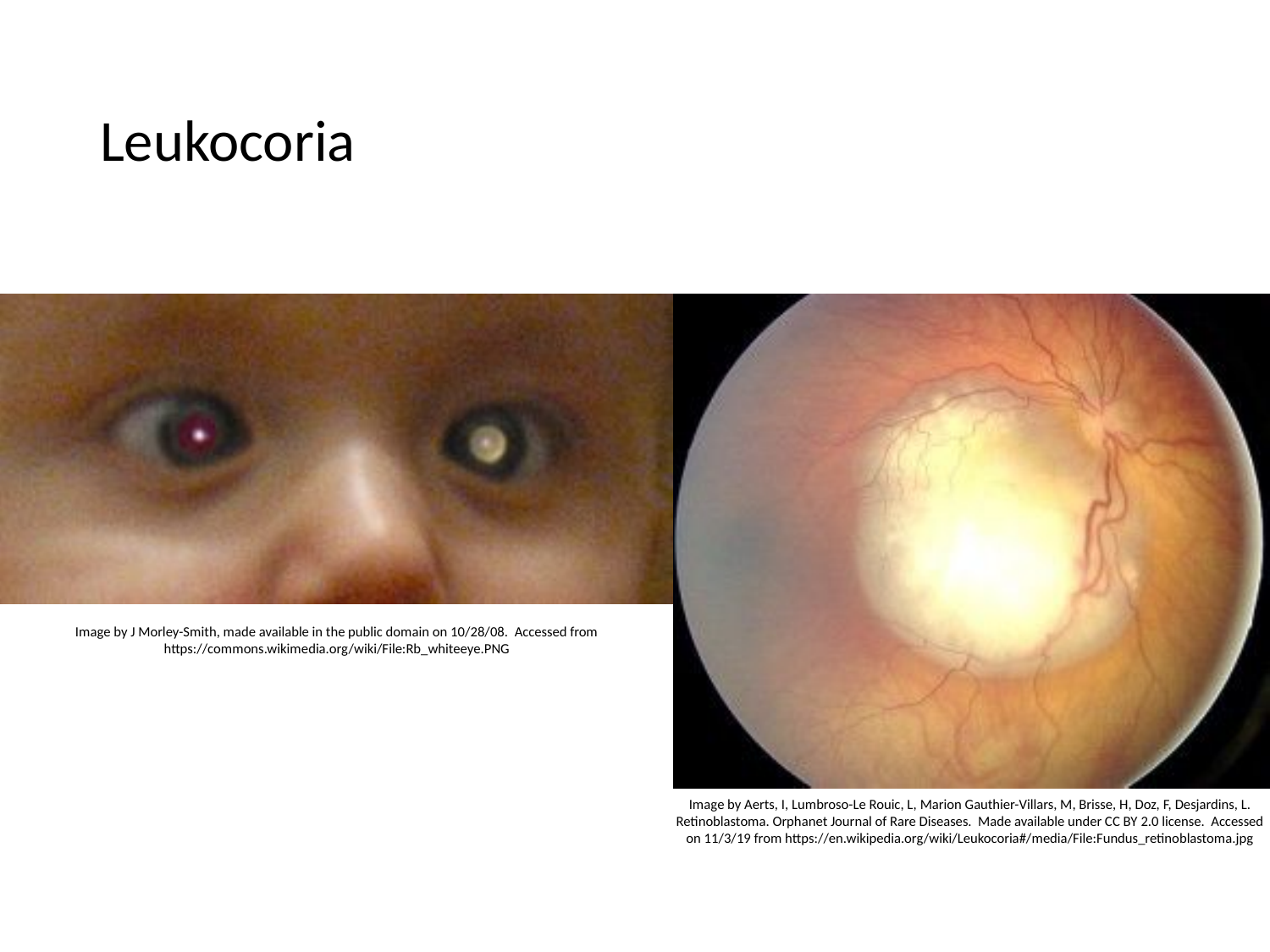

# Leukocoria
Image by J Morley-Smith, made available in the public domain on 10/28/08. Accessed from https://commons.wikimedia.org/wiki/File:Rb_whiteeye.PNG
Image by Aerts, I, Lumbroso-Le Rouic, L, Marion Gauthier-Villars, M, Brisse, H, Doz, F, Desjardins, L. Retinoblastoma. Orphanet Journal of Rare Diseases. Made available under CC BY 2.0 license. Accessed on 11/3/19 from https://en.wikipedia.org/wiki/Leukocoria#/media/File:Fundus_retinoblastoma.jpg

## Slide 18
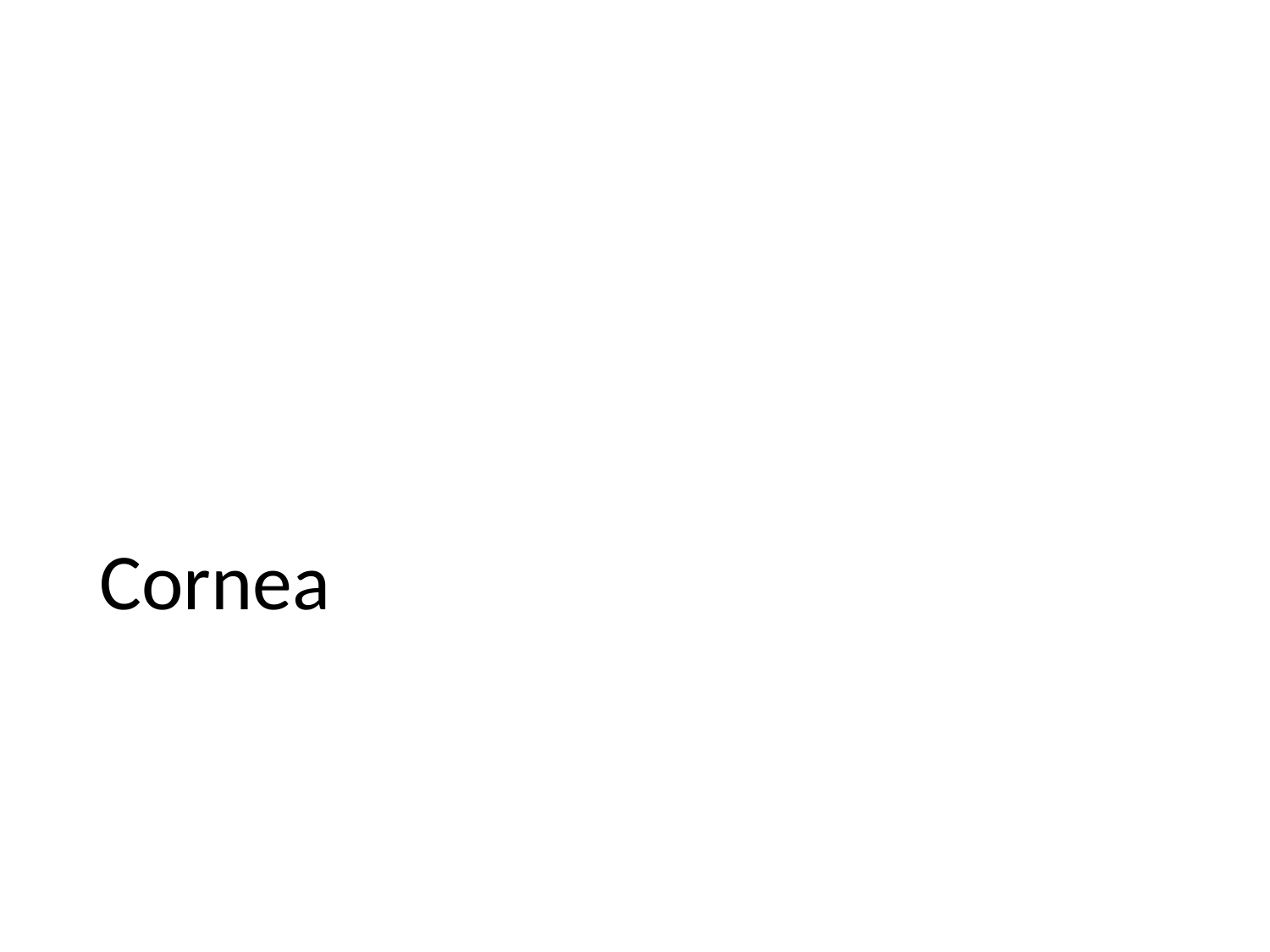

# Cornea

## Slide 19
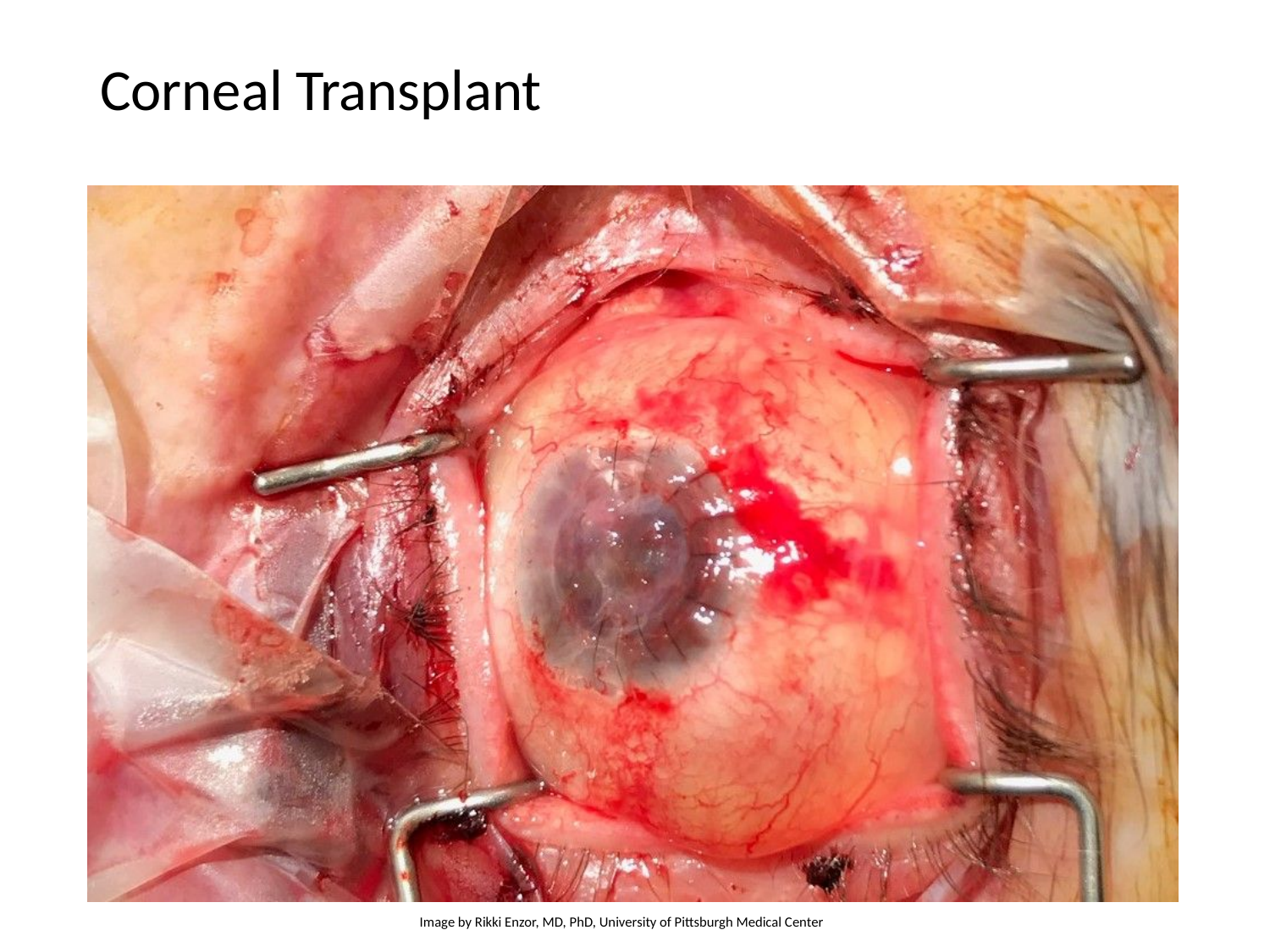

# Corneal Transplant
Image by Rikki Enzor, MD, PhD, University of Pittsburgh Medical Center

## Slide 20
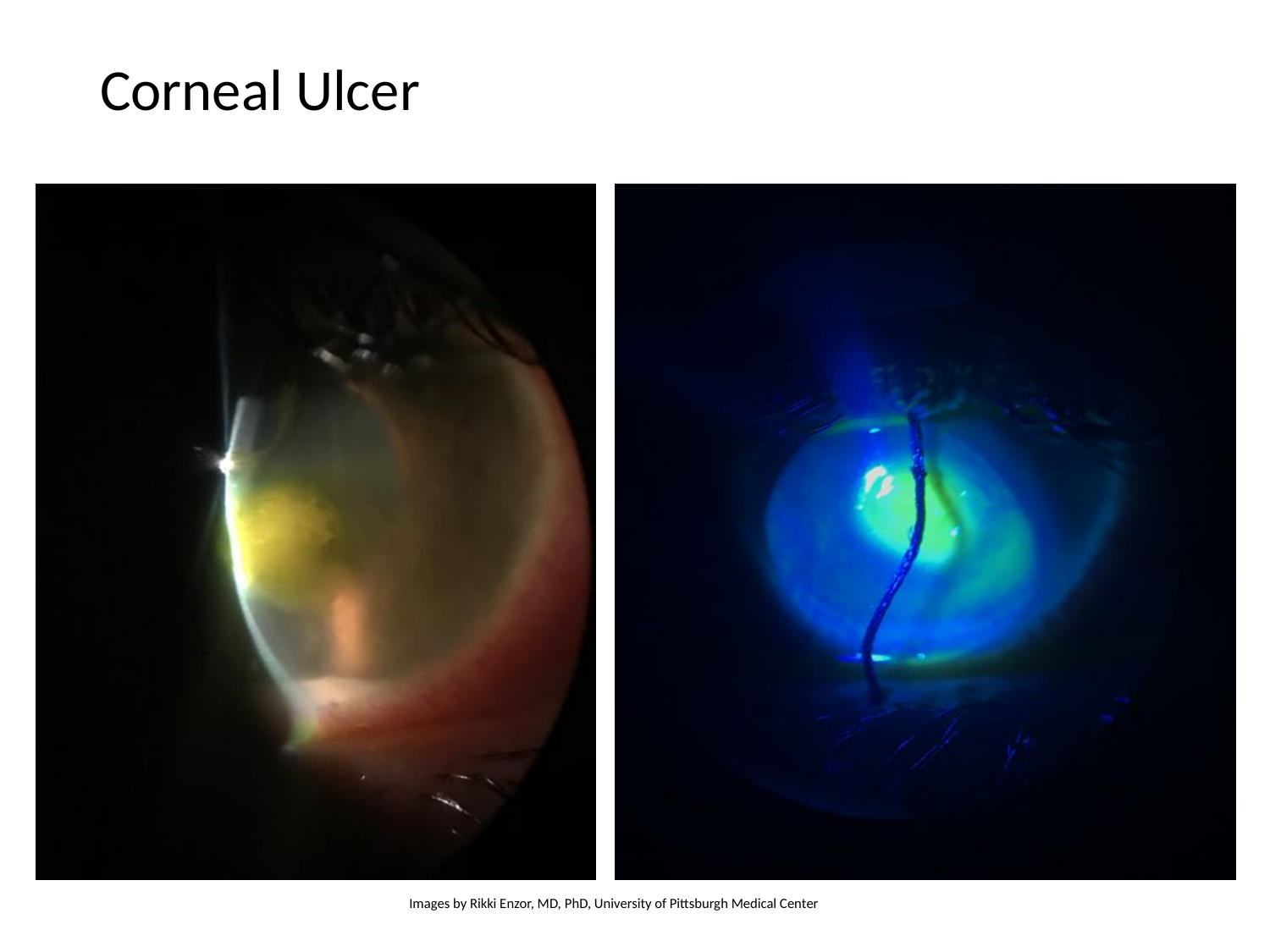

# Corneal Ulcer
Images by Rikki Enzor, MD, PhD, University of Pittsburgh Medical Center

## Slide 21
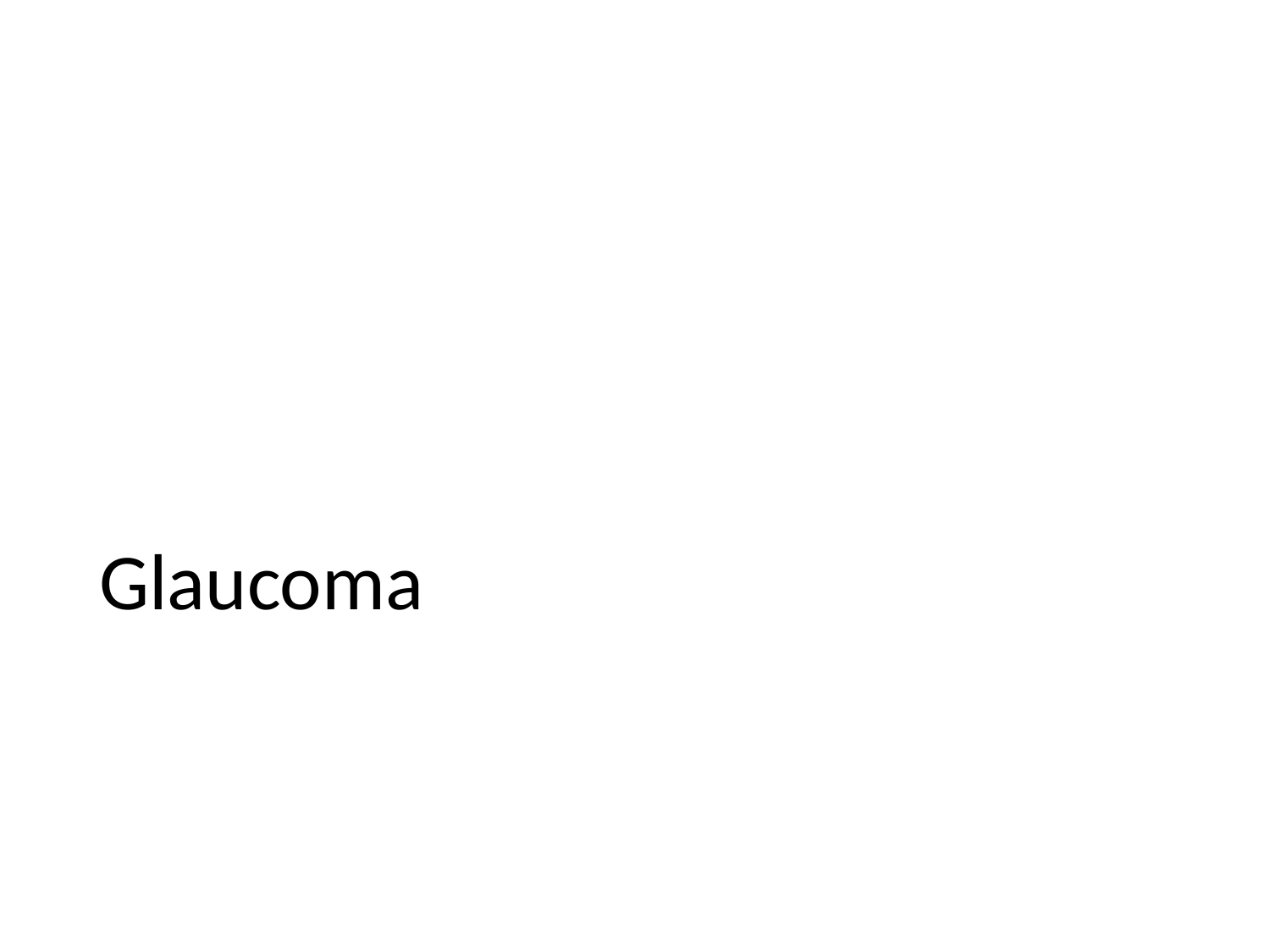

# Glaucoma

## Slide 22
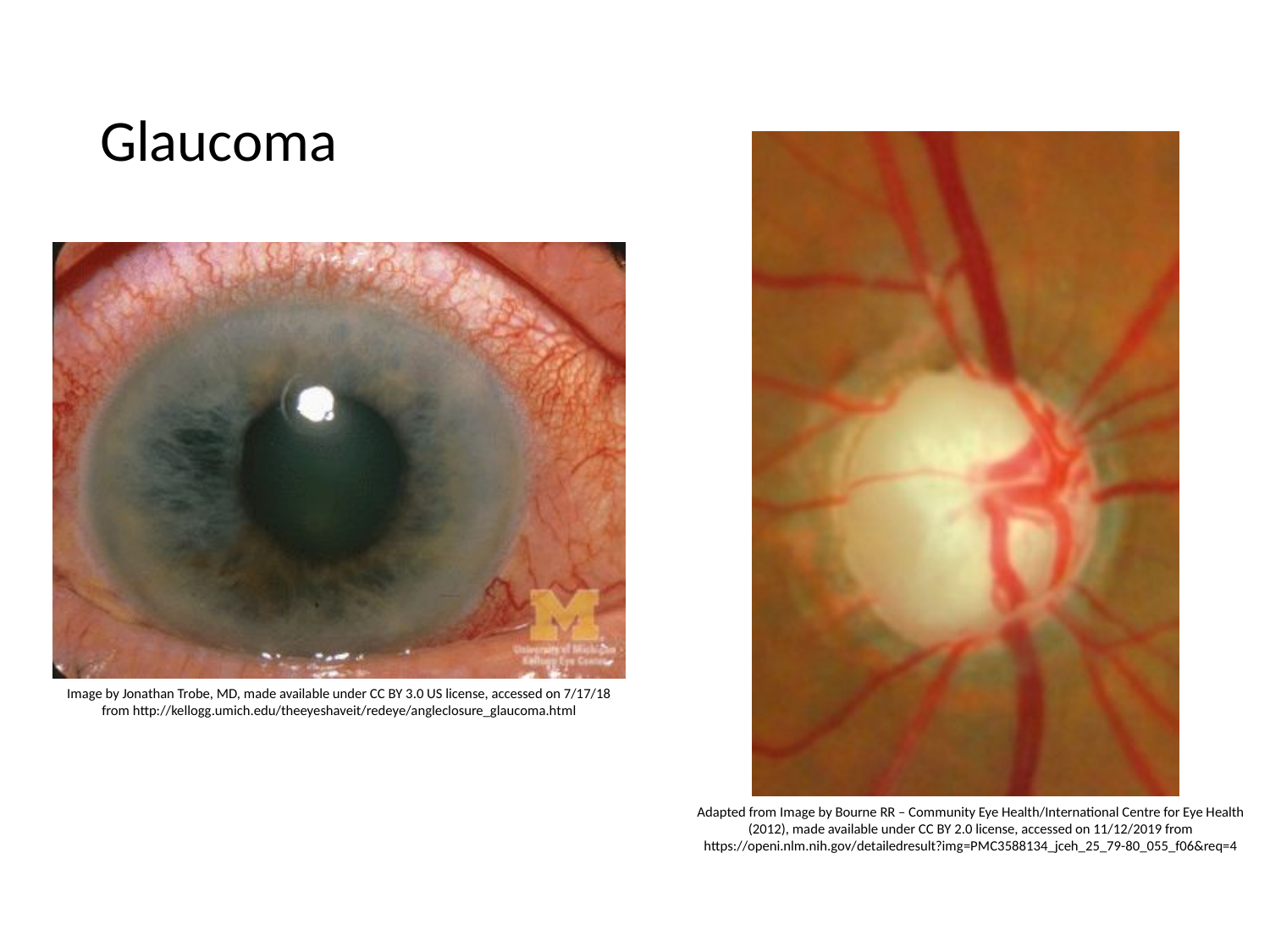

# Glaucoma
Image by Jonathan Trobe, MD, made available under CC BY 3.0 US license, accessed on 7/17/18 from http://kellogg.umich.edu/theeyeshaveit/redeye/angleclosure_glaucoma.html
Adapted from Image by Bourne RR – Community Eye Health/International Centre for Eye Health (2012), made available under CC BY 2.0 license, accessed on 11/12/2019 from https://openi.nlm.nih.gov/detailedresult?img=PMC3588134_jceh_25_79-80_055_f06&req=4

## Slide 23
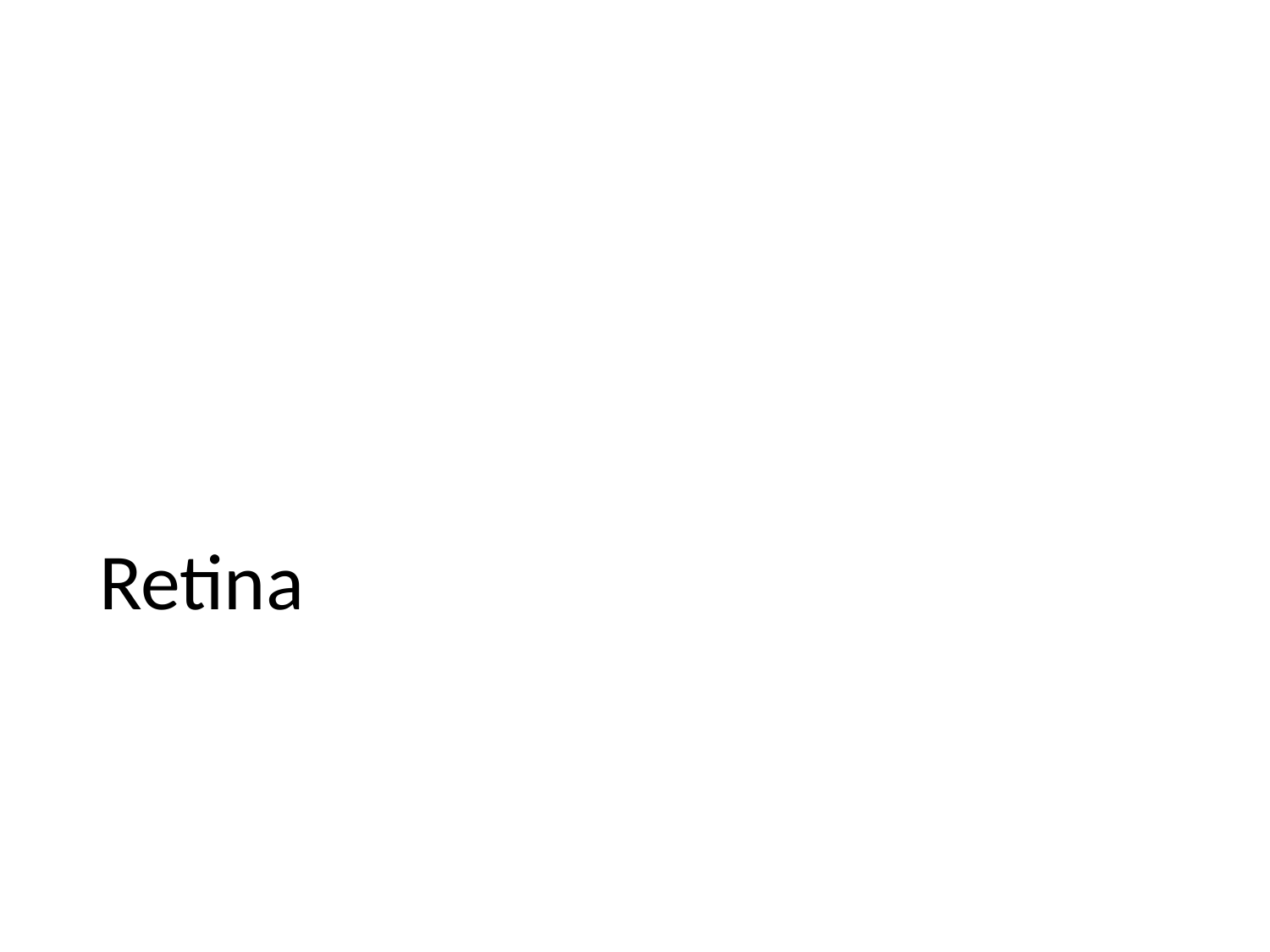

# Retina

## Slide 24
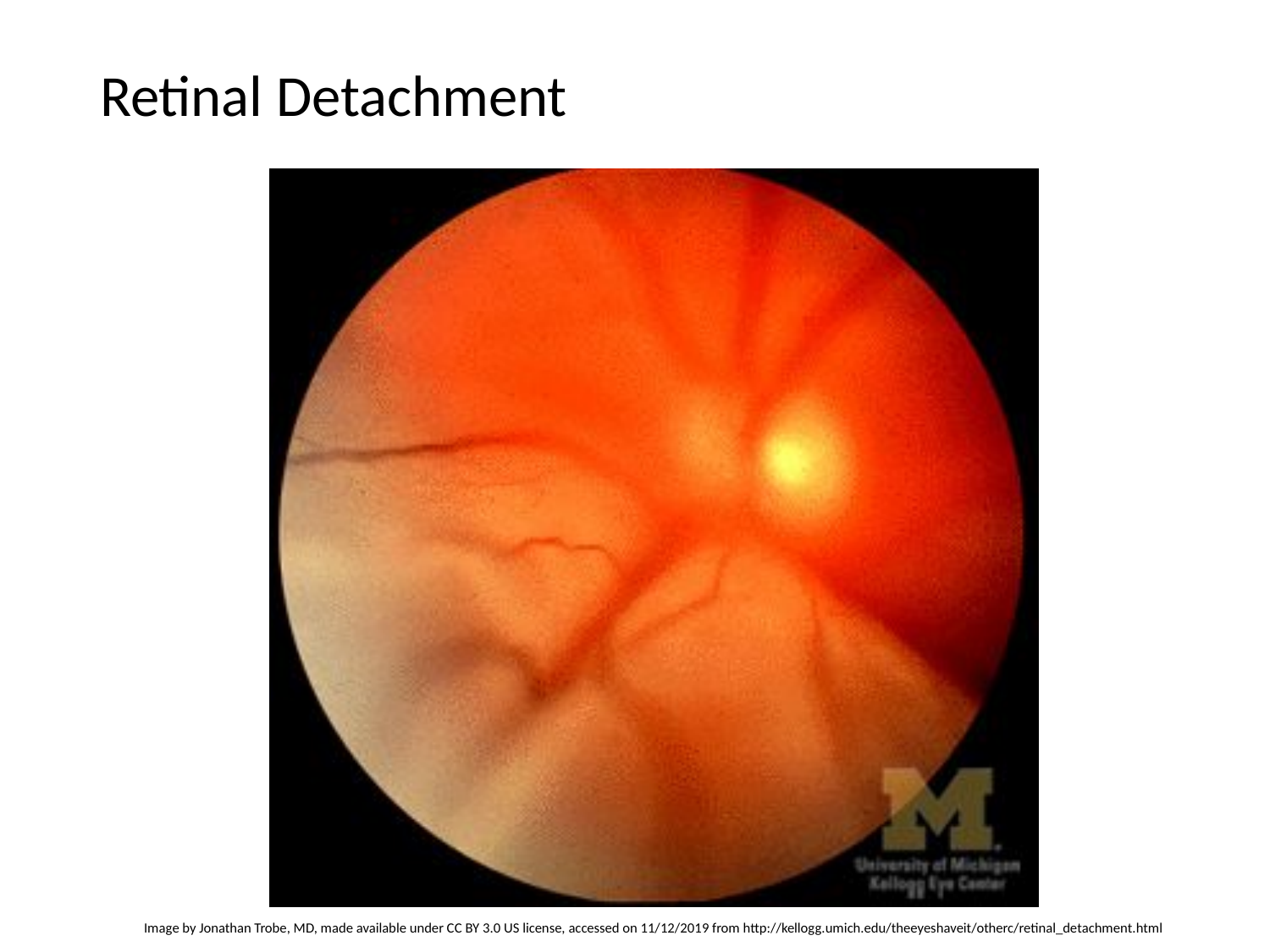

# Retinal Detachment
Image by Jonathan Trobe, MD, made available under CC BY 3.0 US license, accessed on 11/12/2019 from http://kellogg.umich.edu/theeyeshaveit/otherc/retinal_detachment.html

## Slide 25
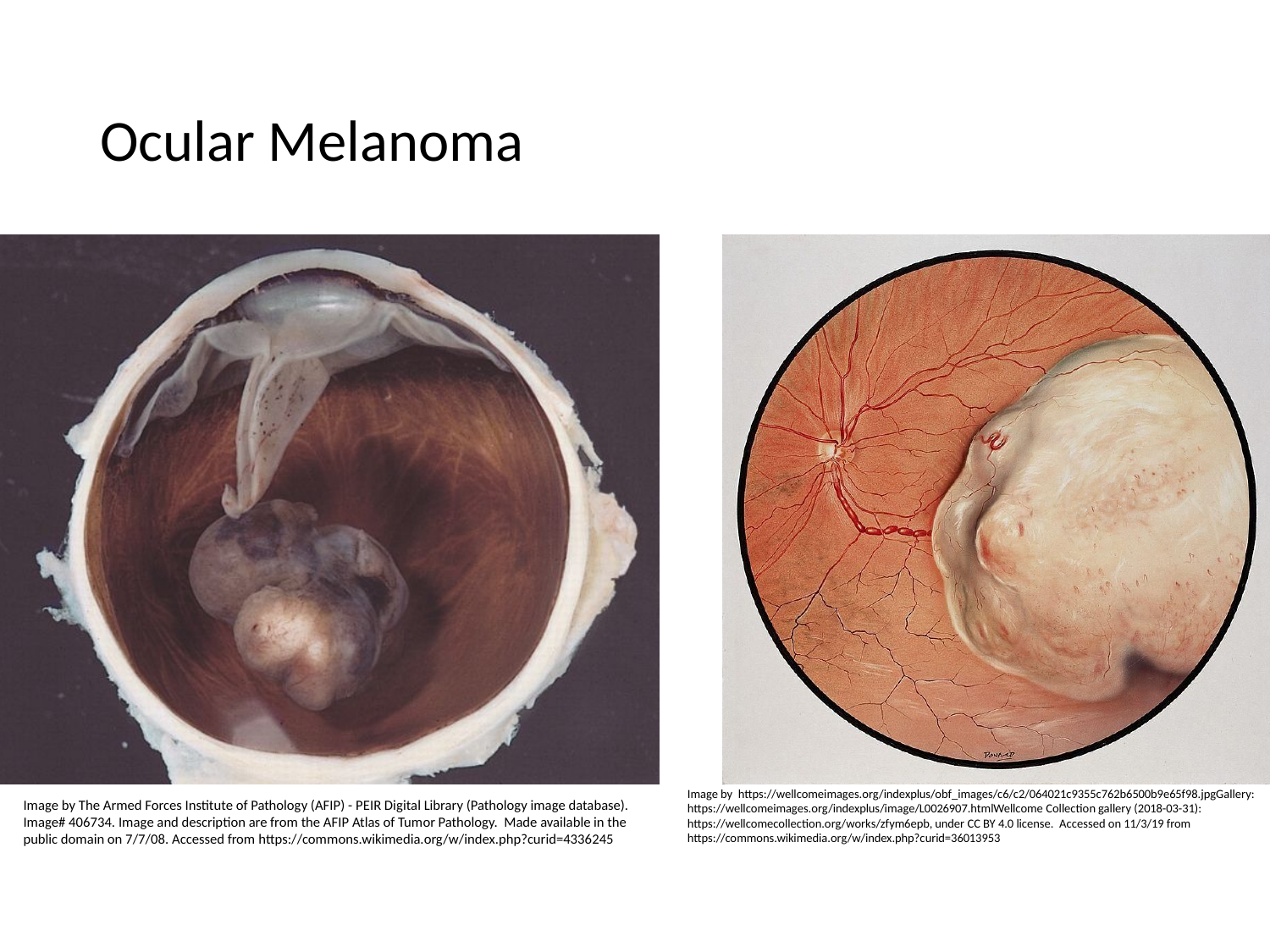

# Ocular Melanoma
Image by https://wellcomeimages.org/indexplus/obf_images/c6/c2/064021c9355c762b6500b9e65f98.jpgGallery: https://wellcomeimages.org/indexplus/image/L0026907.htmlWellcome Collection gallery (2018-03-31): https://wellcomecollection.org/works/zfym6epb, under CC BY 4.0 license. Accessed on 11/3/19 from https://commons.wikimedia.org/w/index.php?curid=36013953
Image by The Armed Forces Institute of Pathology (AFIP) - PEIR Digital Library (Pathology image database). Image# 406734. Image and description are from the AFIP Atlas of Tumor Pathology. Made available in the public domain on 7/7/08. Accessed from https://commons.wikimedia.org/w/index.php?curid=4336245

## Slide 26
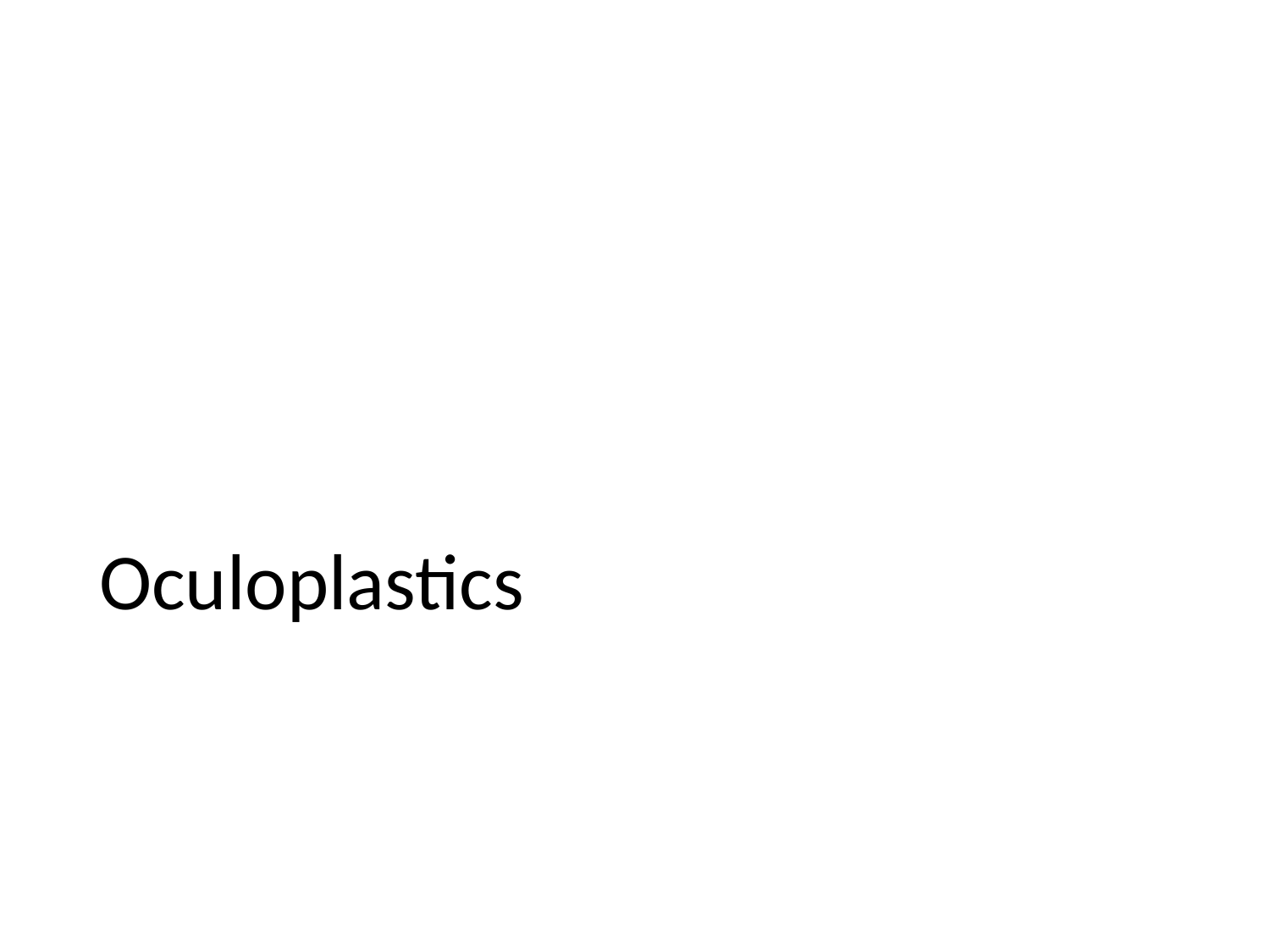

# Oculoplastics

## Slide 27
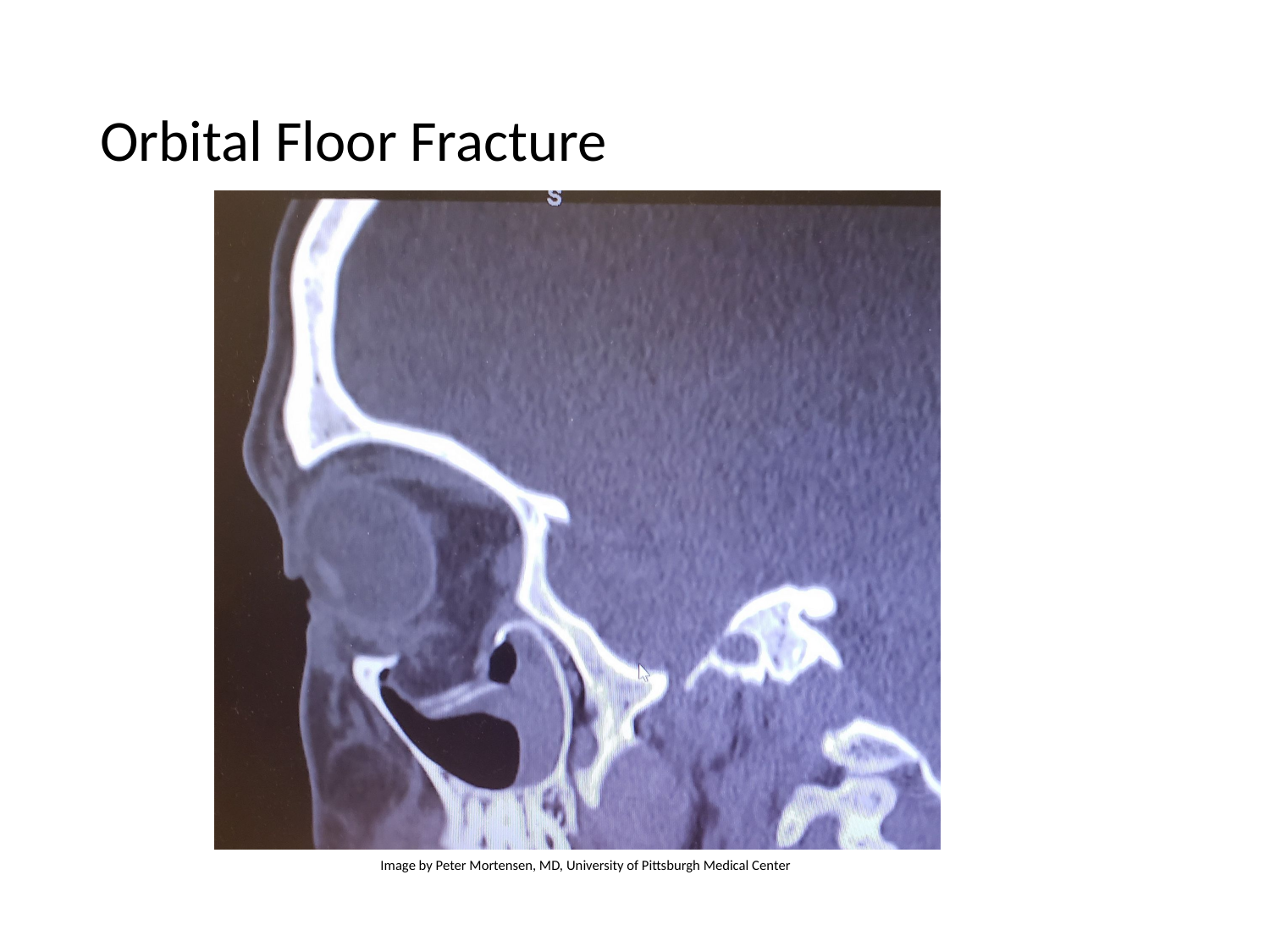

# Orbital Floor Fracture
Image by Peter Mortensen, MD, University of Pittsburgh Medical Center

## Slide 28
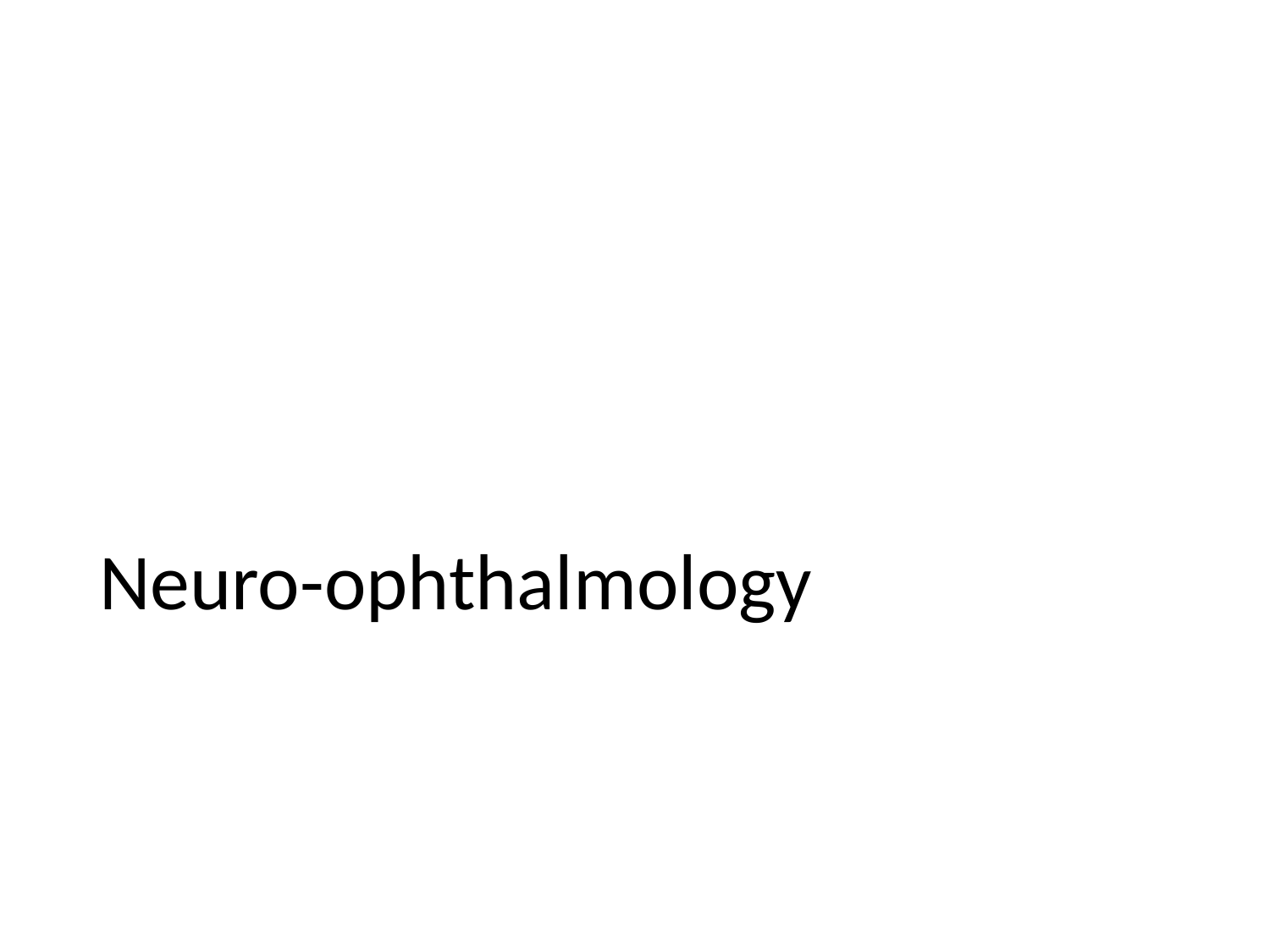

# Neuro-ophthalmology

## Slide 29
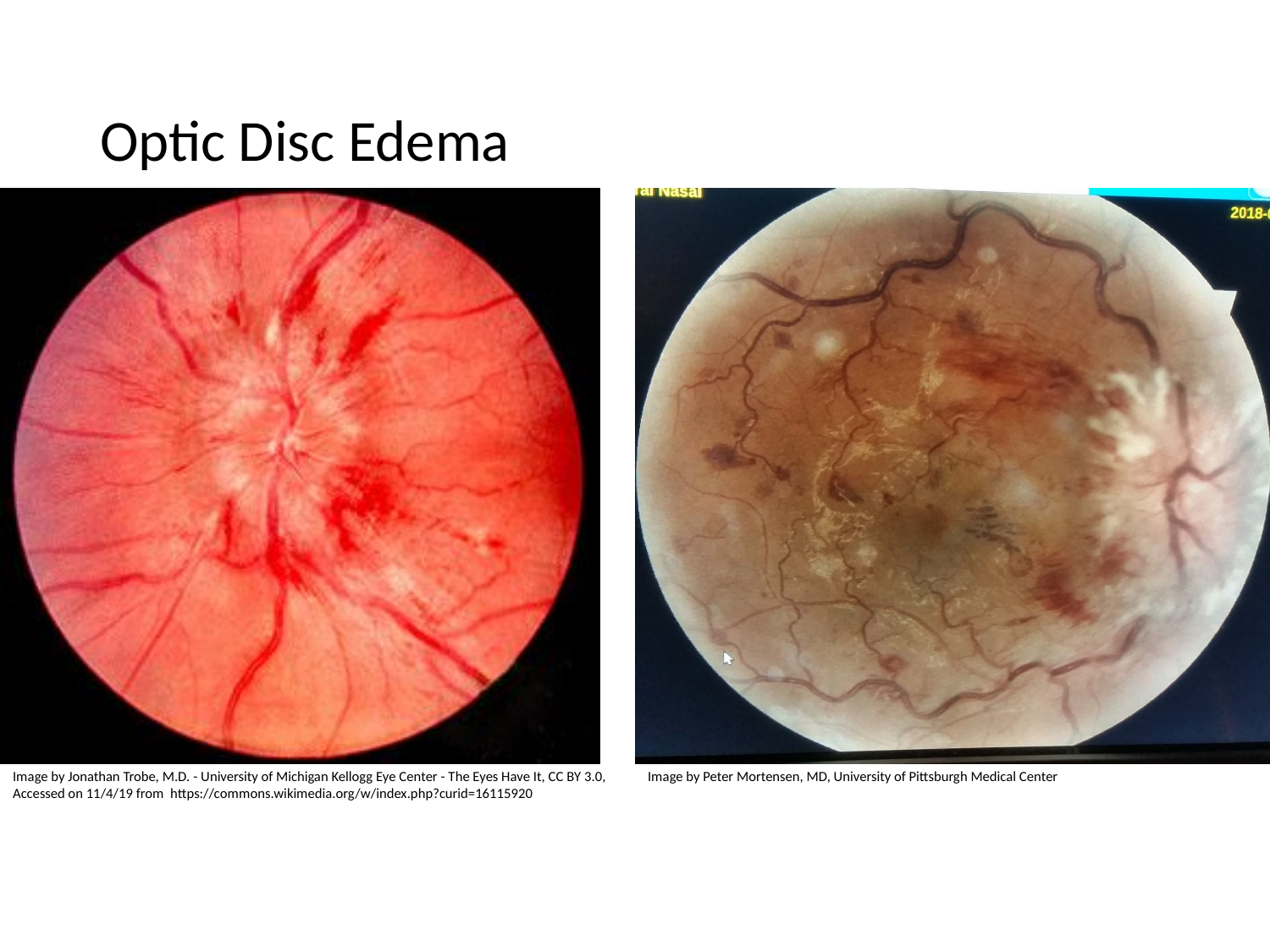

# Optic Disc Edema
Image by Jonathan Trobe, M.D. - University of Michigan Kellogg Eye Center - The Eyes Have It, CC BY 3.0, Accessed on 11/4/19 from https://commons.wikimedia.org/w/index.php?curid=16115920
Image by Peter Mortensen, MD, University of Pittsburgh Medical Center

## Slide 30
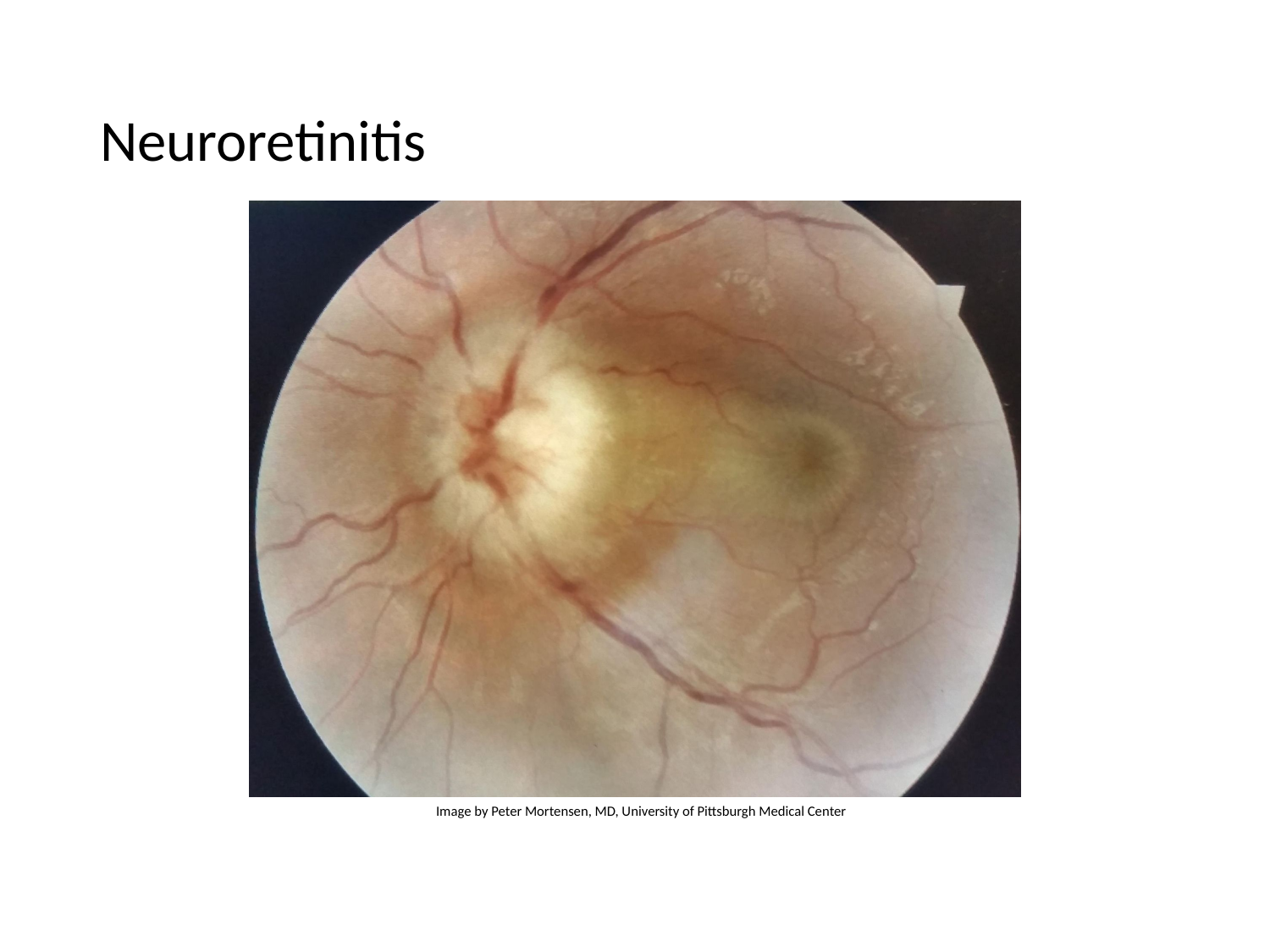

# Neuroretinitis
Image by Peter Mortensen, MD, University of Pittsburgh Medical Center

## Slide 31
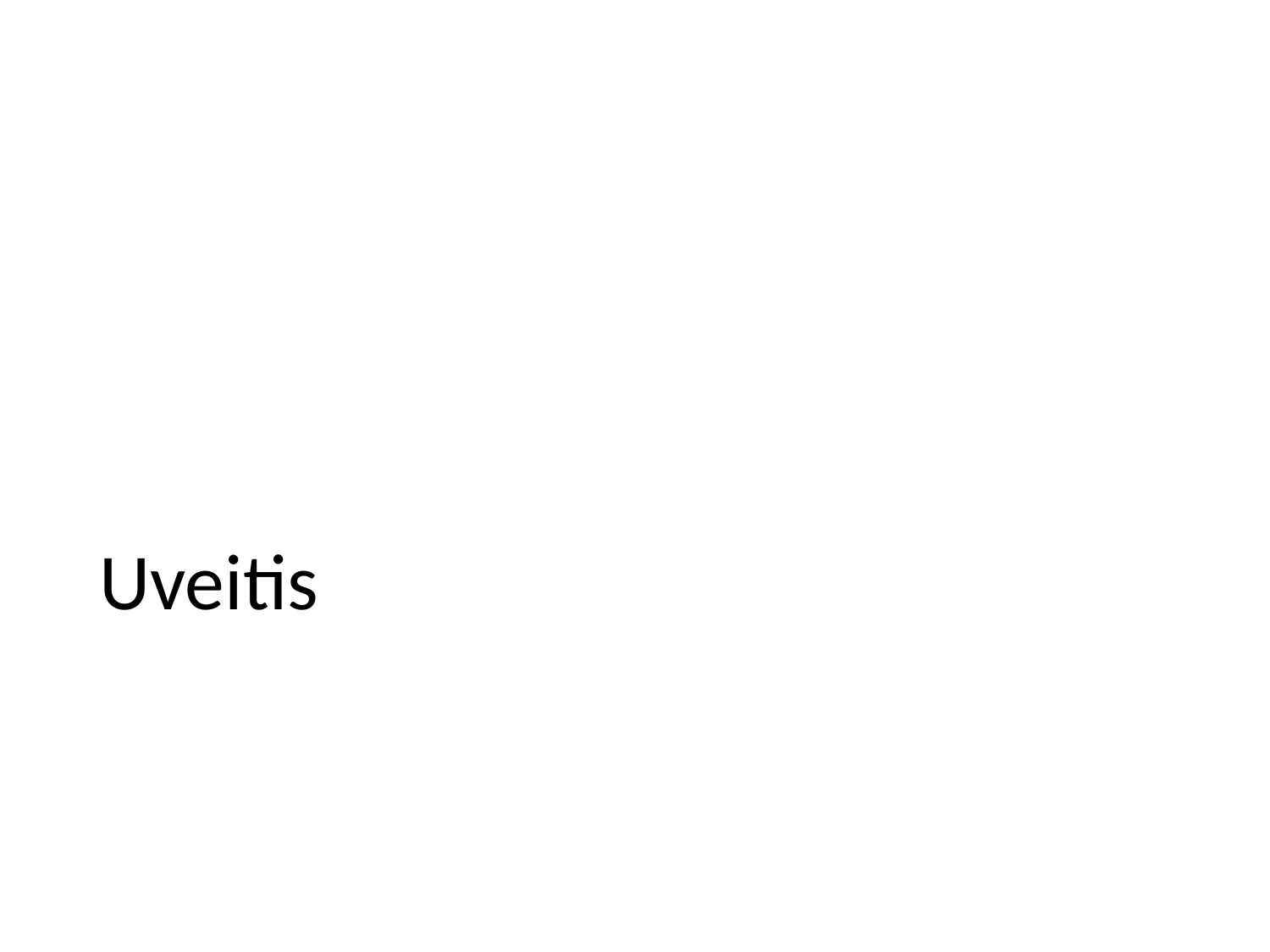

# Uveitis

## Slide 32
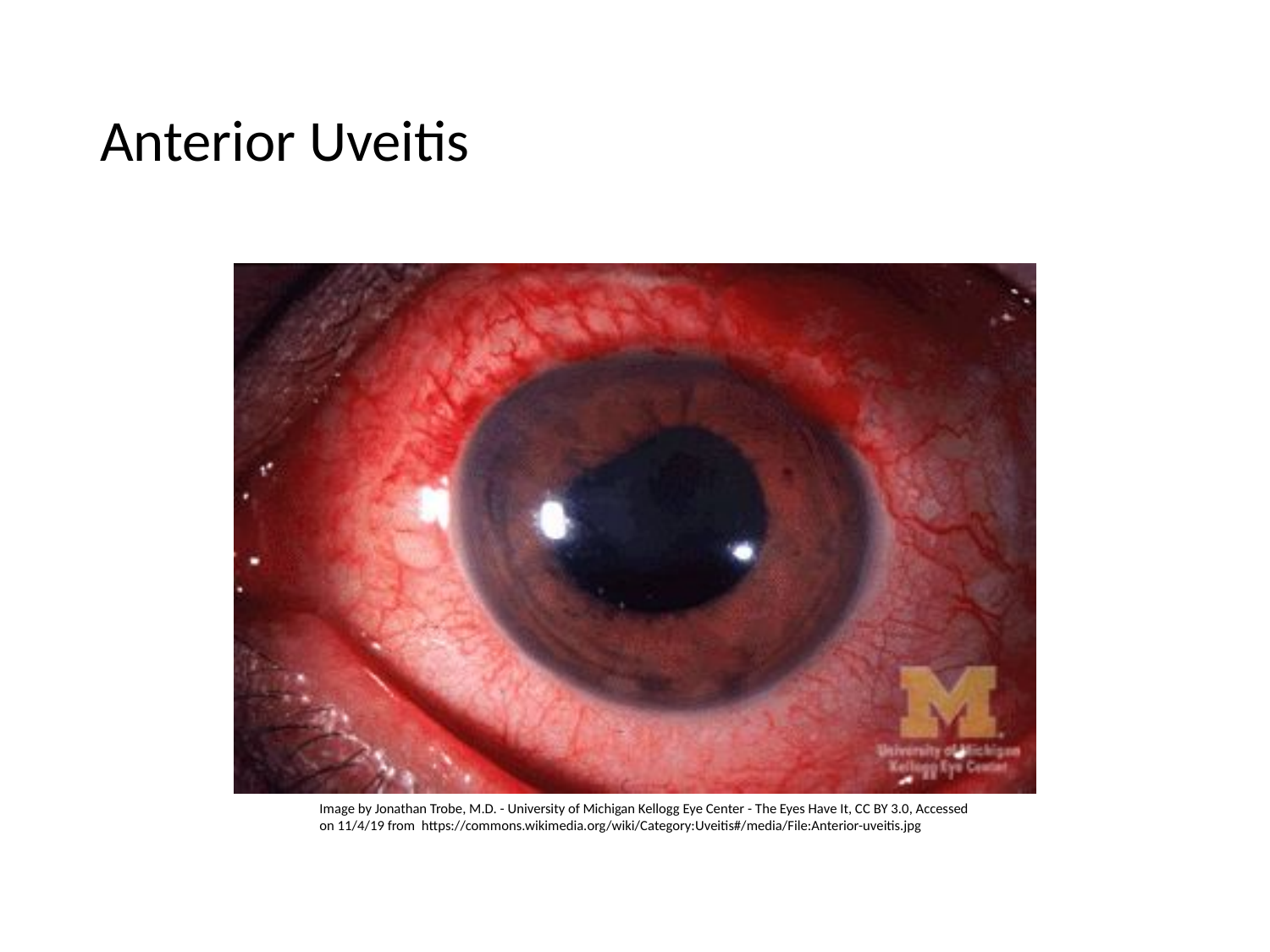

# Anterior Uveitis
Image by Jonathan Trobe, M.D. - University of Michigan Kellogg Eye Center - The Eyes Have It, CC BY 3.0, Accessed on 11/4/19 from https://commons.wikimedia.org/wiki/Category:Uveitis#/media/File:Anterior-uveitis.jpg

## Slide 33
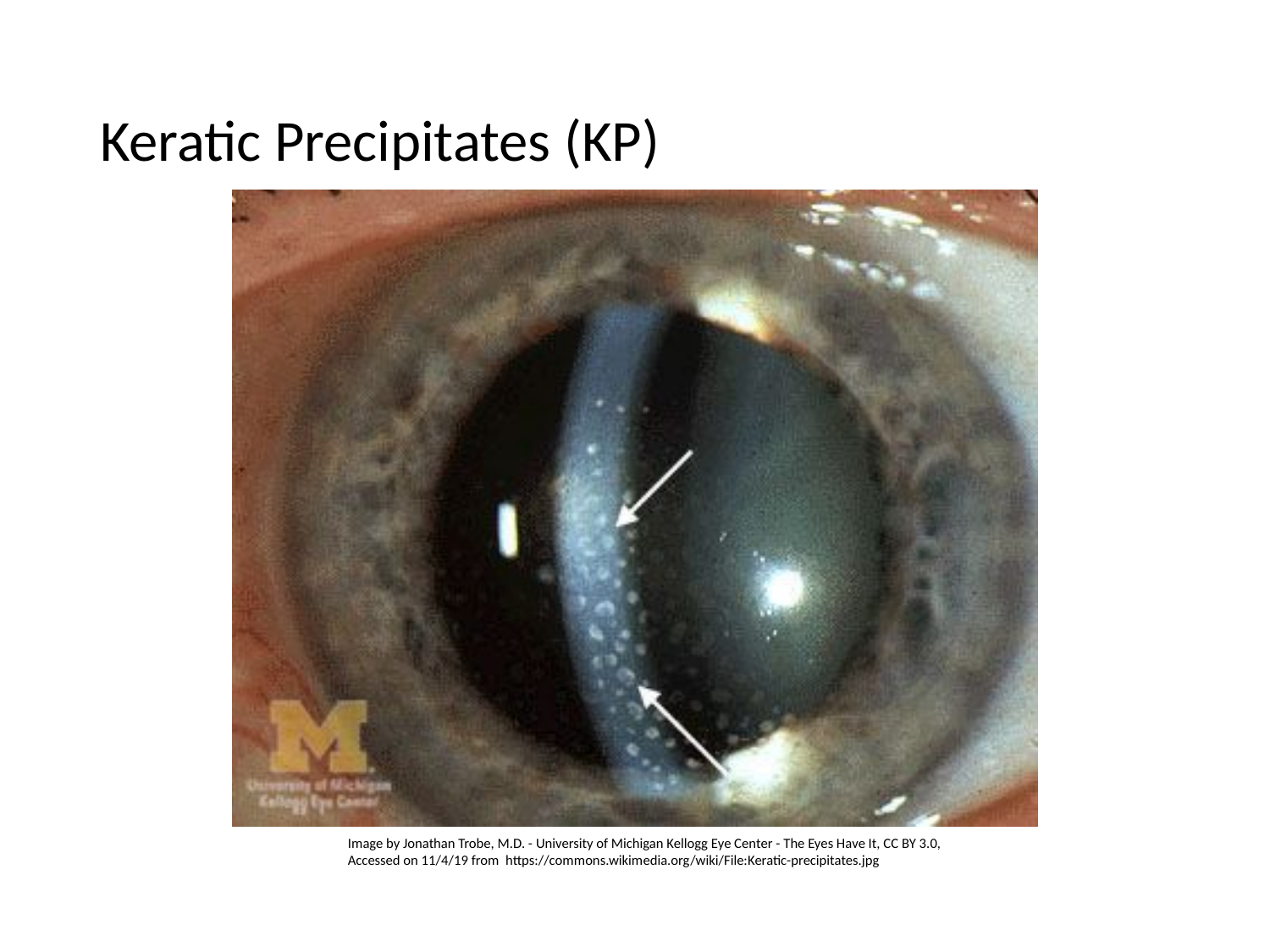

# Keratic Precipitates (KP)
Image by Jonathan Trobe, M.D. - University of Michigan Kellogg Eye Center - The Eyes Have It, CC BY 3.0, Accessed on 11/4/19 from https://commons.wikimedia.org/wiki/File:Keratic-precipitates.jpg

## Slide 34
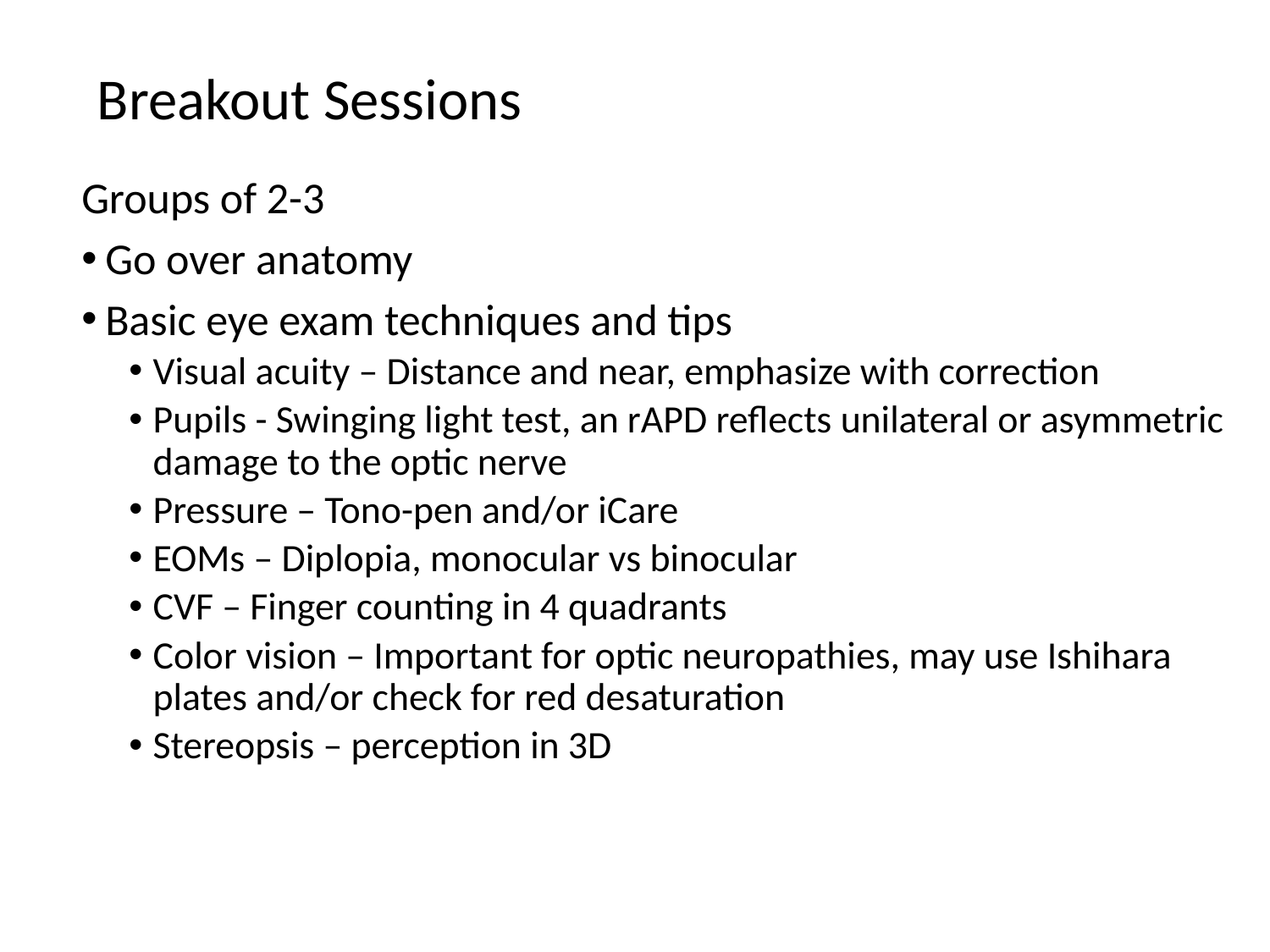

# Breakout Sessions
Groups of 2-3
Go over anatomy
Basic eye exam techniques and tips
Visual acuity – Distance and near, emphasize with correction
Pupils - Swinging light test, an rAPD reflects unilateral or asymmetric damage to the optic nerve
Pressure – Tono-pen and/or iCare
EOMs – Diplopia, monocular vs binocular
CVF – Finger counting in 4 quadrants
Color vision – Important for optic neuropathies, may use Ishihara plates and/or check for red desaturation
Stereopsis – perception in 3D
