# Supplementary material for: The Ophthalmology Mini-Elective Gives Vision to Preclinical Medical Students
Source: MedEdPORTAL. 2020 Nov 23;16:11024. doi: 10.15766/mep_2374-8265.11024 (PMC7703479; doi:10.15766/mep_2374-8265.11024)
Supplement: Supplementary file 1 — Course Syllabus.docxInstructor Introduction.docxWeekly Course Time Line & Objectives.docxSession 1 - Intro to Ophthalmology.pptxSession 2 - Anterior Segment.pptxSession 3 - Posterior Segment.pptxSession 4 - Eye Emergencies and Trauma.pptxLaboratory Session Guide.pdfPrecourse Survey.docxPre- and Posttest.docxPostcourse Survey.docxPre- and Posttest Answers.docx [file mep_2374-8265.11024-s001.zip › E. Session 2 - Anterior Segment.pptx]

## Slide 1
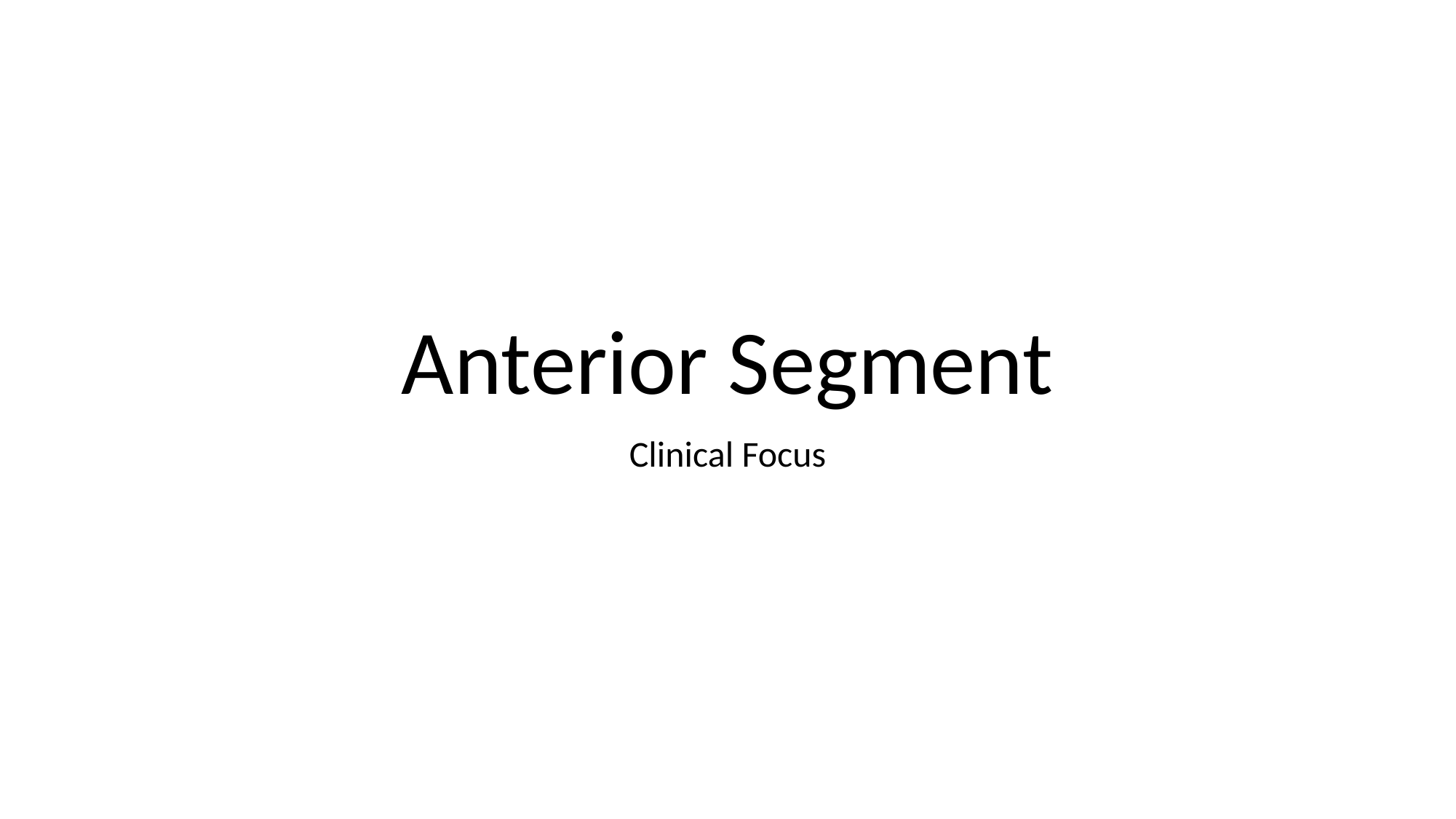

# Anterior Segment
Clinical Focus

## Slide 2
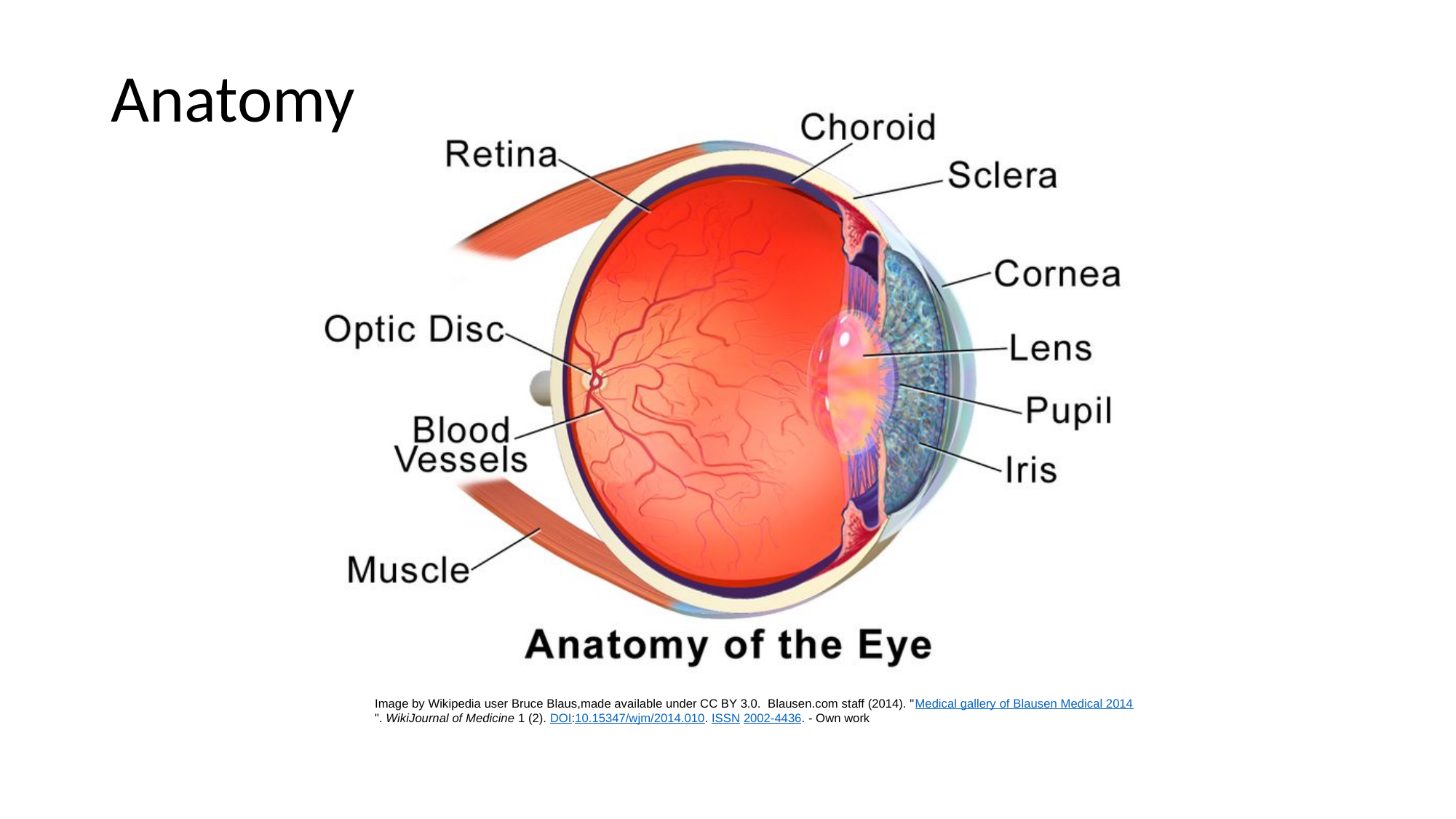

# Anatomy
Image by Wikipedia user Bruce Blaus,made available under CC BY 3.0. Blausen.com staff (2014). "Medical gallery of Blausen Medical 2014". WikiJournal of Medicine 1 (2). DOI:10.15347/wjm/2014.010. ISSN 2002-4436. - Own work

## Slide 3
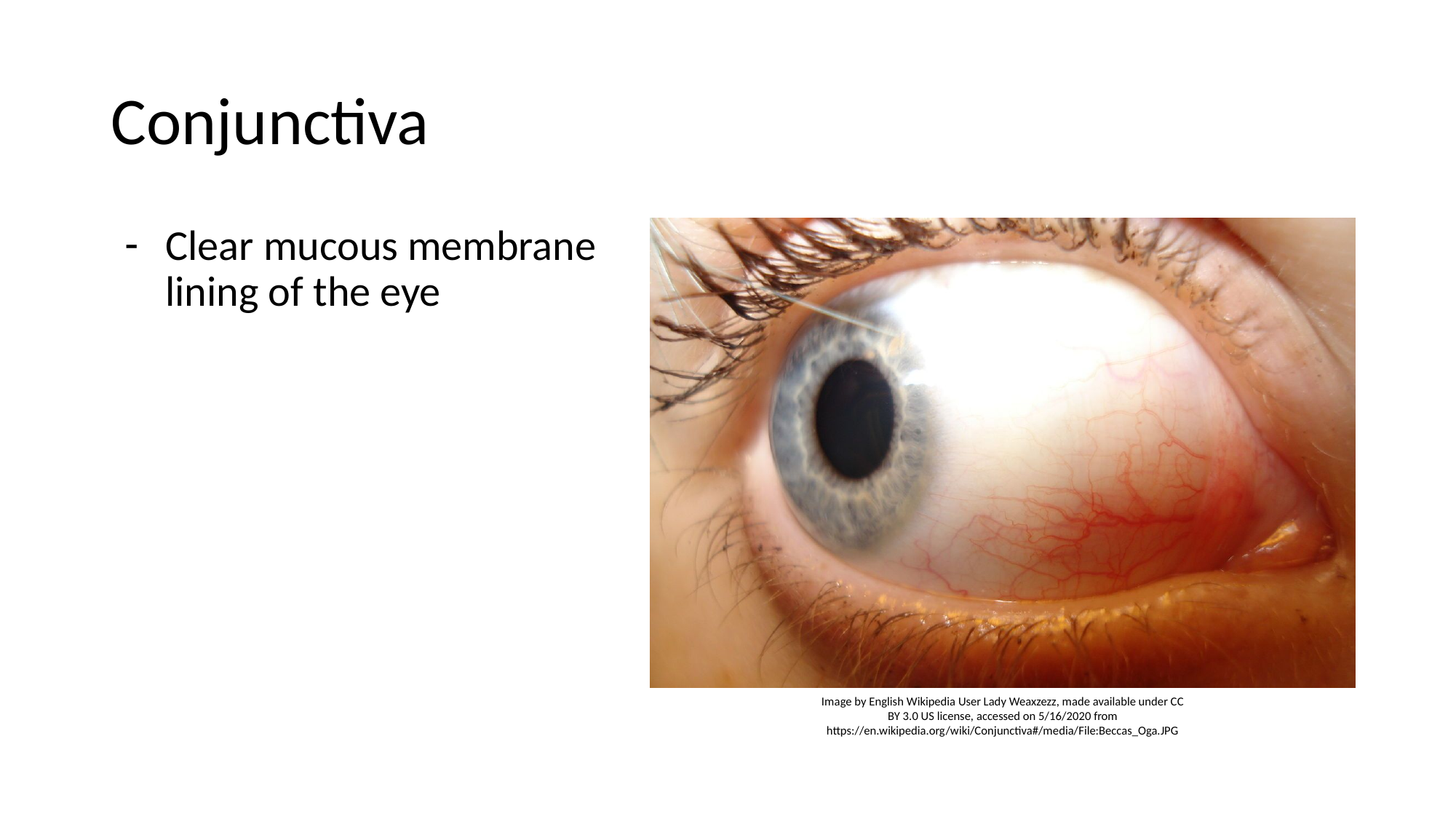

# Conjunctiva
Clear mucous membrane
lining of the eye
Image by English Wikipedia User Lady Weaxzezz, made available under CC BY 3.0 US license, accessed on 5/16/2020 from https://en.wikipedia.org/wiki/Conjunctiva#/media/File:Beccas_Oga.JPG

## Slide 4
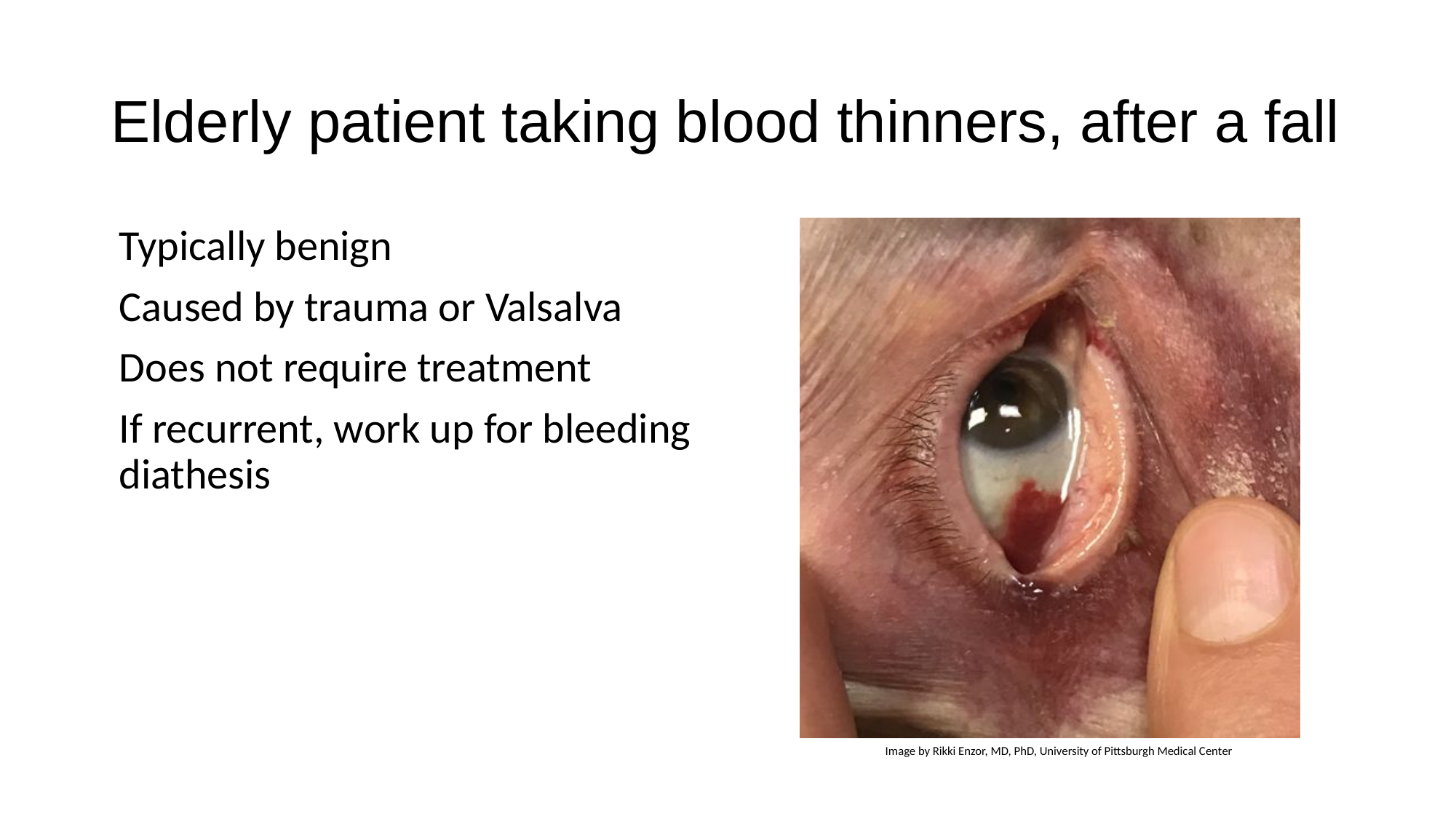

# Elderly patient taking blood thinners, after a fall
Typically benign
Caused by trauma or Valsalva
Does not require treatment
If recurrent, work up for bleeding
diathesis
Image by Rikki Enzor, MD, PhD, University of Pittsburgh Medical Center

## Slide 5
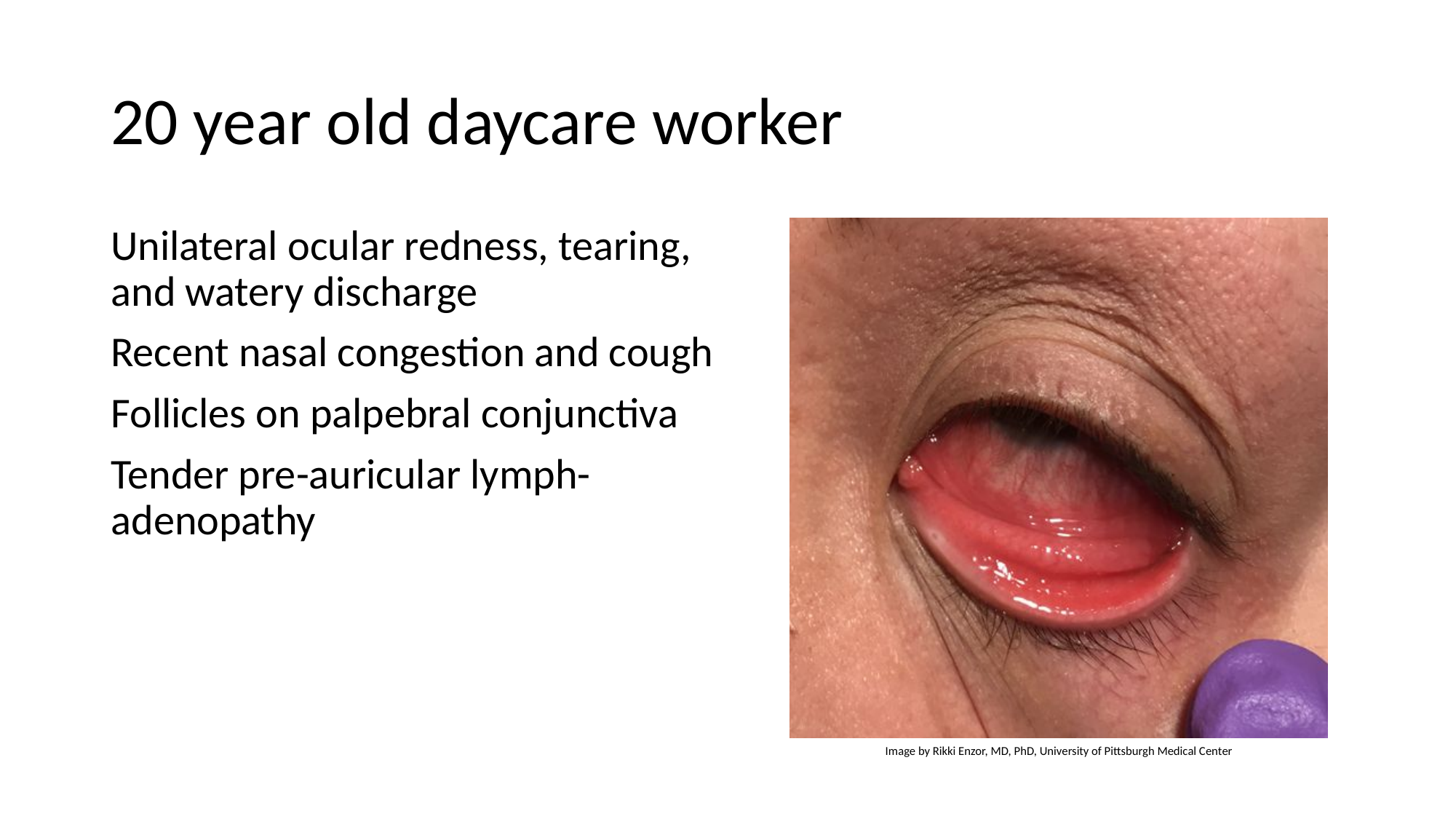

# 20 year old daycare worker
Unilateral ocular redness, tearing,
and watery discharge
Recent nasal congestion and cough
Follicles on palpebral conjunctiva
Tender pre-auricular lymph-
adenopathy
Image by Rikki Enzor, MD, PhD, University of Pittsburgh Medical Center

## Slide 6
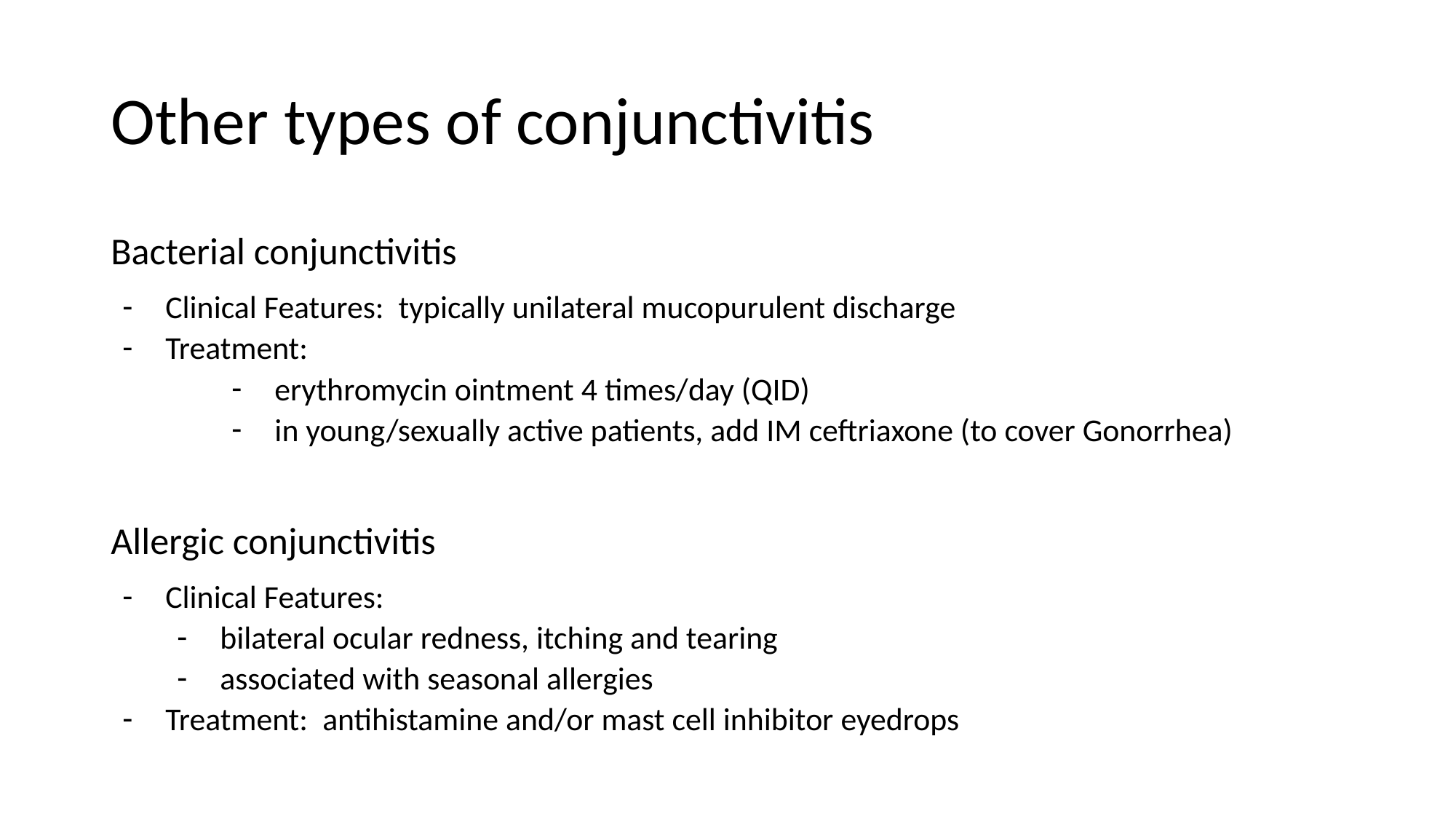

# Other types of conjunctivitis
Bacterial conjunctivitis
Clinical Features: typically unilateral mucopurulent discharge
Treatment:
erythromycin ointment 4 times/day (QID)
in young/sexually active patients, add IM ceftriaxone (to cover Gonorrhea)
Allergic conjunctivitis
Clinical Features:
bilateral ocular redness, itching and tearing
associated with seasonal allergies
Treatment: antihistamine and/or mast cell inhibitor eyedrops

## Slide 7
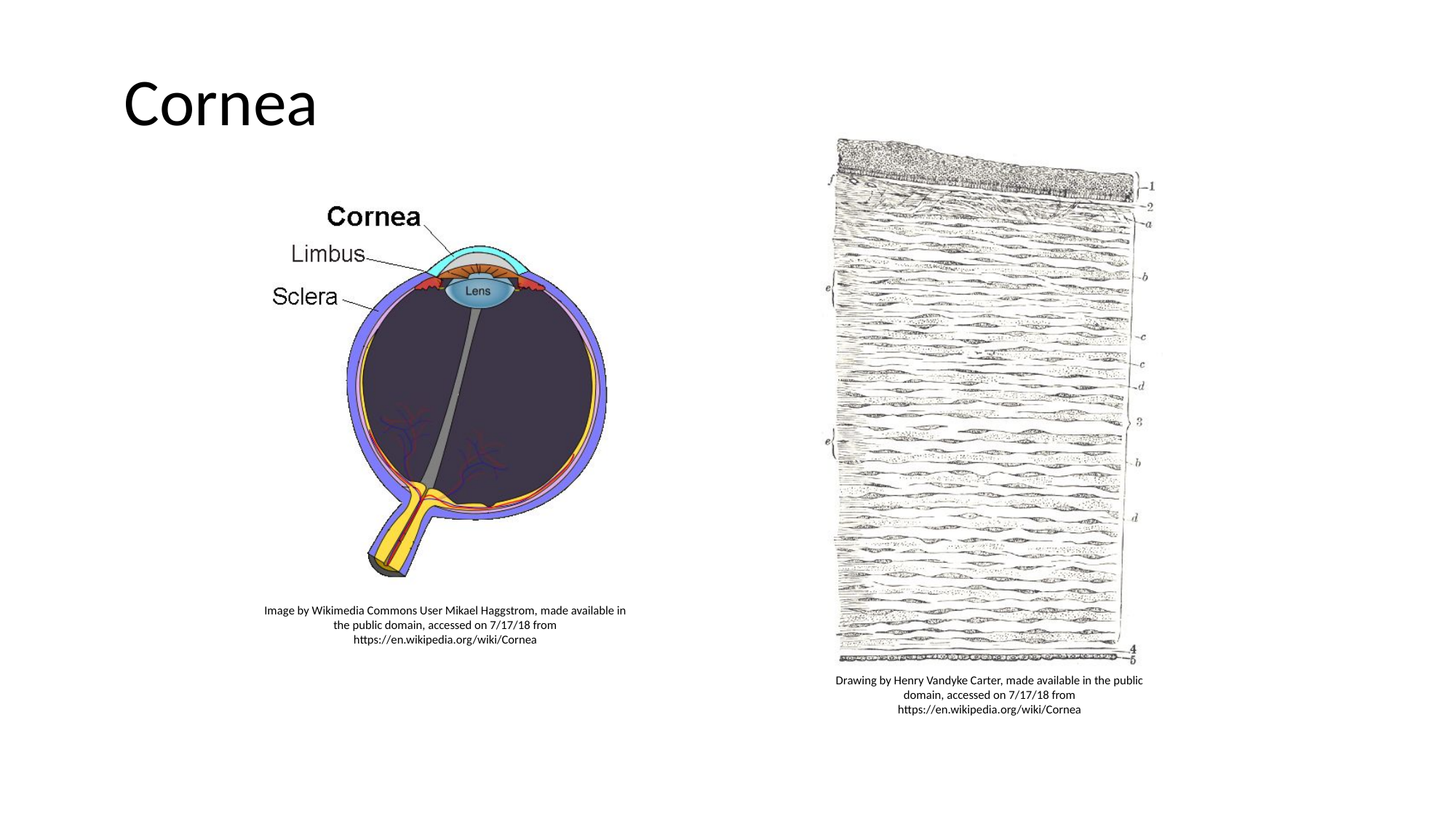

# Cornea
Drawing by Henry Vandyke Carter, made available in the public domain, accessed on 7/17/18 from https://en.wikipedia.org/wiki/Cornea
Image by Wikimedia Commons User Mikael Haggstrom, made available in the public domain, accessed on 7/17/18 from https://en.wikipedia.org/wiki/Cornea

## Slide 8
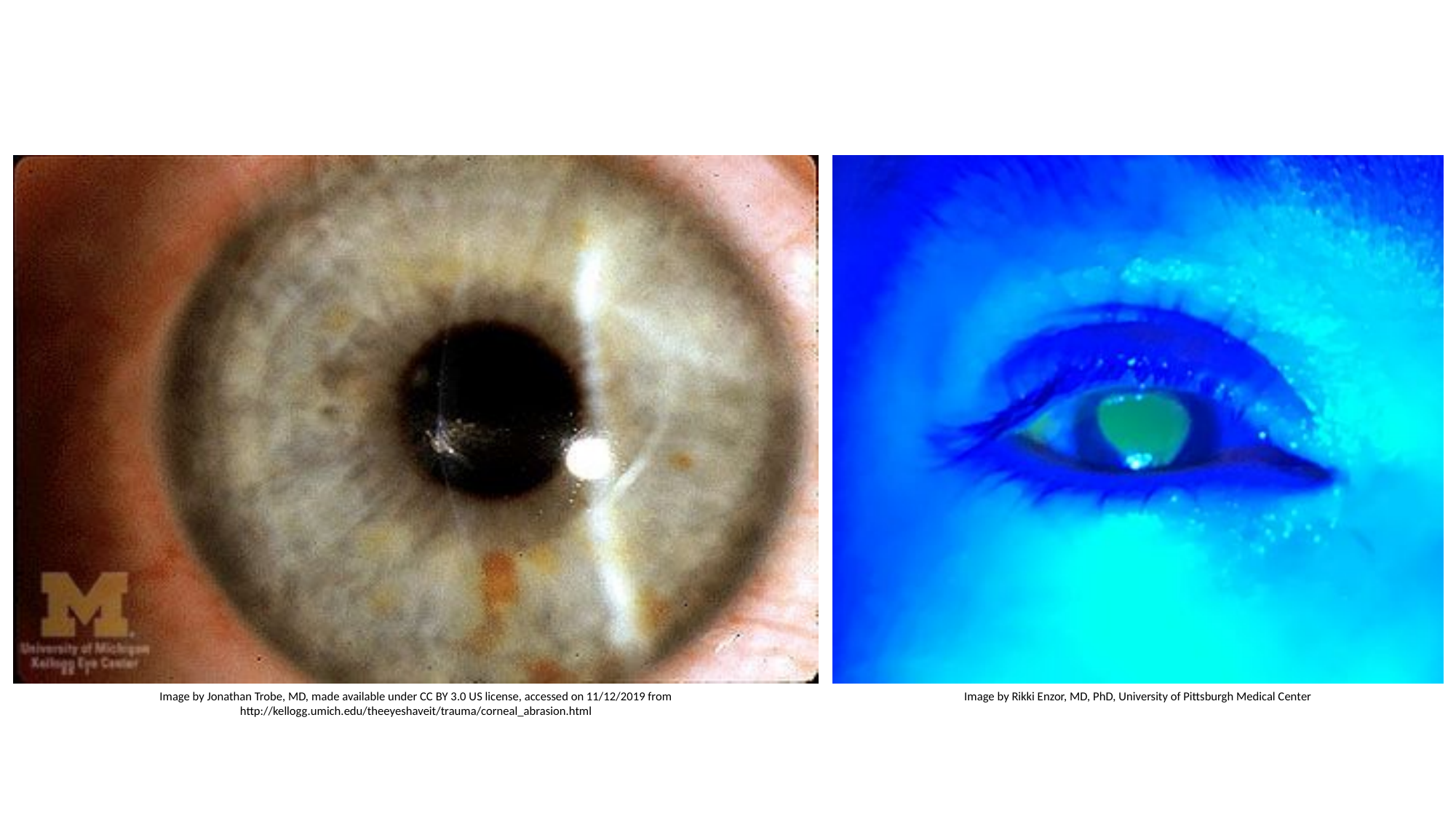

Image by Jonathan Trobe, MD, made available under CC BY 3.0 US license, accessed on 11/12/2019 from http://kellogg.umich.edu/theeyeshaveit/trauma/corneal_abrasion.html
Image by Rikki Enzor, MD, PhD, University of Pittsburgh Medical Center

## Slide 9
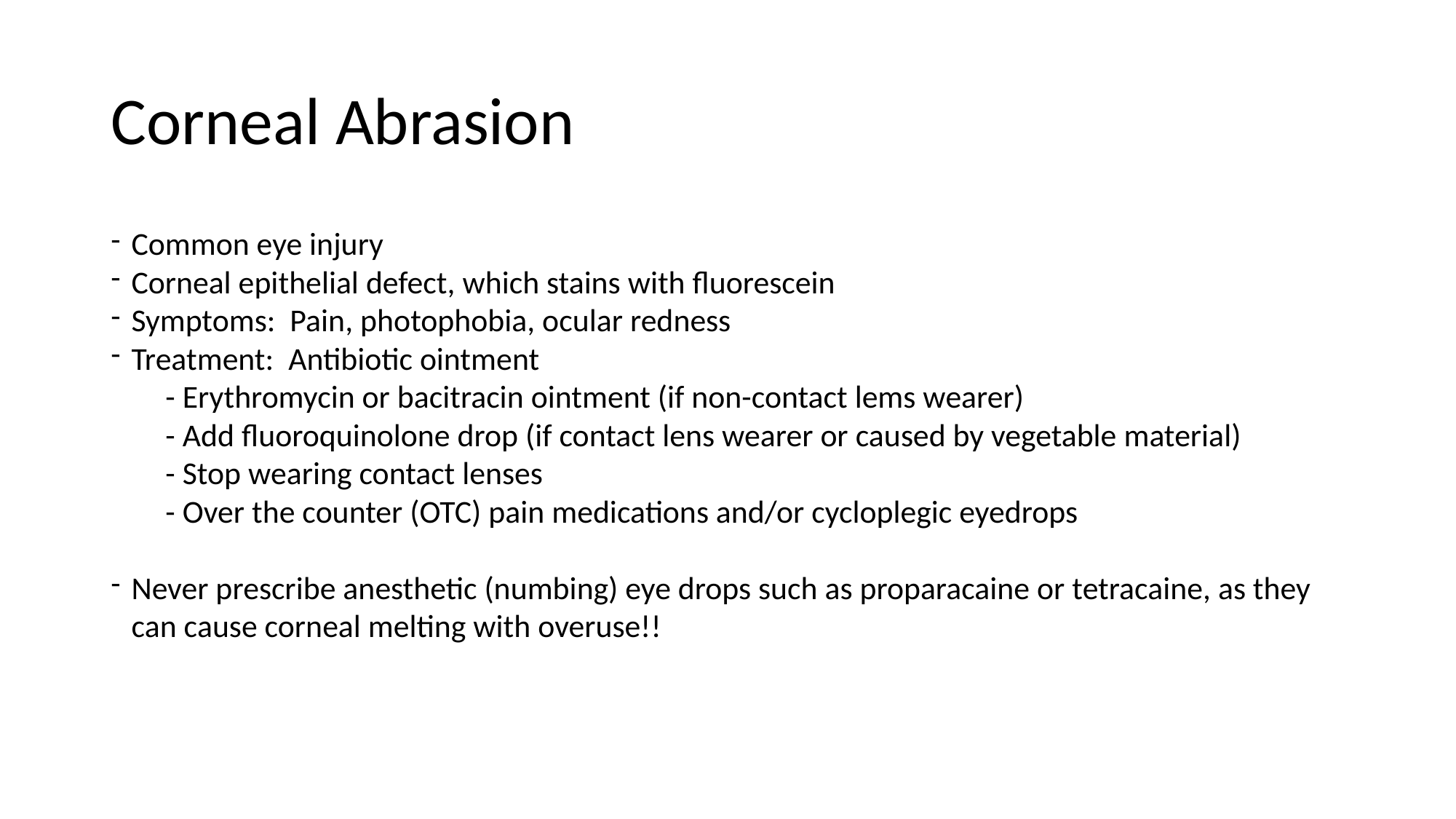

# Corneal Abrasion
Common eye injury
Corneal epithelial defect, which stains with fluorescein
Symptoms: Pain, photophobia, ocular redness
Treatment: Antibiotic ointment
- Erythromycin or bacitracin ointment (if non-contact lems wearer)
- Add fluoroquinolone drop (if contact lens wearer or caused by vegetable material)
- Stop wearing contact lenses
- Over the counter (OTC) pain medications and/or cycloplegic eyedrops
Never prescribe anesthetic (numbing) eye drops such as proparacaine or tetracaine, as they can cause corneal melting with overuse!!

## Slide 10
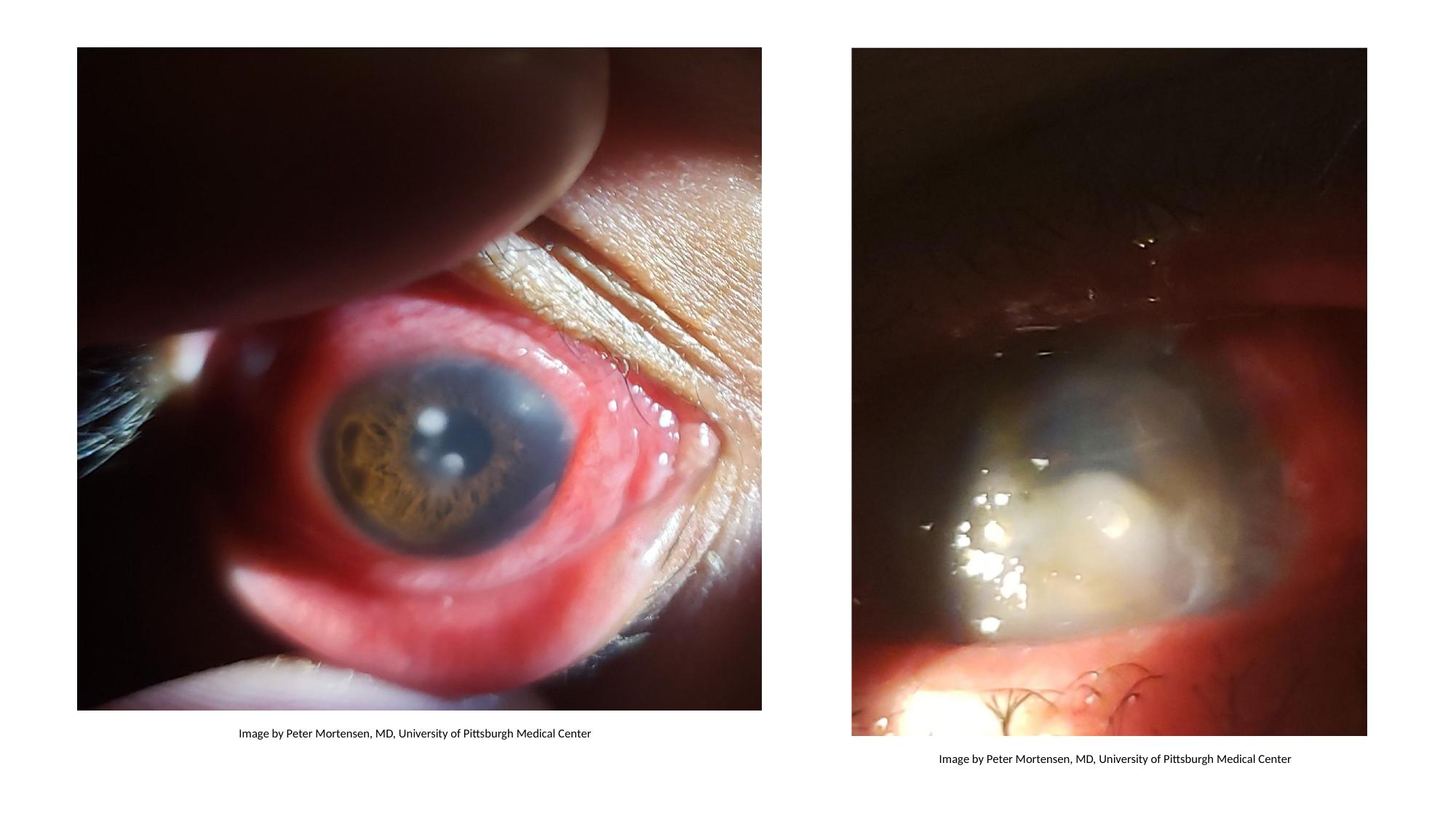

Image by Peter Mortensen, MD, University of Pittsburgh Medical Center
Image by Peter Mortensen, MD, University of Pittsburgh Medical Center

## Slide 11
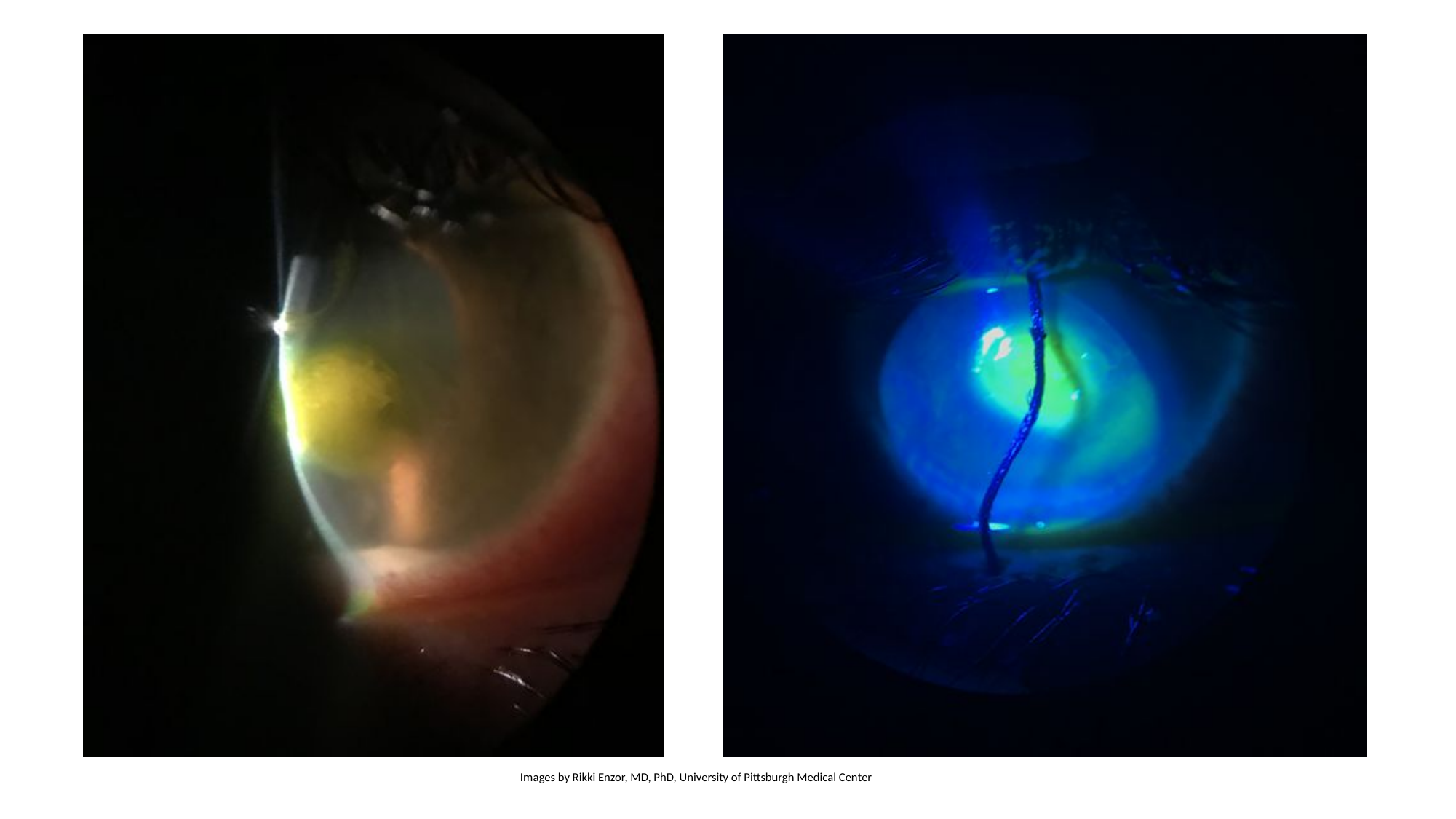

Images by Rikki Enzor, MD, PhD, University of Pittsburgh Medical Center

## Slide 12
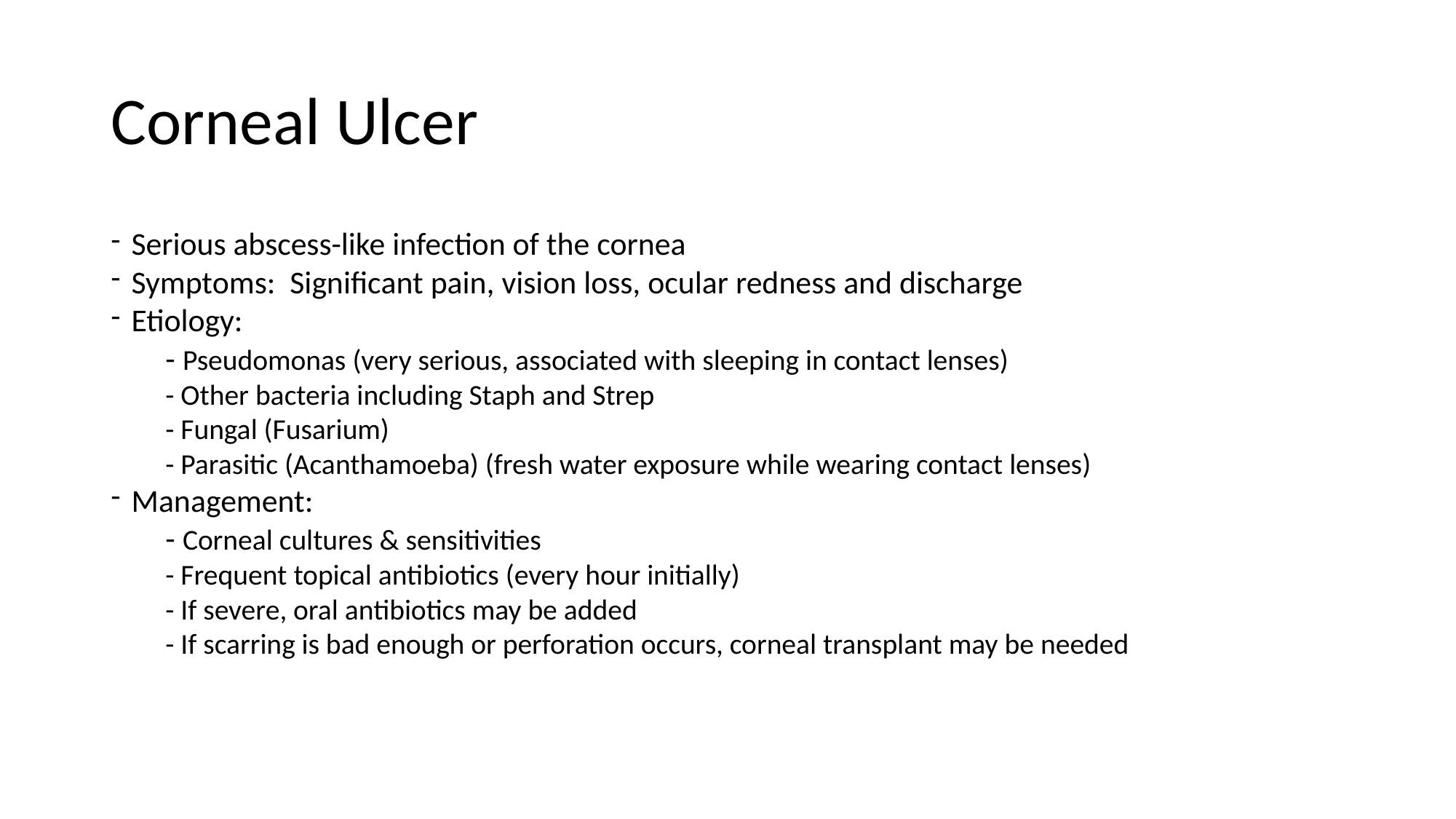

# Corneal Ulcer
Serious abscess-like infection of the cornea
Symptoms: Significant pain, vision loss, ocular redness and discharge
Etiology:
- Pseudomonas (very serious, associated with sleeping in contact lenses)
- Other bacteria including Staph and Strep
- Fungal (Fusarium)
- Parasitic (Acanthamoeba) (fresh water exposure while wearing contact lenses)
Management:
- Corneal cultures & sensitivities
- Frequent topical antibiotics (every hour initially)
- If severe, oral antibiotics may be added
- If scarring is bad enough or perforation occurs, corneal transplant may be needed

## Slide 13
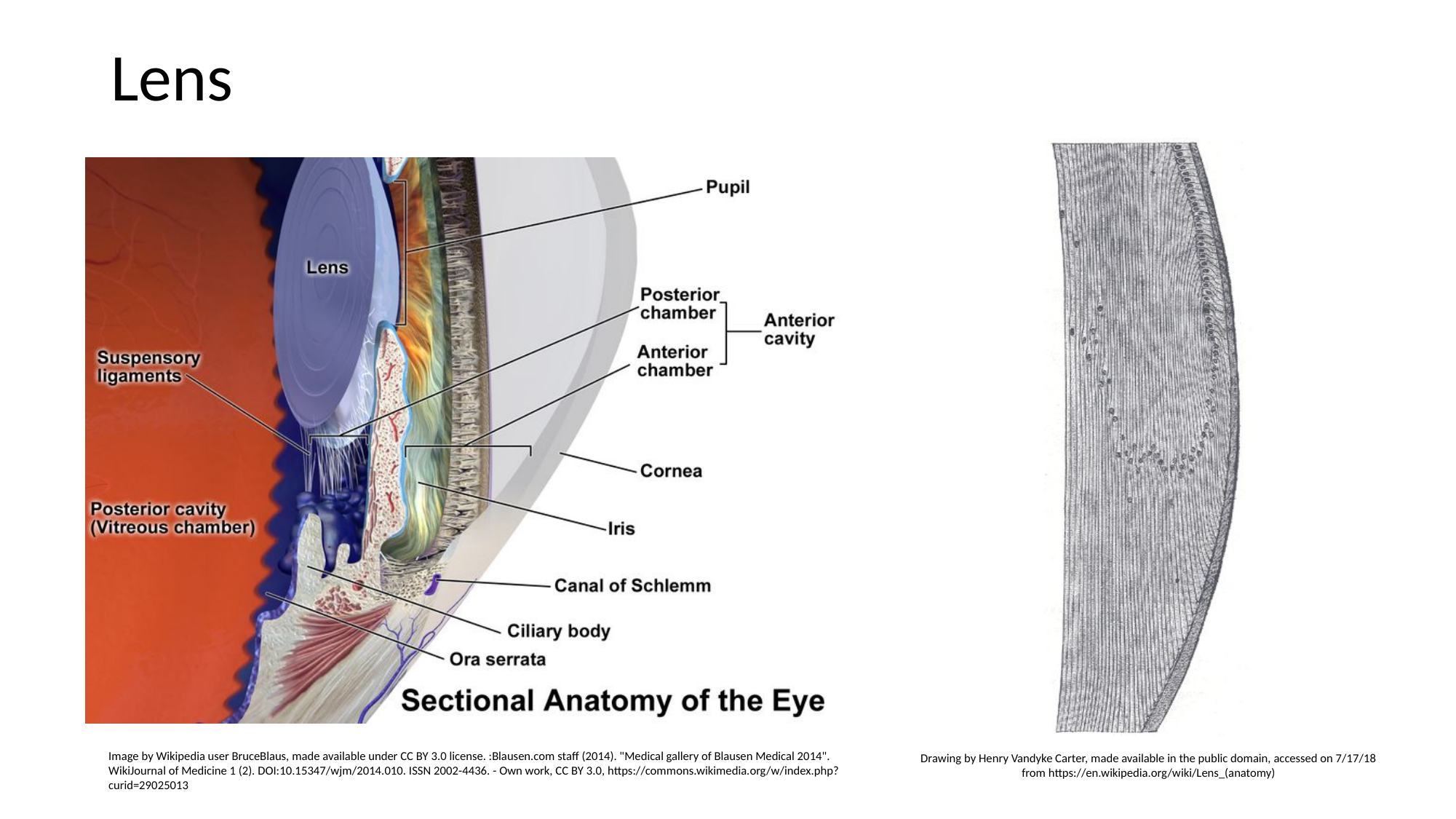

# Lens
Drawing by Henry Vandyke Carter, made available in the public domain, accessed on 7/17/18 from https://en.wikipedia.org/wiki/Lens_(anatomy)
Image by Wikipedia user BruceBlaus, made available under CC BY 3.0 license. :Blausen.com staff (2014). "Medical gallery of Blausen Medical 2014". WikiJournal of Medicine 1 (2). DOI:10.15347/wjm/2014.010. ISSN 2002-4436. - Own work, CC BY 3.0, https://commons.wikimedia.org/w/index.php?curid=29025013

## Slide 14
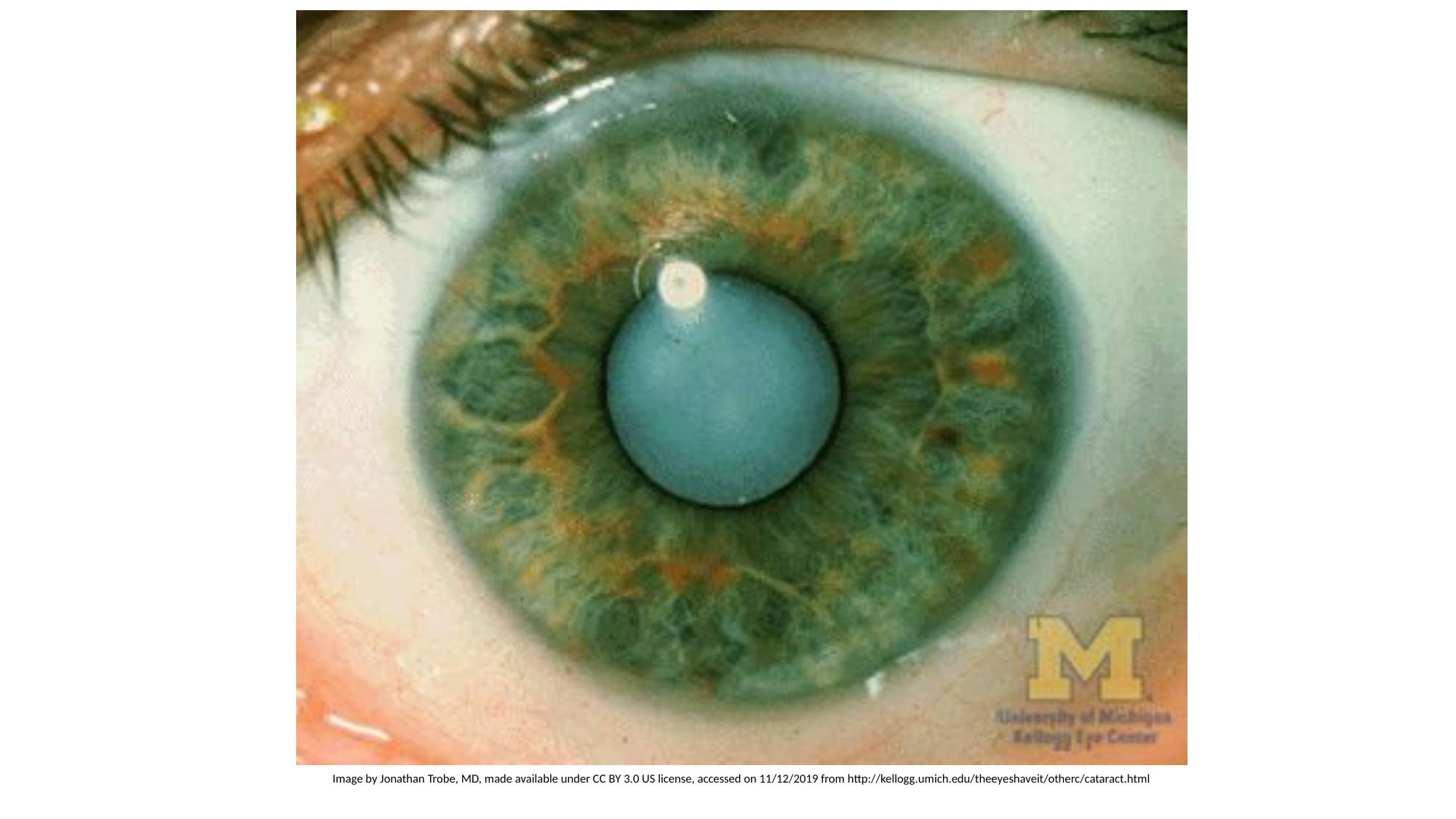

Image by Jonathan Trobe, MD, made available under CC BY 3.0 US license, accessed on 11/12/2019 from http://kellogg.umich.edu/theeyeshaveit/otherc/cataract.html

## Slide 15
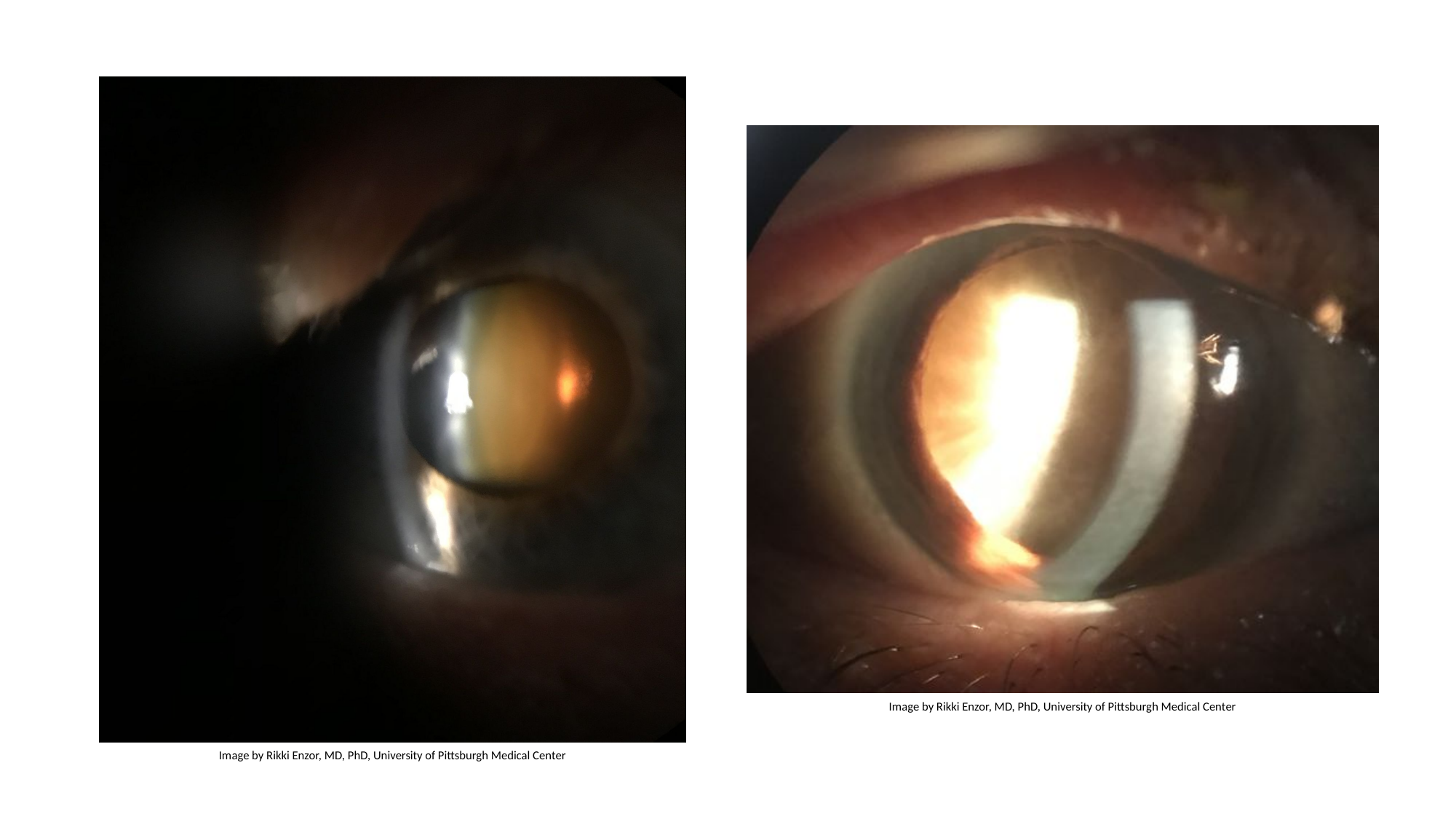

Image by Rikki Enzor, MD, PhD, University of Pittsburgh Medical Center
Image by Rikki Enzor, MD, PhD, University of Pittsburgh Medical Center

## Slide 16
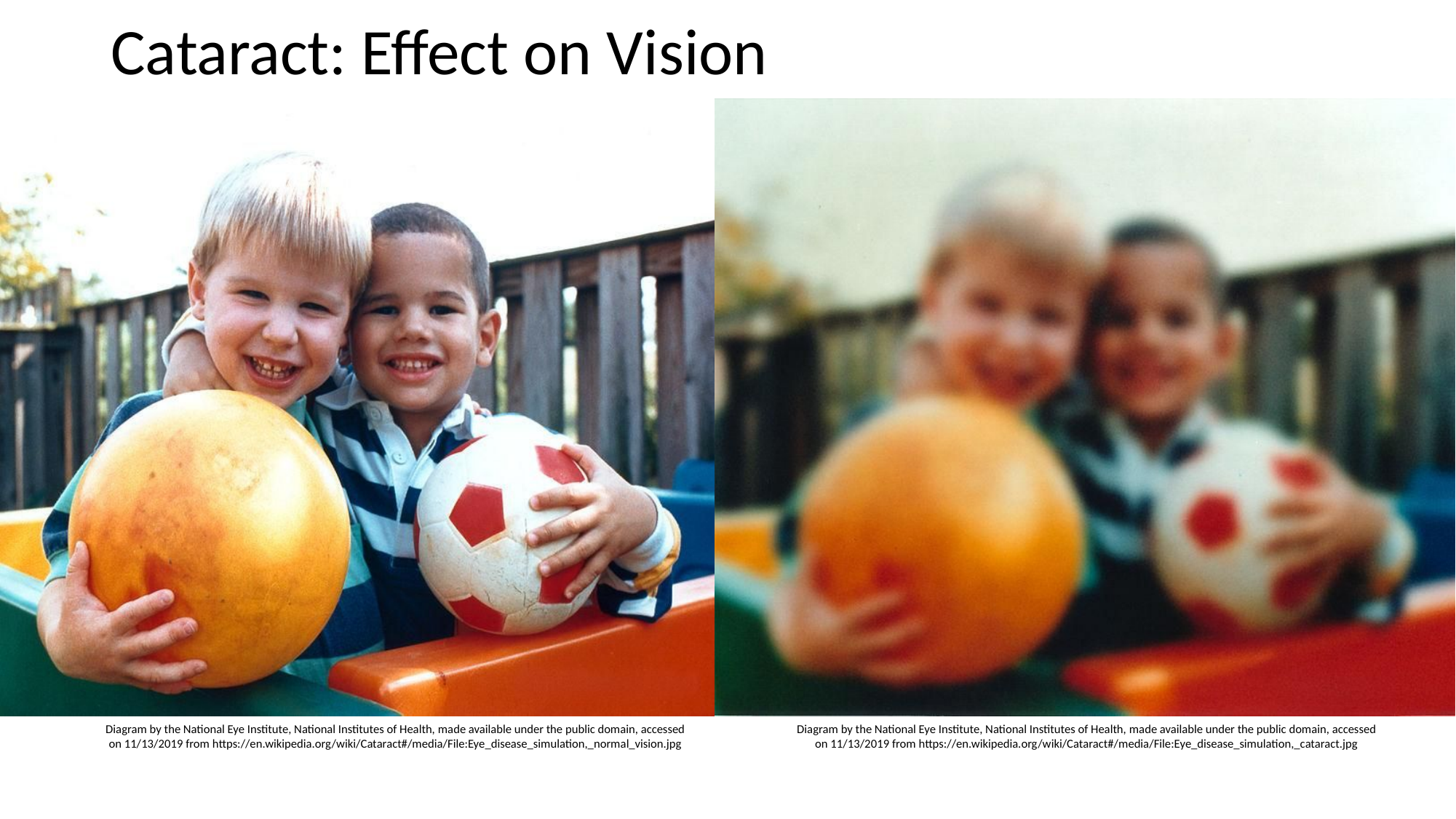

Cataract: Effect on Vision
Diagram by the National Eye Institute, National Institutes of Health, made available under the public domain, accessed on 11/13/2019 from https://en.wikipedia.org/wiki/Cataract#/media/File:Eye_disease_simulation,_normal_vision.jpg
Diagram by the National Eye Institute, National Institutes of Health, made available under the public domain, accessed on 11/13/2019 from https://en.wikipedia.org/wiki/Cataract#/media/File:Eye_disease_simulation,_cataract.jpg

## Slide 17
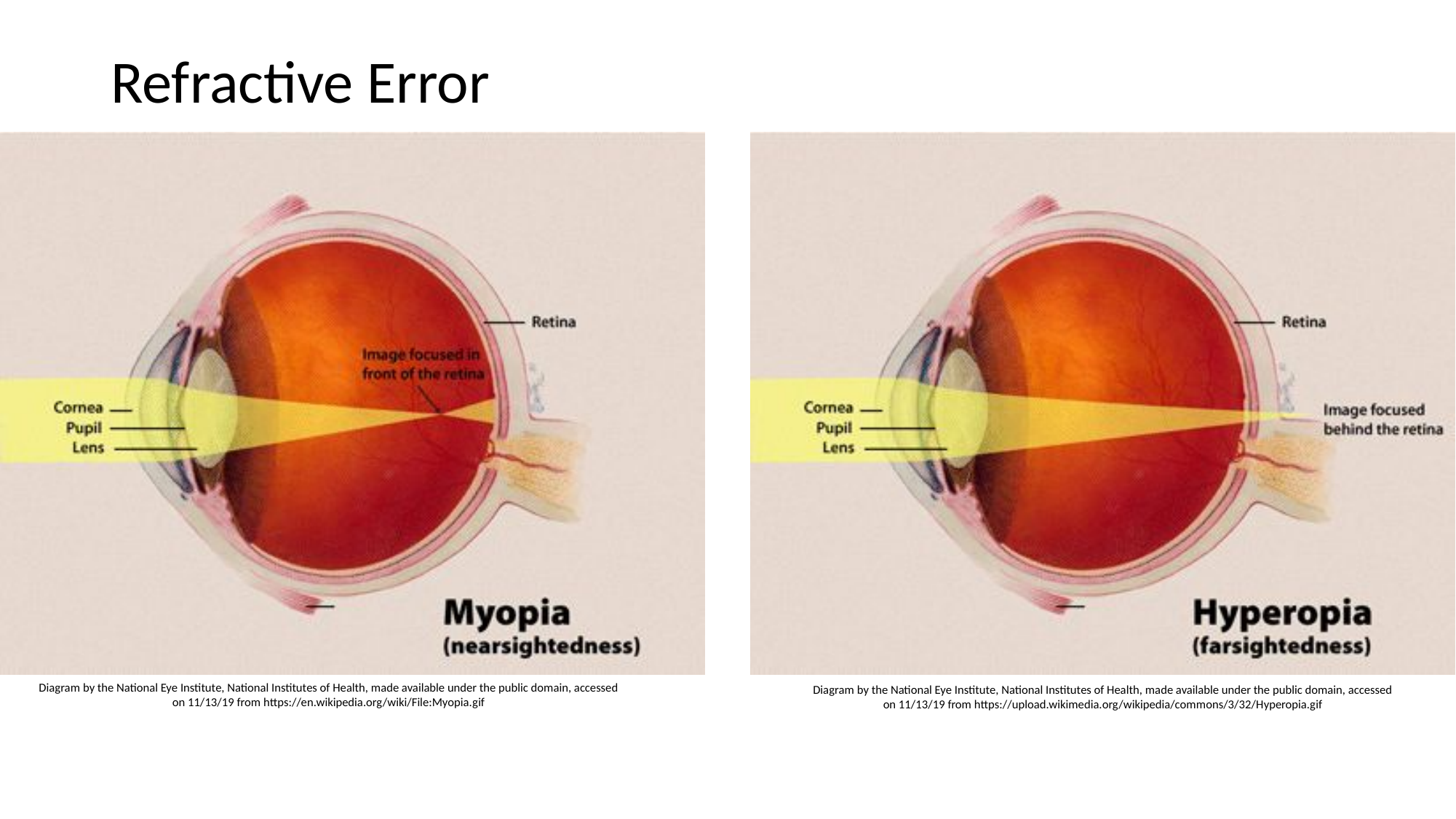

# Refractive Error
Diagram by the National Eye Institute, National Institutes of Health, made available under the public domain, accessed on 11/13/19 from https://en.wikipedia.org/wiki/File:Myopia.gif
Diagram by the National Eye Institute, National Institutes of Health, made available under the public domain, accessed on 11/13/19 from https://upload.wikimedia.org/wikipedia/commons/3/32/Hyperopia.gif

## Slide 18
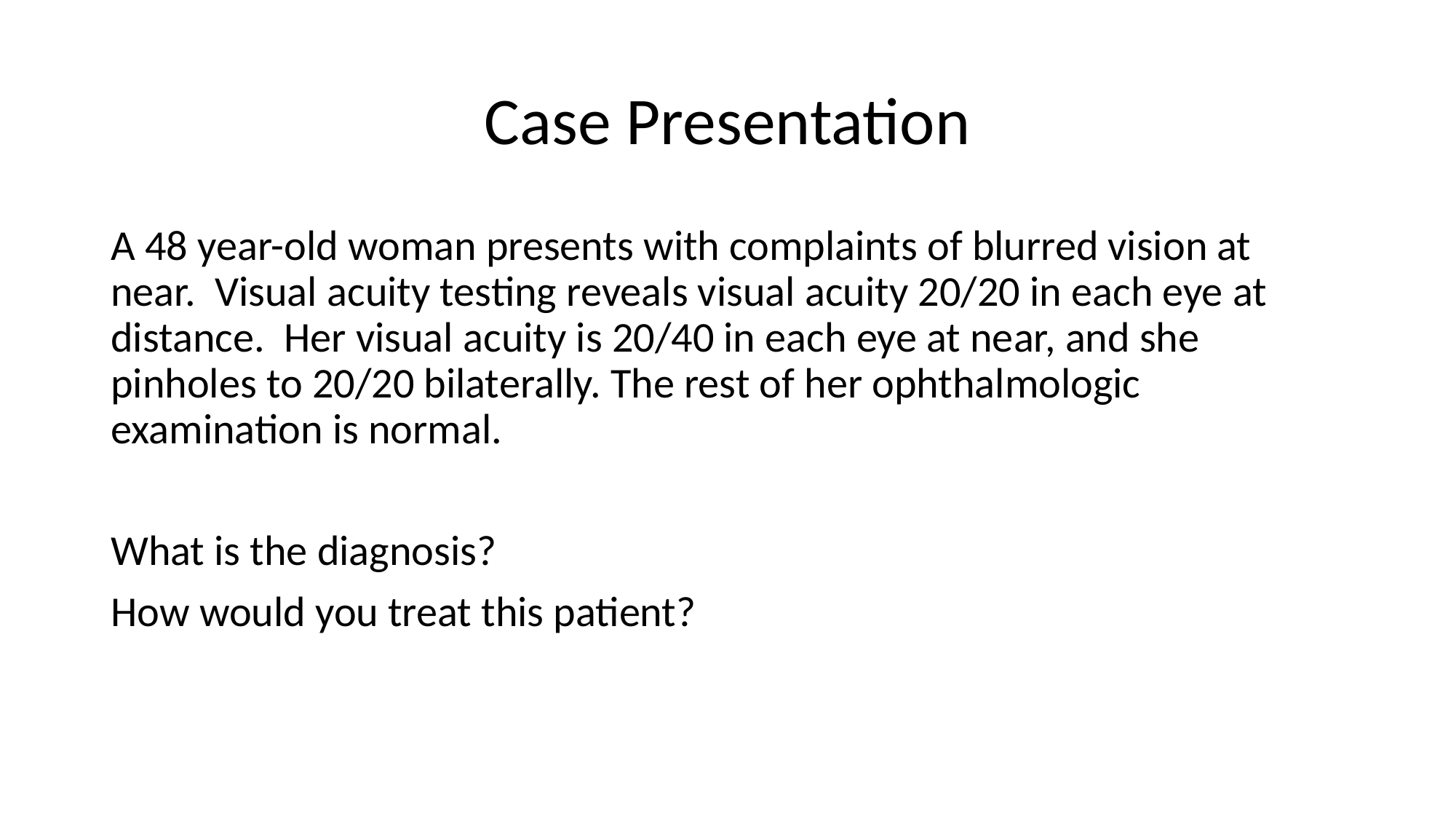

# Case Presentation
A 48 year-old woman presents with complaints of blurred vision at near. Visual acuity testing reveals visual acuity 20/20 in each eye at distance. Her visual acuity is 20/40 in each eye at near, and she pinholes to 20/20 bilaterally. The rest of her ophthalmologic examination is normal.
What is the diagnosis?
How would you treat this patient?

## Slide 19
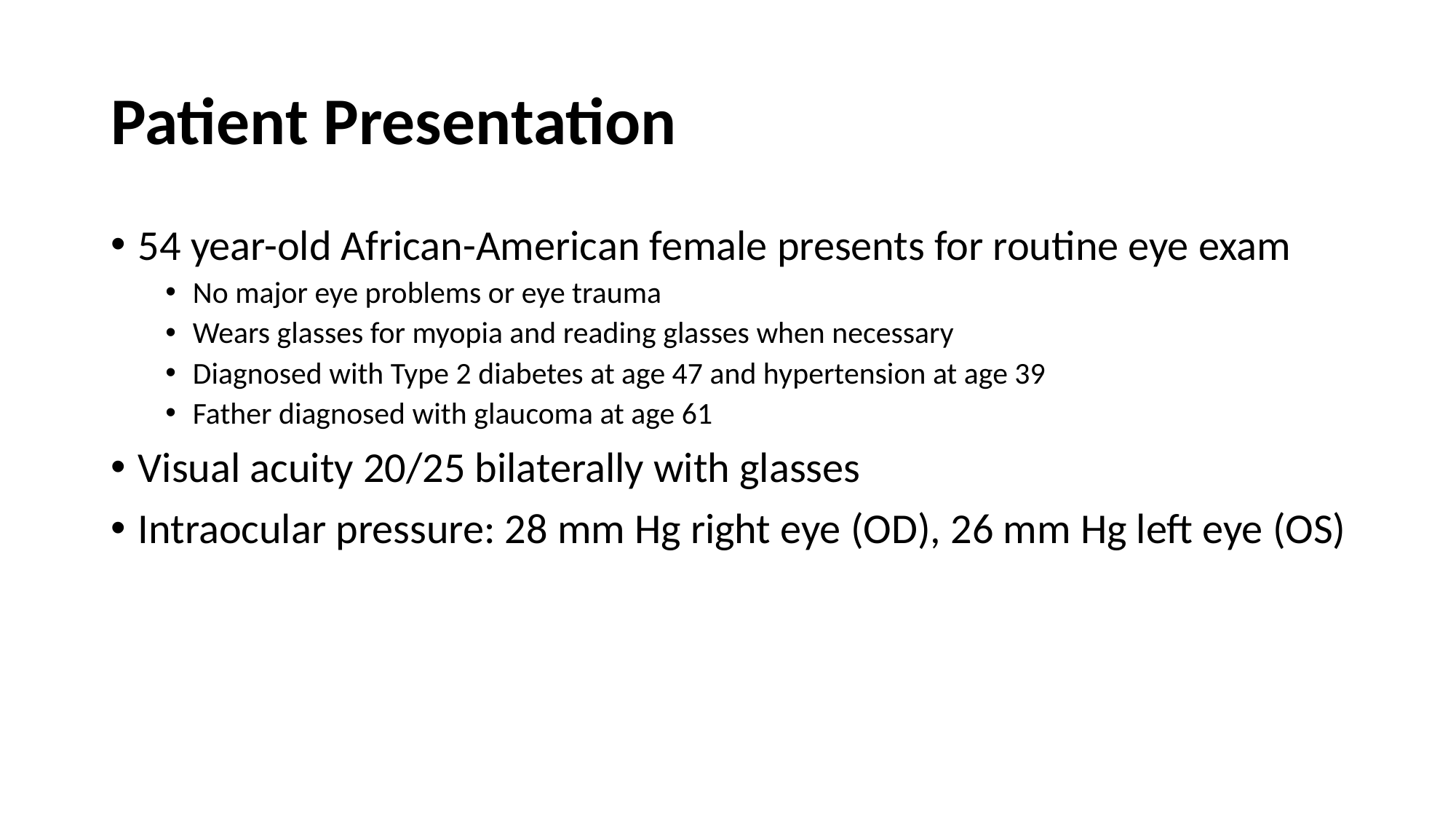

# Patient Presentation
54 year-old African-American female presents for routine eye exam
No major eye problems or eye trauma
Wears glasses for myopia and reading glasses when necessary
Diagnosed with Type 2 diabetes at age 47 and hypertension at age 39
Father diagnosed with glaucoma at age 61
Visual acuity 20/25 bilaterally with glasses
Intraocular pressure: 28 mm Hg right eye (OD), 26 mm Hg left eye (OS)

## Slide 20
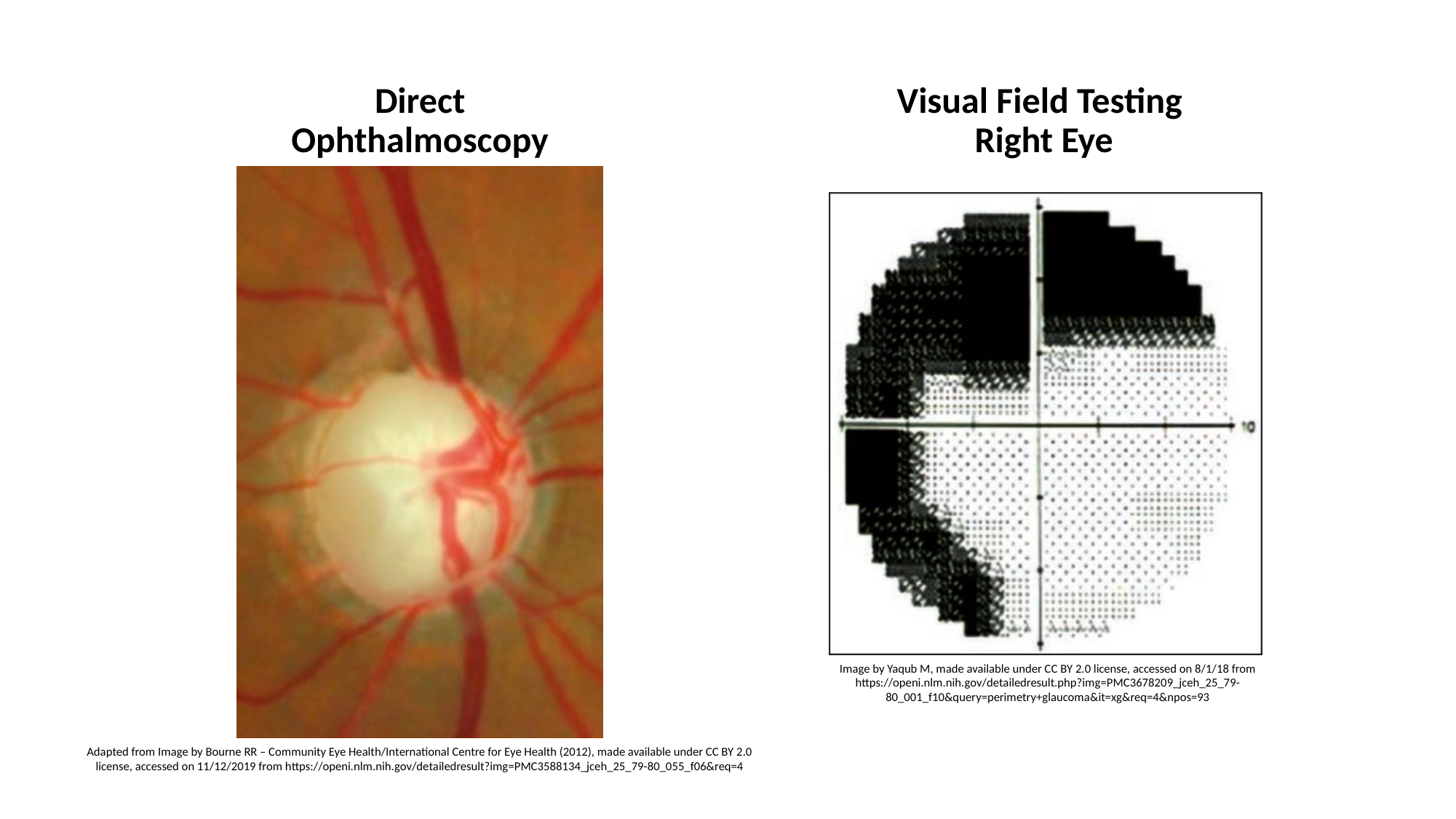

Direct Ophthalmoscopy
Visual Field Testing
Right Eye
Image by Yaqub M, made available under CC BY 2.0 license, accessed on 8/1/18 from https://openi.nlm.nih.gov/detailedresult.php?img=PMC3678209_jceh_25_79-80_001_f10&query=perimetry+glaucoma&it=xg&req=4&npos=93
Adapted from Image by Bourne RR – Community Eye Health/International Centre for Eye Health (2012), made available under CC BY 2.0 license, accessed on 11/12/2019 from https://openi.nlm.nih.gov/detailedresult?img=PMC3588134_jceh_25_79-80_055_f06&req=4

## Slide 21
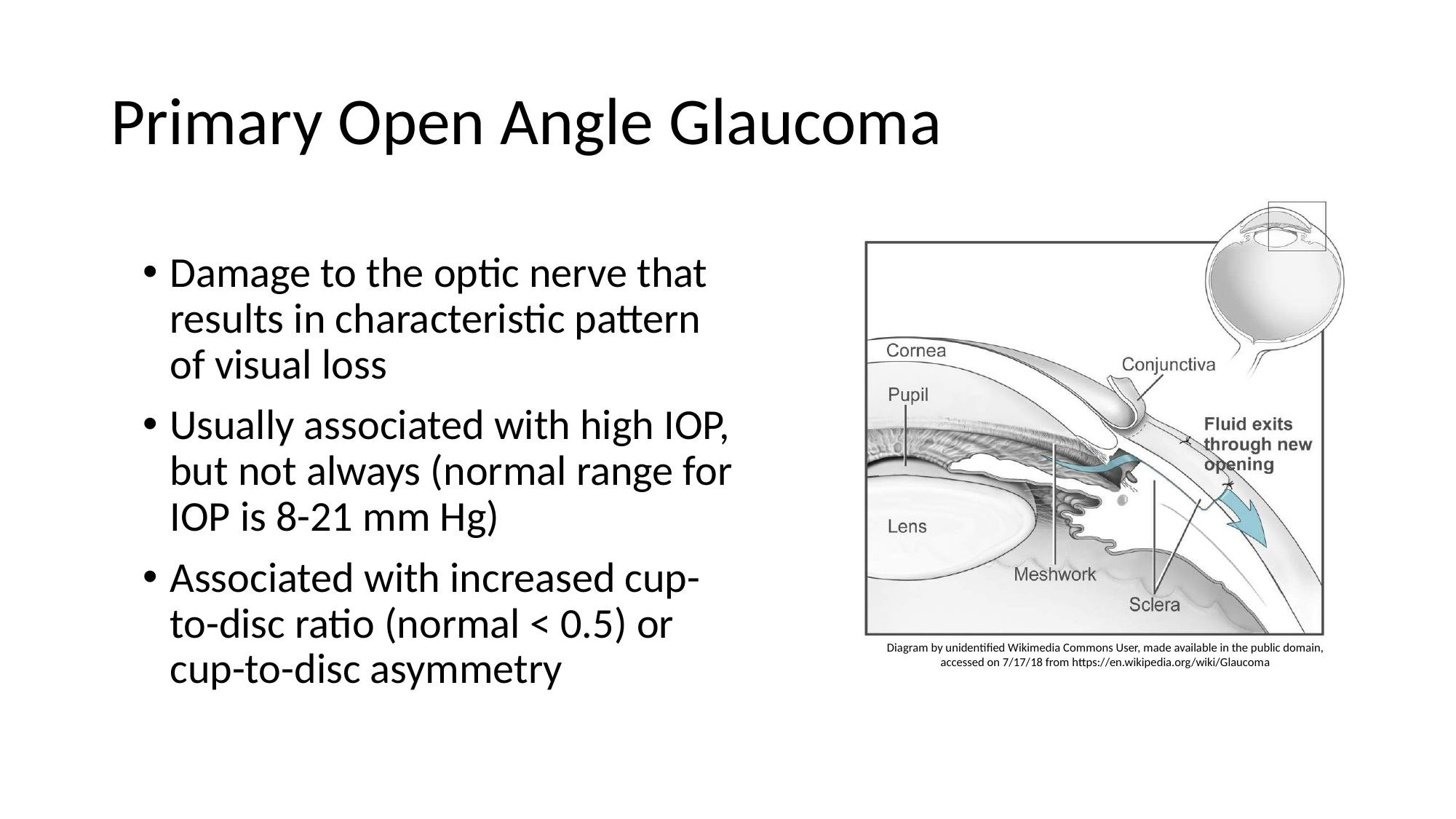

# Primary Open Angle Glaucoma
Damage to the optic nerve that results in characteristic pattern of visual loss
Usually associated with high IOP, but not always (normal range for IOP is 8-21 mm Hg)
Associated with increased cup-to-disc ratio (normal < 0.5) or cup-to-disc asymmetry
Diagram by unidentified Wikimedia Commons User, made available in the public domain, accessed on 7/17/18 from https://en.wikipedia.org/wiki/Glaucoma

## Slide 22
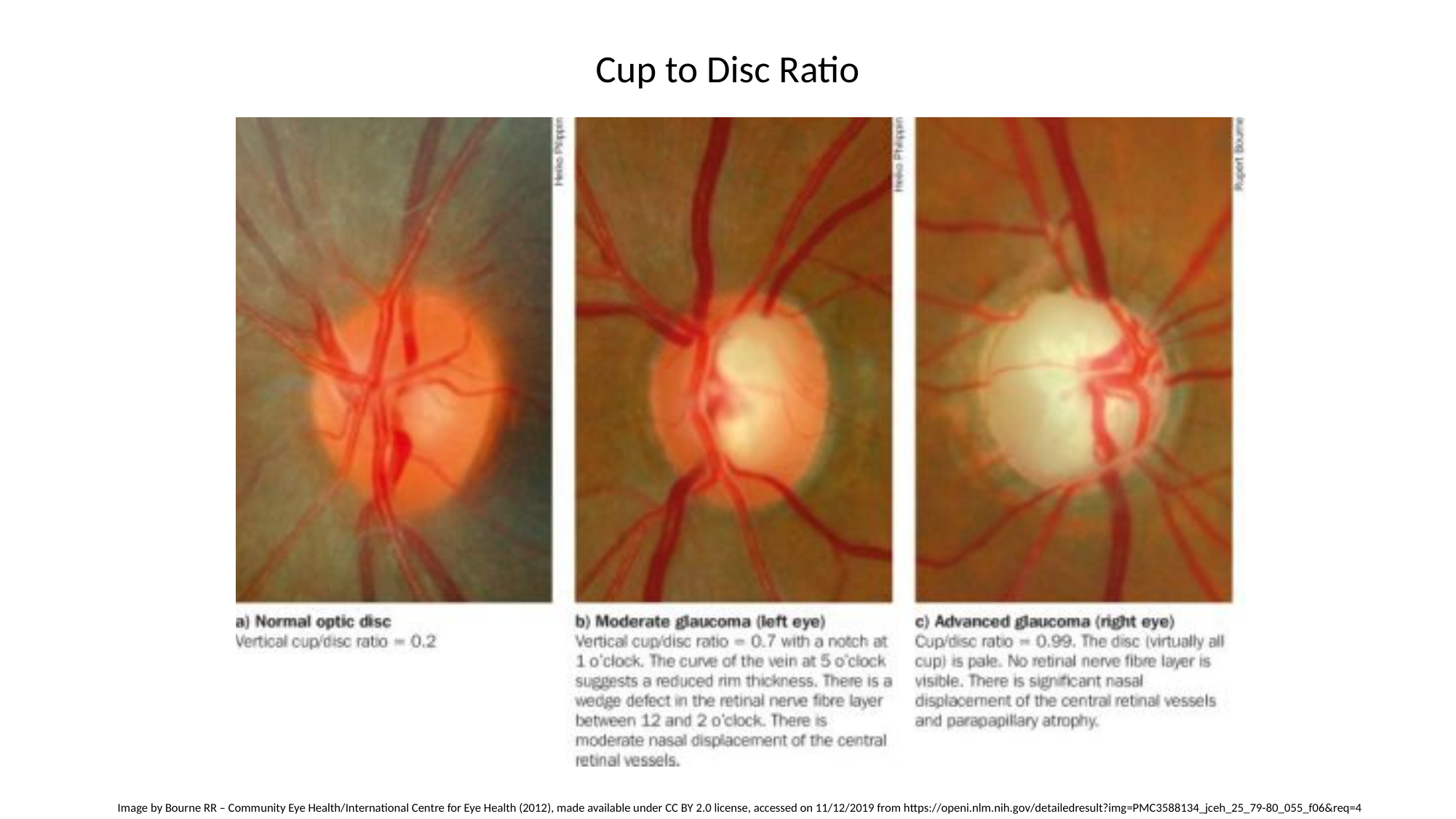

# Cup to Disc Ratio
Image by Bourne RR – Community Eye Health/International Centre for Eye Health (2012), made available under CC BY 2.0 license, accessed on 11/12/2019 from https://openi.nlm.nih.gov/detailedresult?img=PMC3588134_jceh_25_79-80_055_f06&req=4

## Slide 23
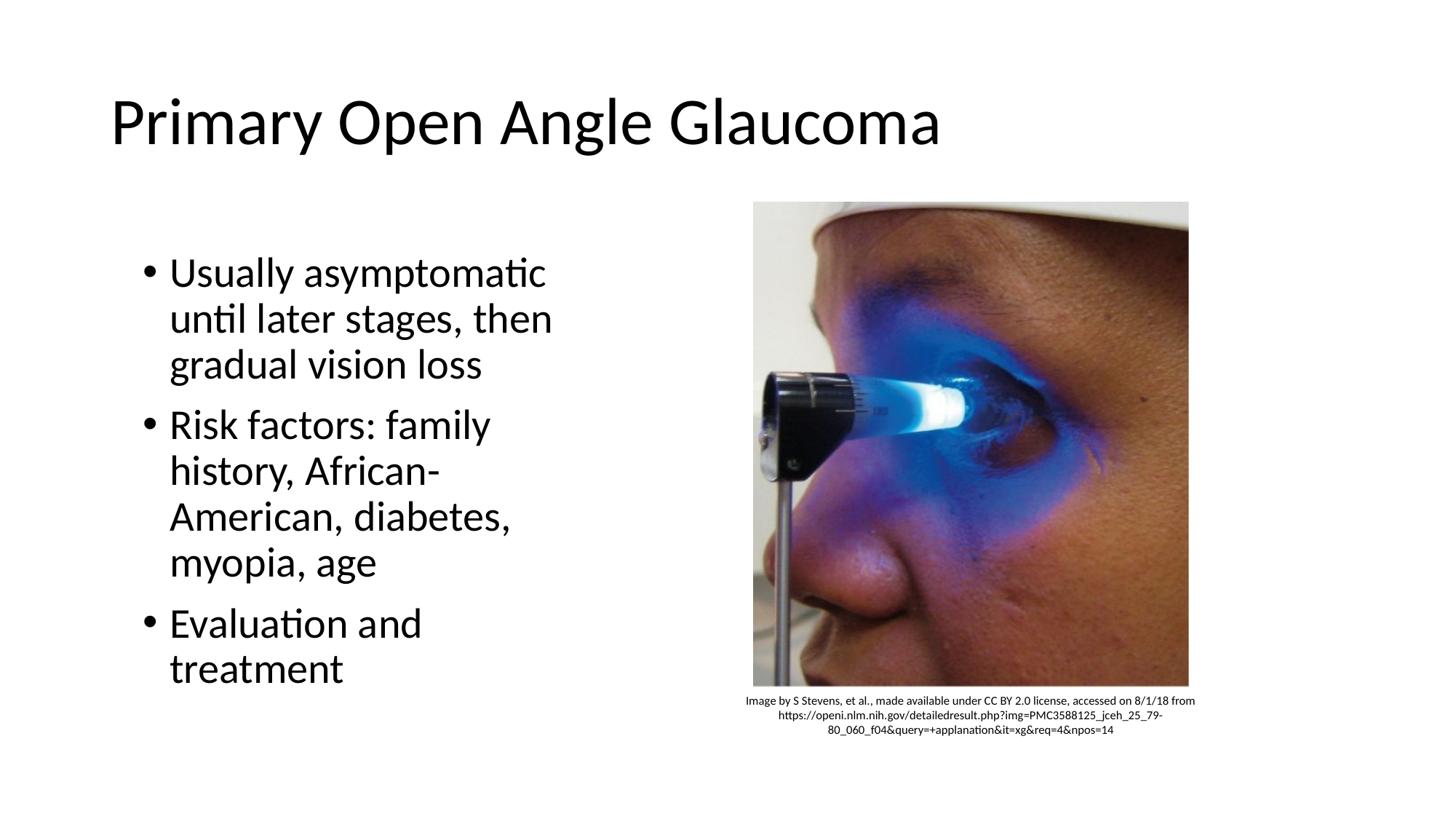

# Primary Open Angle Glaucoma
Image by S Stevens, et al., made available under CC BY 2.0 license, accessed on 8/1/18 from https://openi.nlm.nih.gov/detailedresult.php?img=PMC3588125_jceh_25_79-80_060_f04&query=+applanation&it=xg&req=4&npos=14
Usually asymptomatic until later stages, then gradual vision loss
Risk factors: family history, African-American, diabetes, myopia, age
Evaluation and treatment
