# Supplementary material for: The Ophthalmology Mini-Elective Gives Vision to Preclinical Medical Students
Source: MedEdPORTAL. 2020 Nov 23;16:11024. doi: 10.15766/mep_2374-8265.11024 (PMC7703479; doi:10.15766/mep_2374-8265.11024)
Supplement: Supplementary file 1 — Course Syllabus.docxInstructor Introduction.docxWeekly Course Time Line & Objectives.docxSession 1 - Intro to Ophthalmology.pptxSession 2 - Anterior Segment.pptxSession 3 - Posterior Segment.pptxSession 4 - Eye Emergencies and Trauma.pptxLaboratory Session Guide.pdfPrecourse Survey.docxPre- and Posttest.docxPostcourse Survey.docxPre- and Posttest Answers.docx [file mep_2374-8265.11024-s001.zip › F. Session 3 - Posterior Segment.pptx]

## Slide 1
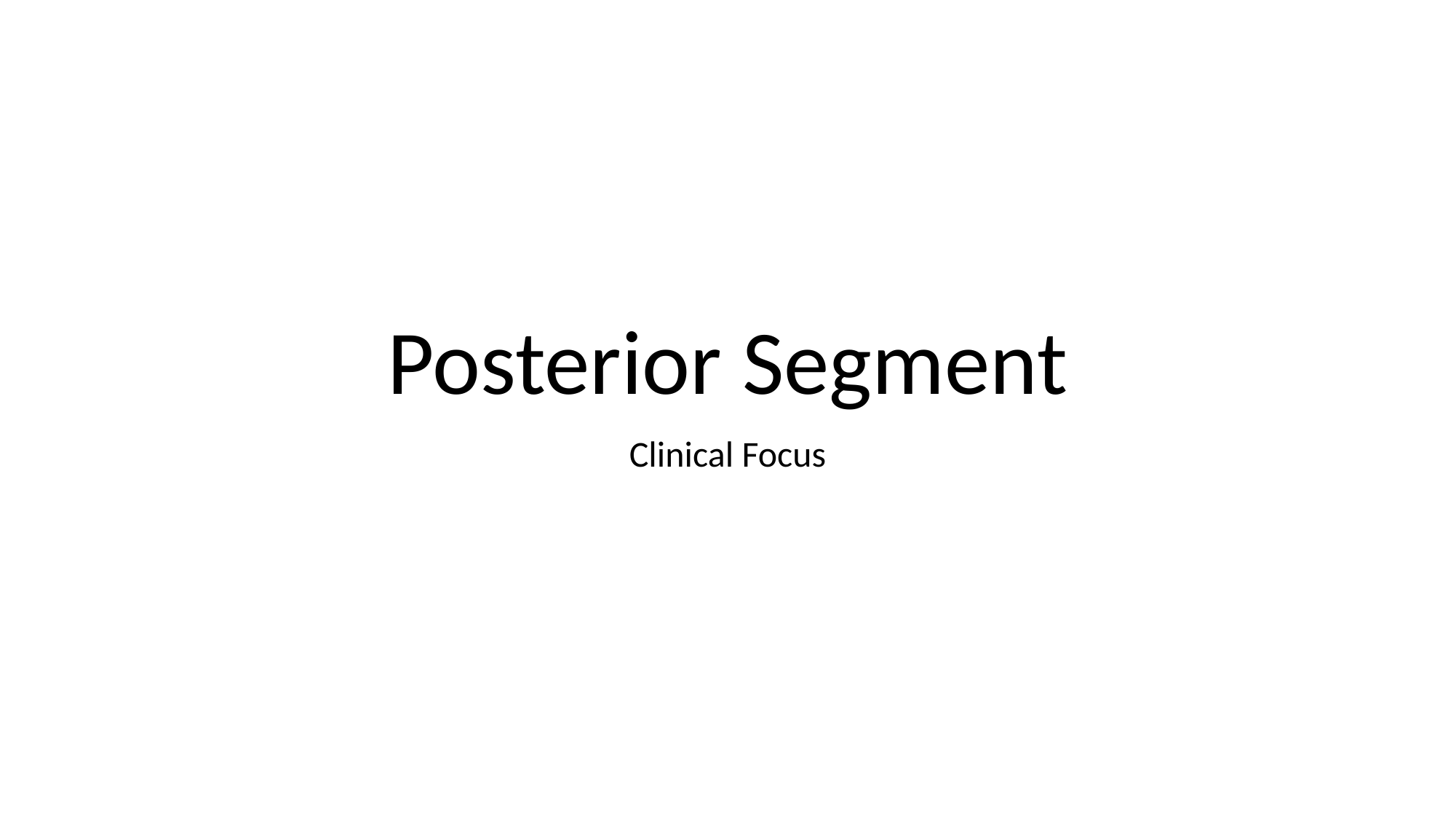

# Posterior Segment
Clinical Focus

## Slide 2
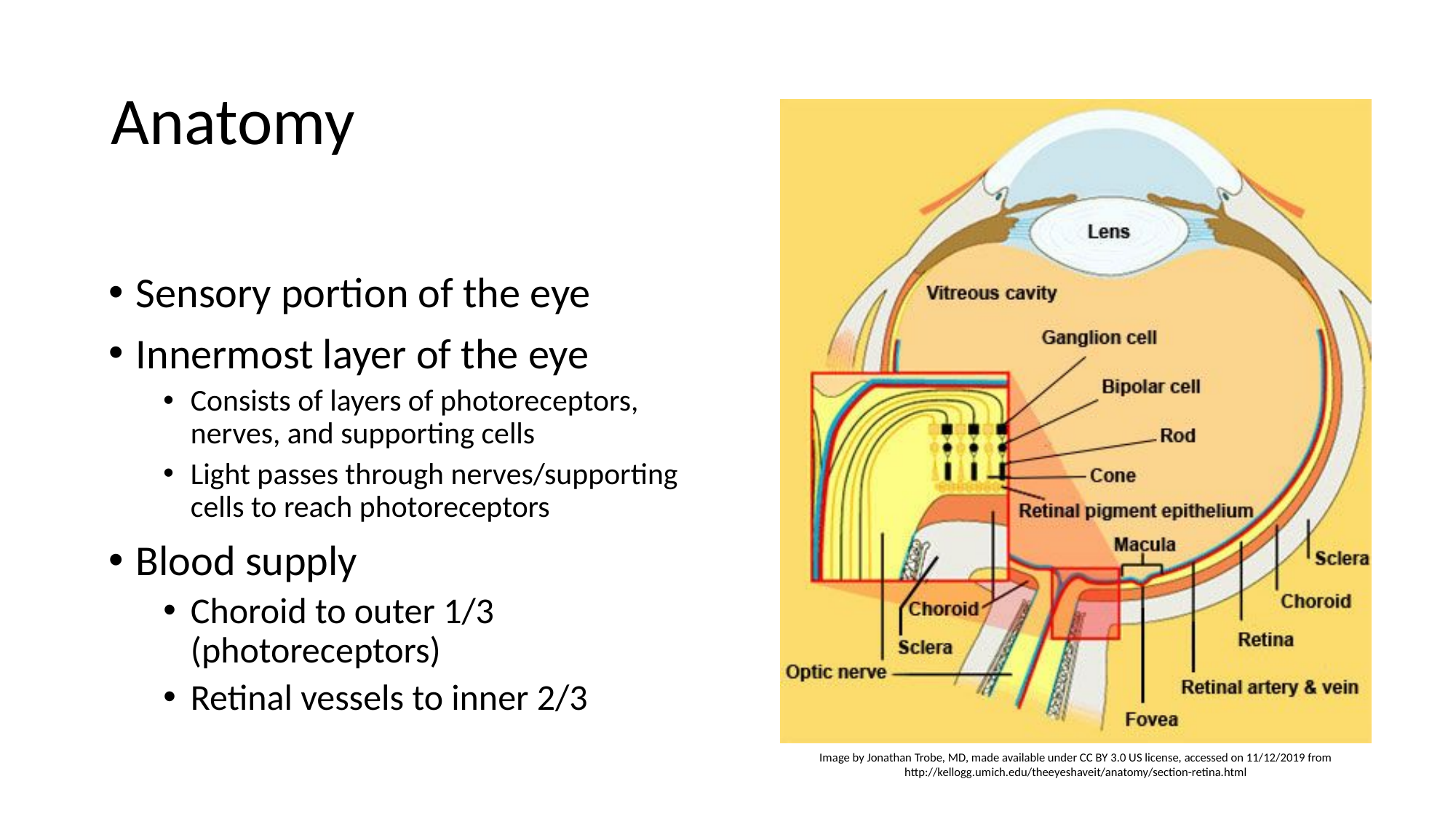

# Anatomy
Sensory portion of the eye
Innermost layer of the eye
Consists of layers of photoreceptors, nerves, and supporting cells
Light passes through nerves/supporting cells to reach photoreceptors
Blood supply
Choroid to outer 1/3 (photoreceptors)
Retinal vessels to inner 2/3
Image by Jonathan Trobe, MD, made available under CC BY 3.0 US license, accessed on 11/12/2019 from http://kellogg.umich.edu/theeyeshaveit/anatomy/section-retina.html

## Slide 3
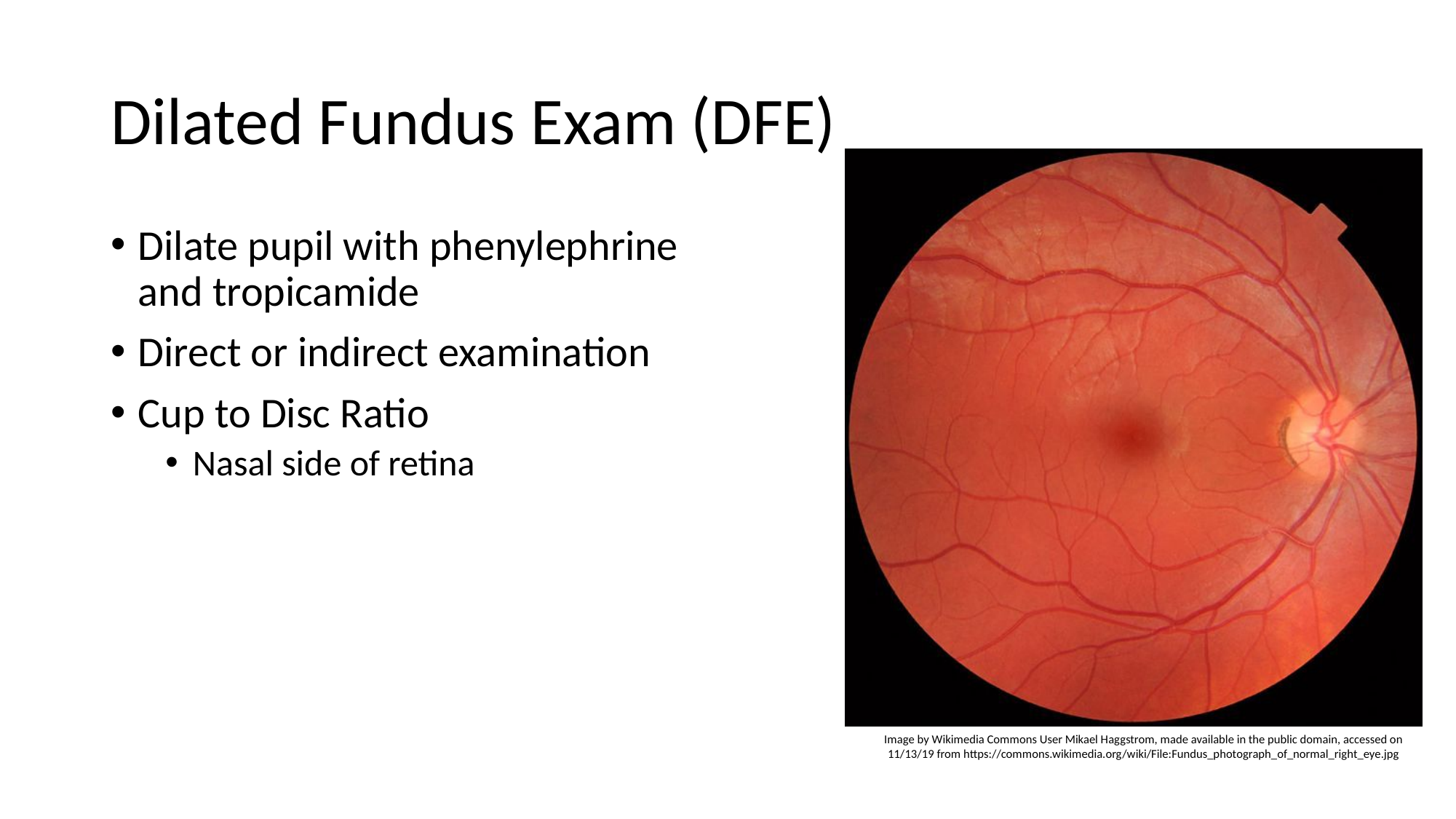

# Dilated Fundus Exam (DFE)
Dilate pupil with phenylephrine and tropicamide
Direct or indirect examination
Cup to Disc Ratio
Nasal side of retina
Image by Wikimedia Commons User Mikael Haggstrom, made available in the public domain, accessed on 11/13/19 from https://commons.wikimedia.org/wiki/File:Fundus_photograph_of_normal_right_eye.jpg

## Slide 4
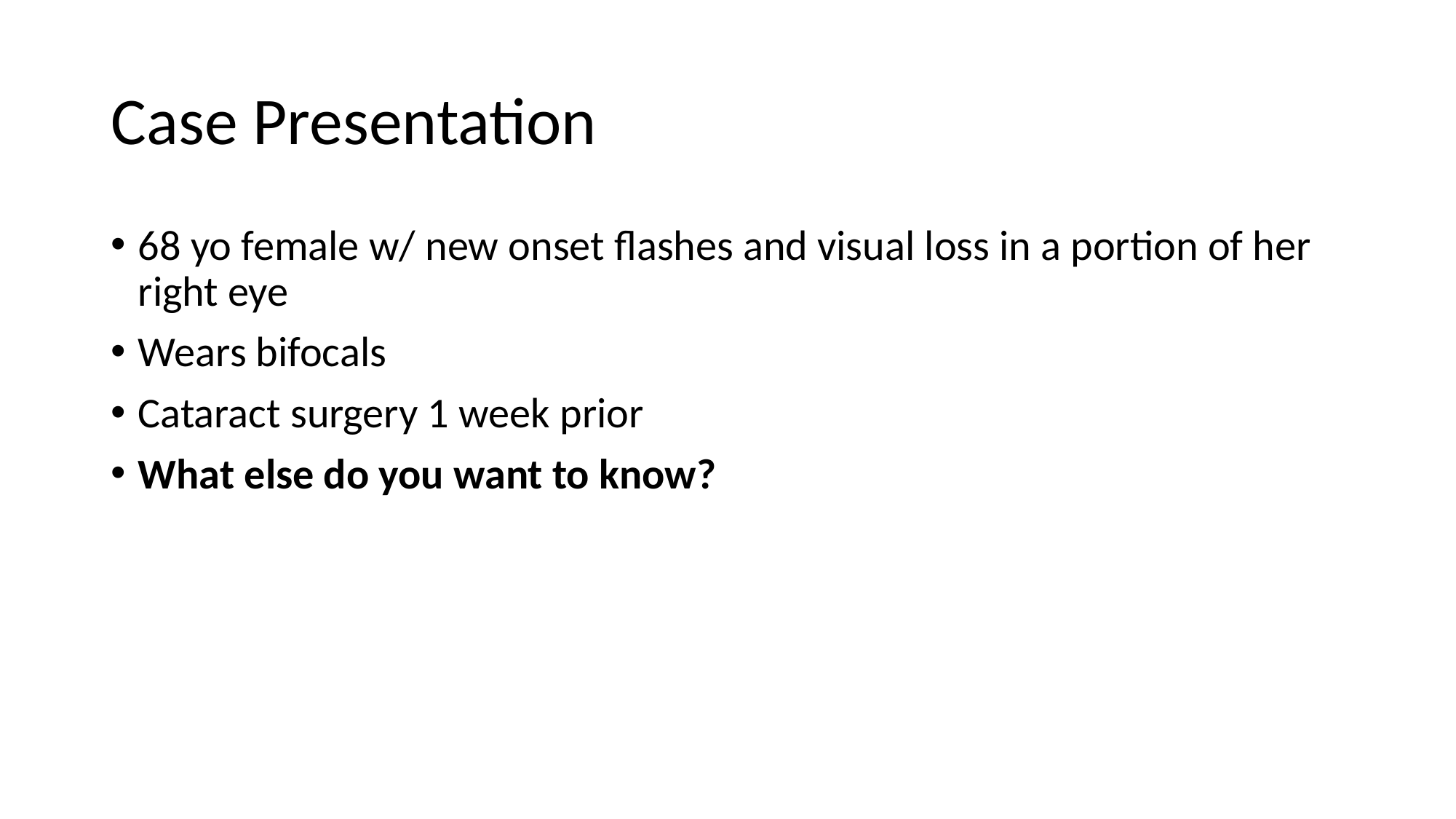

# Case Presentation
68 yo female w/ new onset flashes and visual loss in a portion of her right eye
Wears bifocals
Cataract surgery 1 week prior
What else do you want to know?

## Slide 5
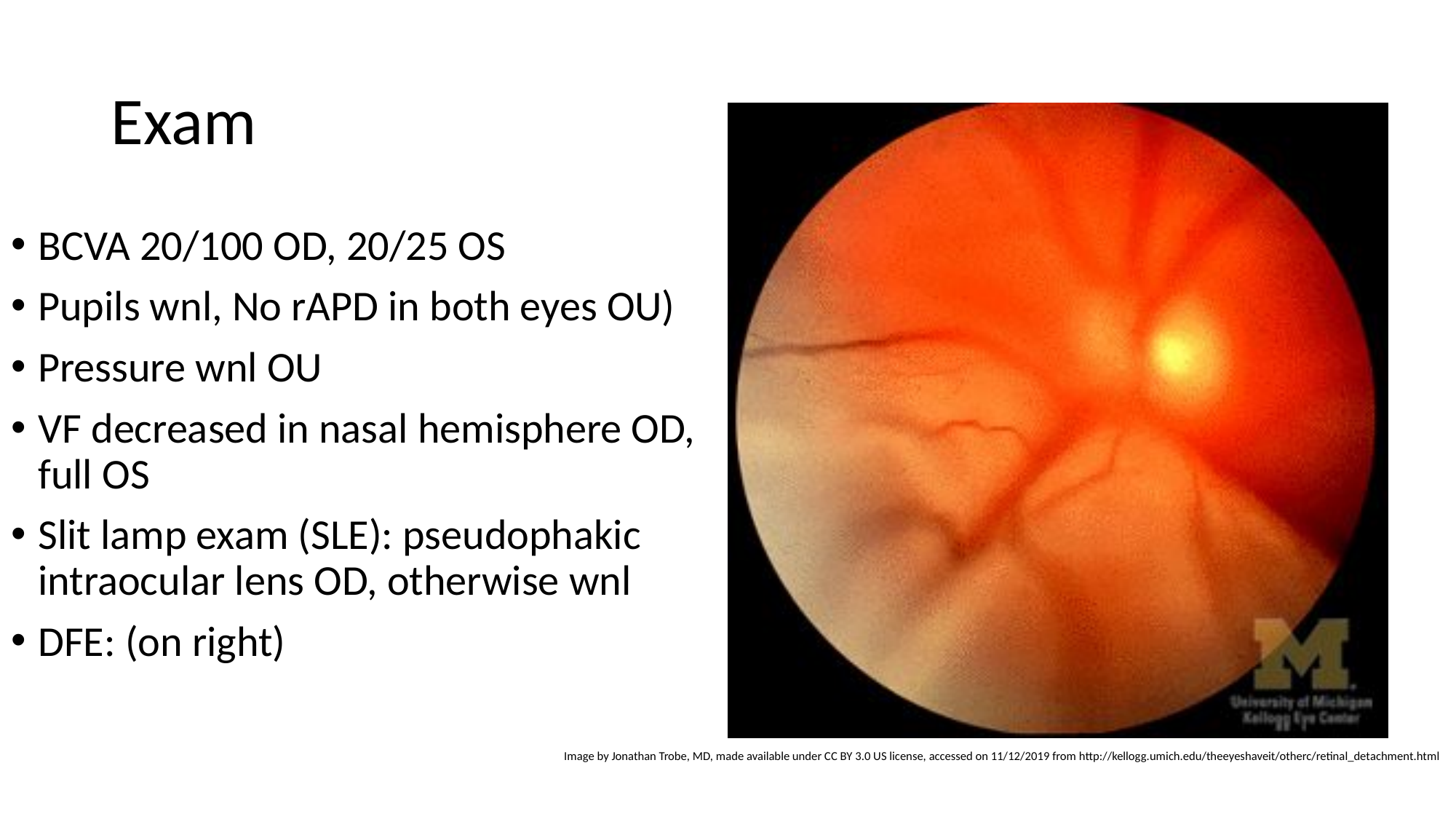

# Exam
BCVA 20/100 OD, 20/25 OS
Pupils wnl, No rAPD in both eyes OU)
Pressure wnl OU
VF decreased in nasal hemisphere OD, full OS
Slit lamp exam (SLE): pseudophakic intraocular lens OD, otherwise wnl
DFE: (on right)
Image by Jonathan Trobe, MD, made available under CC BY 3.0 US license, accessed on 11/12/2019 from http://kellogg.umich.edu/theeyeshaveit/otherc/retinal_detachment.html

## Slide 6
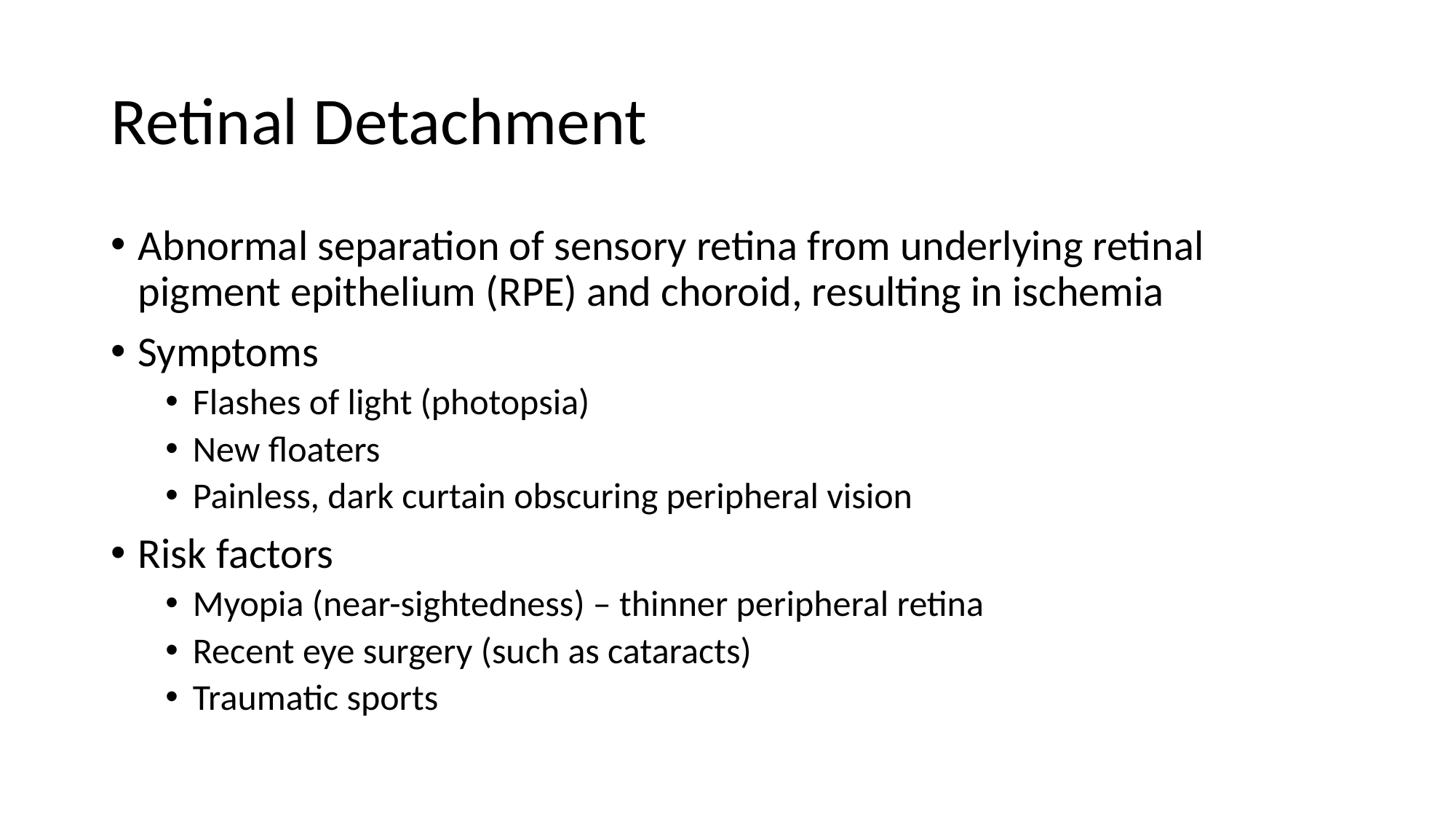

# Retinal Detachment
Abnormal separation of sensory retina from underlying retinal pigment epithelium (RPE) and choroid, resulting in ischemia
Symptoms
Flashes of light (photopsia)
New floaters
Painless, dark curtain obscuring peripheral vision
Risk factors
Myopia (near-sightedness) – thinner peripheral retina
Recent eye surgery (such as cataracts)
Traumatic sports

## Slide 7
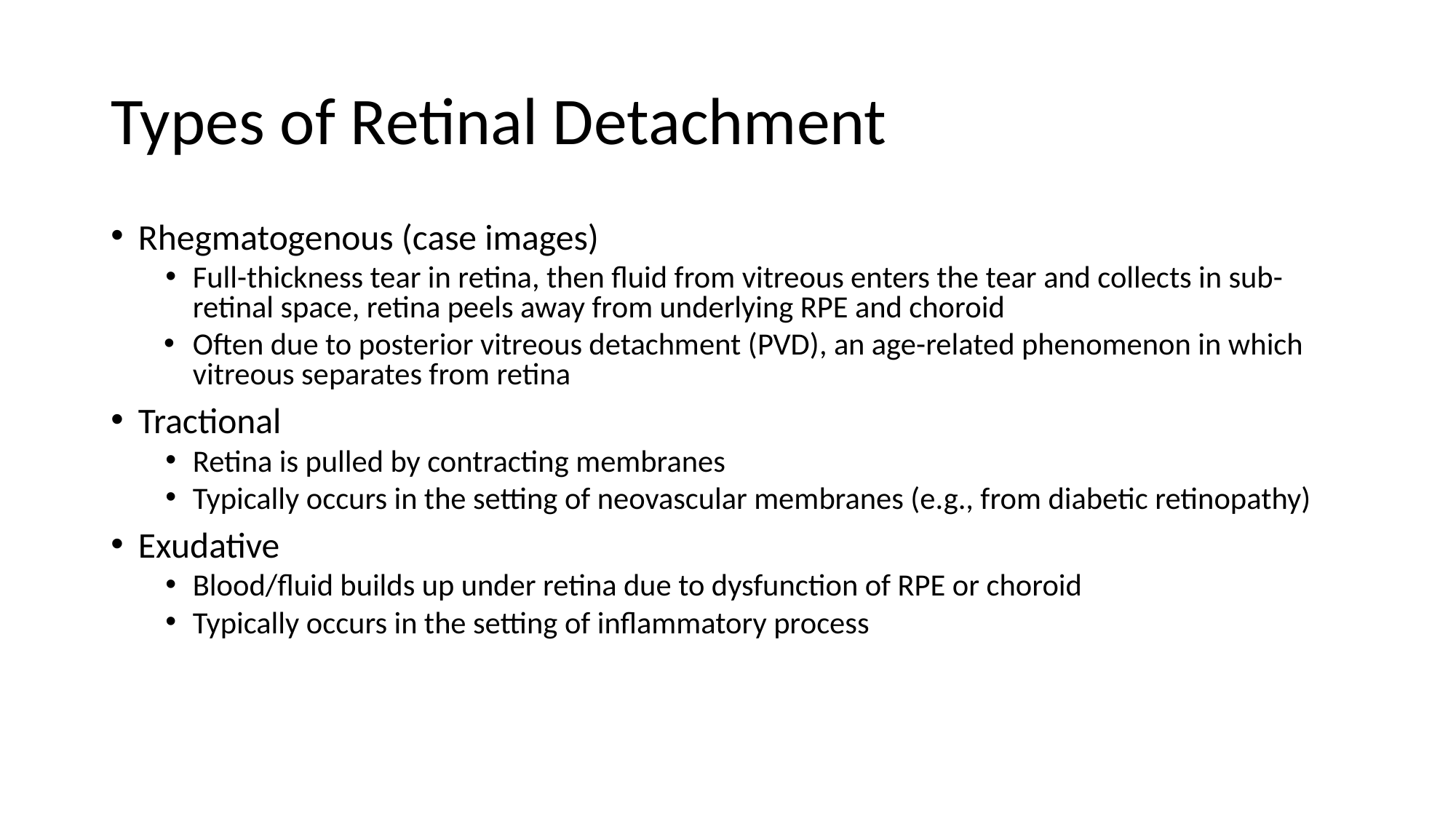

# Types of Retinal Detachment
Rhegmatogenous (case images)
Full-thickness tear in retina, then fluid from vitreous enters the tear and collects in sub-retinal space, retina peels away from underlying RPE and choroid
Often due to posterior vitreous detachment (PVD), an age-related phenomenon in which vitreous separates from retina
Tractional
Retina is pulled by contracting membranes
Typically occurs in the setting of neovascular membranes (e.g., from diabetic retinopathy)
Exudative
Blood/fluid builds up under retina due to dysfunction of RPE or choroid
Typically occurs in the setting of inflammatory process

## Slide 8
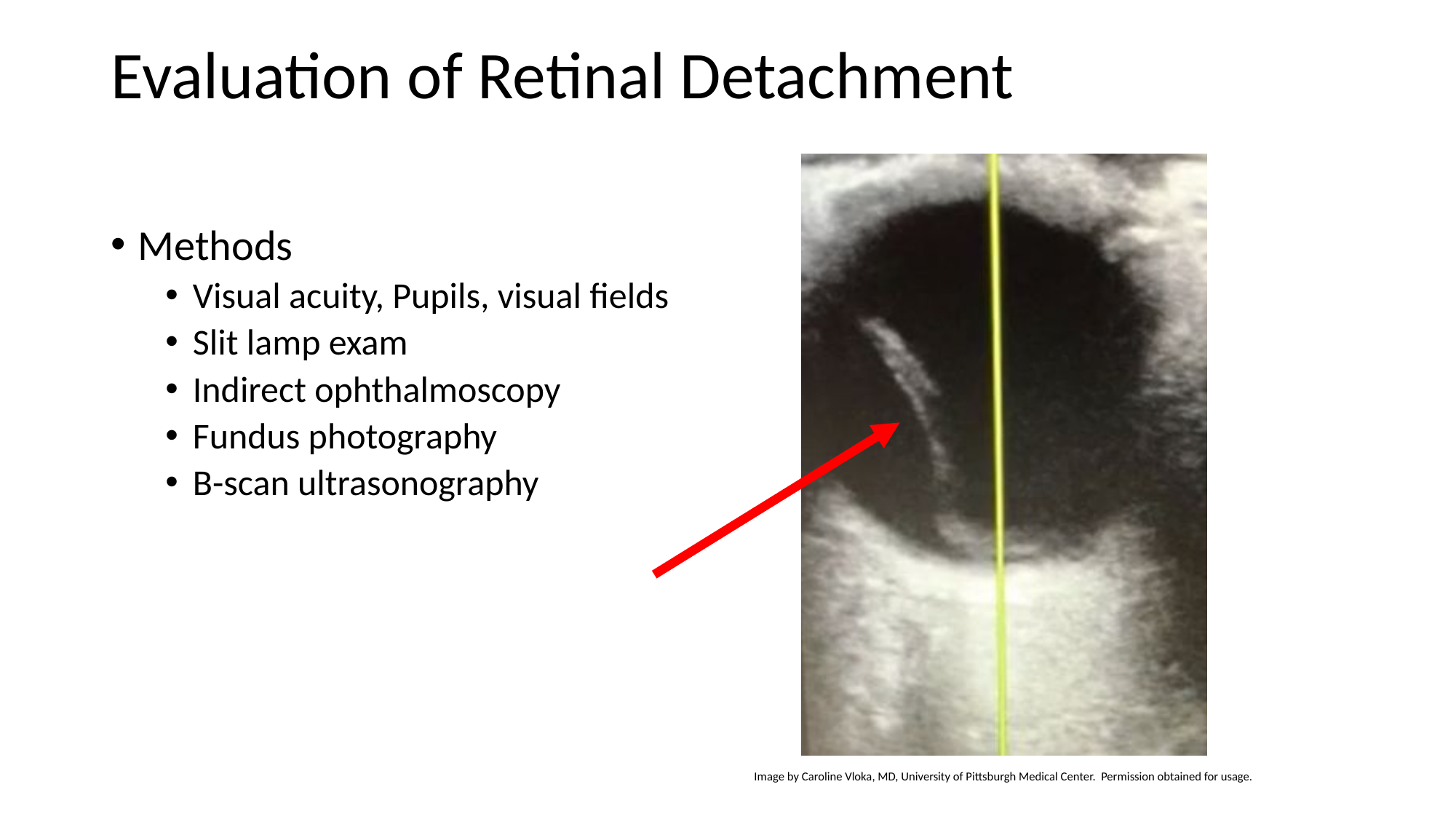

# Evaluation of Retinal Detachment
Methods
Visual acuity, Pupils, visual fields
Slit lamp exam
Indirect ophthalmoscopy
Fundus photography
B-scan ultrasonography
Image by Caroline Vloka, MD, University of Pittsburgh Medical Center. Permission obtained for usage.

## Slide 9
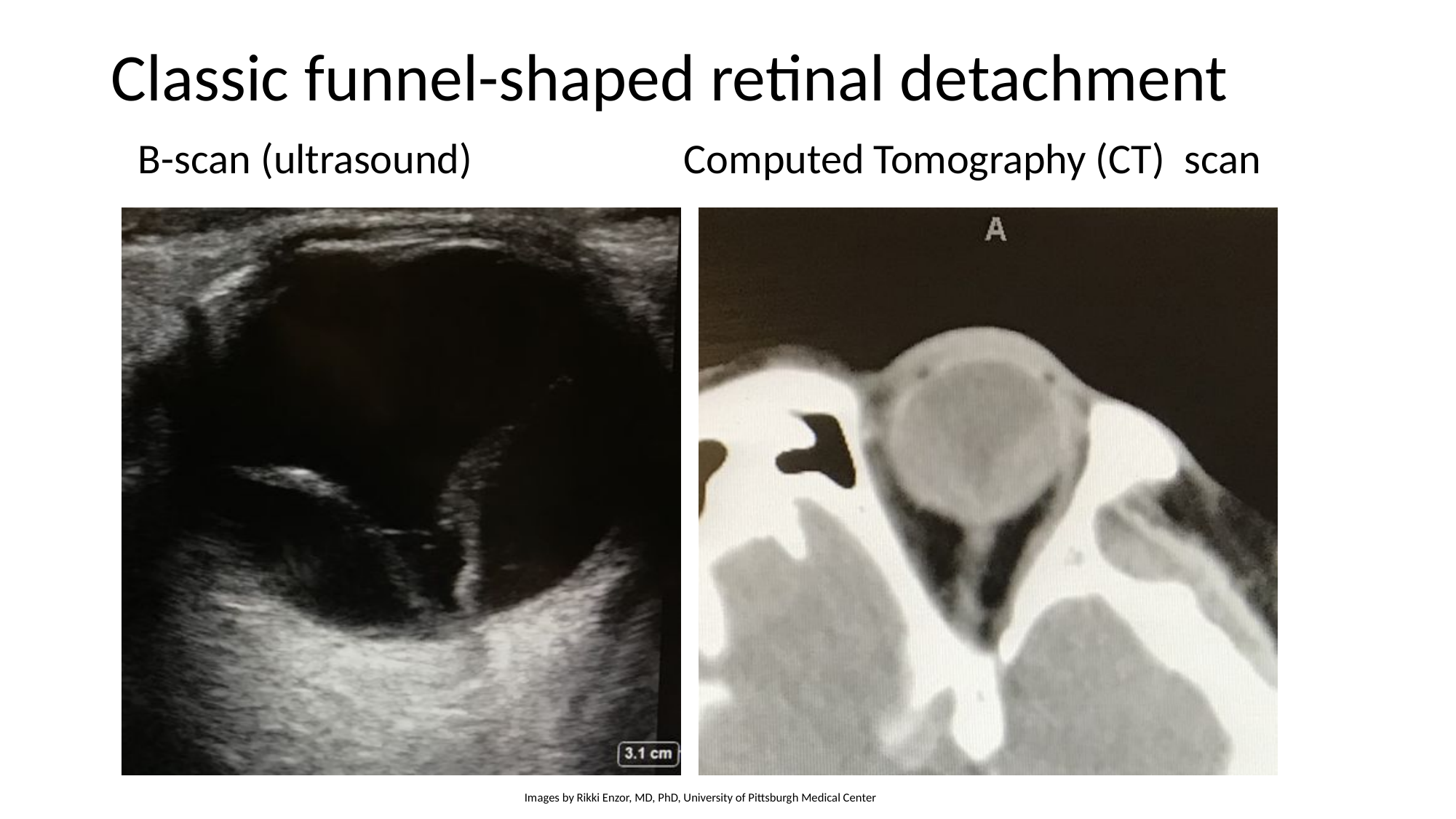

# Classic funnel-shaped retinal detachment
B-scan (ultrasound)		Computed Tomography (CT) scan
Images by Rikki Enzor, MD, PhD, University of Pittsburgh Medical Center

## Slide 10
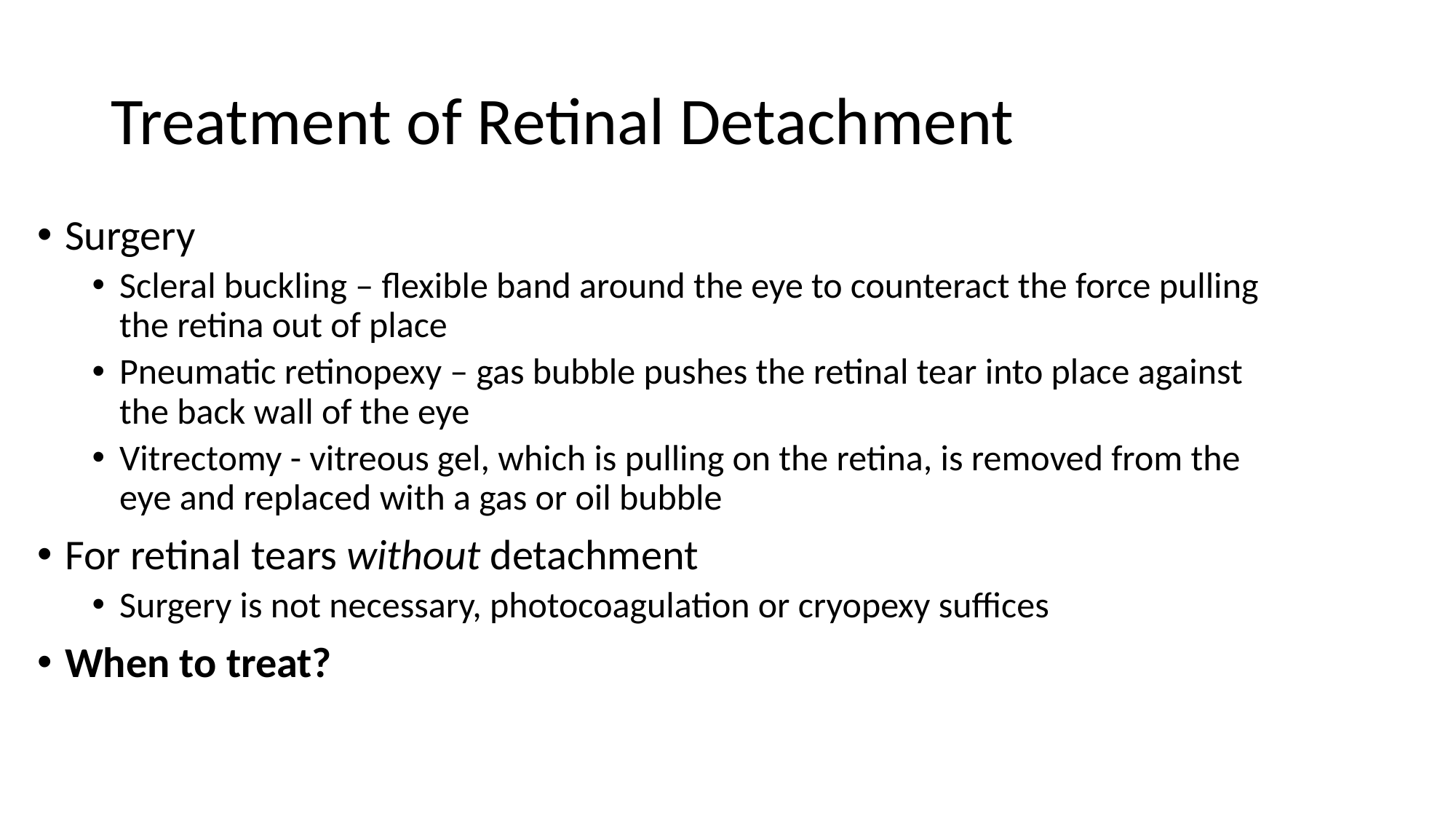

# Treatment of Retinal Detachment
Surgery
Scleral buckling – flexible band around the eye to counteract the force pulling the retina out of place
Pneumatic retinopexy – gas bubble pushes the retinal tear into place against the back wall of the eye
Vitrectomy - vitreous gel, which is pulling on the retina, is removed from the eye and replaced with a gas or oil bubble
For retinal tears without detachment
Surgery is not necessary, photocoagulation or cryopexy suffices
When to treat?

## Slide 11
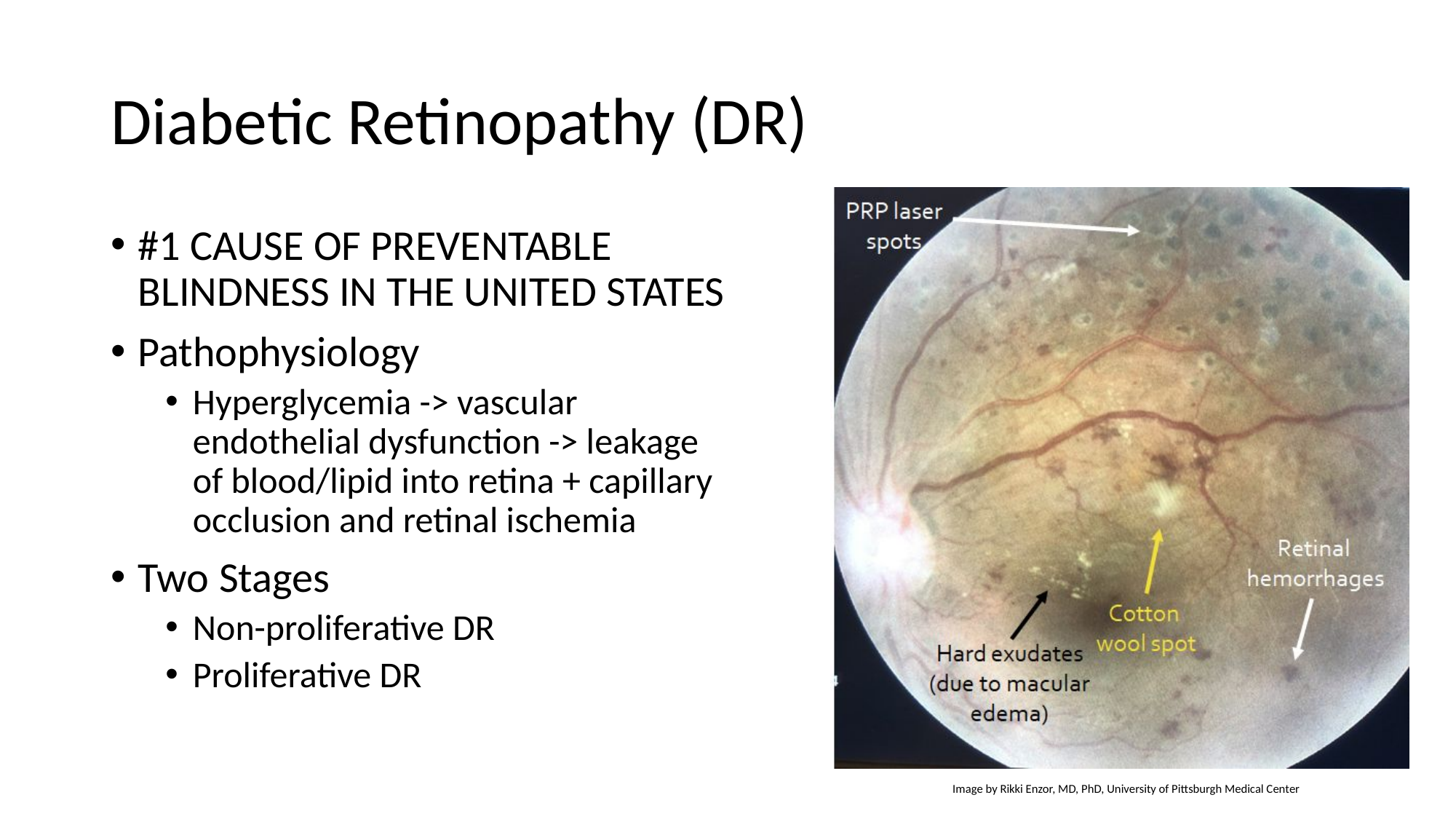

# Diabetic Retinopathy (DR)
#1 CAUSE OF PREVENTABLE BLINDNESS IN THE UNITED STATES
Pathophysiology
Hyperglycemia -> vascular endothelial dysfunction -> leakage of blood/lipid into retina + capillary occlusion and retinal ischemia
Two Stages
Non-proliferative DR
Proliferative DR
Image by Rikki Enzor, MD, PhD, University of Pittsburgh Medical Center

## Slide 12
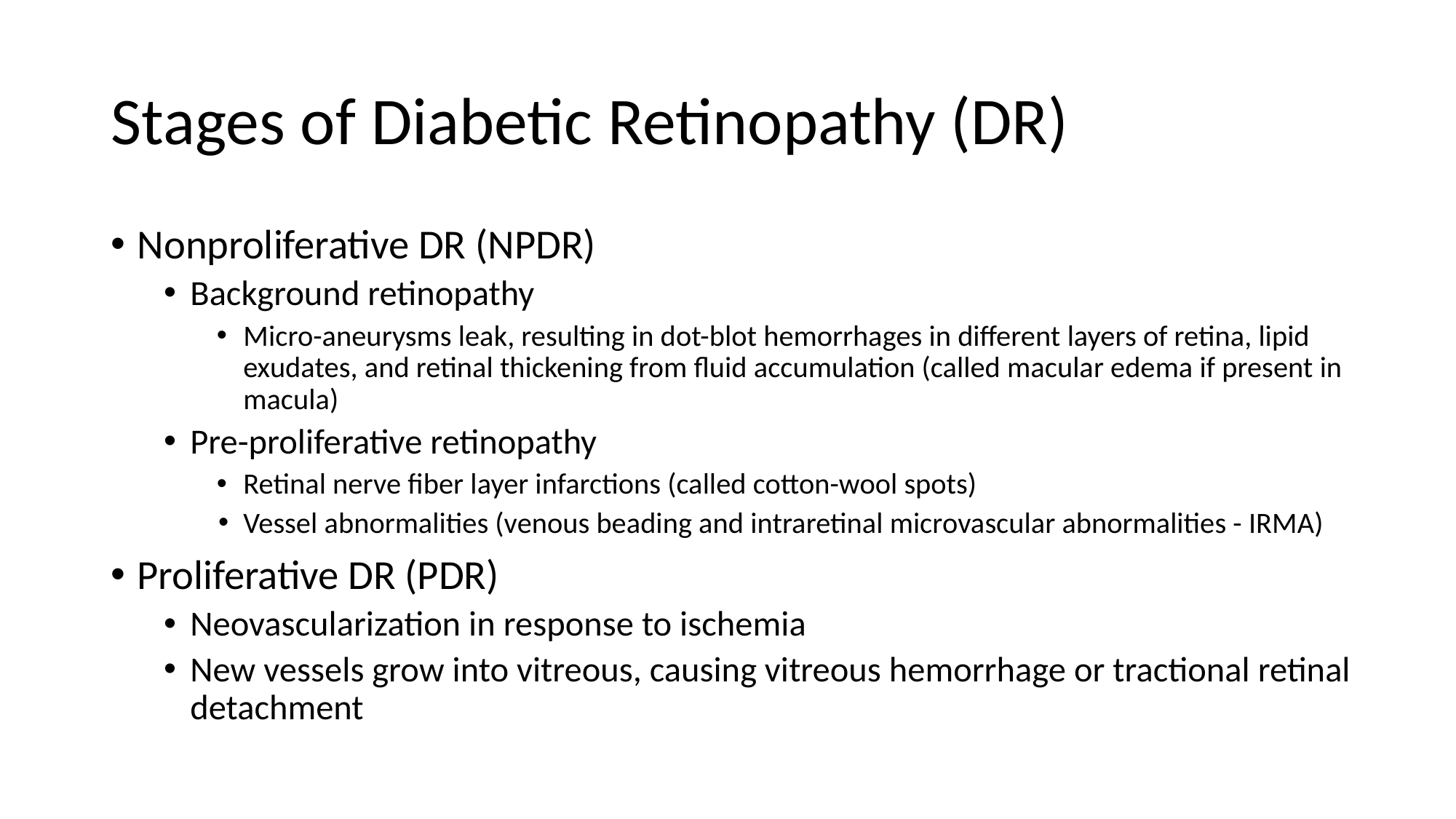

# Stages of Diabetic Retinopathy (DR)
Nonproliferative DR (NPDR)
Background retinopathy
Micro-aneurysms leak, resulting in dot-blot hemorrhages in different layers of retina, lipid exudates, and retinal thickening from fluid accumulation (called macular edema if present in macula)
Pre-proliferative retinopathy
Retinal nerve fiber layer infarctions (called cotton-wool spots)
Vessel abnormalities (venous beading and intraretinal microvascular abnormalities - IRMA)
Proliferative DR (PDR)
Neovascularization in response to ischemia
New vessels grow into vitreous, causing vitreous hemorrhage or tractional retinal detachment

## Slide 13
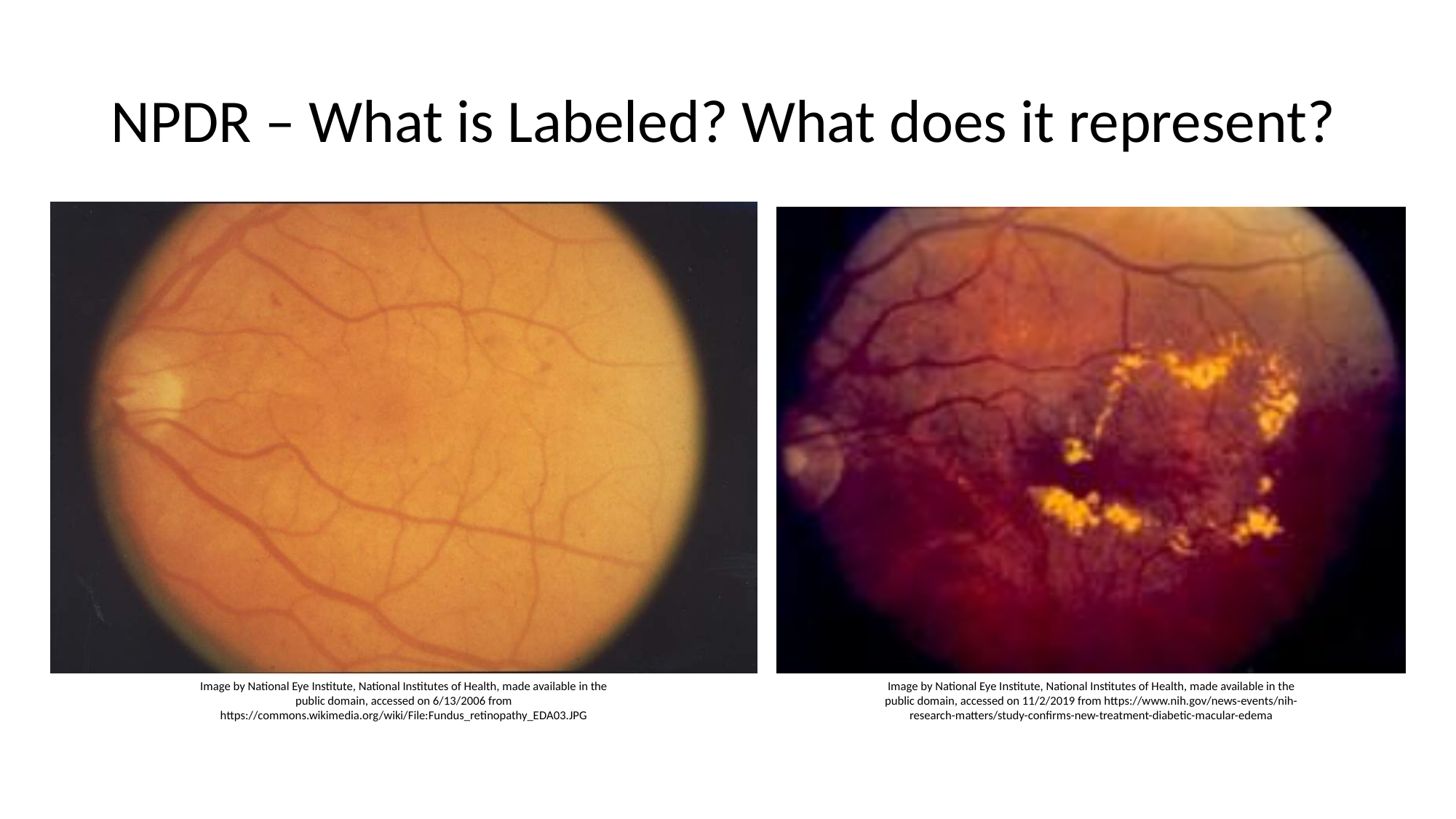

# NPDR – What is Labeled? What does it represent?
Image by National Eye Institute, National Institutes of Health, made available in the public domain, accessed on 11/2/2019 from https://www.nih.gov/news-events/nih-research-matters/study-confirms-new-treatment-diabetic-macular-edema
Image by National Eye Institute, National Institutes of Health, made available in the public domain, accessed on 6/13/2006 from https://commons.wikimedia.org/wiki/File:Fundus_retinopathy_EDA03.JPG

## Slide 14
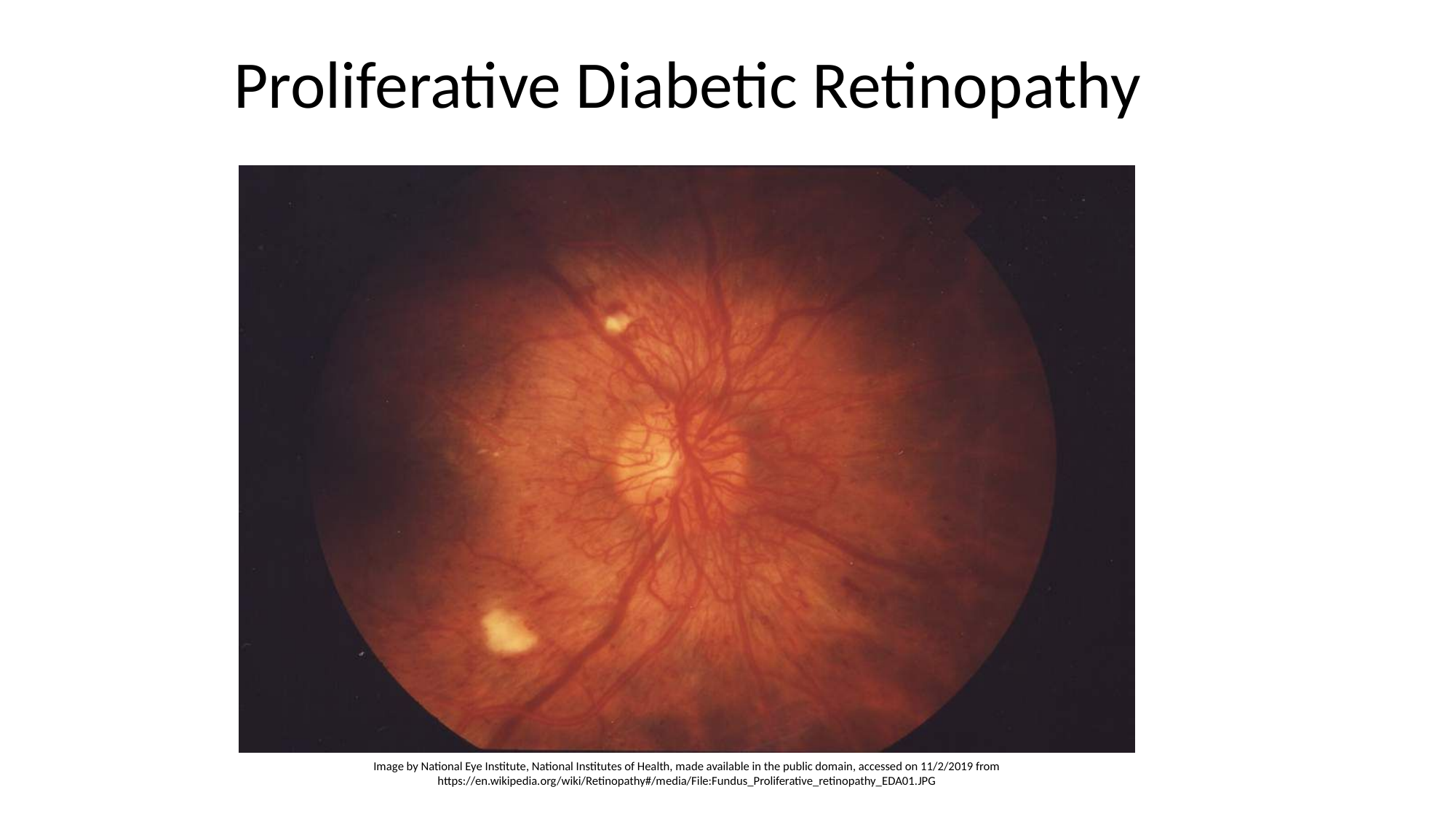

# Proliferative Diabetic Retinopathy
Image by National Eye Institute, National Institutes of Health, made available in the public domain, accessed on 11/2/2019 from https://en.wikipedia.org/wiki/Retinopathy#/media/File:Fundus_Proliferative_retinopathy_EDA01.JPG

## Slide 15
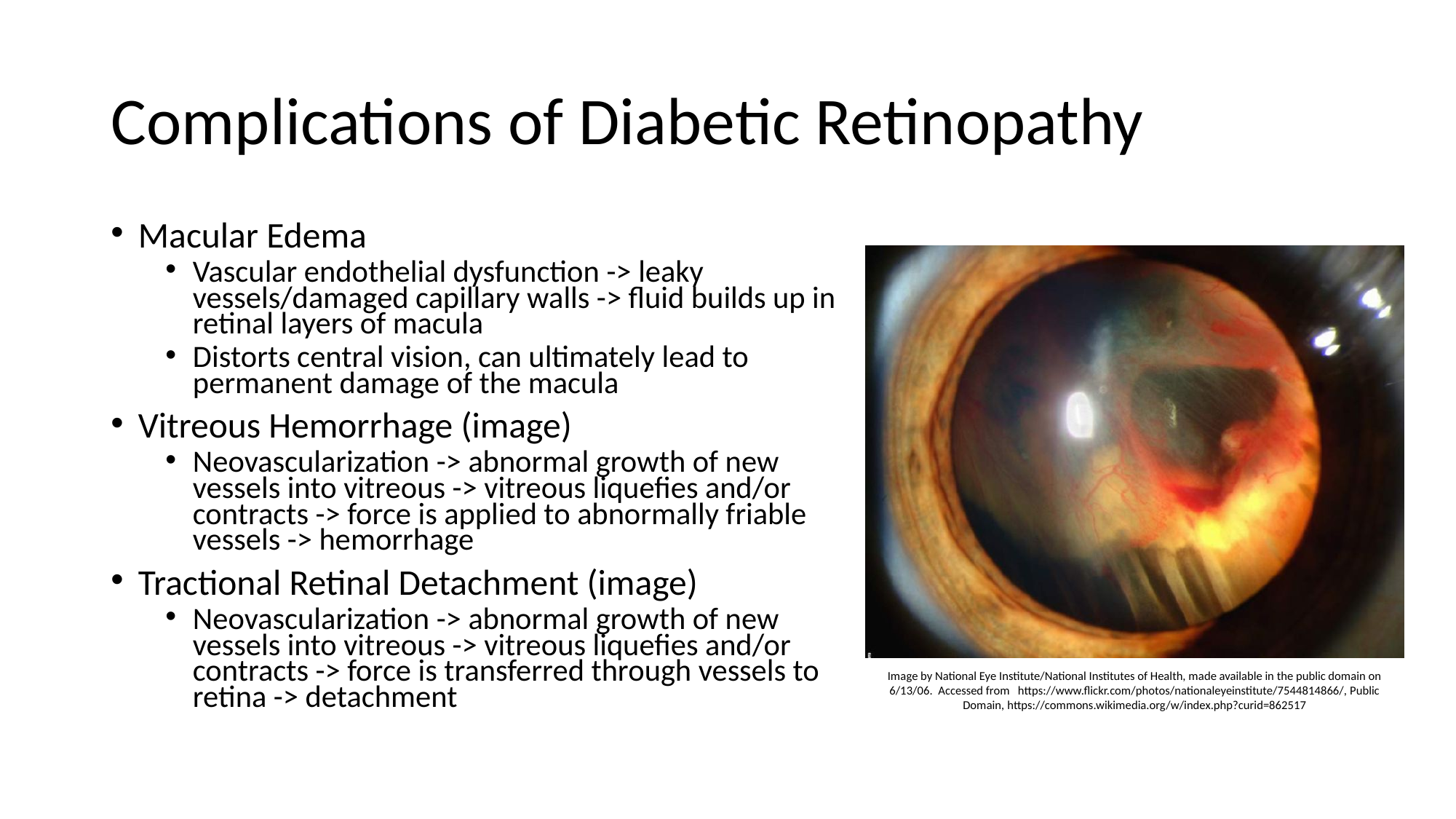

# Complications of Diabetic Retinopathy
Macular Edema
Vascular endothelial dysfunction -> leaky vessels/damaged capillary walls -> fluid builds up in retinal layers of macula
Distorts central vision, can ultimately lead to permanent damage of the macula
Vitreous Hemorrhage (image)
Neovascularization -> abnormal growth of new vessels into vitreous -> vitreous liquefies and/or contracts -> force is applied to abnormally friable vessels -> hemorrhage
Tractional Retinal Detachment (image)
Neovascularization -> abnormal growth of new vessels into vitreous -> vitreous liquefies and/or contracts -> force is transferred through vessels to retina -> detachment
Image by National Eye Institute/National Institutes of Health, made available in the public domain on 6/13/06. Accessed from https://www.flickr.com/photos/nationaleyeinstitute/7544814866/, Public Domain, https://commons.wikimedia.org/w/index.php?curid=862517

## Slide 16
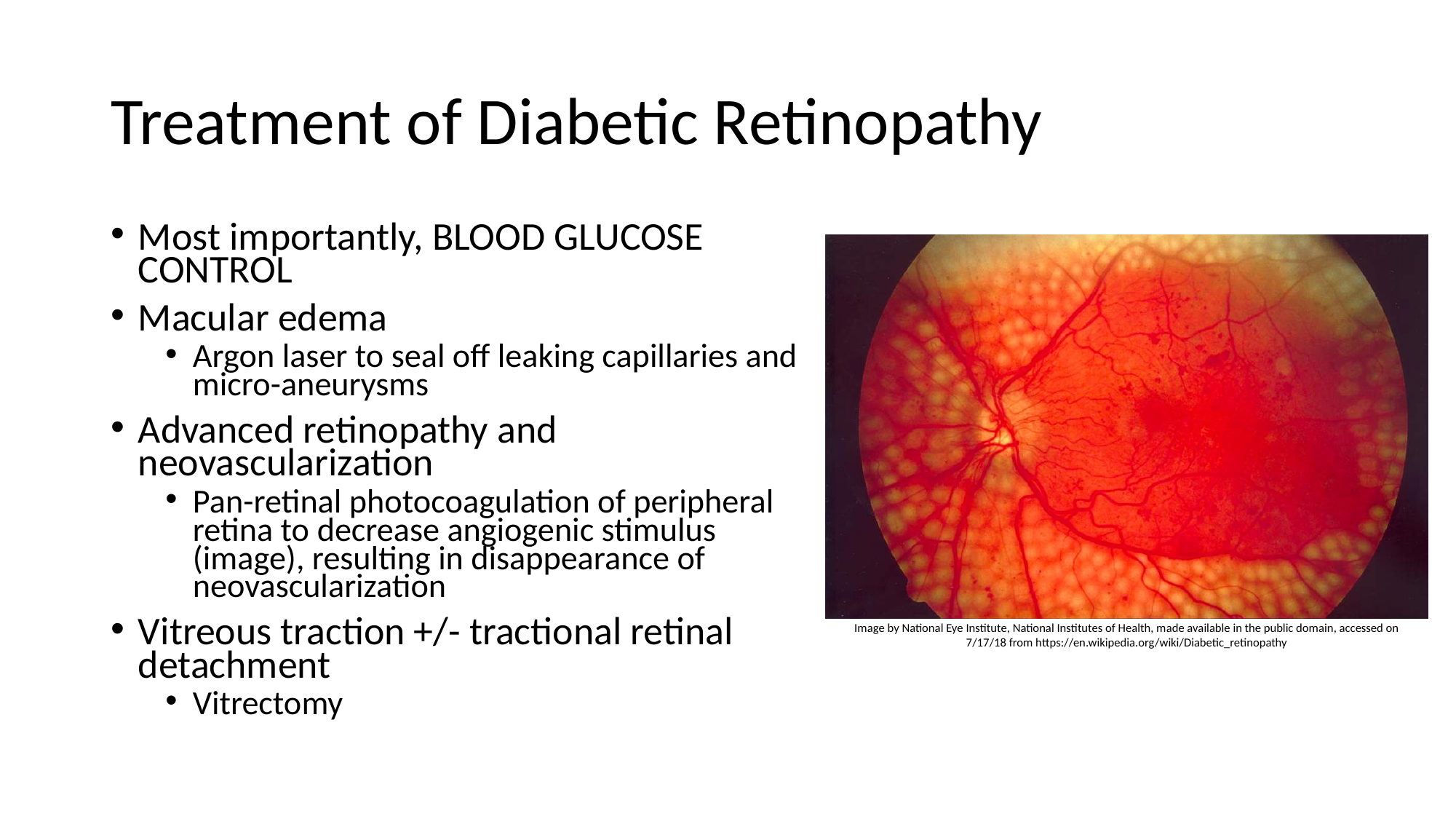

# Treatment of Diabetic Retinopathy
Most importantly, BLOOD GLUCOSE CONTROL
Macular edema
Argon laser to seal off leaking capillaries and micro-aneurysms
Advanced retinopathy and neovascularization
Pan-retinal photocoagulation of peripheral retina to decrease angiogenic stimulus (image), resulting in disappearance of neovascularization
Vitreous traction +/- tractional retinal detachment
Vitrectomy
Image by National Eye Institute, National Institutes of Health, made available in the public domain, accessed on 7/17/18 from https://en.wikipedia.org/wiki/Diabetic_retinopathy

## Slide 17
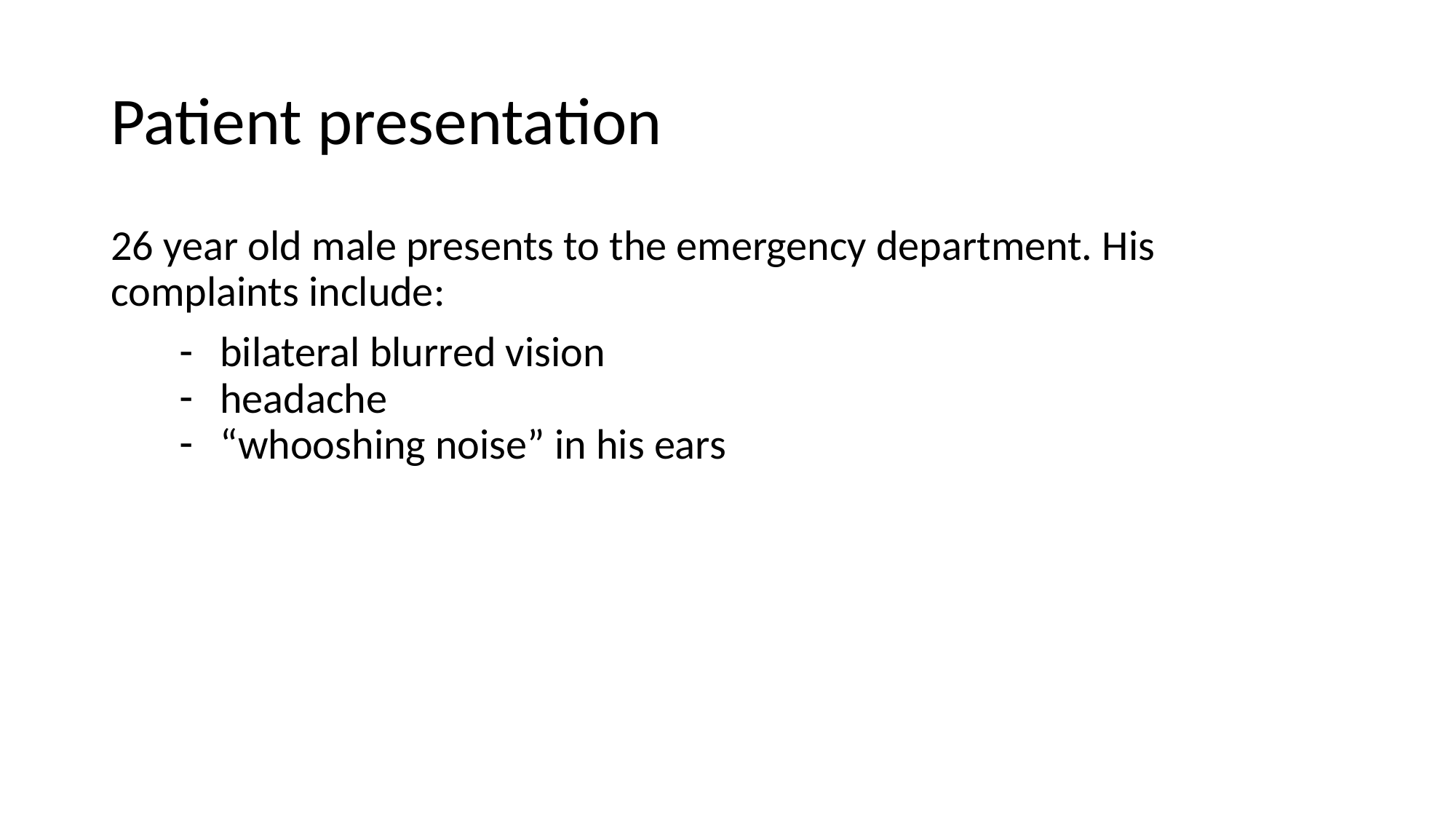

# Patient presentation
26 year old male presents to the emergency department. His complaints include:
bilateral blurred vision
headache
“whooshing noise” in his ears

## Slide 18
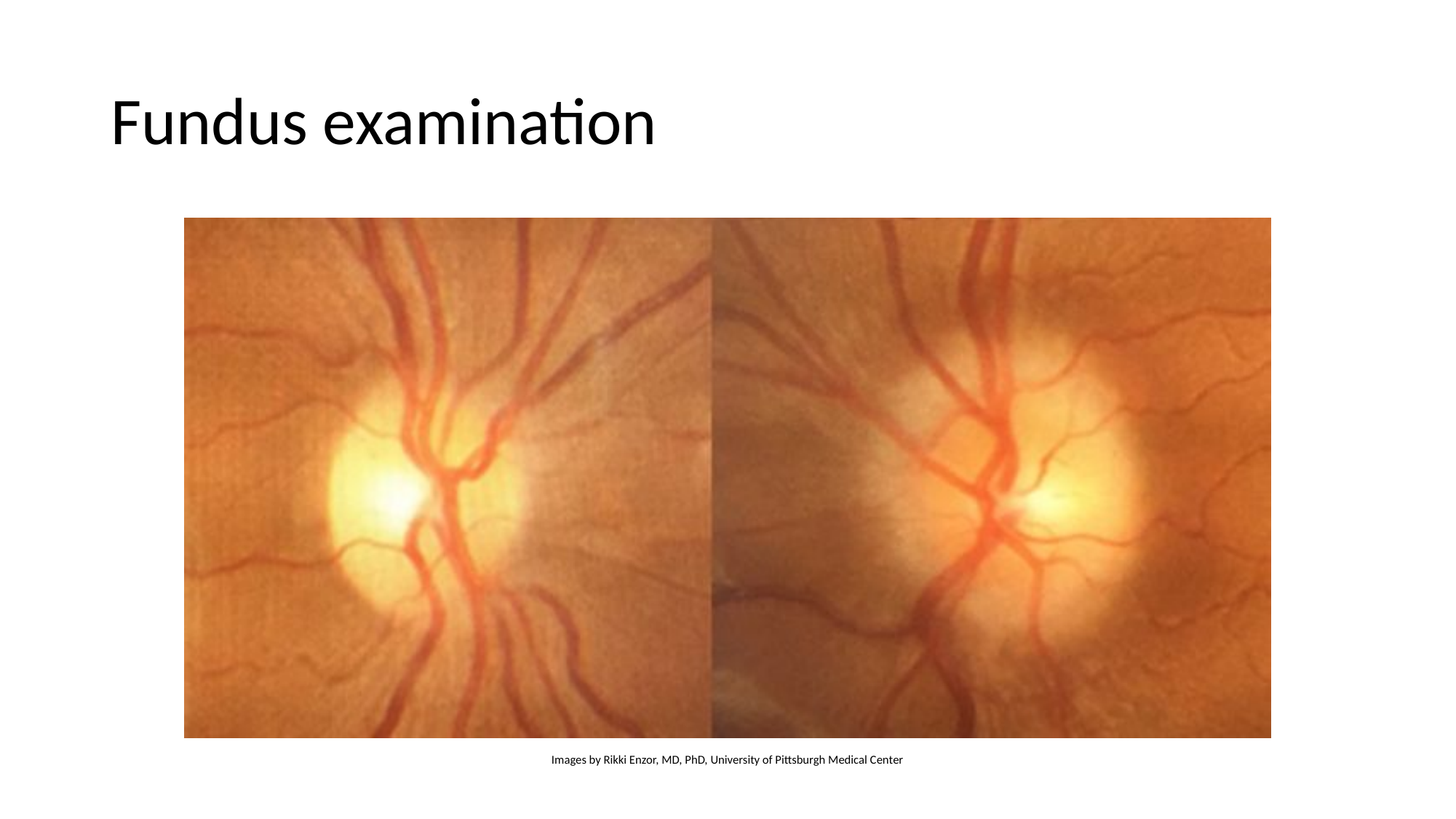

# Fundus examination
Images by Rikki Enzor, MD, PhD, University of Pittsburgh Medical Center

## Slide 19
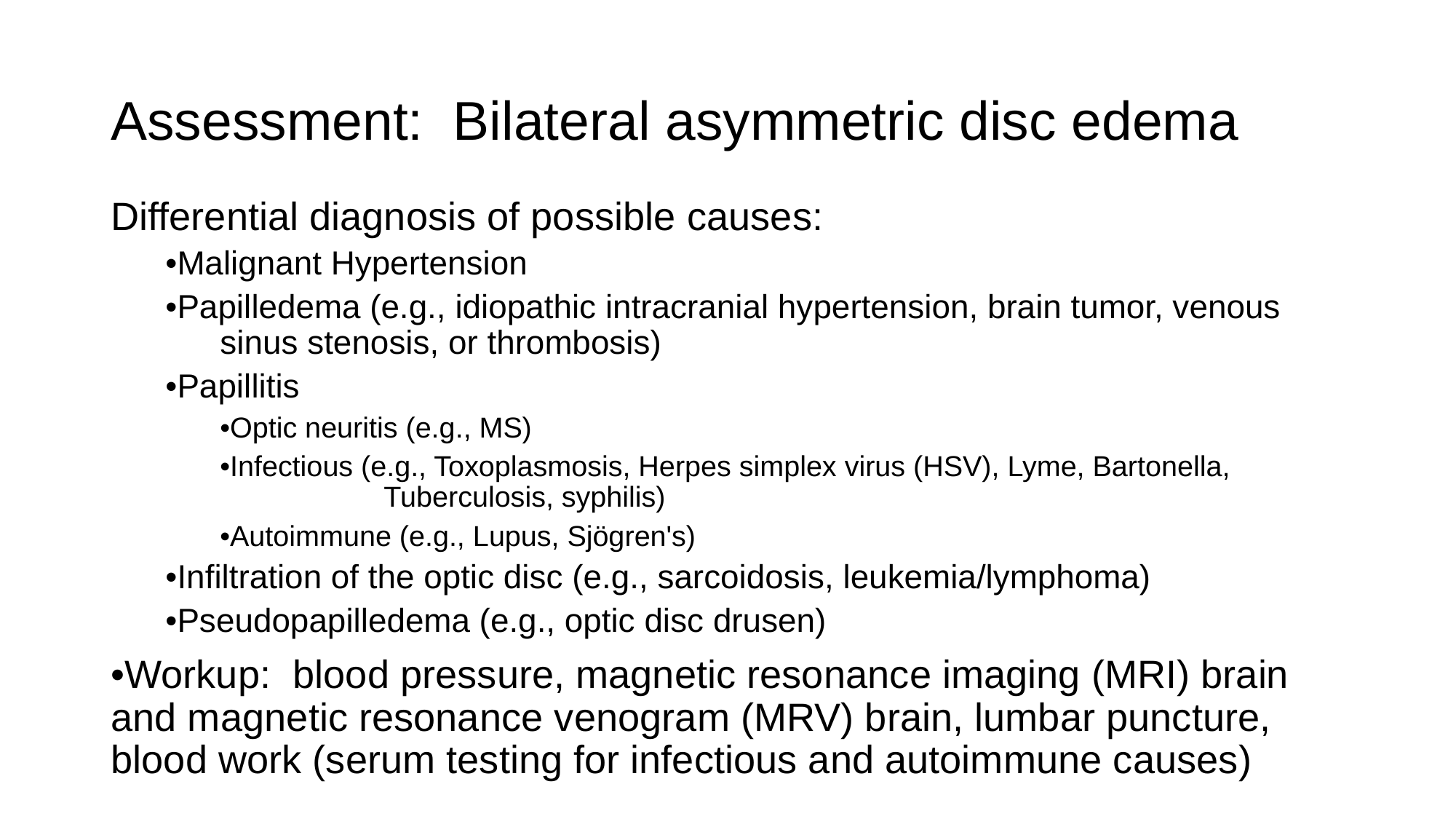

# Assessment: Bilateral asymmetric disc edema
Differential diagnosis of possible causes:
•Malignant Hypertension
•Papilledema (e.g., idiopathic intracranial hypertension, brain tumor, venous 		sinus stenosis, or thrombosis)
•Papillitis
•Optic neuritis (e.g., MS)
•Infectious (e.g., Toxoplasmosis, Herpes simplex virus (HSV), Lyme, Bartonella,			Tuberculosis, syphilis)
•Autoimmune (e.g., Lupus, Sjögren's)
•Infiltration of the optic disc (e.g., sarcoidosis, leukemia/lymphoma)
•Pseudopapilledema (e.g., optic disc drusen)
•Workup: blood pressure, magnetic resonance imaging (MRI) brain and magnetic resonance venogram (MRV) brain, lumbar puncture, blood work (serum testing for infectious and autoimmune causes)

## Slide 20
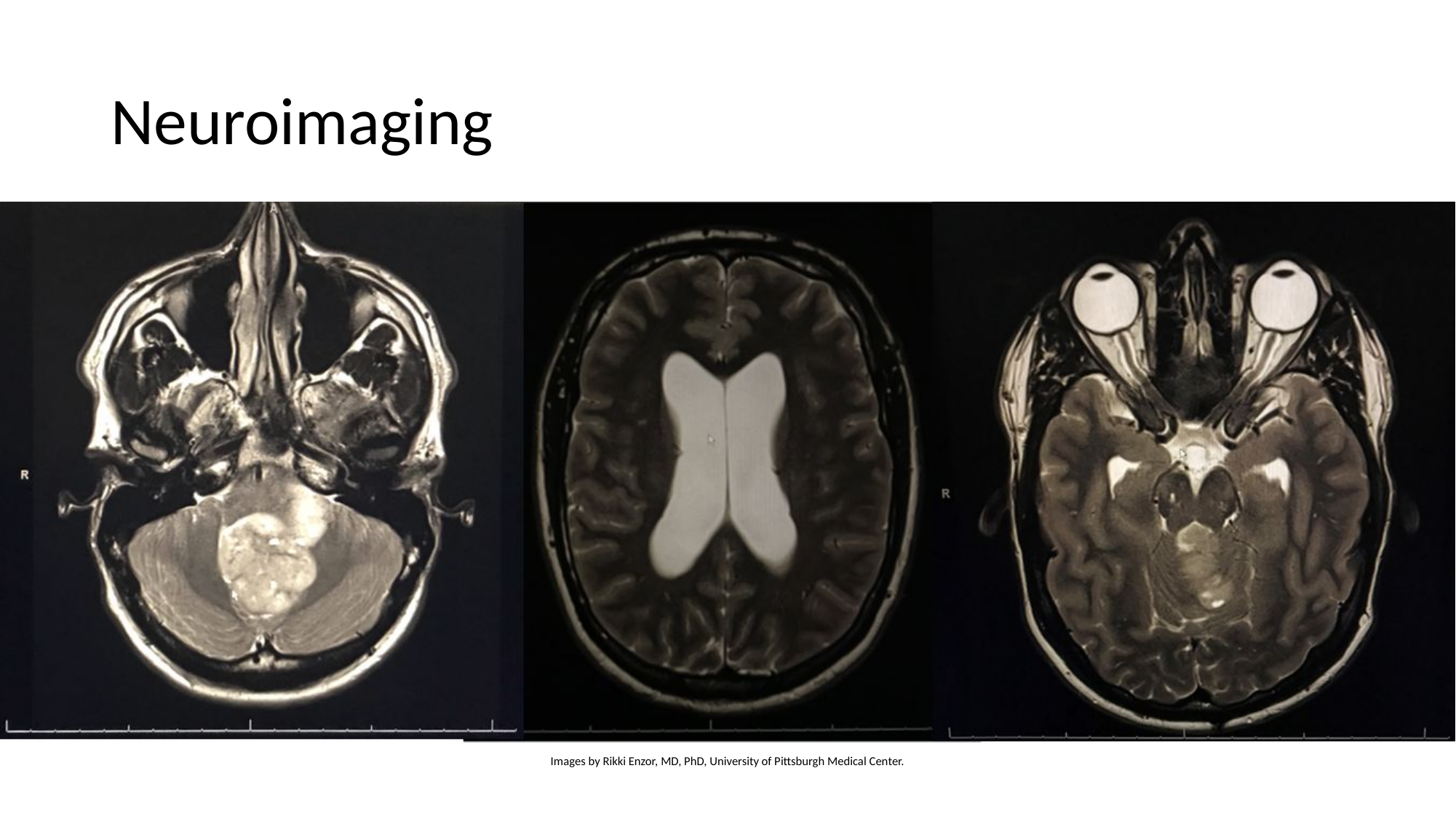

# Neuroimaging
Images by Rikki Enzor, MD, PhD, University of Pittsburgh Medical Center.

## Slide 21
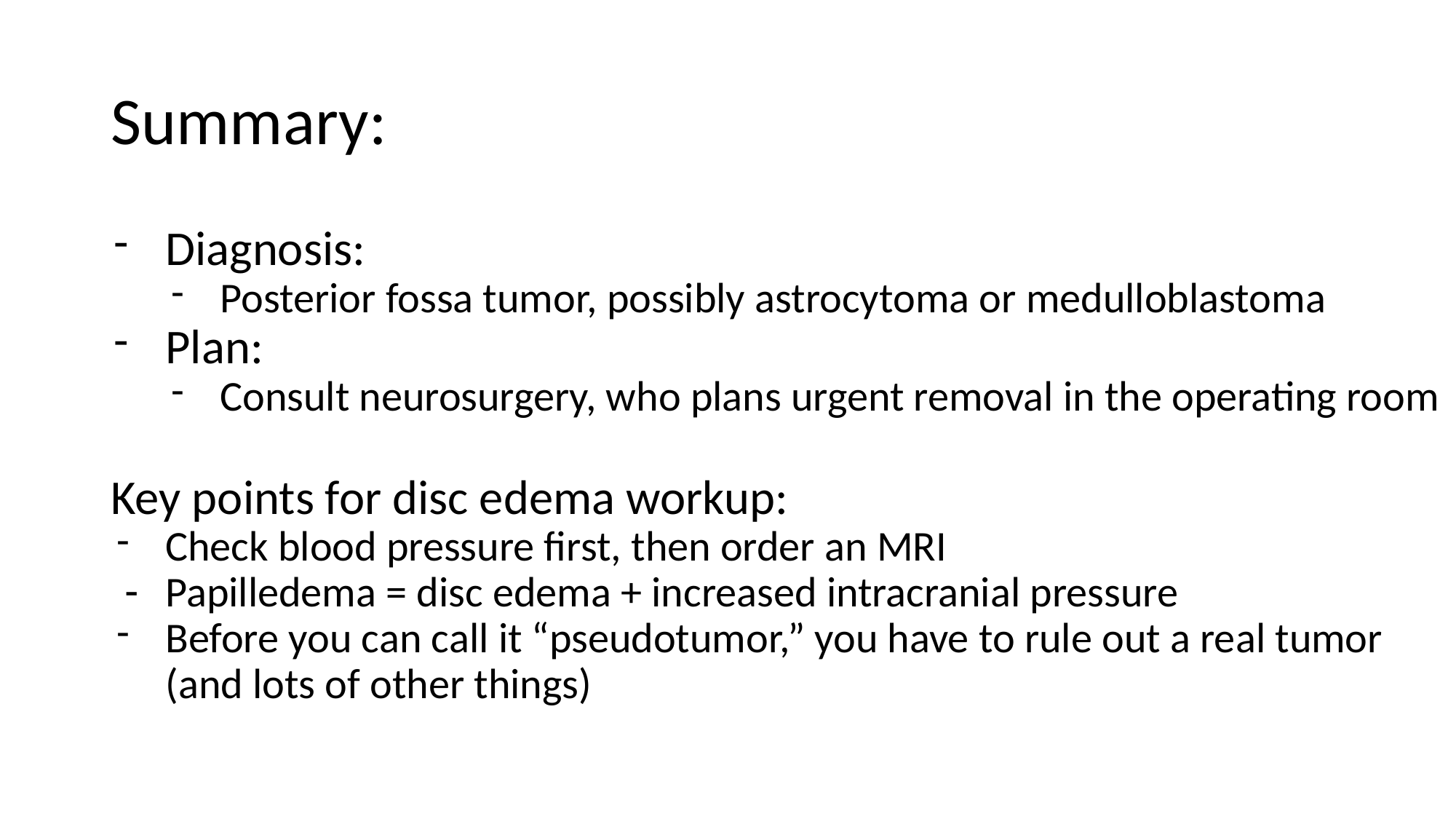

# Summary:
Diagnosis:
Posterior fossa tumor, possibly astrocytoma or medulloblastoma
Plan:
Consult neurosurgery, who plans urgent removal in the operating room
Key points for disc edema workup:
Check blood pressure first, then order an MRI
Papilledema = disc edema + increased intracranial pressure
Before you can call it “pseudotumor,” you have to rule out a real tumor (and lots of other things)

## Slide 22
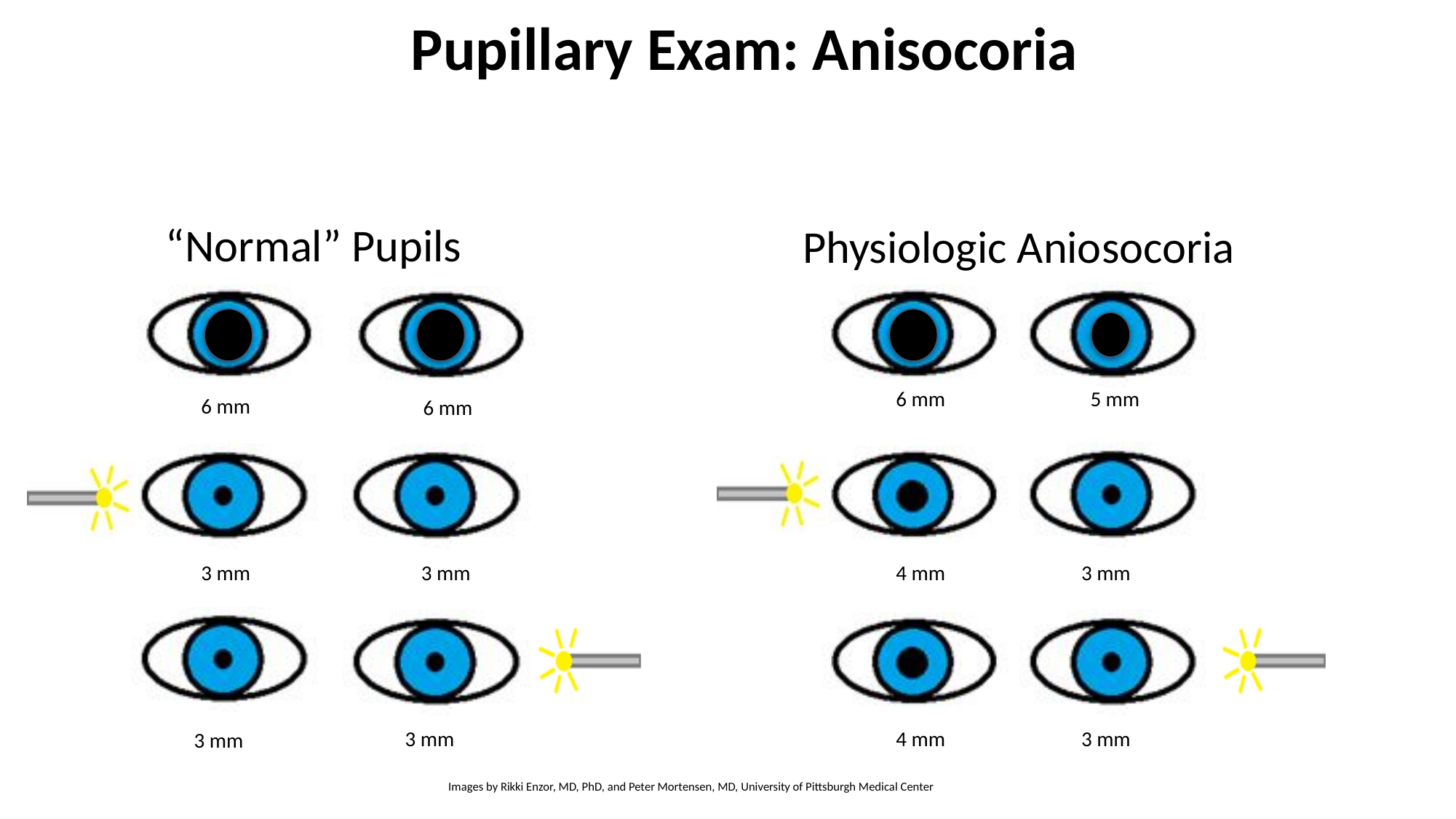

Pupillary Exam: Anisocoria
“Normal” Pupils
Physiologic Aniosocoria
6 mm
5 mm
6 mm
6 mm
3 mm
3 mm
4 mm
3 mm
3 mm
4 mm
3 mm
3 mm
Images by Rikki Enzor, MD, PhD, and Peter Mortensen, MD, University of Pittsburgh Medical Center

## Slide 23
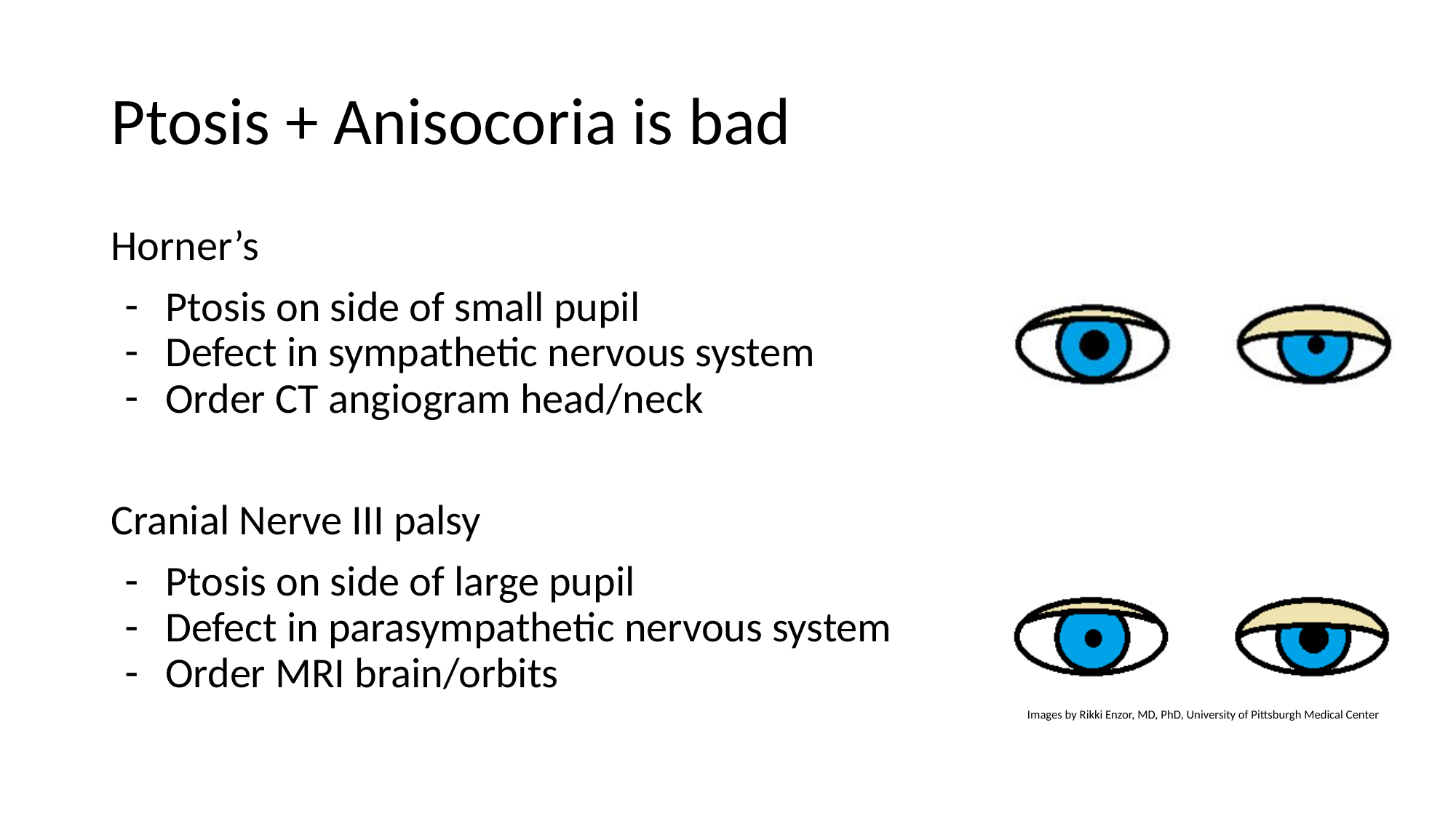

# Ptosis + Anisocoria is bad
Horner’s
Ptosis on side of small pupil
Defect in sympathetic nervous system
Order CT angiogram head/neck
Cranial Nerve III palsy
Ptosis on side of large pupil
Defect in parasympathetic nervous system
Order MRI brain/orbits
Images by Rikki Enzor, MD, PhD, University of Pittsburgh Medical Center
