# Supplementary material for: The Ophthalmology Mini-Elective Gives Vision to Preclinical Medical Students
Source: MedEdPORTAL. 2020 Nov 23;16:11024. doi: 10.15766/mep_2374-8265.11024 (PMC7703479; doi:10.15766/mep_2374-8265.11024)
Supplement: Supplementary file 1 — Course Syllabus.docxInstructor Introduction.docxWeekly Course Time Line & Objectives.docxSession 1 - Intro to Ophthalmology.pptxSession 2 - Anterior Segment.pptxSession 3 - Posterior Segment.pptxSession 4 - Eye Emergencies and Trauma.pptxLaboratory Session Guide.pdfPrecourse Survey.docxPre- and Posttest.docxPostcourse Survey.docxPre- and Posttest Answers.docx [file mep_2374-8265.11024-s001.zip › G. Session 4 - Eye Emergencies and Trauma.pptx]

## Slide 1
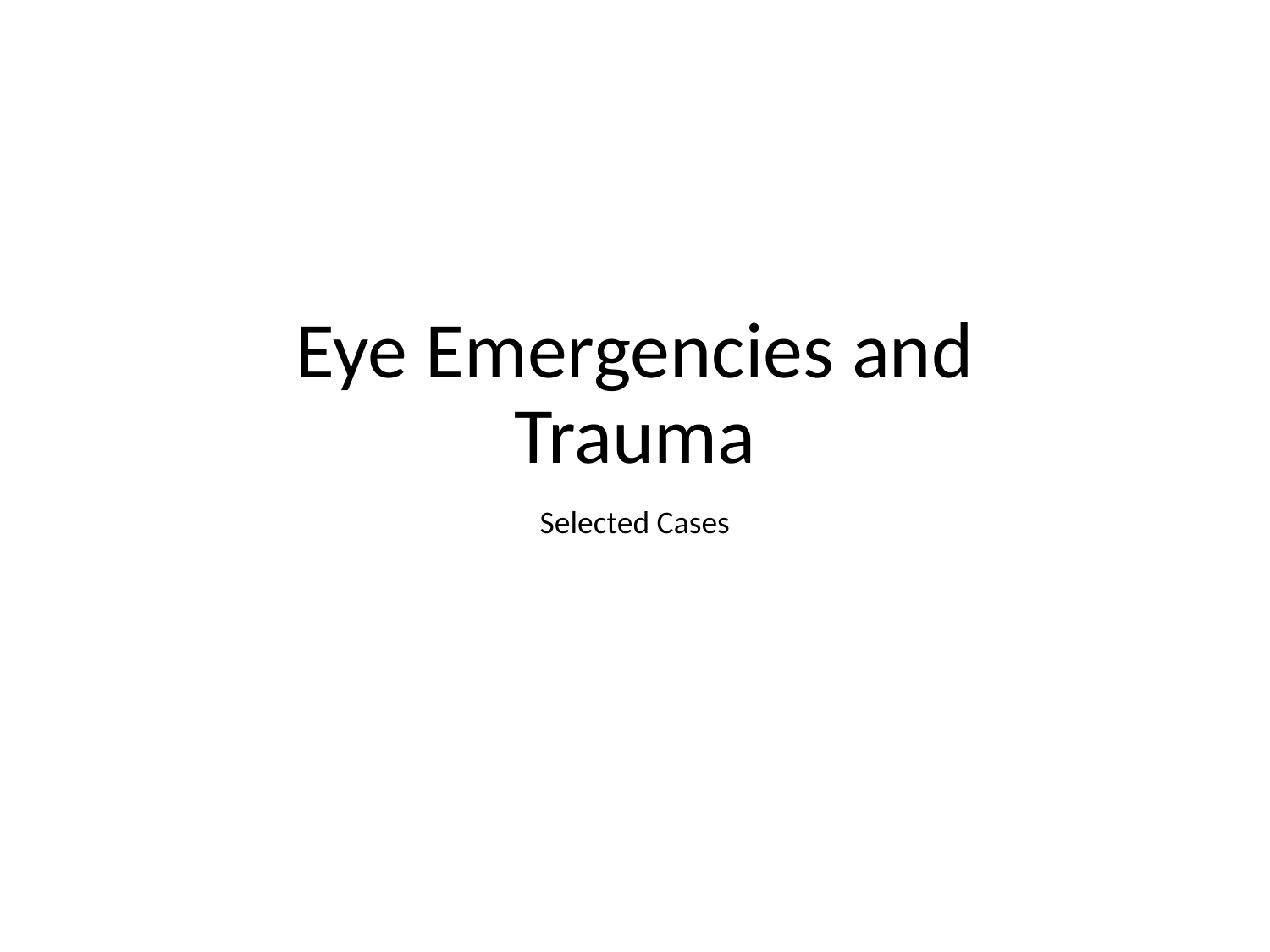

# Eye Emergencies and Trauma
Selected Cases

## Slide 2
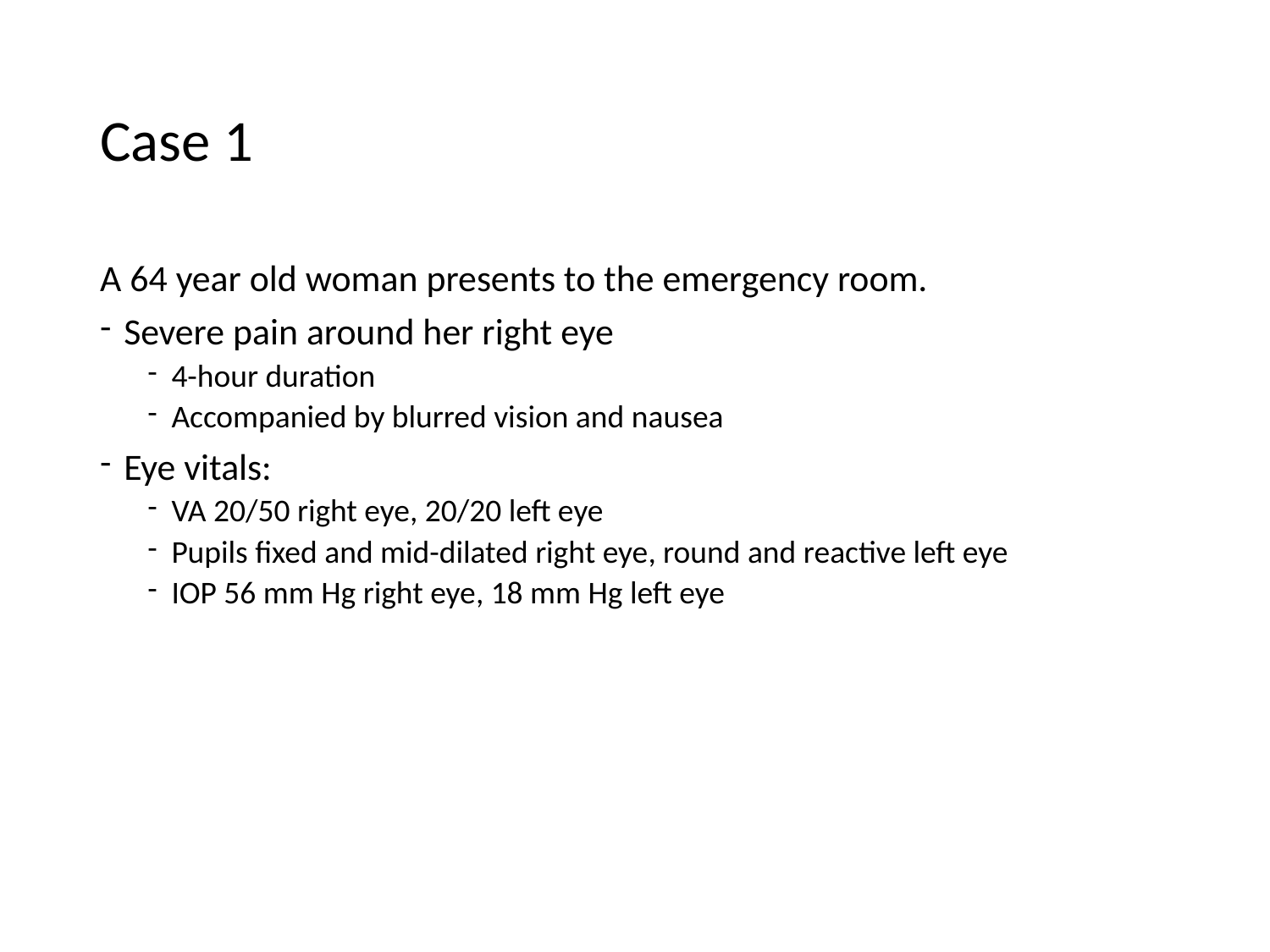

# Case 1
A 64 year old woman presents to the emergency room.
Severe pain around her right eye
4-hour duration
Accompanied by blurred vision and nausea
Eye vitals:
VA 20/50 right eye, 20/20 left eye
Pupils fixed and mid-dilated right eye, round and reactive left eye
IOP 56 mm Hg right eye, 18 mm Hg left eye

## Slide 3
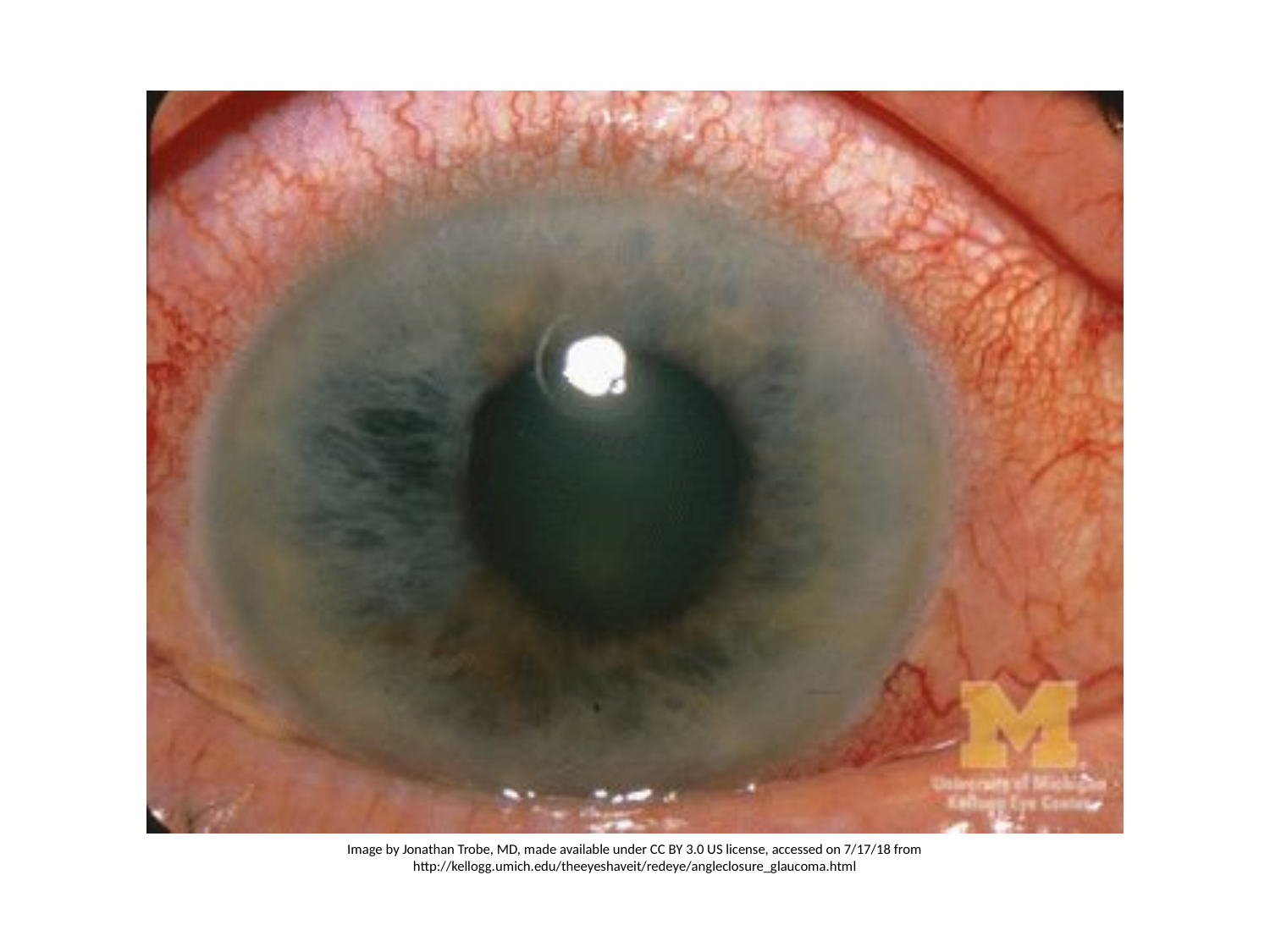

Image by Jonathan Trobe, MD, made available under CC BY 3.0 US license, accessed on 7/17/18 from http://kellogg.umich.edu/theeyeshaveit/redeye/angleclosure_glaucoma.html

## Slide 4
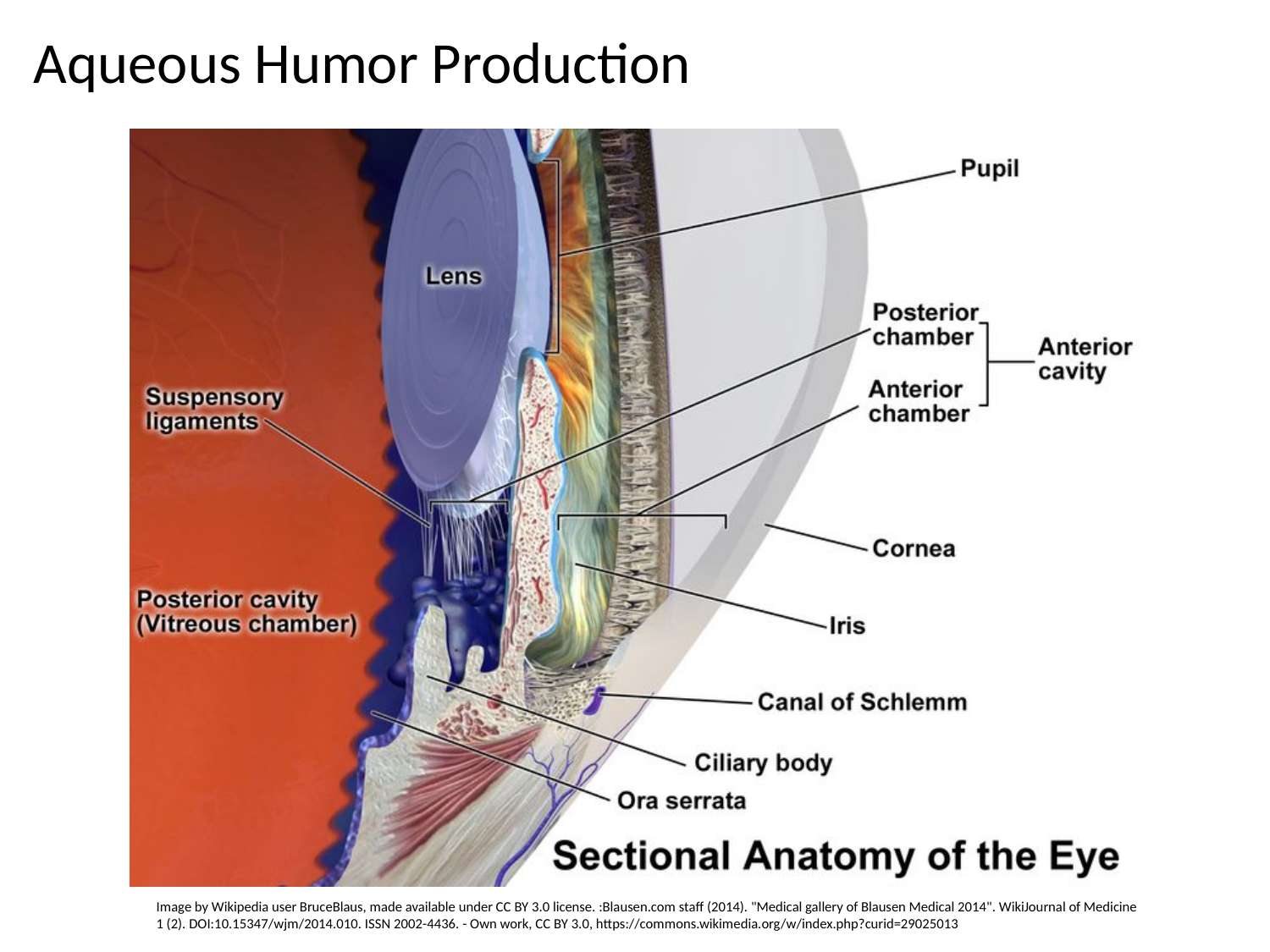

# Aqueous Humor Production
Image by Wikipedia user BruceBlaus, made available under CC BY 3.0 license. :Blausen.com staff (2014). "Medical gallery of Blausen Medical 2014". WikiJournal of Medicine 1 (2). DOI:10.15347/wjm/2014.010. ISSN 2002-4436. - Own work, CC BY 3.0, https://commons.wikimedia.org/w/index.php?curid=29025013

## Slide 5
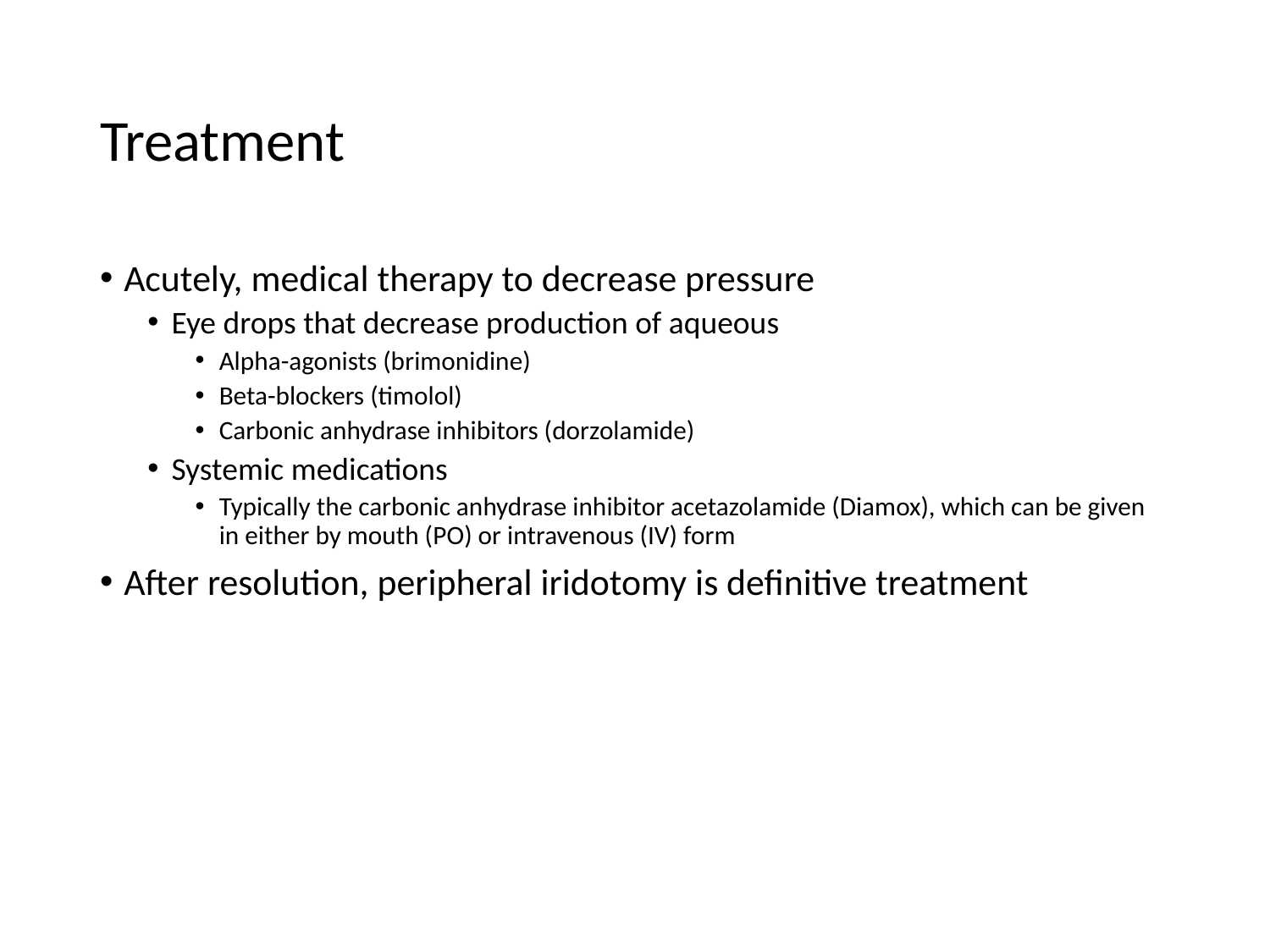

# Treatment
Acutely, medical therapy to decrease pressure
Eye drops that decrease production of aqueous
Alpha-agonists (brimonidine)
Beta-blockers (timolol)
Carbonic anhydrase inhibitors (dorzolamide)
Systemic medications
Typically the carbonic anhydrase inhibitor acetazolamide (Diamox), which can be given in either by mouth (PO) or intravenous (IV) form
After resolution, peripheral iridotomy is definitive treatment

## Slide 6
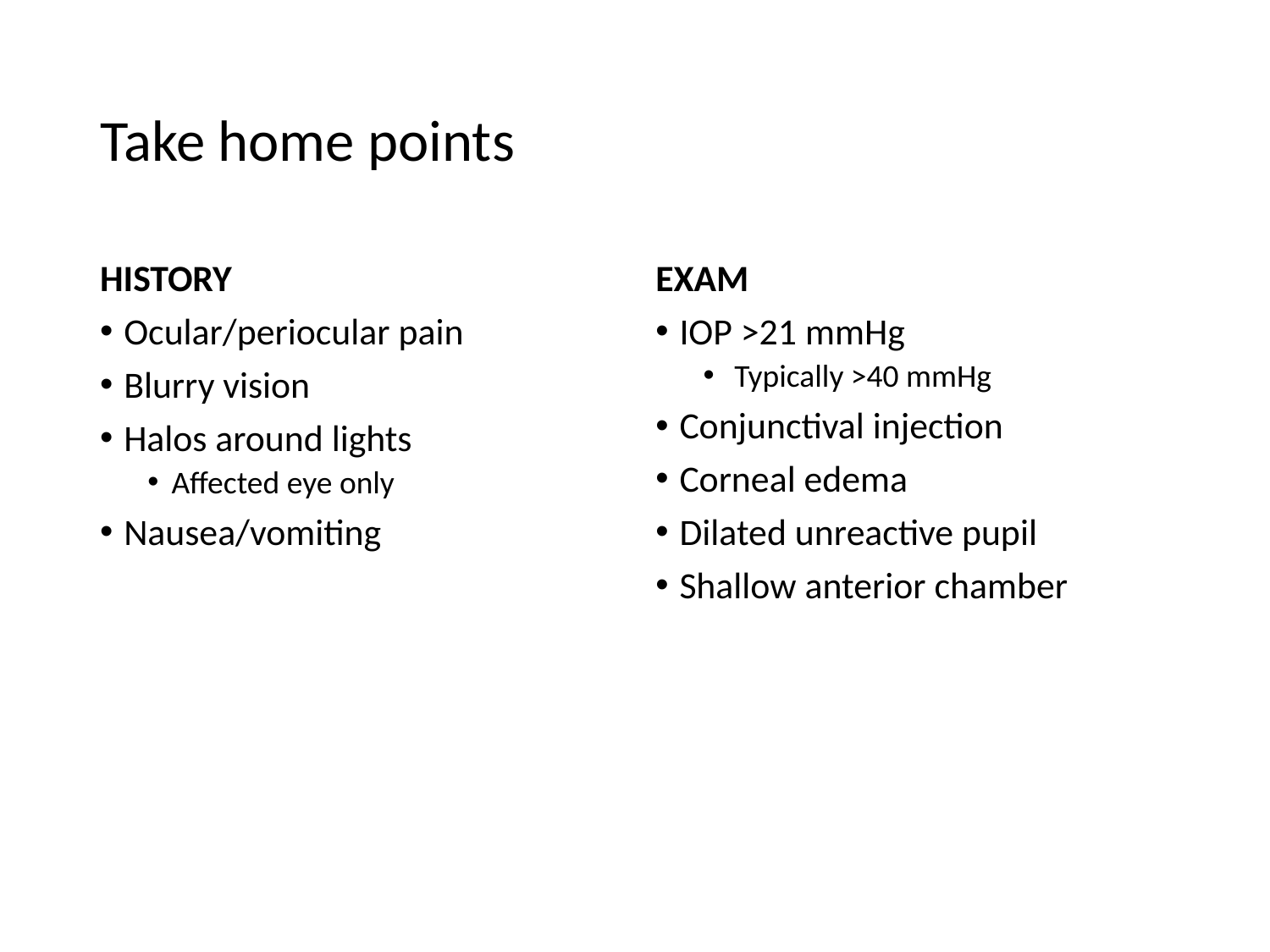

# Take home points
HISTORY
Ocular/periocular pain
Blurry vision
Halos around lights
Affected eye only
Nausea/vomiting
EXAM
IOP >21 mmHg
 Typically >40 mmHg
Conjunctival injection
Corneal edema
Dilated unreactive pupil
Shallow anterior chamber

## Slide 7
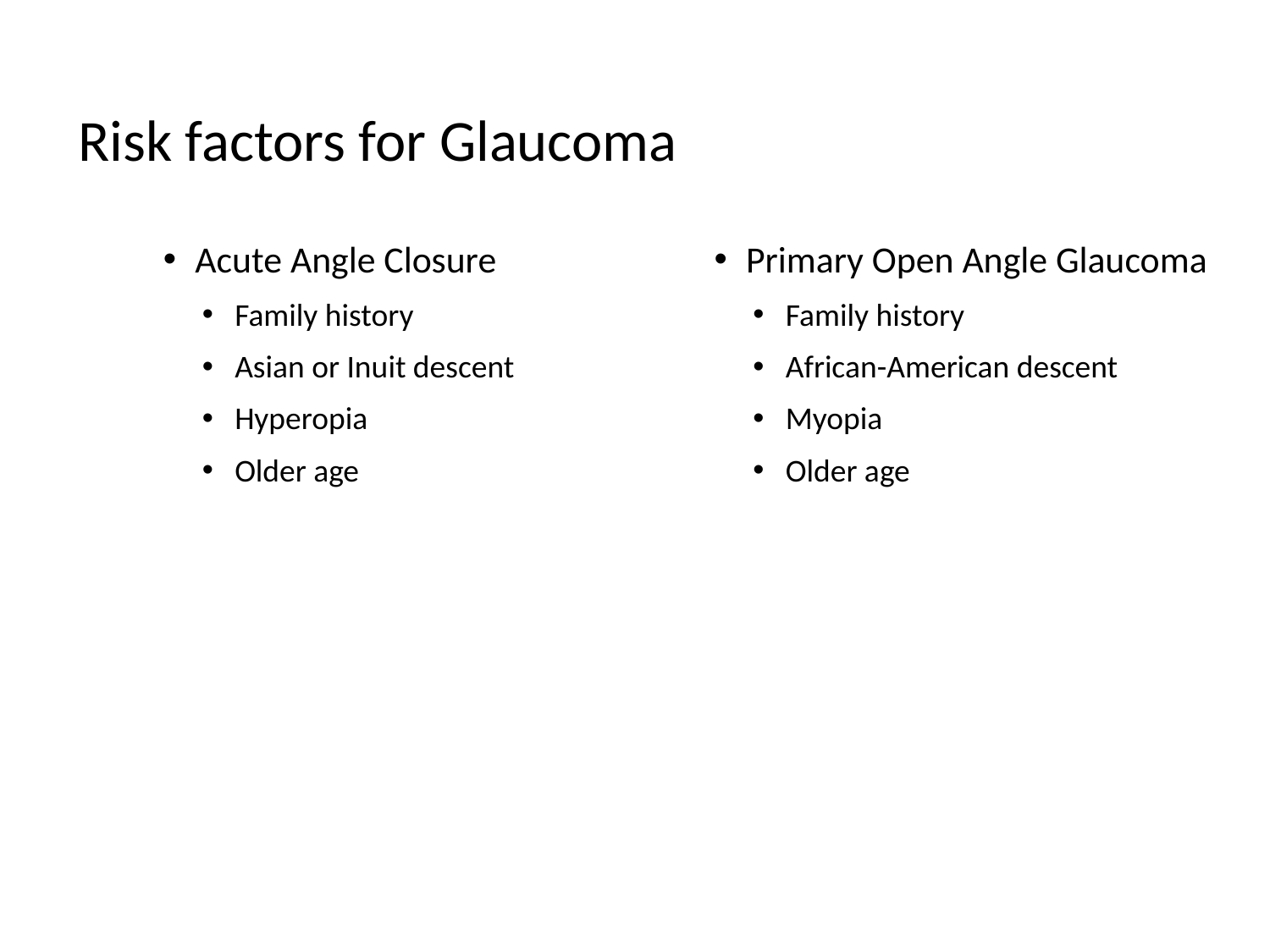

# Risk factors for Glaucoma
Acute Angle Closure
Family history
Asian or Inuit descent
Hyperopia
Older age
Primary Open Angle Glaucoma
Family history
African-American descent
Myopia
Older age

## Slide 8
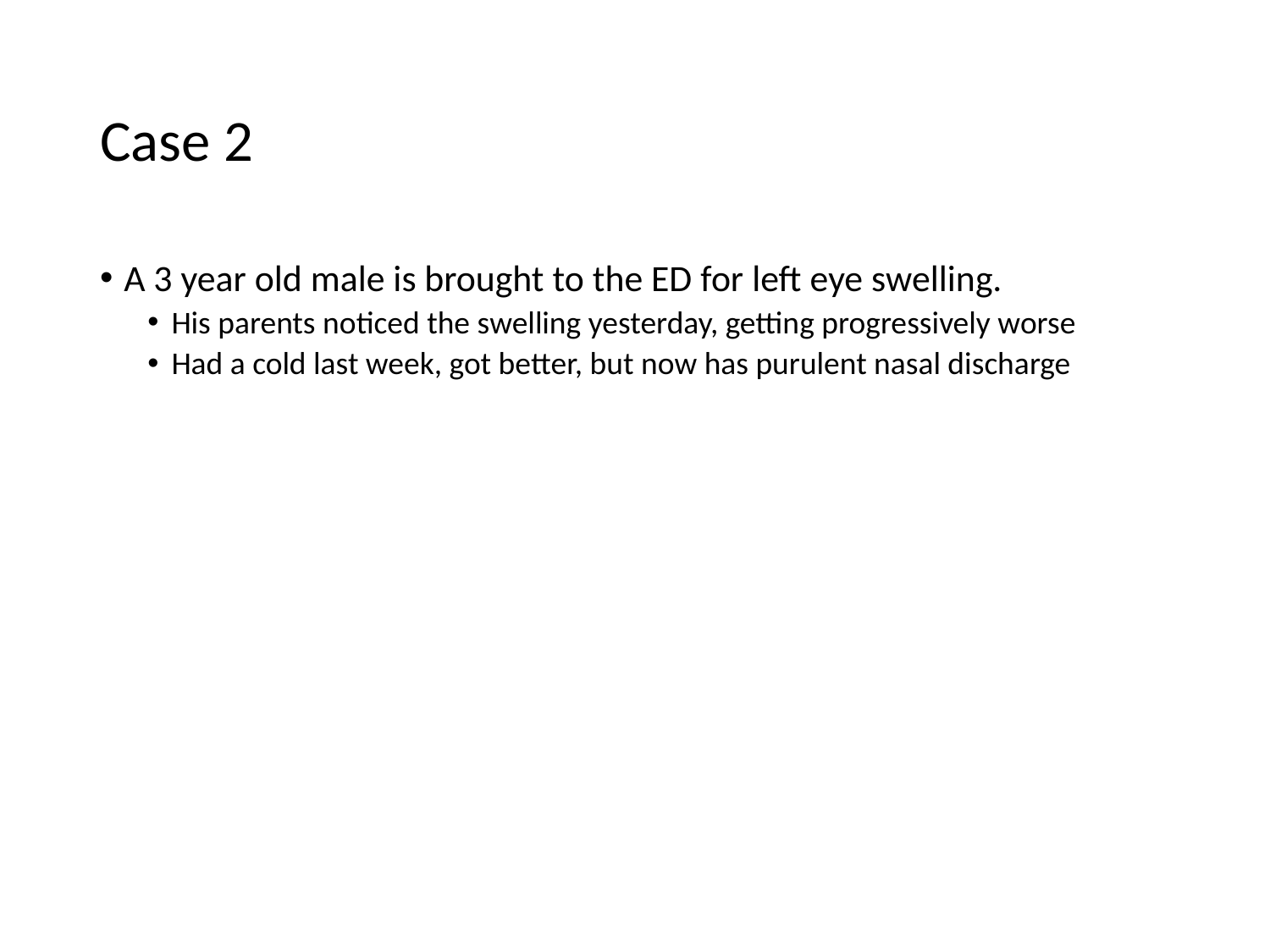

# Case 2
A 3 year old male is brought to the ED for left eye swelling.
His parents noticed the swelling yesterday, getting progressively worse
Had a cold last week, got better, but now has purulent nasal discharge

## Slide 9
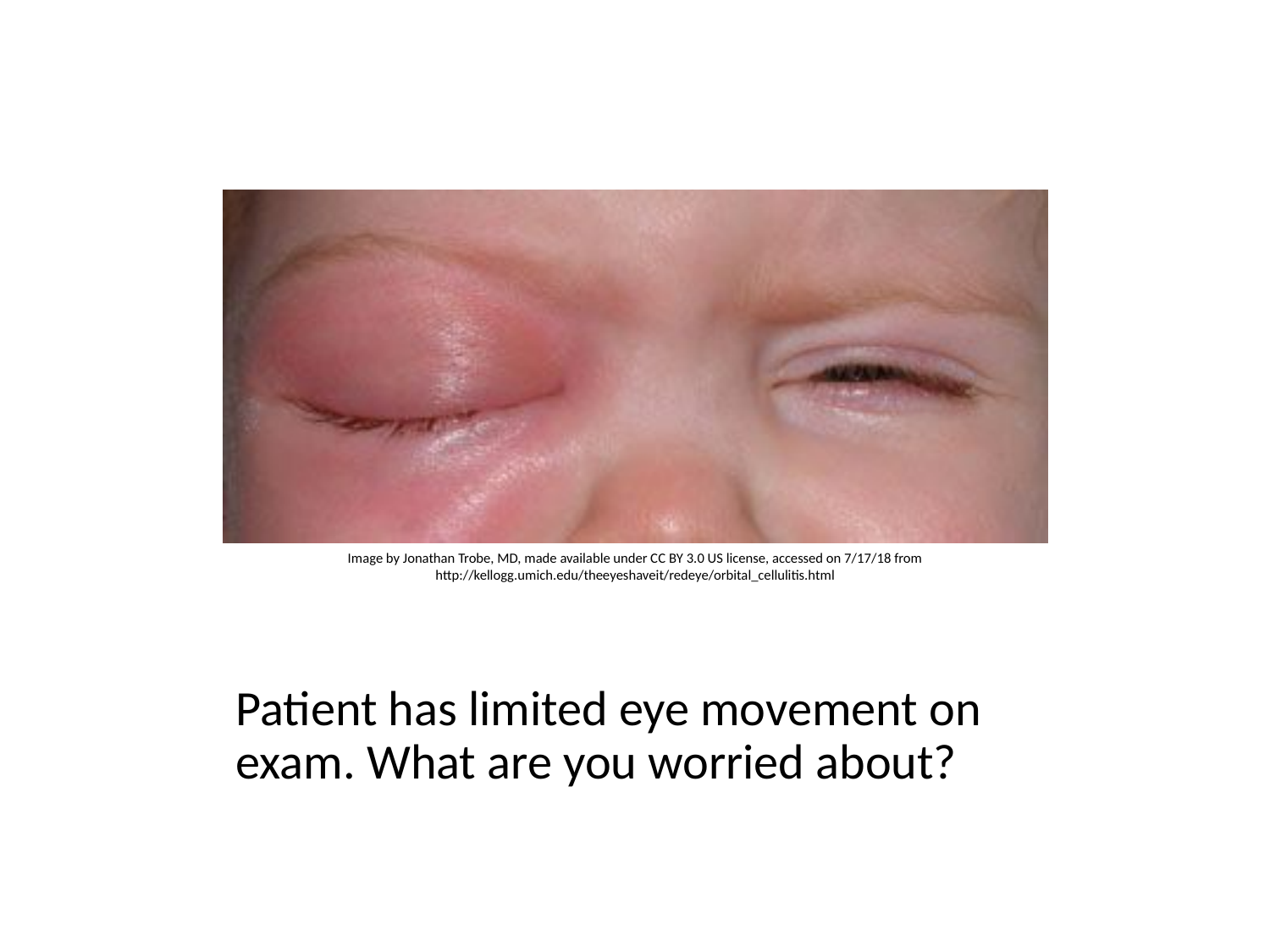

Image by Jonathan Trobe, MD, made available under CC BY 3.0 US license, accessed on 7/17/18 from http://kellogg.umich.edu/theeyeshaveit/redeye/orbital_cellulitis.html
# Patient has limited eye movement on exam. What are you worried about?

## Slide 10
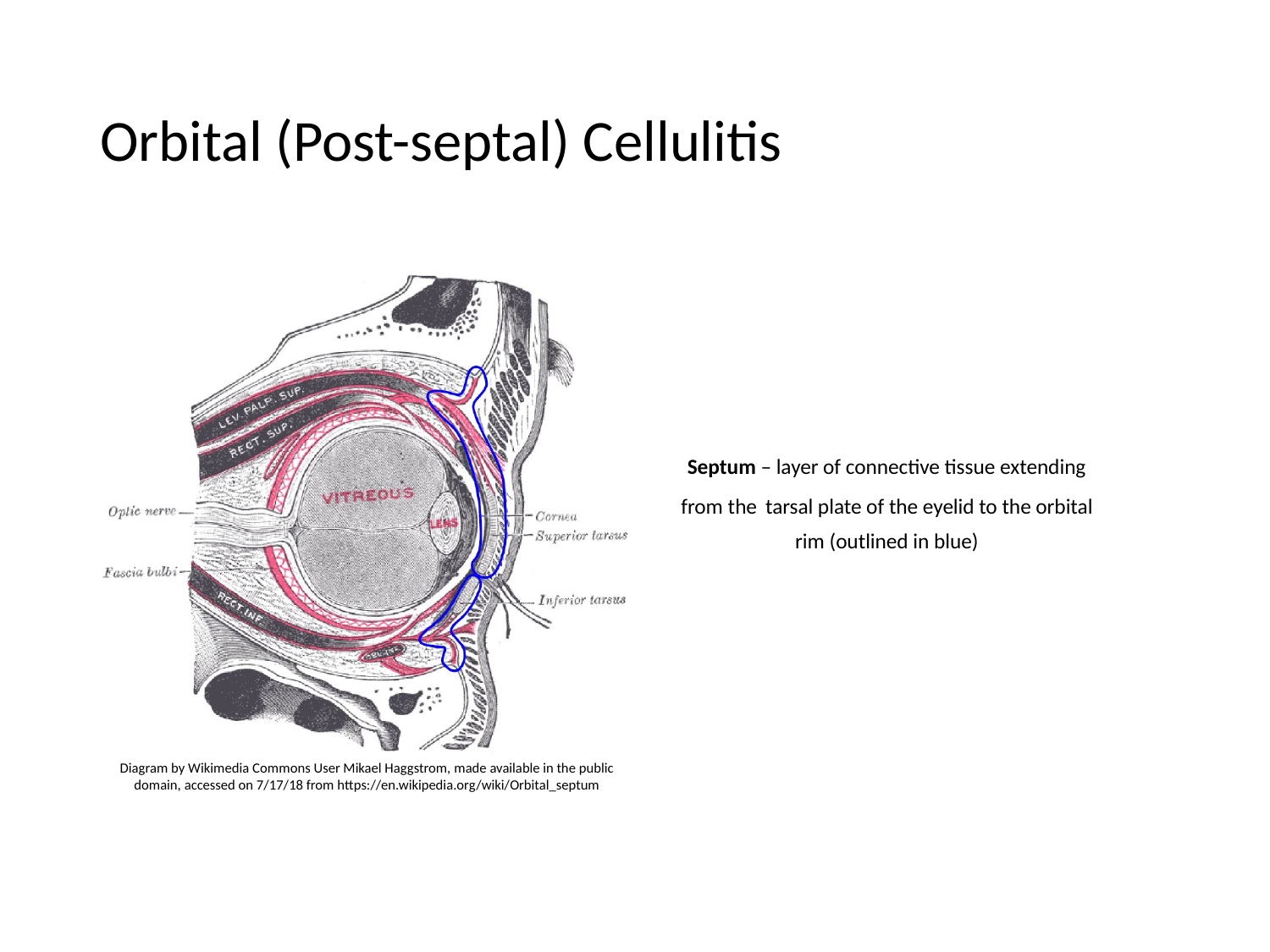

# Orbital (Post-septal) Cellulitis
Septum – layer of connective tissue extending from the tarsal plate of the eyelid to the orbital rim (outlined in blue)
Diagram by Wikimedia Commons User Mikael Haggstrom, made available in the public domain, accessed on 7/17/18 from https://en.wikipedia.org/wiki/Orbital_septum

## Slide 11
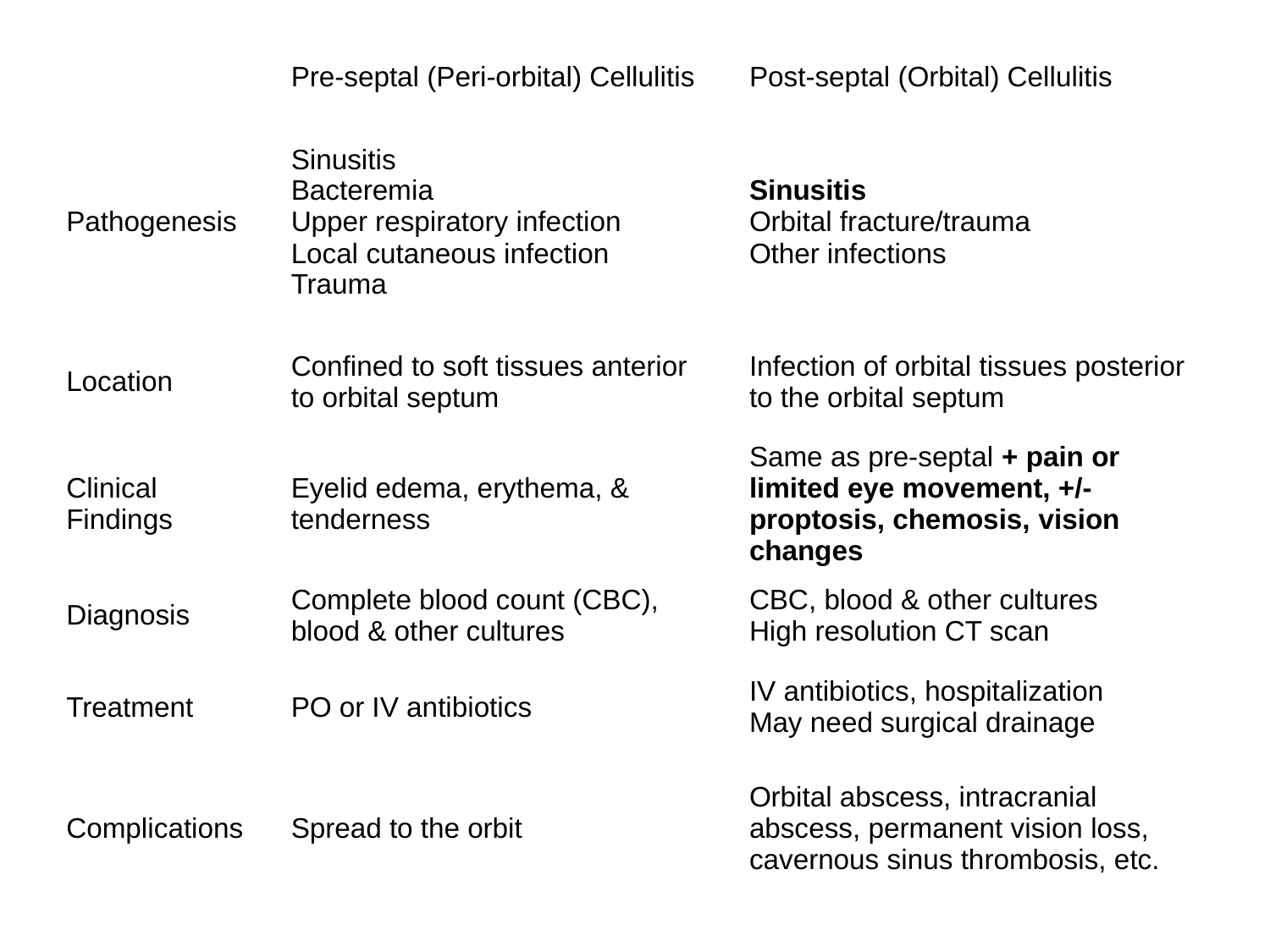

| | Pre-septal (Peri-orbital) Cellulitis | Post-septal (Orbital) Cellulitis |
| --- | --- | --- |
| Pathogenesis | Sinusitis Bacteremia Upper respiratory infection Local cutaneous infection Trauma | Sinusitis Orbital fracture/trauma Other infections |
| Location | Confined to soft tissues anterior to orbital septum | Infection of orbital tissues posterior to the orbital septum |
| Clinical Findings | Eyelid edema, erythema, & tenderness | Same as pre-septal + pain or limited eye movement, +/- proptosis, chemosis, vision changes |
| Diagnosis | Complete blood count (CBC), blood & other cultures | CBC, blood & other cultures High resolution CT scan |
| Treatment | PO or IV antibiotics | IV antibiotics, hospitalization May need surgical drainage |
| Complications | Spread to the orbit | Orbital abscess, intracranial abscess, permanent vision loss, cavernous sinus thrombosis, etc. |

## Slide 12
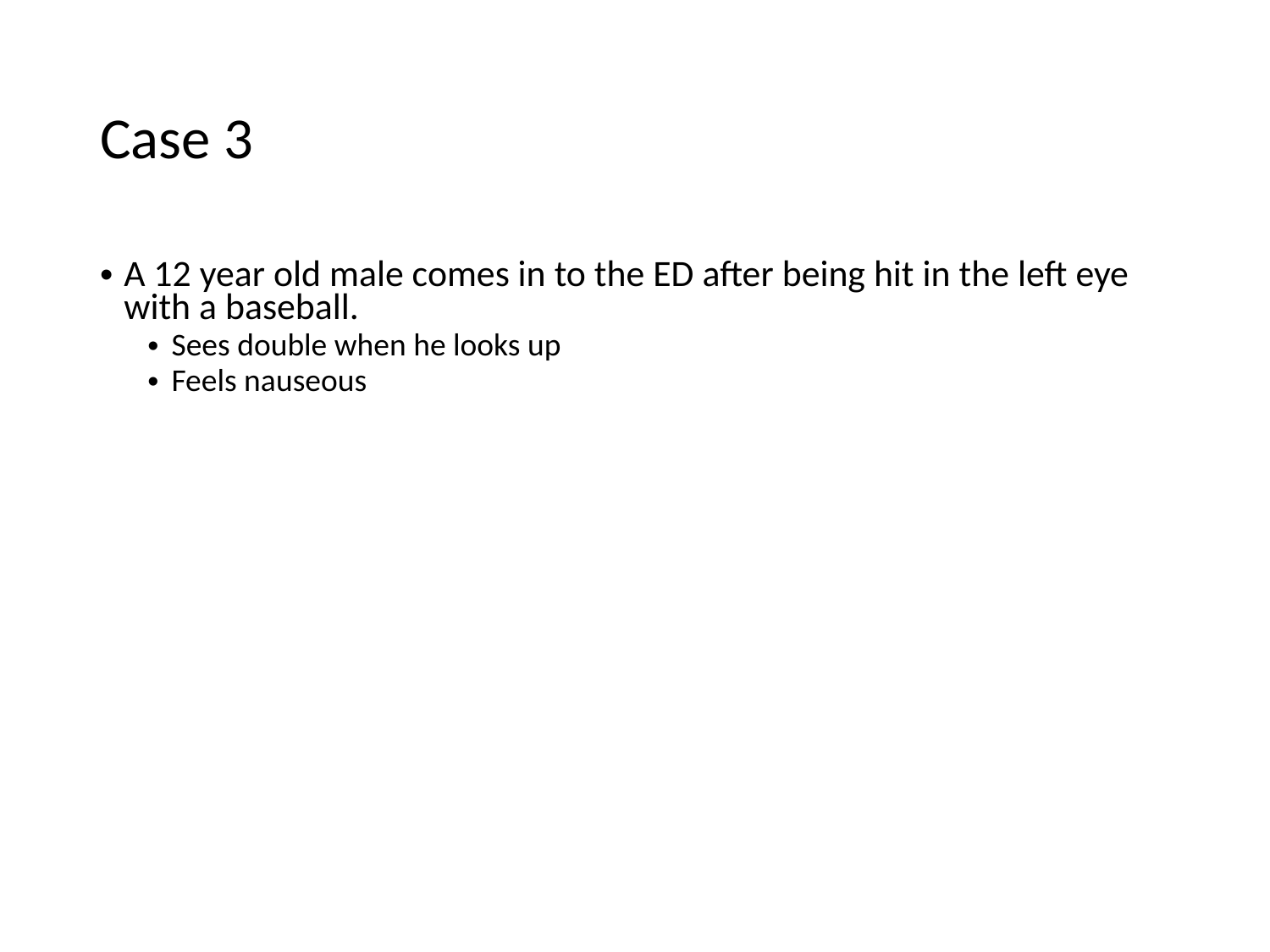

# Case 3
A 12 year old male comes in to the ED after being hit in the left eye with a baseball.
Sees double when he looks up
Feels nauseous

## Slide 13
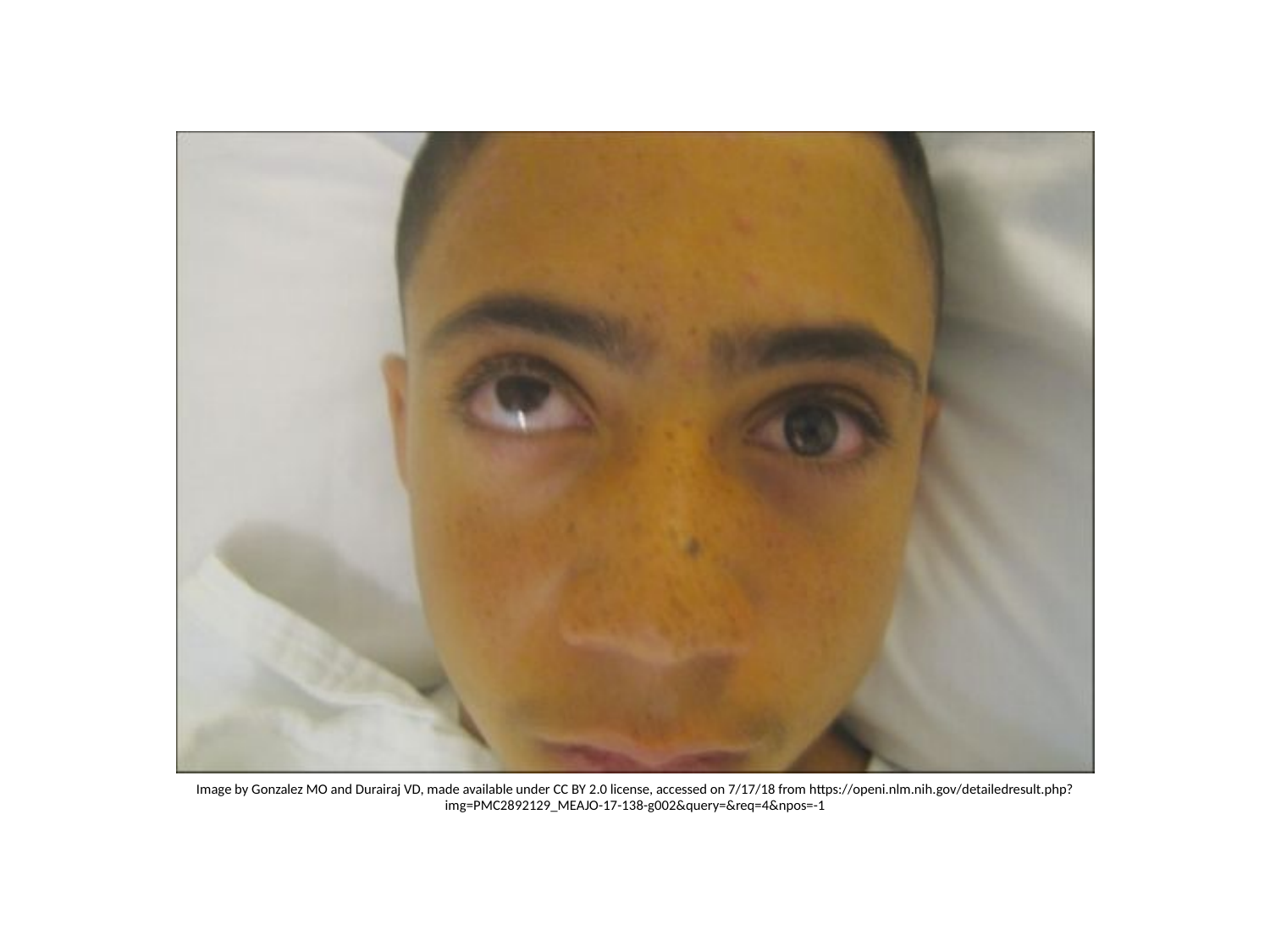

Image by Gonzalez MO and Durairaj VD, made available under CC BY 2.0 license, accessed on 7/17/18 from https://openi.nlm.nih.gov/detailedresult.php?img=PMC2892129_MEAJO-17-138-g002&query=&req=4&npos=-1

## Slide 14
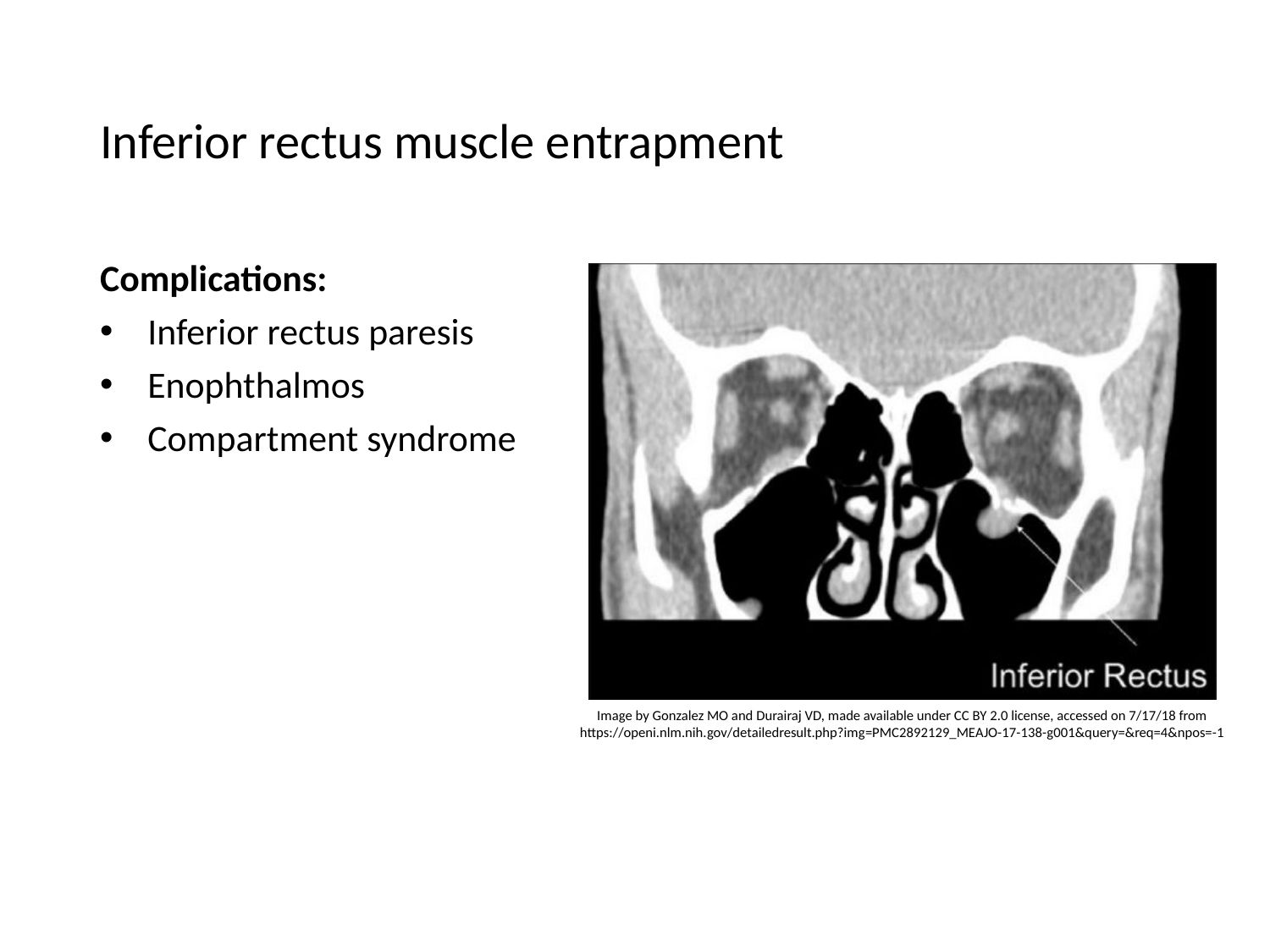

# Inferior rectus muscle entrapment
Complications:
Inferior rectus paresis
Enophthalmos
Compartment syndrome
Image by Gonzalez MO and Durairaj VD, made available under CC BY 2.0 license, accessed on 7/17/18 from https://openi.nlm.nih.gov/detailedresult.php?img=PMC2892129_MEAJO-17-138-g001&query=&req=4&npos=-1

## Slide 15
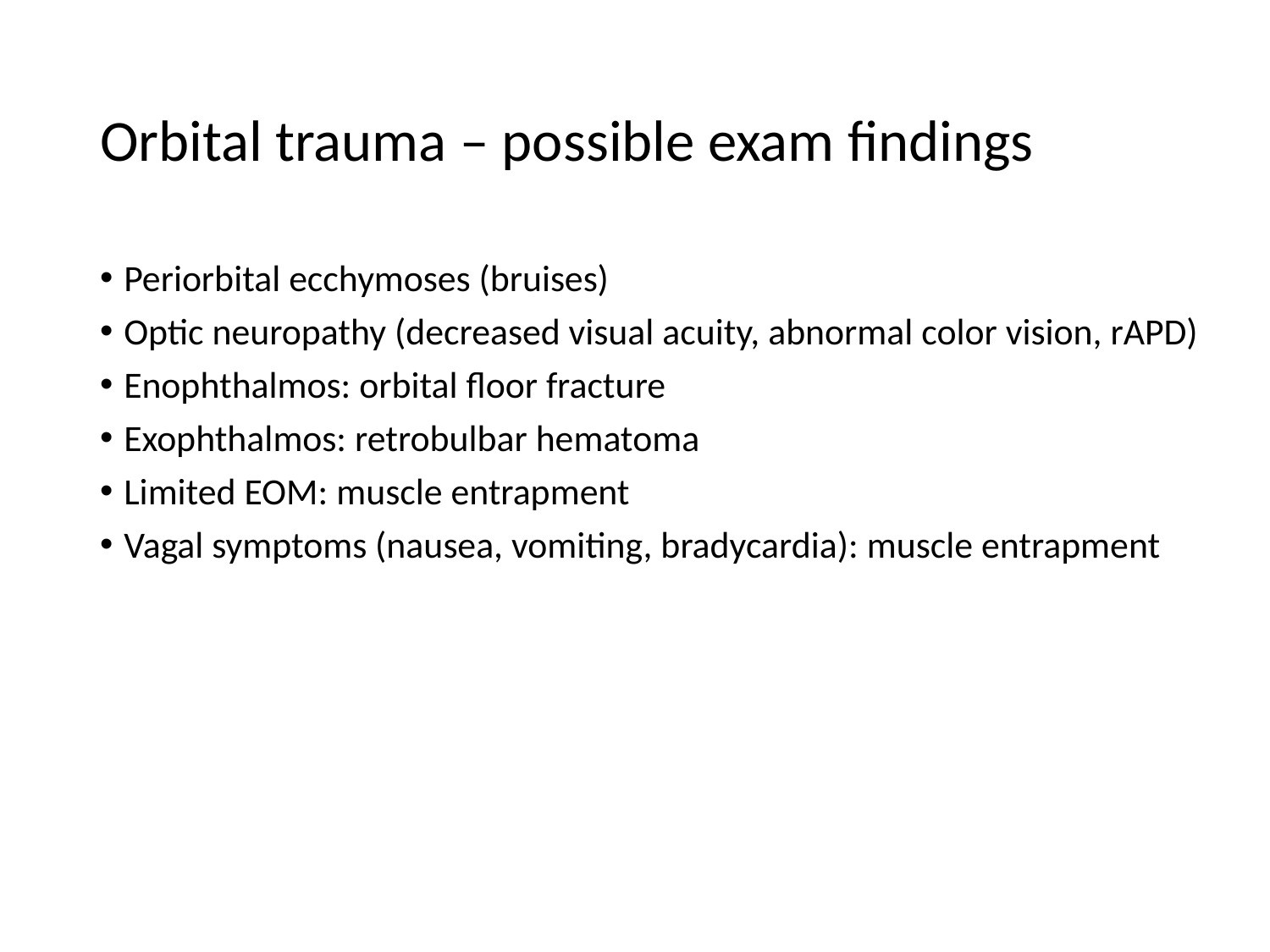

# Orbital trauma – possible exam findings
Periorbital ecchymoses (bruises)
Optic neuropathy (decreased visual acuity, abnormal color vision, rAPD)
Enophthalmos: orbital floor fracture
Exophthalmos: retrobulbar hematoma
Limited EOM: muscle entrapment
Vagal symptoms (nausea, vomiting, bradycardia): muscle entrapment

## Slide 16
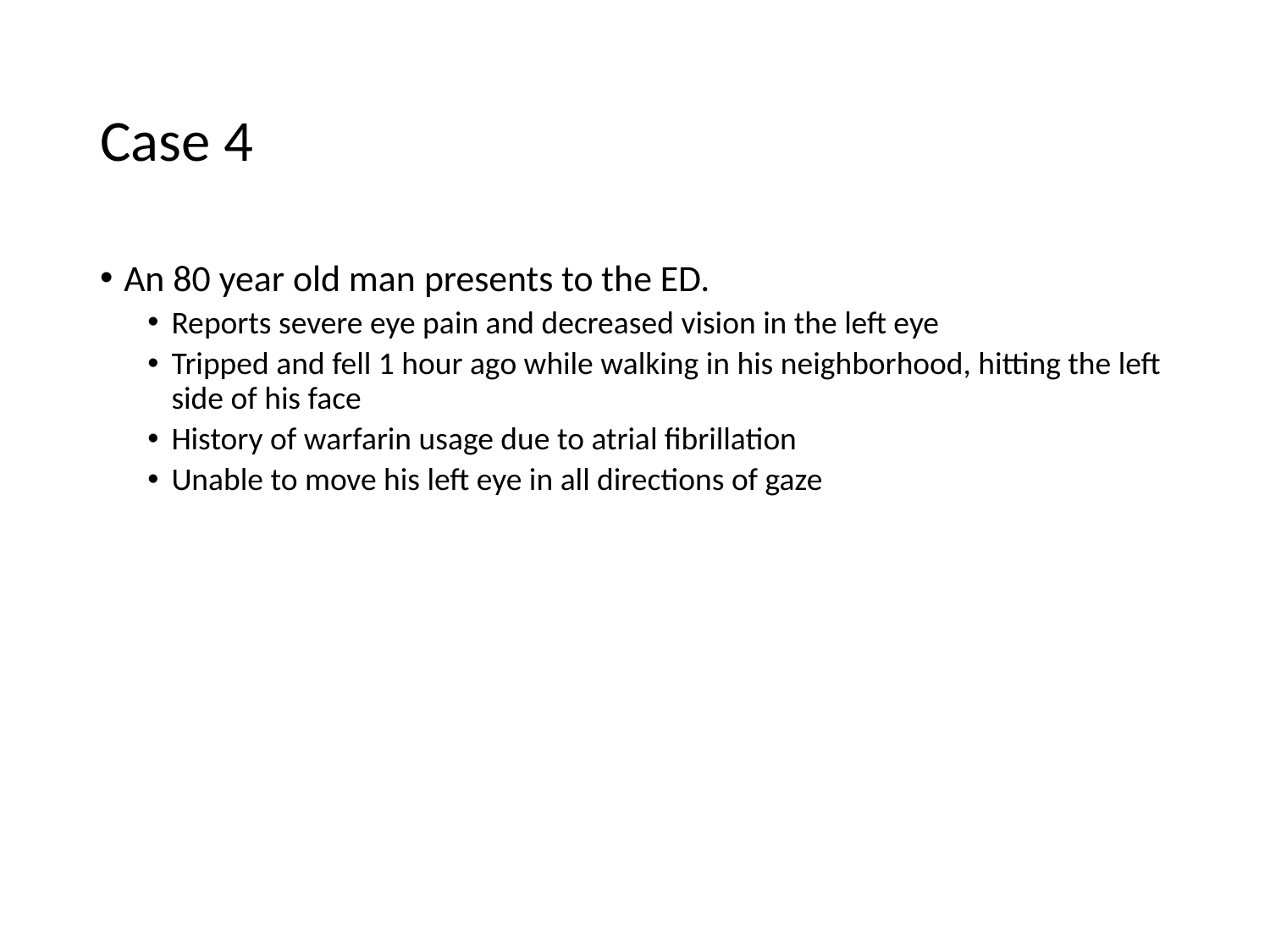

# Case 4
An 80 year old man presents to the ED.
Reports severe eye pain and decreased vision in the left eye
Tripped and fell 1 hour ago while walking in his neighborhood, hitting the left side of his face
History of warfarin usage due to atrial fibrillation
Unable to move his left eye in all directions of gaze

## Slide 17
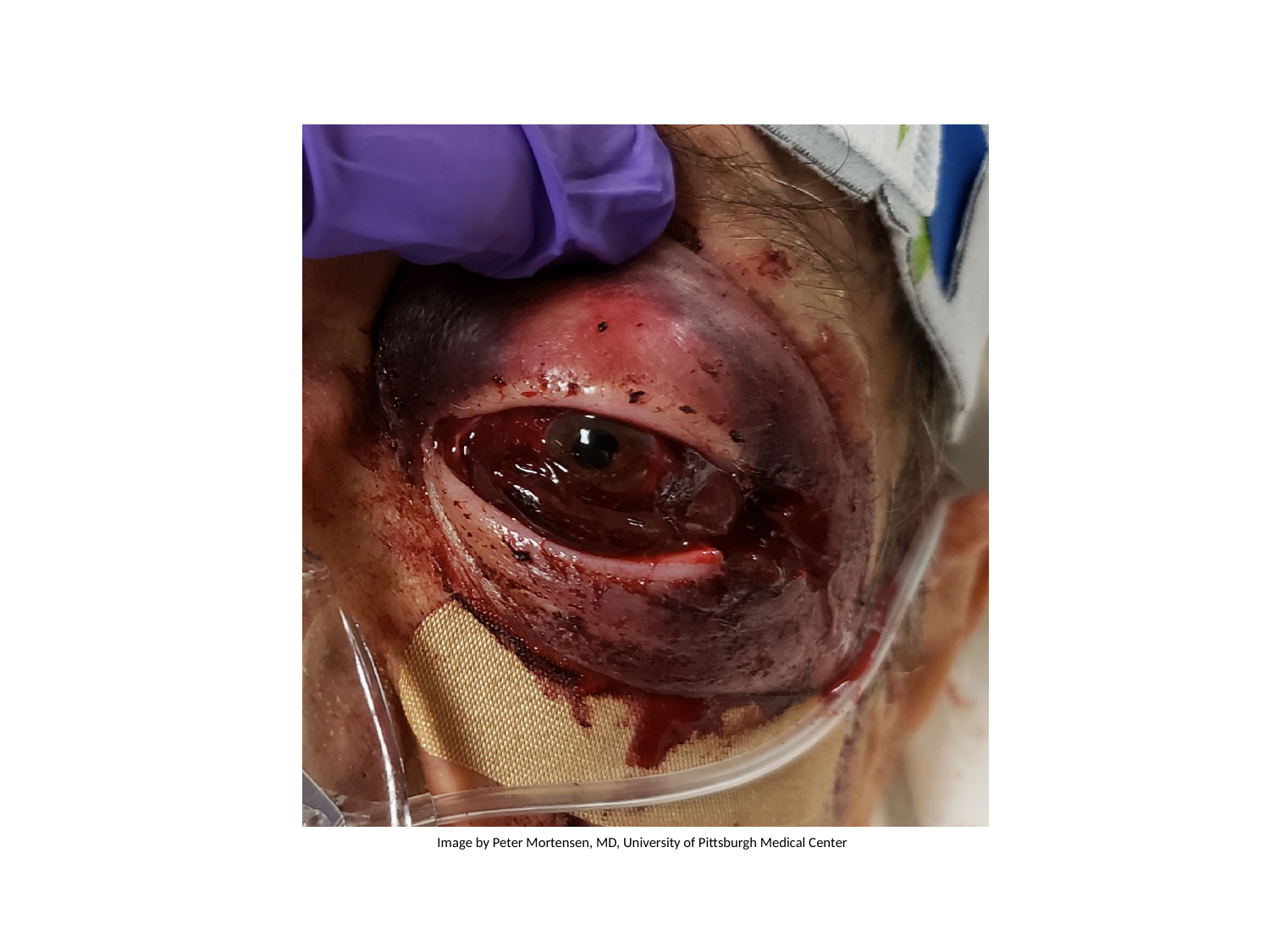

Image by Peter Mortensen, MD, University of Pittsburgh Medical Center

## Slide 18
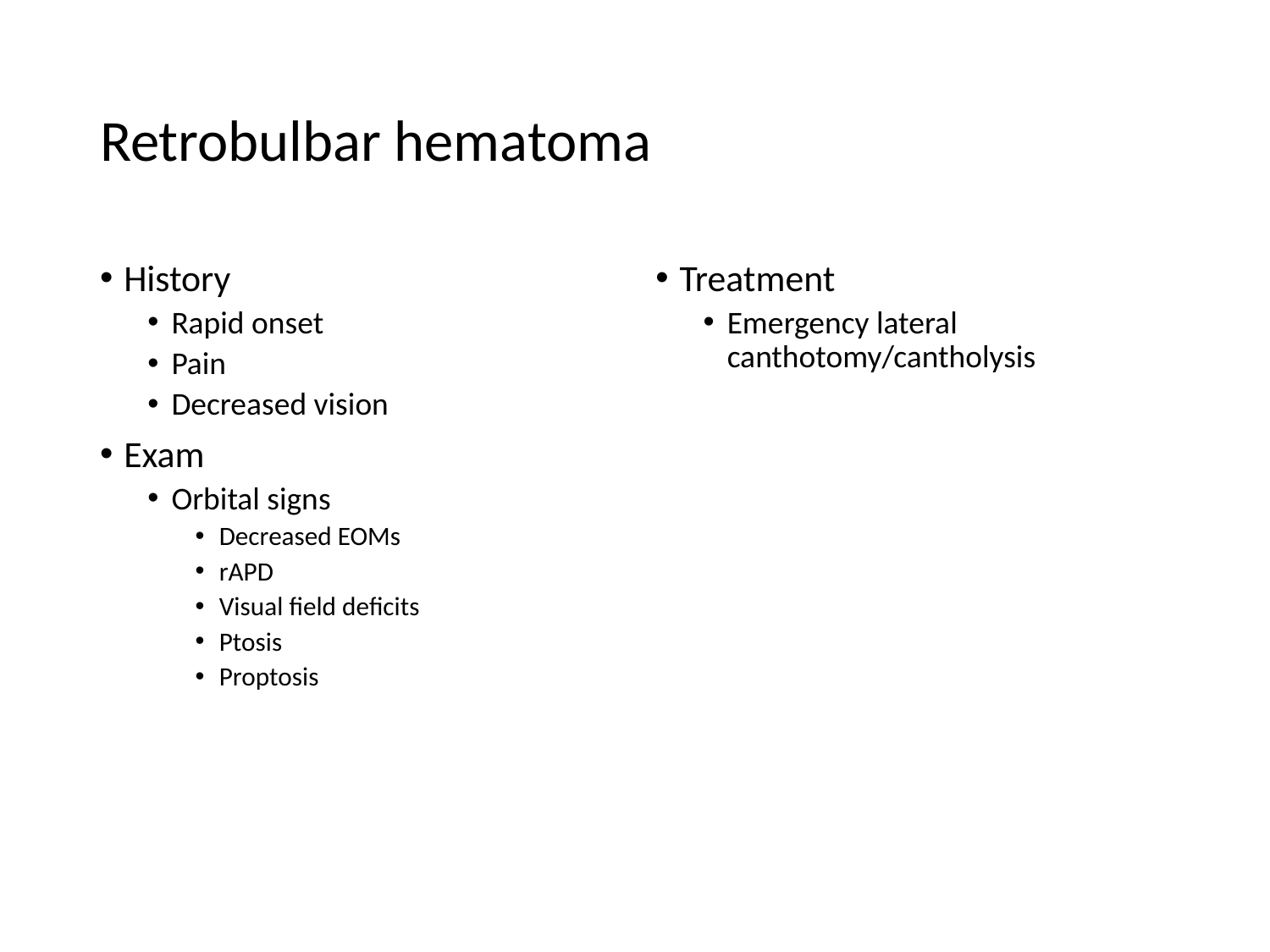

# Retrobulbar hematoma
History
Rapid onset
Pain
Decreased vision
Exam
Orbital signs
Decreased EOMs
rAPD
Visual field deficits
Ptosis
Proptosis
Treatment
Emergency lateral canthotomy/cantholysis

## Slide 19
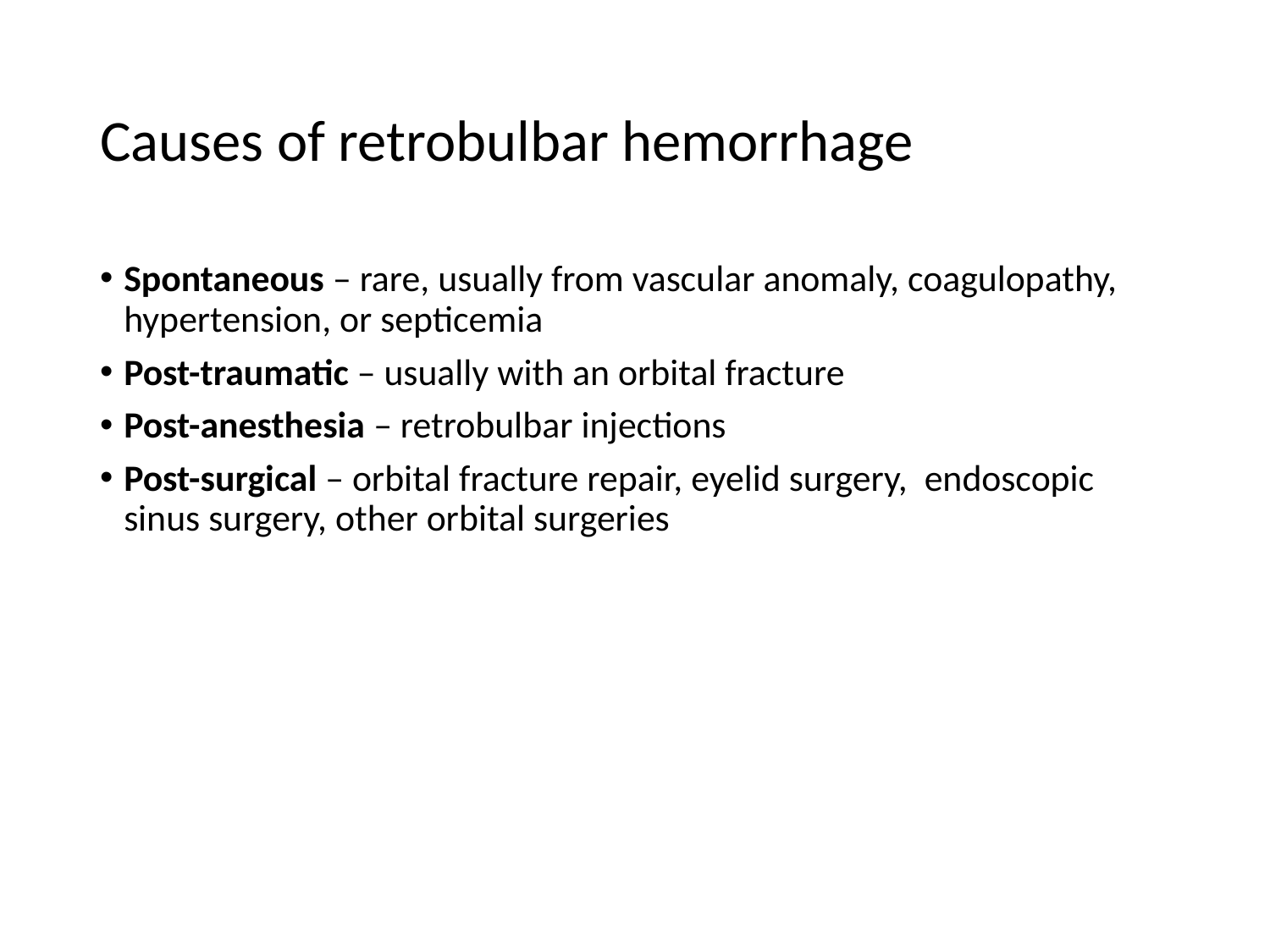

# Causes of retrobulbar hemorrhage
Spontaneous – rare, usually from vascular anomaly, coagulopathy, hypertension, or septicemia
Post-traumatic – usually with an orbital fracture
Post-anesthesia – retrobulbar injections
Post-surgical – orbital fracture repair, eyelid surgery, endoscopic sinus surgery, other orbital surgeries

## Slide 20
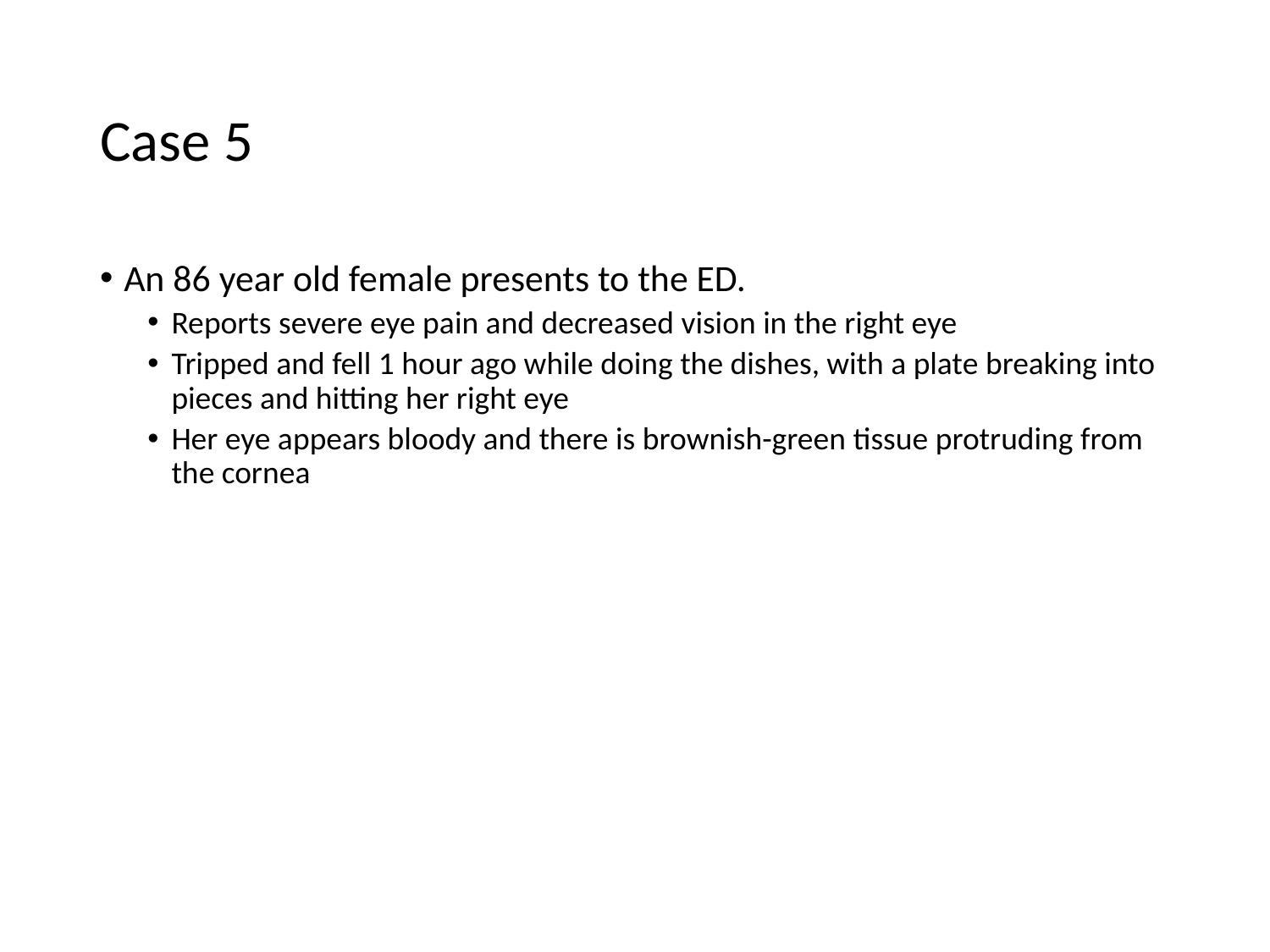

# Case 5
An 86 year old female presents to the ED.
Reports severe eye pain and decreased vision in the right eye
Tripped and fell 1 hour ago while doing the dishes, with a plate breaking into pieces and hitting her right eye
Her eye appears bloody and there is brownish-green tissue protruding from the cornea

## Slide 21
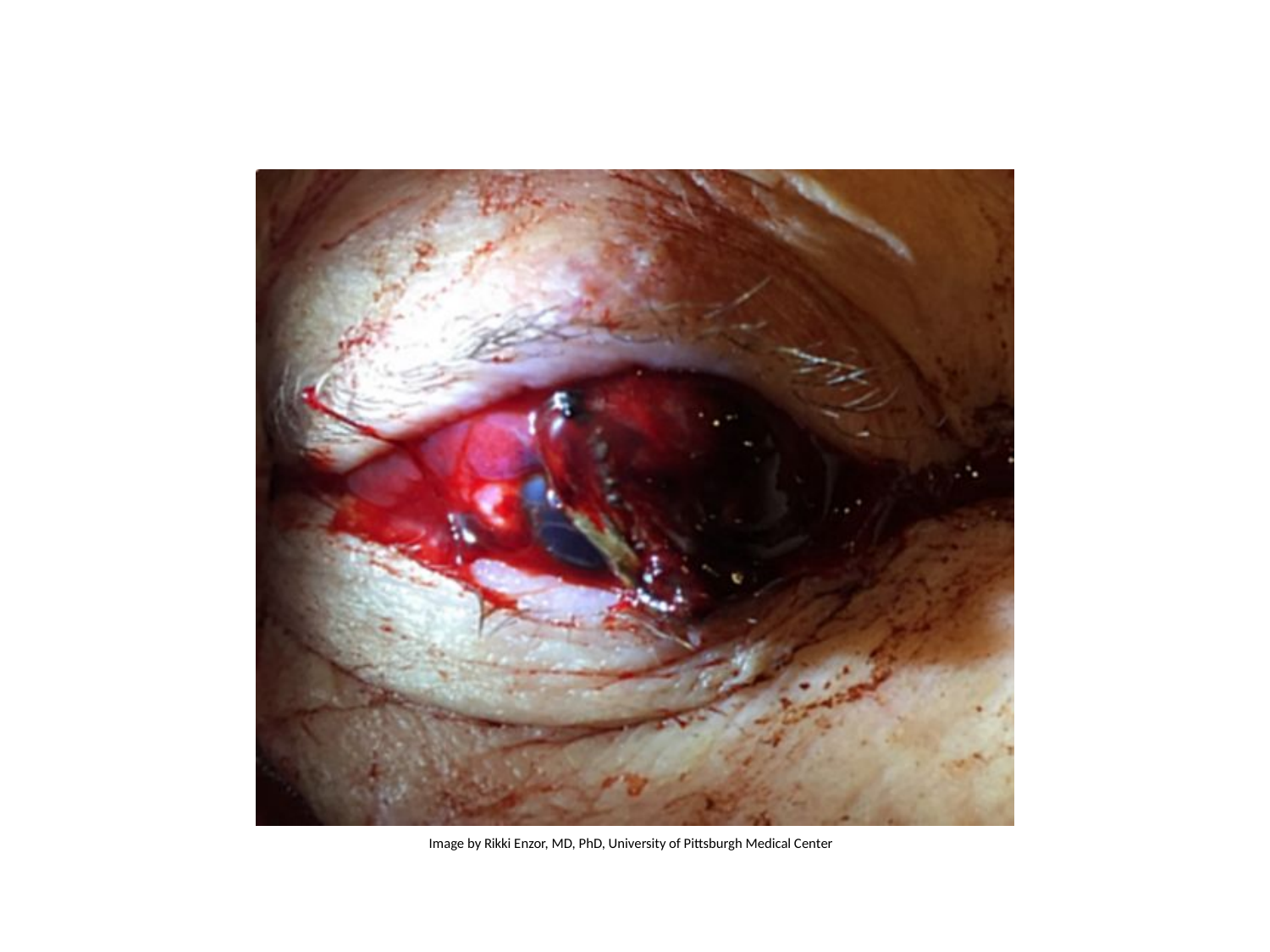

#
Image by Rikki Enzor, MD, PhD, University of Pittsburgh Medical Center

## Slide 22
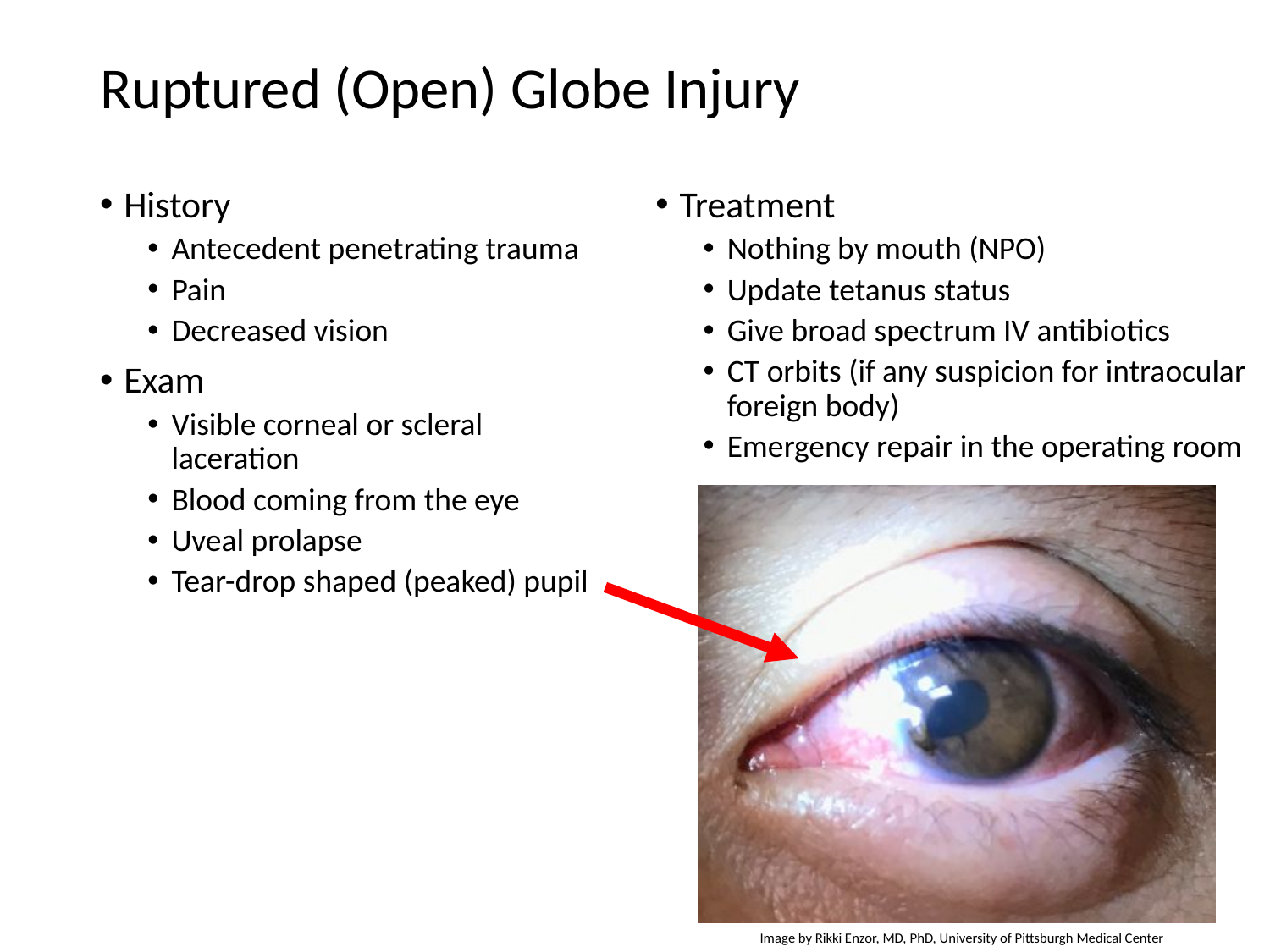

# Ruptured (Open) Globe Injury
History
Antecedent penetrating trauma
Pain
Decreased vision
Exam
Visible corneal or scleral laceration
Blood coming from the eye
Uveal prolapse
Tear-drop shaped (peaked) pupil
Treatment
Nothing by mouth (NPO)
Update tetanus status
Give broad spectrum IV antibiotics
CT orbits (if any suspicion for intraocular foreign body)
Emergency repair in the operating room
Image by Rikki Enzor, MD, PhD, University of Pittsburgh Medical Center

## Slide 23
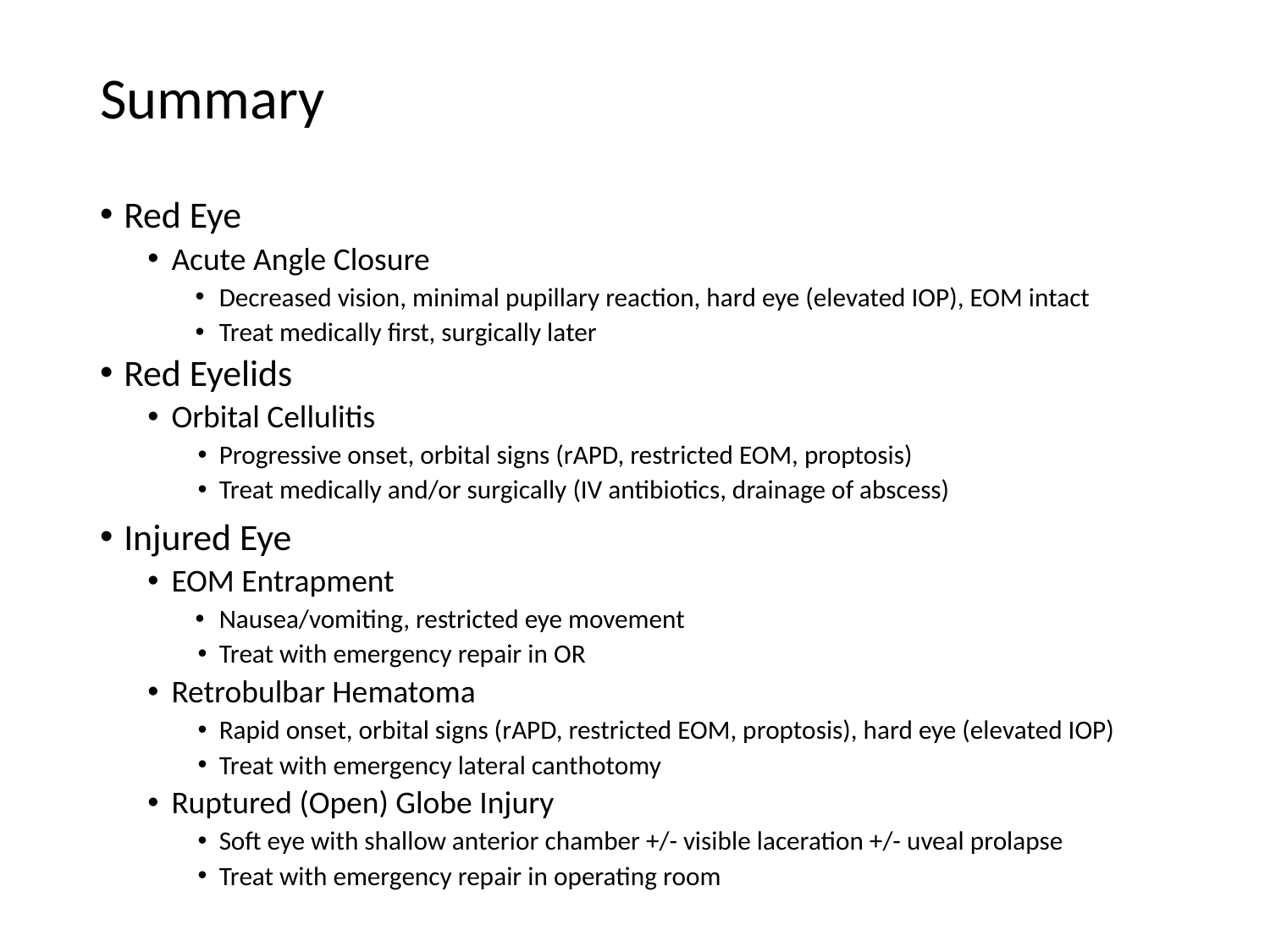

# Summary
Red Eye
Acute Angle Closure
Decreased vision, minimal pupillary reaction, hard eye (elevated IOP), EOM intact
Treat medically first, surgically later
Red Eyelids
Orbital Cellulitis
Progressive onset, orbital signs (rAPD, restricted EOM, proptosis)
Treat medically and/or surgically (IV antibiotics, drainage of abscess)
Injured Eye
EOM Entrapment
Nausea/vomiting, restricted eye movement
Treat with emergency repair in OR
Retrobulbar Hematoma
Rapid onset, orbital signs (rAPD, restricted EOM, proptosis), hard eye (elevated IOP)
Treat with emergency lateral canthotomy
Ruptured (Open) Globe Injury
Soft eye with shallow anterior chamber +/- visible laceration +/- uveal prolapse
Treat with emergency repair in operating room
