# Supplementary material for: The Ophthalmology Mini-Elective Gives Vision to Preclinical Medical Students
Source: MedEdPORTAL. 2020 Nov 23;16:11024. doi: 10.15766/mep_2374-8265.11024 (PMC7703479; doi:10.15766/mep_2374-8265.11024)
Supplement: Supplementary file 1 — Course Syllabus.docxInstructor Introduction.docxWeekly Course Time Line & Objectives.docxSession 1 - Intro to Ophthalmology.pptxSession 2 - Anterior Segment.pptxSession 3 - Posterior Segment.pptxSession 4 - Eye Emergencies and Trauma.pptxLaboratory Session Guide.pdfPrecourse Survey.docxPre- and Posttest.docxPostcourse Survey.docxPre- and Posttest Answers.docx [file mep_2374-8265.11024-s001.zip › H. Laboratory Session Guide.pdf]

Laboratory Session

Modified extracapsular cataract extraction

Learning Objectives

During this activity, students will learn to:
1. Use and work under the stereomicroscope.
2. Identify ophthalmic surgical instruments.
3. Exhibit proficiency with basic surgical movements and instrument use, including working in the anterior chamber safely.
4. Perform selected ophthalmic surgical techniques (e.g., corneal incisions, anterior capsulotomy, hydrodissection, and lens removal).

Step By Step Lab Exercise

1. Set up: Collect materials/instruments and set up the pig eye.

a) Materials needed per student:

- 1 pig eye, stereo dissecting microscope (okay if students have to share), Styrofoam base, plastic tray, sink (for obtaining water) or bottle of saline to share
- 27-gauge needle, hydrodissection cannula, 3-mL syringe, hemostat, viscoelastic in application syringe, paracentesis blade, small flexible plastic ruler (to use as a glide),

b) The pig eye should be placed into an indention in the Styrofoam base, which should then be taped to the plastic tray.

2. Make a bent needle cystatome: Using a hemostat, bend the tip of the 27-gauge needle downward away from bevel. Next, bend the proximal end of the needle at a 60-degree angle, as shown. Attach the cystatome to a 3-mL syringe. (See the diagram below.)

3. Create a paracentesis: A uniplanar paracentesis incision is created just anterior to the corneal limbus, radially positioned nearest the student. In cataract surgery, the surgeon and paracentesis incision are both generally positioned temporally.

4. Inject viscoelastic: Advance the tip of the viscoelastic cannula through the paracentesis, until it is just inside the anterior chamber. Inject viscoelastic to fill the anterior chamber, then withdraw from the eye.

5. Create a capsulotomy: Insert cystatome into the paracentesis incision (rotate sideways while entering), direct the point downward into the center of the anterior lens capsule, then drag to the side to

Making a bent needle cystatome

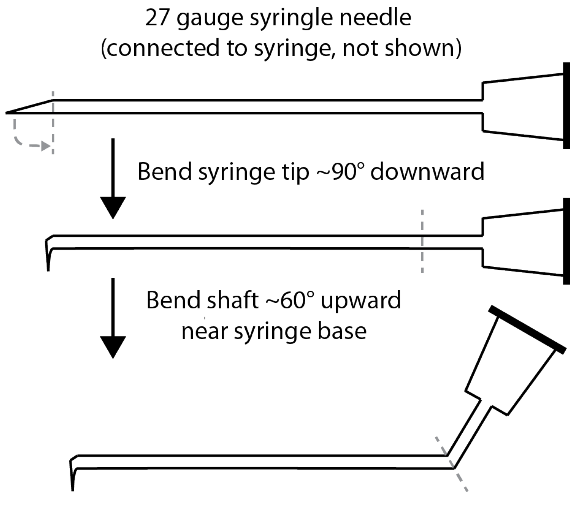

Illustration is author owned.

initiate a continuous curvilinear capsulorrhexis. Convert to can-opener technique by making a series of small radial tears around the edge of the anterior capsule, extending from the periphery into the center (see diagram). (Note: This is not a commonly used method of anterior capsulotomy, but it is easier to perform than a continuous capsulorhexis and has less risk of a tear extending to the posterior capsule.)
Video showing the technique (1min to 2:30min) can be found at:
Aravind Eye Hospital Pondicherry. Episode 2: Capsulotomy in MSICS [Video] Youtube. https://www.youtube.com/watch?v=caresWsYj08. Published 11/15/2019. Accessed 6/2/2020.

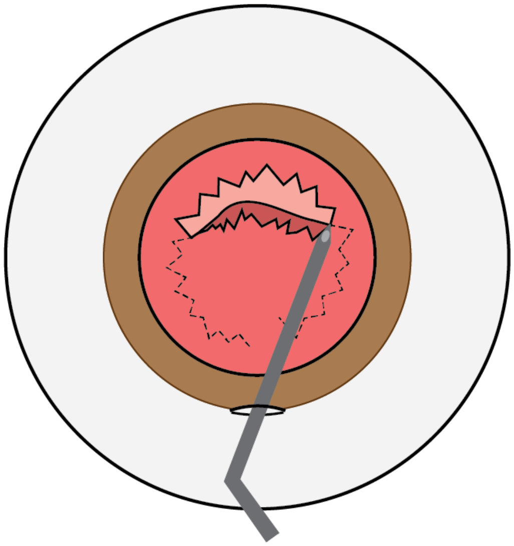

Can-opener capsulotomy
Illustration is author owned.

6. Hydro-dissect and prolapse the lens:
- Remove cystatome from syringe and replace it with the cannula. Remove the plunger, fill the syringe with water or saline, replace plunger.
- Insert dissection cannula under edge of capsule to the right and/or left side, with tip of cannula projecting slightly posteriorly
- Inject water or saline with constant pressure until fluid extends around the nucleus posteriorly. One pole of the nucleus will ideally prolapse into the anterior chamber after this.
- Place the cannula under the lens to lift the proximal end of the nucleus into the anterior chamber.

Video demonstrating hydro-dissection:
Aravind Eye Hospital Pondicherry. Episode 3:Hydro-procedures in MSICS [Video] Youtube. https://www.youtube.com/watch?v=k6vDAdEQWbU. Published 11/22/2019. Accessed 6/2/2020.

7. Expand the paracentesis: Make a large 8-9 mm corneal incision by inserting the paracentesis blade into the paracentesis incision, then rotating the distal tip outward and pulling it back out of the eye, to cut along either side of the corneal incision and enlarge it.

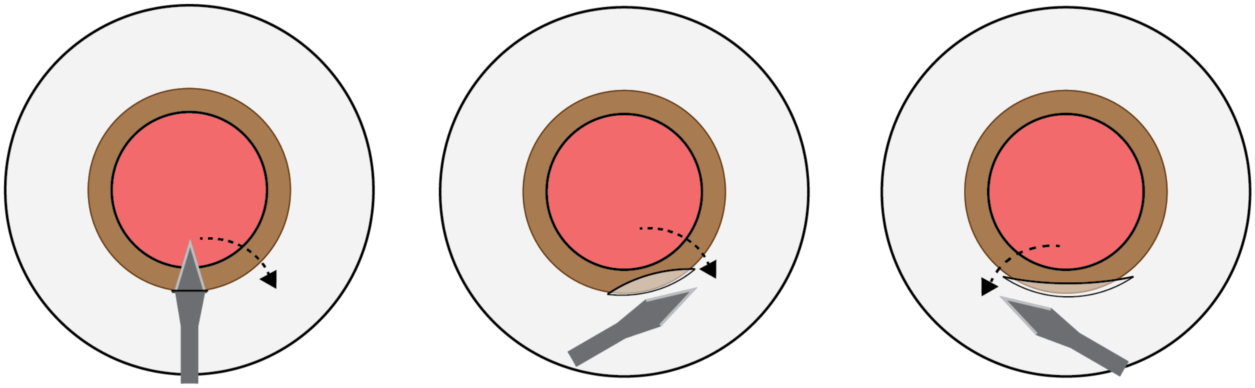

Illustration is author owned.

8. Express the lens: Place the end of the flexible plastic ruler into the eye and under the lens, then apply pressure at the opposite limbus to express the lens from the eye. The eye will collapse, but with enough pressure (and a large enough capsulotomy and corneal incision) the lens will exit the eye.

Congratulations on successfully completing your first cataract surgery!

9. Optional activity: Dissect the eye to look at major anatomical features, by using a straight razor blade to slice through the cornea straight down to the optic nerve to bisect the eye.
