# Supplementary material for: The Ophthalmology Mini-Elective Gives Vision to Preclinical Medical Students
Source: MedEdPORTAL. 2020 Nov 23;16:11024. doi: 10.15766/mep_2374-8265.11024 (PMC7703479; doi:10.15766/mep_2374-8265.11024)
Supplement: Supplementary file 1 — Course Syllabus.docxInstructor Introduction.docxWeekly Course Time Line & Objectives.docxSession 1 - Intro to Ophthalmology.pptxSession 2 - Anterior Segment.pptxSession 3 - Posterior Segment.pptxSession 4 - Eye Emergencies and Trauma.pptxLaboratory Session Guide.pdfPrecourse Survey.docxPre- and Posttest.docxPostcourse Survey.docxPre- and Posttest Answers.docx [file mep_2374-8265.11024-s001.zip › I. Precourse Survey.docx]

**Ophthalmology Mini-Elective – Pre-Course Survey**

1. Name as many conditions as you can in 30 seconds that may give you a red eye.
2. Glaucoma can be hereditary.

True False

1. The ophthalmology content in your 1st and 2nd year medical school classes is limited.

1 2 3 4 5

Strongly Somewhat Neutral Somewhat Strongly

Disagree Disagree Agree Agree

1. What is your background in Ophthalmology? Circle all that apply.

None Research Family Member Prior Rotation Prior Job Other (Describe)

1. How much experience do you have using ophthalmic diagnostic equipment?

1 2 3 4 5

Zero Limited Moderate Significant Very significant

Experience (slit lamp and indirect ophthalmoscopy)

1. What made you interested in Ophthalmology? Circle all that apply.

Lectures Someone recommended it I didn’t know anything about it

Prior rotation Research Guerilla Eye Service Other (Describe)

1. How interested are you in Ophthalmology as a career?

1 2 3 4 5

Not at all Minimally Moderately Very Set on ophthalmology as a career

Interested Interested Interested Interested

1. How related do you think Ophthalmology is to General Medicine?

1 2 3 4 5

Very Somewhat Neutral Somewhat Very

Unrelated Unrelated Related Related

1. How interested are you in doing surgery?

1 2 3 4 5

Not at all Minimally Moderately Very Set on a surgical specialty as a career

Interested Interested Interested Interested

1. How exciting is Ophthalmology to you?

1 2 3 4 5

Very Somewhat Neutral Somewhat Very

Boring Boring Exciting Exciting

11. How comfortable are you taking a basic general medicine history?

1 2 3 4 5

Very Somewhat Neutral Somewhat Very

Uncomfortable Uncomfortable Comfortable Comfortable

12. How comfortable are you taking a basic ophthalmic history?

1 2 3 4 5

Very Somewhat Neutral Somewhat Very

Uncomfortable Uncomfortable Comfortable Comfortable

13. How comfortable are you performing a basic general medicine physical examination?

1 2 3 4 5

Very Somewhat Neutral Somewhat Very

Uncomfortable Uncomfortable Comfortable Comfortable

14. How comfortable are you performing the ophthalmology component of the physical exam?

1 2 3 4 5

Very Somewhat Neutral Somewhat Very

Uncomfortable Uncomfortable Comfortable Comfortable

15. How would you rate your capabilities in presenting ophthalmology patients to an attending?

1 2 3 4 5

Very Somewhat Average Above Superior

Poor Poor Average

16. How would you rate your understanding of common eye problems?

1 2 3 4 5

Poor Superior

17. What are your personal goals for this mini-elective course?
