# Supplementary material for: The Ophthalmology Mini-Elective Gives Vision to Preclinical Medical Students
Source: MedEdPORTAL. 2020 Nov 23;16:11024. doi: 10.15766/mep_2374-8265.11024 (PMC7703479; doi:10.15766/mep_2374-8265.11024)
Supplement: Supplementary file 1 — Course Syllabus.docxInstructor Introduction.docxWeekly Course Time Line & Objectives.docxSession 1 - Intro to Ophthalmology.pptxSession 2 - Anterior Segment.pptxSession 3 - Posterior Segment.pptxSession 4 - Eye Emergencies and Trauma.pptxLaboratory Session Guide.pdfPrecourse Survey.docxPre- and Posttest.docxPostcourse Survey.docxPre- and Posttest Answers.docx [file mep_2374-8265.11024-s001.zip › J. Pre- and Posttest.docx]

**Ophthalmology Mini Elective Pre-Test/Post-Test**

1. A college student presents to the ER on Saturday morning with “a bright red spot on the white part of my right eye.” He reports drinking a large amount of alcohol the night prior followed by multiple episodes of vomiting. He reports no significant vision changes or eye pain. Which of the following is the most likely diagnosis?
   1. Hyphema
   2. Subconjunctival hemorrhage
   3. Conjunctivitis
   4. Corneal abrasion
2. A patient presents with a corneal abrasion. Which of the following is NEVER an acceptable option in the treatment of corneal abrasion?
   1. Atropine
   2. Erythromycin
   3. Proparacaine
   4. Moxifloxacin
3. A 23 year old female taking oral contraceptives presents with headaches, pulsatile tinnitus, and blurred vision in both eyes. Which of the following would NOT be high on your differential?
   1. Viral meningitis
   2. Idiopathic intracranial hypertension (pseudotumor cerebri)
   3. Migraine
   4. Dural venous sinus thrombosis
4. What value is often considered to be the upper limit of normal for intraocular pressure?
   1. 18 mm Hg
   2. 21 mm Hg
   3. 27 mm Hg
   4. 30 mm Hg
5. A 7-year-old boy presents with a grossly swollen eyelid. What feature is more consistent with preseptal cellulitis rather than orbital cellulitis?
   1. Warmth and erythema of the eyelids
   2. Pain with eye movements
   3. Proptosis
   4. Decreased vision
6. A patient presents with flashing lights, floaters, and dark curtain-like sensation across his vision starting yesterday in his right eye. Which of the following is the most likely diagnosis?
   1. Retinal detachment
   2. Acute angle closure glaucoma
   3. Migraine with visual aura
   4. Amaurosis fugax
7. Where does aqueous exit the eye?
   1. Anterior chamber into the corneal wedge
   2. Vitreous chamber into the emissary veins
   3. Posterior chamber into the ciliary body
   4. Trabecular meshwork into the canal of Schlemm
8. A 47 year old man presents with worsening vision at near over the past 3-4 years. He reports that his distance vision seems relatively unchanged. What is the most likely diagnosis?
   1. Presbyopia
   2. Hyperopia
   3. Cataract
   4. Dry eyes
9. On examination, a patient is noted to have anisocoria (difference in pupil size). Suppose that his pupils in the light are 3 mm in the right eye and 4 mm in the left eye. In the dark, his right pupil is 6 mm. Assuming that his anisocoria is physiologic, how large would his left pupil be in the dark?
   1. 5 mm
   2. 6 mm
   3. 7 mm
   4. Anisocoria is never physiologic
10. What type of retinal detachment most often occurs due to proliferative diabetic retinopathy?
    1. Serous
    2. Tractional
    3. Exudative
    4. Rhegmatogenous
11. Which of the following must be present for a diagnosis of glaucoma?
    1. Damage to the optic nerve
    2. Decline in visual acuity
    3. Elevated intraocular pressure
    4. Afferent pupillary defect
12. A patient in the ICU has a fixed, dilated pupil. What must you suspect?
    1. Horner’s syndrome due to carotid injury
    2. Optic nerve compression due to increased intracranial pressure
    3. Cranial nerve III palsy due to uncal herniation
    4. Cranial nerve VI palsy due to increased intracranial pressure
13. Which of the following is the biggest risk factor for acute angle closure?
    1. Asian ancestry
    2. Myopia
    3. Thin corneas
    4. Large optic discs
14. In the absence of lens accommodation, a hyperopic eye focuses images where?
    1. Behind the lens
    2. Behind the retina
    3. In front of the lens
    4. In front of the retina
15. Which conjunctivitis is typically unilateral and causes prominent discharge?
    1. Viral
    2. Bacterial
    3. Allergic
    4. Vernal
