# Supplementary material for: The Ophthalmology Mini-Elective Gives Vision to Preclinical Medical Students
Source: MedEdPORTAL. 2020 Nov 23;16:11024. doi: 10.15766/mep_2374-8265.11024 (PMC7703479; doi:10.15766/mep_2374-8265.11024)
Supplement: Supplementary file 1 — Course Syllabus.docxInstructor Introduction.docxWeekly Course Time Line & Objectives.docxSession 1 - Intro to Ophthalmology.pptxSession 2 - Anterior Segment.pptxSession 3 - Posterior Segment.pptxSession 4 - Eye Emergencies and Trauma.pptxLaboratory Session Guide.pdfPrecourse Survey.docxPre- and Posttest.docxPostcourse Survey.docxPre- and Posttest Answers.docx [file mep_2374-8265.11024-s001.zip › K. Postcourse Survey.docx]

**Ophthalmology Mini-Elective – Post-Course Survey**

1. Name as many conditions as you can in 30 seconds that may give you a red eye.
2. Glaucoma can be hereditary.

True False

1. How interested are you in Ophthalmology as a career?

1 2 3 4 5

Not at all Minimally Moderately Very Set on ophthalmology as a career

Interested Interested Interested Interested

1. How related do you think Ophthalmology is to General Medicine?

1 2 3 4 5

Very Somewhat Neutral Somewhat Very

Unrelated Unrelated Related Related

1. How exciting is Ophthalmology to you?

1 2 3 4 5

Very Somewhat Neutral Somewhat Very

Boring Boring Exciting Exciting

6. How comfortable are you taking a basic ophthalmic history?

1 2 3 4 5

Very Somewhat Neutral Somewhat Very

Uncomfortable Uncomfortable Comfortable Comfortable

7. How comfortable are you performing the ophthalmology component of the physical exam?

1 2 3 4 5

Very Somewhat Neutral Somewhat Very

Uncomfortable Uncomfortable Comfortable Comfortable

8. How would you rate your capabilities in presenting ophthalmology patients to an attending?

1 2 3 4 5

Very Somewhat Average Above Superior

Poor Poor Average

9. How would you rate your understanding of common eye problems?

1 2 3 4 5

Very Somewhat Average Above Superior

Poor Poor Average

10. Was the course well organized?

1 2 3 4 5

Extremely Somewhat Average Well- Very Well-

Disorganized Disorganized Organized Organized

11. Was the course taught at an appropriate level for your level of experience?

1 2 3 4 5

Not at all Minimally Somewhat To a Absolutely

significant

degree

12. Did this mini-elective reach your expectations?

1 2 3 4 5

Not at all Minimally Somewhat To a Absolutely

significant

degree

13. Instructors treated me and others with respect:

1 2 3 4 5

Strongly Somewhat Neutral Somewhat Strongly

Disagree Disagree Agree Agree

14. Overall quality of the course:

1 2 3 4 5

Very Somewhat Average Above Superior

Poor Poor Average

15. What components of this course should there be less of? What other changes would you make?

16. What components of this course should there be more of? What helped you to learn?

17. General comments and evaluations of specific instructors:
