# Supplementary material for: The Ophthalmology Mini-Elective Gives Vision to Preclinical Medical Students
Source: MedEdPORTAL. 2020 Nov 23;16:11024. doi: 10.15766/mep_2374-8265.11024 (PMC7703479; doi:10.15766/mep_2374-8265.11024)
Supplement: Supplementary file 1 — Course Syllabus.docxInstructor Introduction.docxWeekly Course Time Line & Objectives.docxSession 1 - Intro to Ophthalmology.pptxSession 2 - Anterior Segment.pptxSession 3 - Posterior Segment.pptxSession 4 - Eye Emergencies and Trauma.pptxLaboratory Session Guide.pdfPrecourse Survey.docxPre- and Posttest.docxPostcourse Survey.docxPre- and Posttest Answers.docx [file mep_2374-8265.11024-s001.zip › L. Pre- and Posttest Answers.docx]

**Ophthalmology Mini Elective Pre-Test/Post-Test Answer Sheet**

1. A college student presents to the ER on Saturday morning with “a bright red spot on the white part of my right eye.” He reports drinking a large amount of alcohol the night prior followed by multiple episodes of vomiting. He reports no significant vision changes or eye pain. Which of the following is the most likely diagnosis?
   1. Hyphema
   2. **Subconjunctival hemorrhage**
   3. Conjunctivitis
   4. Corneal abrasion

Hyphema = blood in the anterior chamber
Subconjunctival hemorrhage = blood on the white part of the eye
Conjunctivitis = inflammation (redness) of the mucous membrane lining over the white part of the eye, spares the limbus (unlike more serious causes of ocular redness)
Corneal abrasion = scratch on the cornea

1. A patient presents with a corneal abrasion. Which of the following is NEVER an acceptable option in the treatment of corneal abrasion?
   1. Atropine
   2. Erythromycin
   3. **Proparacaine**
   4. Moxifloxacin

Atropine = dilating/cycloplegic drop (bottle has a red cap), may be used for comfort in patient with iritis (inflammation of the iris, inflammatory cells are seen floating in the anterior chamber on exam), sometimes also used for comfort in a patient with a large corneal abrasion
Erythromycin = macrolide antibiotic, typically in ointment form, frequently used for patients with corneal abrasions
Proparacaine = topical anesthetic, NEVER give this to a patient
Moxifloxacin = fluoroquinolone antibiotic drop (bottle has a tan cap), may be used for patients with corneal abrasions, fluoroquinolone antibiotic should be used (rather than erythromycin) for patient with corneal abrasion involving vegetable material (e.g., dirt, soil, garden rake, tree branch, etc) due to increased risk of Gram negative rods

1. A 23 year old female taking oral contraceptives presents with headaches, pulsatile tinnitus, and blurred vision in both eyes. Which of the following would NOT be high on your differential?
   1. Viral meningitis
   2. Idiopathic intracranial hypertension (pseudotumor cerebri)
   3. **Migraine**
   4. Dural venous sinus thrombosis

When a patient presents with headaches, pulsatile tinnitus, and blurred vision, one should be concerned for increased intracranial pressure (ICP). The DDx for increased intracranial pressure includes infectious (e.g., viral or bacterial meningitis – think lyme, syphilis, and herpes viruses), autoimmune/inflammatory (e.g., lupus, Sjogren’s, sarcoidosis), malignant (e.g., GBM, primary CNS lymphoma, mets, others), thrombotic (i.e., dural venous sinus thrombosis). Idiopathic intracranial hypertension (pseudotumor cerebri) is a diagnosis of exclusion, which can only be made after all of the other possible etiologies have been ruled out.
While migraine is a very common cause of headaches among ED patients, presence of pulsatile tinnitus and blurred vision should raise concern for increased intracranial pressure. Also, the patient has risk factors for IIH (e.g., young obese female) and dural venous sinus thrombosis (e.g., taking oral contraceptives).

1. What value is often considered to be the upper limit of normal for intraocular pressure?
   1. 18 mm Hg
   2. **21 mm Hg**
   3. 27 mm Hg
   4. 30 mm Hg

The normal range for IOP (intraocular pressure) is 8-21 mm Hg.

1. A 7-year-old boy presents with a grossly swollen eyelid. What feature is more consistent with preseptal cellulitis rather than orbital cellulitis?
   1. **Warmth and erythema of the eyelids**
   2. Pain with eye movements
   3. Proptosis
   4. Decreased vision

Orbital signs include decreased vision, a relative afferent pupillary defect, pain with eye movements, restricted eye movements, and proptosis. These should raise concern for orbital cellulitis.
The DDx for a patient with a swollen eyelid includes both preseptal and orbital cellulitis.

1. A patient presents with flashing lights, floaters, and dark curtain-like sensation across his vision starting yesterday in his right eye. Which of the following is the most likely diagnosis?
   1. **Retinal detachment**
   2. Acute angle closure glaucoma
   3. Migraine with visual aura
   4. Amaurosis fugax

Retinal detachment = flashing lights, floaters, and dark curtain progressing across the vision
Acute angle closure glaucoma = pain, redness, blurred vision, nausea/vomiting, and colored haloes around lights
Migraine = HA (usually unilateral throbbing), nausea/vomiting, photophobia, phonophobia, may be preceded by 20-30 minutes of photopsias (flashing lights, shimmering of objects, blurred vision) which are called visual aura
Amaurosis fugax = transient episodes of decreased vision, typically in one eye, due to decreased blood flow, may be a warning sign of something more serious (e.g., impending blindness from giant cell arteritis)

1. Where does aqueous exit the eye?
   1. Anterior chamber into the corneal wedge
   2. Vitreous chamber into the emissary veins
   3. Posterior chamber into the ciliary body
   4. **Trabecular meshwork into the canal of Schlemm**

Aqueous is produced in the posterior chamber (between the iris and lens) by the ciliary body and travels forward through the pupil into the anterior chamber (between the cornea and iris). Once it reaches the anterior chamber, the aqueous drains peripherally in the iridocorneal angle, which exists in a circular ring located at the edge of the cornea and iris. Within the iridocorneal angle, the aqueous drains through a structure called the trabecular meshwork into the canal of Schlemm, which ultimately feeds into the venous system.

1. A 47 year old man presents with worsening vision at near over the past 3-4 years. He reports that his distance vision seems relatively unchanged. What is the most likely diagnosis?
   1. **Presbyopia**
   2. Hyperopia
   3. Cataract
   4. Dry eyes

Presbyopia = worsening near vision, typically around age 45-50, due to loss of accommodation
Hyperopia = far sightedness, often due to a small eye, with light rays focusing behind the retina
Cataract = worsening vision at distance and near, often associated with complaint of glare with headlights especially when driving at night or in the rain, due to changes in the structure of the lens often with aging
Dry eyes = very common, causes transient episodes of blurred vision which resolve with blinking or closing the eyes

1. On examination, a patient is noted to have anisocoria (difference in pupil size). Suppose that his pupils in the light are 3 mm in the right eye and 4 mm in the left eye. In the dark, his right pupil is 6 mm. Assuming that his anisocoria is physiologic, how large would his left pupil be in the dark?
   1. 5 mm
   2. 6 mm
   3. **7 mm**
   4. Anisocoria is never physiologic

Physiologic anisocoria is a difference in pupil size, which is the same in bright and dark lighting conditions. It occurs in 20% of patients and typically has a difference of 1 mm or less between the two eyes.
Horner’s syndrome = miosis, ptosis, anhidrosis. (Miosis = abnormally small pupil.)
Cranial Nerve III palsy = mydriasis, ptosis, eye infraducted and abducted (AKA “down and out”). (Mydriasis = abnormally large pupil).

1. What type of retinal detachment most often occurs due to proliferative diabetic retinopathy?
   1. Serous
   2. **Tractional**
   3. Exudative
   4. Rhegmatogenous

Serous and exudative retinal detachment are the same thing and occur when inflammation causes fluid to leak under the retina, in the absence of a retinal break.
Tractional retinal detachment occurs with formation and contraction of fibrovascular membranes, in the setting of diabetic retinopathy.
Rhegmatogenous retinal detachment occurs when fluid gets under the retina in the setting of a retinal tear.

1. Which of the following must be present for a diagnosis of glaucoma?
   1. **Damage to the optic nerve**
   2. Decline in visual acuity
   3. Elevated intraocular pressure
   4. Afferent pupillary defect

Glaucoma is a progressive optic neuropathy with characteristic nerve changes including cupping and characteristic visual field changes including peripheral nasal and arcuate defects. While glaucoma is pressure-dependent, it may develop even in patients with an intraocular pressure that always measures within the normal range. Visual acuity frequently remains normal until very late in the disease course, as peripheral vision is affected first. An afferent pupillary defect likewise may develop very late in the disease course (if the glaucoma is very asymmetric) or not at all (since glaucoma typically affects both eyes).

1. A patient in the ICU has a fixed, dilated pupil. What must you suspect?
   1. Horner’s syndrome due to carotid injury
   2. Optic nerve compression due to increased intracranial pressure
   3. **Cranial nerve III palsy due to uncal herniation**
   4. Cranial nerve VI palsy due to increased intracranial pressure

Horner’s syndrome = miosis, ptosis, anhidrosis
Increased intracranial pressure = HA, blurred vision, pulsatile tinnitus (whooshing noise or heartbeat sound in ears), sometimes restricted abduction due to cranial nerve VI palsy
Cranial nerve III palsy = mydriasis, ptosis, eye infraducted and abducted (AKA “down and out”).

1. Which of the following is the biggest risk factor for acute angle closure?
   1. **Asian ancestry**
   2. Myopia
   3. Thin corneas
   4. Large optic discs

Risk factors for angle closure glaucoma include hyperopia, female sex, Asian or Inuit ancestry, positive family history, medications (especially topiramate), and lens dislocation.

1. In the absence of lens accommodation, a hyperopic eye focuses images where?
   1. Behind the lens
   2. **Behind the retina**
   3. In front of the lens
   4. In front of the retina

A hyperopic eye (far sighted, smaller eye with short axial length) focuses images behind the retina.
A myopic eye (near sighted, bigger eye with long axial length) focuses images in front of the retina.

1. Which conjunctivitis is typically unilateral and causes prominent discharge?
   1. Viral
   2. **Bacterial**
   3. Allergic
   4. Vernal

Bacterial conjunctivitis = typically unilateral, prominent mucopurulent discharge
Viral conjunctivitis = may start unilateral but frequently spreads to the other eye, typically less discharge than bacterial, associated with viral URI symptoms (patient reports having a recent cold)
Allergic conjunctivitis = bilateral, symmetric, itching and tearing, associated with seasonal allergies
Vernal conjunctivitis = a subset of allergic conjunctivitis in which symptomatology peaks in the early teens and tends to improve or resolve by age 30; tends to affect the superior > inferior tarsus (upper > lower eyelid)
